# Argonaute 2 drives miR-145-5p-dependent gene expression program in breast cancer cells

**DOI:** 10.1038/s41419-018-1267-5

**Published:** 2019-01-08

**Authors:** Teresa Bellissimo, Claudia Tito, Federica Ganci, Andrea Sacconi, Silvia Masciarelli, Giuseppe Di Martino, Natale Porta, Mirko Cirenza, Melissa Sorci, Luciana De Angelis, Paolo Rosa, Antonella Calogero, Alessandro Fatica, Vincenzo Petrozza, Giulia Fontemaggi, Giovanni Blandino, Francesco Fazi

**Affiliations:** 1 Department of Anatomical, Histological, Forensic & Orthopedic Sciences, Section of Histology & Medical Embryology, Sapienza University of Rome, laboratory affiliated to Istituto Pasteur Italia-Fondazione Cenci Bolognetti, Rome, Italy; 2 Oncogenomic and Epigenetic Unit, IRCCS Regina Elena National Cancer Institute, Rome, Italy; 3 Department of Medico-Surgical Sciences and Biotechnologies, Sapienza University of Rome, Pathology Unit, ICOT, Latina, Italy; 4 Department of Biology and Biotechnology “C. Darwin”, Sapienza University of Rome, Rome, Italy

## Abstract

To perform their regulatory functions, microRNAs (miRNAs) must assemble with any of the four mammalian Argonaute (Ago) family of proteins, Ago1–4, into an effector complex known as the RNA-induced silencing complex (RISC). While the mature miRNA guides the RISC complex to its target mRNA, the Ago protein represses mRNA translation. The specific roles of the various Ago members in mediating miRNAs activity, however, haven’t been clearly established. In this study, we investigated the contribution of Ago2, the only human Ago protein endowed with nuclease activity, to the function of tumor-suppressor miR-145-5p in breast cancer (BC). We show that miR-145-5p and Ago2 protein are concomitantly downregulated in BC tissues and that restoration of miR-145-5p expression in BC cells leads to Ago2 protein induction through the loosening of Ago2 mRNA translational repression. Functionally, miR-145-5p exerts its inhibitory activity on cell migration only in presence of Ago2, while, upon Ago2 depletion, we observed increased miR-145/Ago1 complex and enhanced cell motility. Profiling by microarray of miR-145-5p target mRNAs, in BC cells depleted or not of Ago2, revealed that miR-145-5p drives Ago2-dependent and -independent activities. Our results highlight that the Ago2 protein in cancer cells strictly dictates miR-145-5p tumor suppressor activity.

## Introduction

MicroRNAs (miRNAs) are small non-coding RNAs able to regulate gene expression at post-transcriptional level. They control cell fate decision in several developmental programs and their deregulation has been associated with tumor progression^1^. It has been extensively demonstrated that miRNAs may behave as oncogenes or tumor suppressors; indeed, their altered expression during tumorigenesis can promote or inhibit cancer progression. MiRNAs are considered a relevant hallmark of cancer and their clinical use to assess patient outcome, to monitor progression and also for therapeutic purpose in cancer, is rapidly emerging^1^.

To perform their regulatory functions, miRNAs must assemble with any of the four mammalian Argonaute (Ago) family of proteins, Ago1–4, into an effector complex known as the RNA-induced silencing complex (RISC)^2^. The miRNAs loaded in the RISC complex usually function as negative regulators of gene expression, mainly by targeting the 3’-untranslated region (UTR) of their target mRNAs. In general, while the mature miRNA guides the RISC complex to its target mRNA, the Ago protein complex represses mRNA translation or induces deadenylation-dependent mRNA decay, leading to silencing of gene expression^2–4^. Of note, despite a remarkable homology that extends to the PIWI domains among all four human Ago proteins, Argonaute2 (Ago2) is the only human Ago protein endowed with nuclease activity^5–11^.

As for miRNAs, also Ago2 deregulation has been linked to cancer progression and treatment^12–14^. A number of studies have shown Ago2 modulation also in breast carcinoma, providing contradictory results. For example Kwon et al.^15^ and Blenkiron et al.^16^ showed, respectively, down- and up-regulation of Ago2 mRNA in BC. With concern to Ago2 function, Ago2 has been related to miRNA-dependent tumor suppression as well as to pro-tumoral functions, and the level of expression of specific miRNAs may be an important determinant for Ago2 function^17^.

MiR-145-5p represents one of the miRNAs with tumor suppressor function that is highly expressed in normal tissues and downregulated in several human cancers, including colorectal and breast cancer^18^. miR-145-5p is located at chromosome 5q33.1 and is usually transcribed in a bicistronic primary transcript with miR-143. The epigenetic silencing of this region or the deletion of this well known fragile site are common events linked to cancer phenotype^19–21^. Of note, the downregulation of miR-145-5p expression is associated to the deregulation of several biological processes, such as cell proliferation, cell migration, invasiveness, and chemoresistance^18–21^. In lung cancer, for example, the restoration of miR-145-5p expression reduces cell proliferation and tumor growth targeting both EGFR and NUDT1^22^. miR-145-5p expression is consistently downregulated also in prostate cancer, and its ectopic expression in prostate cancer cells inhibits cancer cell migration and invasion by targeting GOLM-1 (Golgi membrane protein-1) mRNA^23^. GOLM-1 protein expression has been reported to be highly upregulated in cancer tissues and high GOLM-1 expression has been related to poor outcome. In breast cancer, miR-145-5p is strongly downregulated in cancer tissues compared to normal samples^24^. Interestingly, the expression of this miRNA has been found to inversely correlate with the expression of two proteins, JAM-A and FASCIN, in breast cancer cell lines^25^. Restoration of miR-145-5p expression in MDA-MB-231, MCF-7, MDA-MB-468 breast cancer cell lines resulted in strong reduction of cell motility and invasion, demonstrating the important role of this miRNA in suppressing tumor dissemination^25^. miR-145-5p also impacts on drug response, by post-transcriptional regulation of specific pharmacogenomic-related genes, as MRP1 (multidrug resistance (MDR)-associated protein 1, also known as ABCC1), the most important human ABC transporter involved in drug disposition and in multi-drug resistance (MDR)^26^. The contribution of miR-145-5p to tumor initiation and progression is however still debated as this miRNA has been recently described also as a non-cell autonomous oncogenic factor rather than a tumor suppressor^27^. We wondered whether Ago2 activity influenced the tumor suppressor/oncogenic function of miR-145-5p in breast cancer cells. We also explored if decreased Ago2 level favored the formation of alternative complexes with other Argonaute family members.

## Results

### miR-145-5p specifically upregulates Ago2 expression

As mentioned, downregulation of tumor suppressor miR-145-5p is a frequent event in human tumors, included breast cancer, and leads to the increased expression of its target mRNAs. For example, Thymic Epithelial Tumors show downregulated miR-145-5p and increased expression of its motility-related target genes Golm-1, EGFR and CDH2; moreover, in breast cancer miR-145 exogenous expression was shown to promote the inhibition of cell growth and the induction of apoptosis by targeting the Rho-effector rhotekin (RTKN)^18,21^. Also Ago2, crucial mediator of miRNAs activity, has been reported to be modulated in breast cancer^15^, despite contradictory results have been obtained regarding the kind of modulation (up- or down-regulated depending on the considered study) and the functional output of such modulation.

**Fig. 1:**
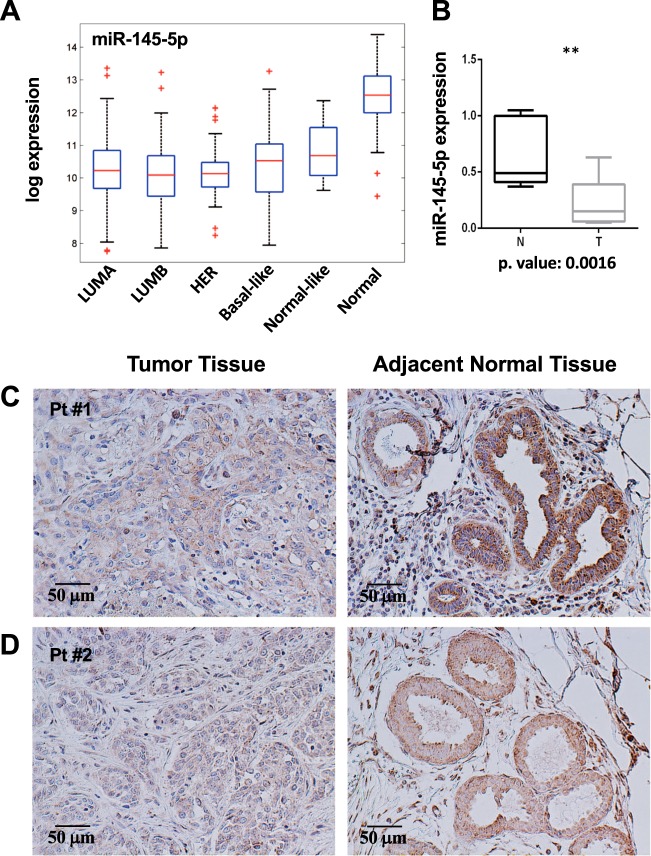
The loss of Ago2 expression correlates with miR-145-5p downregulation in breast cancer FFPE tumor samples. **a** Box plot showing the miR-145-5p expression in the various intrinsic subtypes of breast cancer tissues relative to normal tissues by using TGCA data; Clinical information of breast cancer patients and normalized TCGA miRNAseq data were obtained from http://gdac.broadinstitute.org/ (doi. 10.7908/C11G0KM9) and from Genomic Data Commons Data Portal (https://cancergenome.nih.gov/). Deregulation among different subgroups of samples was assessed by one way Anova test and two-sided *t*-test. **b** Box plot showing the modulation of miR-145-5p expression in 11 matched triple negative tumor tissues vs normal samples; **c**, **d** Immunohistochemistry for Ago2 in slides from 2 representative patients (Pt) with relative scale bars

Aiming to investigate the contribution of Ago2 to miR-145-5p function in breast cancer, we first evaluated their expression in BC tissues. We confirmed that miR-145-5p expression is reduced in breast cancer compared to normal tissues in the TGCA breast cancer dataset (Fig. 1a) and in a small group of matched FFPE (Formalin-Fixed, Paraffin-Embedded) primary triple-negative BC and normal adjacent tissues (Fig. 1b).

Using this small group of tumors and normal adjacent counterparts we then evaluated by IHC the expression of Ago2 protein. We observed that miR-145-5p downregulation was accompanied by a reduction also of Ago2 protein level in tumor tissues compared to the normal counterparts (Fig. 1c, d). In normal tissues, presenting organized tissue morphology, Ago2 mainly localized in the epithelial cells of glandular ducts (Fig. 1c, d).

**Fig. 2:**
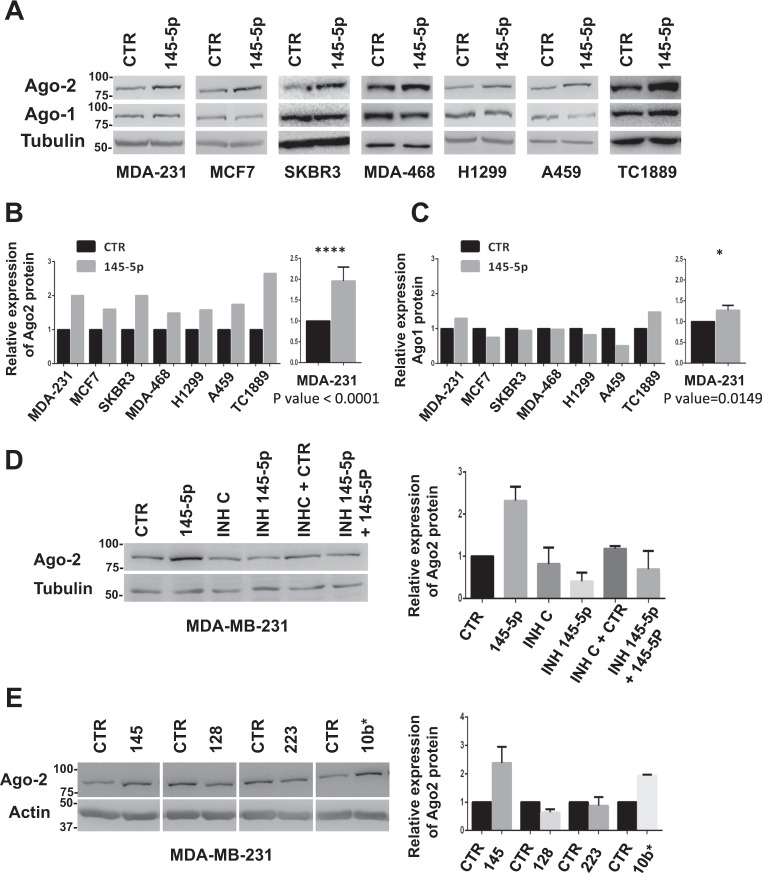
miR-145-5p regulates Ago2 expression. **a** Western blot analysis of Ago2 and Ago1 proteins from whole cell lysates of MDA-MB-231, MCF7, SKBR3, MDA-MB-468, H1299, A459 and TC1889, after 48 h of miR-145-5p ectopic expression; **b, c** Graphs representing Ago2 (**b**) and Ago1 (**c**) protein expression obtained by densitometry of Western blots shown in **a** and normalized over tubulin signals; analysis of MDA-MB-231 (right graphs) derives from three biological replicates. *P*-values were calculated by two-tailed *t*-test. **d** Western blot analysis of Ago2 protein from whole cell lysates of MDA-MB-231 transfected for 48 h with miR-145-5p or the relative control mimic, the inhibitor of miR-145-5p (INH-145) or negative control (INH-C) or transfected with both the control mimic and the negative inhibitor (CTR + INH-C) or both miR-145-5p and the inhibitor of miR-145-5p (INH-145); histogram (right) shows the densitometric analysis of Ago2 levels relative to Tubulin expression; data are presented as mean ± SEM from two biological replicates. **e** Western blot image (left) and densitometric analysis (right) of Ago2 protein after 48 h of miR-145-5p, miR-128, miR-223, miR-10b* overexpression; data are presented as mean  ± EM from two biological replicates

On the basis of the downregulation observed for both miR-145-5p and Ago2 in BC, we moved to breast cancer cell lines, to evaluate whether modulation of miR-145-5p levels had any effect on Ago2 expression. Ago2 protein levels have been indeed reported to depend on miRNAs overall expression^28,29^. Ectopic expression of miR-145-5p in a panel of breast cancer cell lines (MDA-MB-231, MCF7, SKBR3, MDA-MB-468) induced a consistent increase of Ago2 protein, compared to control miRNA (CTR) (Fig. 2a, b). Interestingly, a similar effect was also observed in additional non-breast carcinoma cell lines (Fig. 2a, b). Differently from what observed for Ago2, we did not observe a pervasive modulation of Ago1 protein in the analyzed cell lines (Fig. 2a–c).

Analysis of mRNA levels evidenced that miR-145-5p doesn’t affect Ago1 nor Ago2 mRNA expression (Supplementary Figure 1A–B), except in the MDA-MB-231 cell line where Ago2 transcript is induced (Supplementary Figure 1A). These results suggest that miR-145-5p could affect Ago2 expression mainly through post-transcriptional mechanisms.

In agreement, the transfection of miR-145-5p inhibitor oligonucleotide in Hs578T breast cancer cells, expressing high level of miR-145-5p (Supplementary Figure 1C), led to a decrease of Ago2 protein level compared to control inhibitor (Supplementary Figure 1D–E).

To further confirm the specificity of the effect observed on Ago2 protein levels, we evaluated the ability of miR-145-5p inhibitor oligonucleotide to counteract miR-145-5p-dependent Ago2 induction. As shown in Fig. 2d, Western blot and relative densitometry evidenced that miR-145-5p inhibitor oligonucleotide impaired the ability of miR-145-5p to induce Ago2 expression.

To evaluate if any miRNA caused Ago2 induction, we transfected MDA-MB-231 cells with a panel of mimic miRNAs (miR-145, miR-128, miR-223, and miR-10b*) and the relative controls. By Western blot we evidenced that only miR-145-5p and miR-10b* were able to upregulate of Ago2 expression (Fig. 2e), suggesting the existence of specific miRNA-dependent modulation of Ago2 expression.

### miR-145-5p enhances Ago2 mRNA translation

**Fig. 3:**
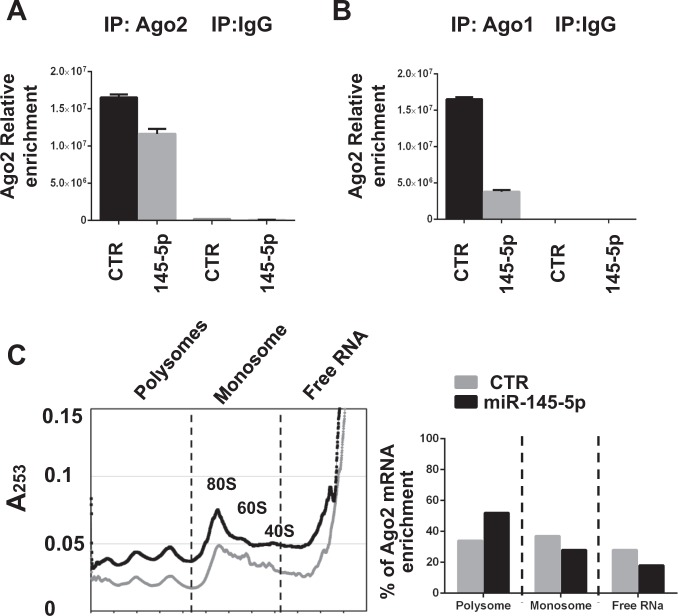
miR-145-5p enhances Ago2 mRNA translation. **a**, **b** RIP (RNA Immuno Precipitation) assays performed in control (CTR) and miR-145-5p overexpressing (145-5p) MDA-MB-231 cells using antibodies directed to Ago2, Ago1, or IgG as negative control. mRNA abundance of Ago2 was evaluated by RT-qPCR and normalized over GAPDH mRNA; a representative experiment of a biological duplicate is shown. **c** Cytoplasmic extracts from MD-MB-231 cells overexpressing miR-145-5p and the relative control cells were loaded on 15–50% sucrose gradients and fractions measured by absorbance at 253 nm (left panel). Fraction density decreases from left to right; a representative profile out of two independent biological replicates is showed. Right panel: pooled fractions of RNA associated with polysomes, monosomes and free RNA were analyzed by RT-qPCR. The percentage of Ago2 mRNA enrichment, normalized over GAPDH mRNA is reported

Ago2 mRNA itself has been shown to undergo miRNA-dependent translational repression^30,31^. To clarify the molecular mechanisms supporting the miR-145-5p-dependent Ago2 protein induction, we first evaluated the interaction of Ago2 mRNA with Ago1 and Ago2 proteins, important components of the post-transcriptional control machinery. Immunoprecipitations of Ago2 and Ago1 proteins followed by RT-qPCR for Ago2 mRNA (RIP assay) showed that Ago2 transcript interacts with both proteins in MDA-MB-231 cells (Fig. 3a, b). Interestingly, ectopic expression of miR-145-5p reduces the interaction of Ago2 mRNA with Ago2 and Ago1 proteins (Fig. 3a, b), suggesting a loosening of the miRNA-dependent negative control of Ago2 mRNA expression.

We next evaluated the abundance of Ago2 mRNA in cellular fractions of different ribosomal density. We observed that miR-145-5p transfection resulted in a shift of Ago2 mRNAs from low- to high-density ribosomal fractions, indicating increased translation (Fig. 3c). Altogether these results are in agreement with a release of the translational repression leading to Ago2 protein induction following miR-145-5p expression.

### miR-145-5p inhibits migration in Ago2-dependent manner

To investigate the functional output of miR-145-5p-Ago2 axis, we decided to evaluate its impact on cell migration, a function controlled by miR-145-5p in breast cancer cells^25,32^.

To this end, we first checked if miR-145-5p ectopic expression in MDA-MB-231 cells recapitulated the major functions previously reported for this miRNA in breast cancer cells. Specifically, we confirmed that miR-145-5p induced a significant downregulation of its targets Jam-A and Golm-1 (Supplementary Figure 2A–C), implicated in cell migration control. Accordingly, the migration ability of MDA-MB-231 cells overexpressing miR-145-5p was reduced compared to control (Supplementary Figure 2D). On the contrary, cell cycle progression was not affected by miR-145-5p expression (Supplementary Figure 2E) at least at the analyzed time point.

We then evaluated the impact of Ago2 modulation on MDA-MB-231 cell motility. Enforced expression of Ago2 has been reported to sustain cell motility in MCF-7 cells^33^. We observed that ectopic expression of Ago2 increased the migratory ability also of MDA-MB-231 cells (Supplementary Figure 3A), while Ago2 silencing consistently correlated with decreased migration capacity of cells (Supplementary Figure 3B).

**Fig. 4:**
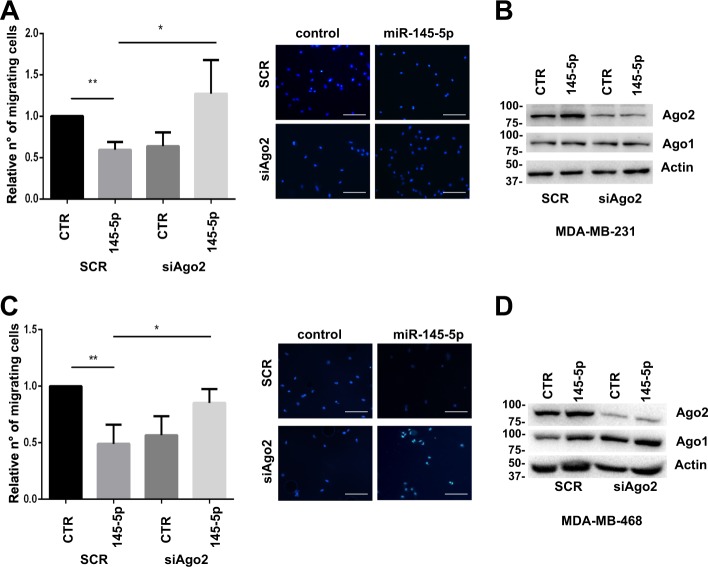
Ago2 expression is required for miR-145-5p-dependent inhibition of migration. **a**, **c** Migration assay and relative images from MDA-MB-231 **a** and MDA-MB-468 **c** cells transfected with control mimic or miR-145-5p in cells depleted or not of Ago2 protein expression (Scale bars, 100 μm). Data are presented as mean  ± standard deviation from three independent experiments; *P*-values were calculated by two-tailed *t*-test, *P < 0.05, **P < 0.005. **b**, **d** Western blot analysis to evaluate Ago2 and Ago1 protein expression in the same experimental conditions as in **a** and **c**

Finally, we investigated the impact of miR-145-5p overexpression on cell migration of cells depleted or not of Ago2 expression. As expected, miR-145-5p overexpression inhibited migration in control MDA-MB-231 and MDA-MB-468 cells (SCR) (Fig. 4a–d). Migration was reduced to a similar extent after Ago2 silencing (siAgo2) (Fig. 4a–d). Interestingly, miR-145-5p overexpression in Ago2-depleted cells did not induce further reduction of migration but, on the contrary, enabled recovery of migration ability (Fig. 4a–d).

These results indicate that miR-145-5p elicits its inhibitory effect on migration only in presence of Ago2 expression, while, in the absence of Ago2 protein, it exerts an opposite pro-migratory function.

### The absence of Ago2 enhances to the formation of miR-145-5p/Ago1 alternative active complex

**Fig. 5:**
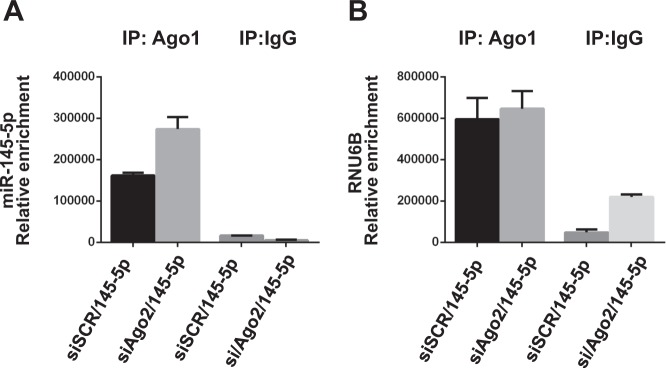
Depletion of Ago2 expression favors the formation of miR-145-5p/Ago1 complex. RIP assays performed in MDA-MB-231 cells transfected with miR-145-5p mimic, and depleted or not of Ago2 protein expression, using an antibody directed to Ago1 or IgG as negative control. Levels of interacting miR-145-5p (**a**) and RNU6B (**b**) were evaluated by RT-qPCR. A representative experiment of a biological duplicate is shown

To investigate if, in the absence of Ago2, a potentiation of the alternative miR-145-5p/Ago1 active complex is observed, we performed RNA immunoprecipitation (RIP) assays. To this end, MDA-MB-231 cells overexpressing miR-145-5p, depleted (siAgo2-145-5p) or not (scr-145-5p) of Ago2 expression, were used to perform immunoprecipitations with anti-Ago1 antibody or IgG as control. As shown in Fig. 5a, b, we observed increased miR-145-5p/Ago1 interaction in cells depleted of Ago2, compared to si-SCR cells, while no change for RNU6B/Ago1 interaction was observed. These results indicate that when Ago2 is depleted, miR-145-5p specifically increases its interaction with Ago1 protein. On this basis, we wondered if Ago1 protein might change its subcellular localization upon Ago2 depletion. As shown in Supplementary Figure 4, neither miR-145-5p ectopic expression nor Ago2 depletion altered the localization of Ago1 or Ago2 proteins, which are mostly cytoplasmatic. Based on these results, it is possible to speculate that miR-145-5p behaves as a tumor suppressor miRNA when it is complexed with Ago2 protein, while miR-145-5p activity may become oncogenic in the absence of Ago2, through the interaction with other RNA-binding proteins (RBPs), as for example Ago1.

### miR-145-5p exerts Ago2-dependent and -independent activities

To evaluate the impact of miR-145-5p on global gene expression, in presence or absence of Ago2 protein, we performed gene expression profiling in MDA-MB-231 cells, overexpressing or not miR-145-5p and depleted or not of Ago2 expression, as described in the two previous paragraphs. Microarray analysis revealed that miR-145-5p expression leads to the modulation of 1538 transcripts, of which 698 downregulated and 840 upregulated (Supplementary Figure 5 and Supplementary File 1). Among the downregulated mRNAs, potential direct targets of miR-145-5p, we found genes controlling cell proliferation (as for example CCNG1, CDK6, MAP3K1, MAP3K8, MAP3K4), cell motility (as PCDH1, GOLM1, JAM-A and various members of RAB family of proteins) and chemoresistance (as the five members of ATP-binding cassette transporters ABCA1, ABCA11P, ABCB10, ABCD4 and ABCE1). Thirty-five of the downregulated genes were previously identified and validated as direct targets of miR-145-5p as indicated by the miRTarBase data repository^34^ (Supplementary File 2).

**Fig. 6:**
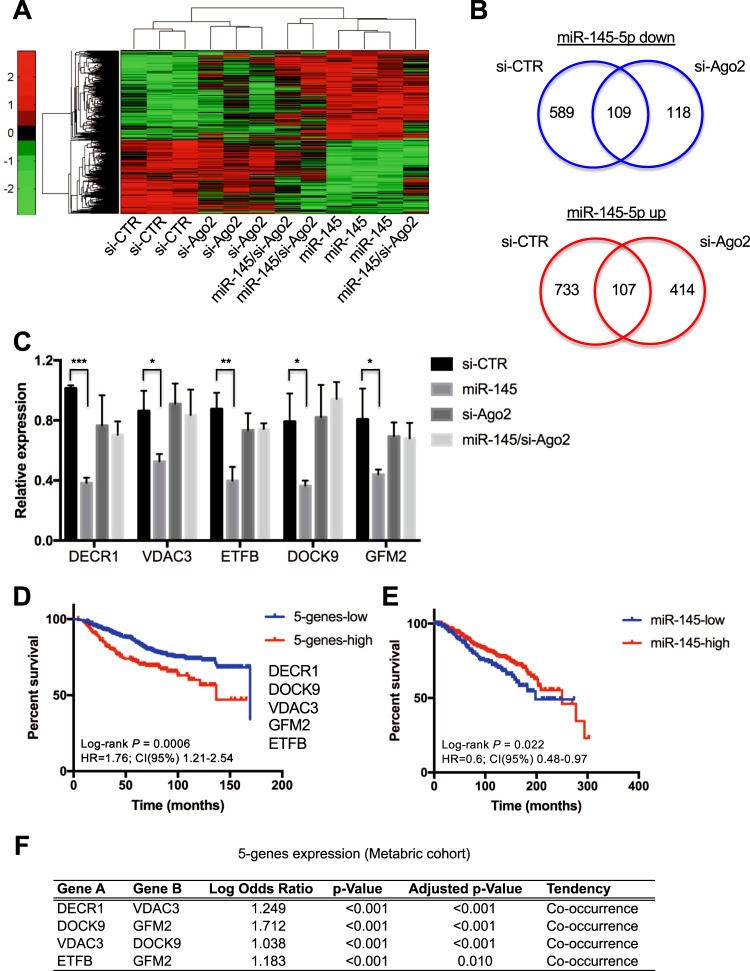
miR-145-5p exerts Ago2-dependent and -independent activities. **a** Expression matrix of transcripts whose expression is modulated by miR-145-5p in Ago2-dependent manner. **b** Diagrams showing the number of transcripts downregulated (upper panel) and up-regulated (lower panel) by miR-145-5p in presence (si-CTR) and in absence (si-Ago2) of Ago2 expression. **c** Validation by RT-qPCR of the expression of 5 top modulated miR-145-5p-dependent genes in the indicated experimental conditions, performed on three biological replicates. **d** Kaplan–Meier analysis of overall survival has been performed on a compendium of breast cancer microarray datasets deposited in the KMplot database, using a signature comprising the 5 transcripts most strongly modulated by miR-145-5p. **e** Kaplan–Meier analysis of overall survival has been performed on the Metabric cohort of breast cancer evaluating miR-145-5p expression levels. **f** Co-occurrence analysis of the overexpression of the 5 genes most strongly modulated by miR-145-5p in the Metabric breast cancer cohort performed using the cBioPortal cancer genomics database

Of note, we observed that, in the absence of Ago2 expression, miR-145-5p loses the ability to control 86% of its modulated transcripts, while retains 14% of its functions (Fig. 6a–b). Pathway and Gene Ontology analyses reporting the functions of the genes controlled by miR-145-5p, retained or not upon depletion of Ago2, is shown in Supplementary File 3.

Interestingly, we observed that miR-145-5p gains new functions in the absence of Ago2, acquiring the ability to modulate the expression of a different panel of genes (118 down- and 414 up-regulated transcripts), possibly through the formation of complexes with other RBP, as for example Ago1, at least for the downregulated targets (Fig. 6b). Pathway and Gene Ontology analyses revealed that different functions are exerted by miR-145-5p in absence of Ago2 compared to control cells (Supplementary File 3). Interestingly, different sets of cell adhesion molecules (CAMs) are modulated by miR-145-5p in presence or in absence of Ago2; this might reflect the opposite effect on cell motility observed upon miR-145-5p exogenous expression in these two conditions (as shown in Fig. 4a).

Based on our results, miR-145-5p is supposed to exert its tumor suppressor function only in presence of Ago2 expression; we then reasoned that the Ago2-dependent transcripts modulated by miR-145-5p in MDA-MB-231 cells could mediate tumor suppressor function and may represent predictive indicators in breast cancer. To test this, we first selected a few genes among the most significantly downregulated by miR-145-5p in presence of Ago2, we validated their modulation (Figure 6c) and we evaluated their predictive power using various breast cancer cohorts. Specifically, we observed that low expression of a signature comprising the top 5 downregulated genes (*DECR1*, *GFM2*, *DOCK9*, *VDAC3* and *ETFB*) (*P* < 0.0005) associates with increased overall survival in patients from a compendium of breast cancer studies (*N* = 626) included in the KMplot database (Fig. 6d)^35^. A similar result was observed evaluating the Metabric and the TCGA cohorts for overall and disease-free survival, respectively (Supplementary Figure 6). Opposite behavior was observed for miR-145-5p expression, whose high levels associate with higher survival probability (Fig. 6e); this result is specific for lymph node-negative patients, indicating that deregulation of miR-145-5p expression might be relevant for the initial stages of breast carcinogenesis.

Analysis of TCGA and Metabric cohorts enabled also to assess that various couples of these genes present co-occurring overexpression in breast cancer (Figure 6f and Supplementary Figure 6D).

Overall these results indicate that miR-145-5p exerts its tumor suppressor function in cells expressing Ago2 protein and suggest that Ago2 protein might contribute to the choice of targets by miR-145-5p.

## Discussion

Our study shows that both miR-145-5p and Ago2 protein are downregulated in breast cancer. Results about miR-145-5p are in line with report by Sempere and colleagues^36^, showing that expression of miR-145-5p is reduced or even completely abrogated in breast tumors.

Moreover, we show that miR-145-5p specifically induces post-transcriptionally Ago2 protein expression in both breast and non-breast carcinoma cell lines. This is obtained possibly through the loosening of Ago2 translational repression following miR-145-5p overexpression. Induction of Ago2 protein is necessary to allow miR-145-5p tumor suppressor function. In absence of Ago2 indeed miR-145-5p-dependent inhibition of migration is not observed while, on the contrary, we observed an enhancement of migration. At molecular level, upon Ago2 depletion an increase of miR-145-5p/Ago1 complex is observed. This suggests that when Ago2 is downregulated miR-145-5p might be involved in interactions with additional RNA-binding proteins (RBPs), as Ago1 for example, and acquire different functions. This is also reflected by the microarray analysis in cells overexpressing miR-145-5p. We indeed observed that most of miR-145-5p functions are lost upon Ago2 depletion, while additional functions are gained. miR-145-5p indeed leads to the modulation of a huge number of genes in the absence of Ago2, genes whose modulation is not observed in the presence of Ago2 expression. We can hypothesize that these genes may depend on the activity of miR-145-5p complexed with other members of Ago family of proteins or additional RNA-binding proteins. Further studies will enable clarifying the functional output of the modulation of this Ago2-independent set of genes. However, as restoration of tumor suppressor miRNA levels has been proposed recently as a promising therapeutic intervention to cure diverse cancers, our study underlines how information about Ago2 protein expression level becomes crucial for the success of these novel therapeutic approaches. Moreover, Ago2 protein activity and stability are controlled by post-translational modifications, as for example phosphorylation at Tyr 393;^37^ possibly, these Ago2 post-translational modifications might contribute to the definition of miRNA-specific target recognition and, consequently, determine the biological output of tumor suppressor and oncogenic miRNAs.

## Conclusions

In summary, our results show that miR-145-5p dependent tumor suppressor function is exerted only in presence of Ago2 protein expression. Our findings suggest that Ago2 downregulation in breast cancer might strongly influence the activity of miRNAs and highlight the importance of evaluating Ago proteins levels when studying miRNAs activity in cancer.

## Materials and methods

### Cell culture and transfection

MDA-MB-231, SKBR3, MDA-MB-468, H1299, A459 cell lines were cultured in DMEM medium supplemented with 50 U/mL penicillin, 50 U/ml streptomycin and 10% heat-inactivated FBS at 37 °C in an incubator with humidified 5% CO_2_ atmosphere. Human Thymic Carcinoma cell line TC1889 was kindle provided by Dott. M Breining^38^ and cultured in RPMI 1640 containing 4.5 g/L glucose, 25 mM Hepes, 50 U/mL penicillin, 50 U/ml streptomycin and 10% heat-inactivated FBS at 37 °C in an incubator with humidified 5% CO2 atmosphere. Hs578T was cultured in DMEM with 0.01 mg/mL human insulin with a fetal bovine serum to a final concentration of 10%. MCF10A was cultured in F12 medium supplemented with 5% horse serum, 10 ug/mL Insulin, 0.5 ug/mL Hydrocortisone and 20 ng/mL EGF.

Pre-miR-145-5p (PM11480 #AM17100, Ambion), Pre-miR-223 (PM12672 #AM17100, Ambion), Pre-miR-10b* (PM12387, #AM17100 Ambion), Pre-miR-128 (PM24586 #AM17100, Ambion) and Pre-miRNA Negative Control (#AM17110, Ambion) were transfected at final concentration of 5 nM using Lipofectamine RNAiMAX (Invitrogen) according to the manufacturer’s instructions.

For gene silencing experiments, MDA-MB-231 were transfected with Dicer-substrate short interfering RNAs (DsiRNAs) for AGO2 (IDT, Belgium) and NC1 negative control (IDT, Belgium) at 500 pM for 72 h.

For the inhibition of miRNA expression, MDA-MB-231 cells were transfected with hsa-miR-145-5p mirVana miRNA inhibitor (#MH11480, Ambion) and negative control #1 (#4464077, Ambion) at final concentration of 5 nM using Lipofectamine RNAiMAX (Invitrogen) according to the manufacturer’s instructions.

For Ago2-HA exogenous expression, MDA-MB-231 cell line was transfected with Ago2-HA vector provided by Prof. Filipowicz^39^ by using Lipofectamine 2000 (Invitrogen), according to the manufacturer’s instructions.

### Total RNA extraction from cells and reverse transcription

Total RNA was extracted using the TRIZOL Reagent (GIBCO). 500 ng of total RNA were reverse-transcribed using High-Capacity RNA-to-cDNA Kit (Applied Biosystems) and diluted 1:5 for PCR reactions. PCR analyses were carried out by using oligonucleotides specific for the genes listed in Supplementary Table 1 and gene expression was measured by real-time PCR using the Sybr Green assay (Applied Biosystems, Carlsbad, CA, USA) on Abi Prism 7500 (Applied Biosystems). All reactions were performed in duplicate.

RNA from FFPE samples was extracted using the miRneasy FFPE kit (Qiagen, Chatsworth, CA) following the manufacturer’s instructions. 50 ng of total RNA was reverse transcribed in 20 μl by using miScript II RT kit (Qiagen, Chatsworth, CA) and 1 μl of cDNA dilution (1:5) was used for quantitative real-time PCR (RT-qPCR) experiments. Quantification of miR-145-5p was carried out by miScript Primer Assay (Hs_miR-145_1 miScript Primer Assay MS00003528 Qiagen, Chatsworth, CA), normalizing over RNU6B control (Hs_RNU6-2_11 miScript Primer Assay MS00033740 Qiagen, Chatsworth, CA) and using the miScript SYBR Green PCR kit (Qiagen, Chatsworth, CA) on Abi Prism 7500 (Applied Biosystems). All reactions were performed in duplicate.

### Lysate preparation and immunoblotting analysis

Cells were lysed in 2% SDS buffer (25 mM Tris-Hcl pH 7.5, 100 mM Nacl, 3 mM EDTA, 7% Glycerol) and fresh protease inhibitors. Extracts were sonicated for 10 s and centrifuged at 12,000 × rpm for 10 min to remove cell debris. Western blotting was performed using the following primary antibodies: mouse monoclonal anti-Gapdh 6C5 (#sc32233, Santa Cruz Biotechnology); mouse monoclonal anti-Tubulin B512 (#T5168, Sigma-Aldrich, Milan, Italy); mouse monoclonal anti-b-Actin (ACTBD11B7) (Santa Cruz Biotechnology sc-81178), rabbit polyclonal anti-Golm1 (#PA5-30622, Thermo Scientific); rabbit polyclonal antibodies: anti-Ago2 (#2897, Cell Signaling); rabbit monoclonal antibodies anti-Ago1 (D84G10) XP (#5053, Cell Signaling); rabbit monoclonal antibodies anti HA-Tag (#3724, Cell Signaling).

As secondary antibodies goat anti-mouse and anti-rabbit conjugated to horseradish peroxidase were used (Bethyl Laboratories). Western blot analysis was performed with the aid of the enhanced chemiluminescence system (Thermo Fisher Scientific, Rockford, IL, USA). ECL detection was done using a ChemiDoc-It Imaging System (UVP, Upland, CA) instrument.

### Nuclear and cytoplasmic extracts preparation

Cells were lysed in hypotonic buffer (50 mM HEPES; 150 mM NaCl; 2 mM EDTA; 10 mM MgCl_2_, 10 mM KCL; 0.5% Nonidet P-40; fresh DTT 2.5 mM and fresh protease inhibitors) for 15 min on ice followed by 5 min of centrifugation at 1200 rpm. The supernatants were removed and used as cytosolic fraction and the pellet (corresponding to the nuclei) was resuspended in 2% SDS buffer (25 mM Tris-Hcl pH 7.5, 100 mM NaCl, 3 mM EDTA, 7% Glycerol) and fresh protease inhibitors. Extracts were sonicated for 10 s and centrifuged at 12,000 × rpm for 10 min to remove cell debris and to obtain nuclear proteins. Mouse anti-Gapdh 6C5 (#sc32233, Santa Cruz Biotechnology) and rabbit polyclonal anti Histone H3 (#Abcam 1791) were used as markers of the cytoplasmic and nuclear fraction, respectively.

### Cell cycle analysis

For cell cycle analysis, 2 × 10^5^ cells were resuspended in 50% FCS, fixed in 70% ethanol for 24 h, incubated with 50 μg/ml propidium iodide (Sigma-Aldrich) and 50 units/ml Dnase-free RNase A (Sigma-Aldrich) and analyzed after 3 h (1 × 10^4^ events) using an Epics XL Cytometer (Beckman Coulter).

### Transwell migration assay

Migration assay was performed by using a 24-well plate with a non-coated 8-mm pore size filter in the insert chamber (BD Falcon, Franklin Lakes, NJ, USA). MDA-MB-231 or MDA-MB-468 cell lines were transfected with pre-miR-145-5p and the pre-miRNA Precursor-Negative Control (Ambion) for 48 h or silenced with siAgo2 or NC1 negative control (IDT, Belgium) at 500 pMoles or transfected with pcDNA3-Ago2-HA or pcDNA3-HA vectors for 72 h. After transfection, 5 × 10^4^ cells were resuspended in serum-free DMEM and seeded into the insert chamber. Cells were allowed to migrate for 24 h into the bottom chamber containing 0,7 ml of DMEM supplemented with EGF (30 ng/ml) in a humidified incubator at 37 °C in 5% CO_2_. Cell migration was evaluated by discarding cells which did not migrate (upper side) and by counting the number of cells on the underside of the membrane using DAPI staining.

### Immunohistochemistry

Ago2 protein expression was analyzed by immunohistochemistry (IHC) in a set of 11 triple negative FFPE breast tumors and 11 normal counterparts from patients who underwent surgery from 2014 to 2015 (Sapienza University of Rome, Pathology Unit, ICOT, Latina, Italy) and summarized in the Supplementary Table 2. None of the patients had received any radio or radio-chemotherapy before surgery. Formalin-fixed, paraffin-embedded 5μm sections were Haematoxylin and Eosin stained to select well-represented epithelial cell compartment. Serial/subsequent sections were stained with the anti-Ago2 antibody (#ab57113, Abcam) at 1:100 dilution. Protein staining was revealed through DAB enzymatic reaction while nuclei were counterstained with hematoxylin.

### Polysome profiling

After 48 h of transfection with Pre-miR-145-5p (PM11480 #AM17100, Ambion) and Pre-miRNA Precursor Negative Control (#AM17110, Ambion), 20 × 10^6^ cells were lysed with 400 μl of lysis buffer (10 mM Tris pH 7.5, 10 mM NaCl, 10 mM MgCl_2_, 1% Triton X-100) supplemented with 10 mM fresh DTT, 100 μg/ml cycloheximide, 1X PIC (Complete, EDTA free, Roche) and 1X RNase guard (Thermo Scientific).

Cells were allowed to swell for 10 min on ice. The lysates were centrifuged for 10 min at 13,000 rpm at 4 °C. The supernatants were collected and centrifuged on 15–50% sucrose gradient at 38,000 rpm with a SW41 rotor (Beckman) for 1 h 30 min at 4 °C. Fractions were collected with a Bio-logic LP (Biorad). Total RNA derived from 300 μl of fraction was extracted using 700 μl of TRIZOL Reagents (GIBCO).

### RNA-binding protein immunoprecipitation (RIP) assay

Lysates from 20 × 10^6^ cells were immunoprecipitated using the Magna RIP kit (#17-700, Millipore) following the manufacturer’s instructions with an anti-Ago2 antibody (Millipore, # 03-110) and anti-Ago1 antibody (Millipore #03-249) or control IgG.

Binding of miRNAs and Ago2 mRNA respectively to Ago1 or Ago2 was evaluated by RT-qPCR. The quantification of miR-145-5p or RNU6B or Ago2 mRNA was carried out on Abi Prism 7500 (Applied Biosystems) using the miScript SYBR Green PCR kit (Qiagen, Chatsworth, CA) and all the reactions were performed in duplicate.

### Microarray analysis

Expression profiling by microarray has been performed on the Agilent platform using Low Input Quick Amp WT Labeling Kit, one-color, on SurePrint G3 Human Exon 2 × 400 K slides. Gene expression profiling from Agilent arrays was extracted by Agilent Feature Extraction software. Values lower than 1 were considered below detection and thresholded to 1. Signals were quantile normalized and log2-transformed. Gene modulation was assessed using a permutation test and a paired T-test. A false discovery procedure was also included for multiple comparisons. Unsupervised hierarchical clustering was performed by using the Euclidean distance metric.

Bioinformatic analyses were performed by MATLAB (The MathWorks). Pathway and Gene Ontology analyses were performed using the DAVID functional annotation tool (https://david.ncifcrf.gov). Microarray data are available as GSE120896 in GEO Database (https://www.ncbi.nlm.nih.gov/geo).

### Survival analyses

Survival analyses were performed on the KMplot database^35^ using as input the 6-genes signature of miR-145-5p top downregulated genes on the whole breast cancer dataset (*N* = 626). Logrank *P* and HR were calculated using GraphPad Prism 7 software. Survival analyses on the Metabric and TCGA cohorts were performed using the cBioPortal Database (http://www.cbioportal.org) using as input the mRNA expression levels of the 6-genes signature.

### Statistical analyses

The statistical analyses were performed using GraphPad Prism 7 (GraphPad Software, San Diego California USA). Values of *P* ≤ 0.05 (*) and *P* ≤ 0.01 (**) were considered statistically significant.

### List of abbreviations

Ago: Argonaute; BC: breast cancer; miRNAs: microRNA; RISC: RNA-induced silencing complex; UTR: untranslated region; EGFR: epidermal growth factor receptor; NUDT1: nudix hydrolase 1; GOLM-1: Golgi membrane protein-1; MDR: multi-drug resistance.

## Supplementary information

Supplementary Figure 1

Supplementary Figure 2

Supplementary Figure 3

Supplementary Figure 4

Supplementary Figure 5

Supplementary Figure 6

Supplementary File 1

Supplementary File 2

Supplementary File 3

Supplementary Table 1

Supplementary Table 2
