# Supplementary material for: Argonaute 2 drives miR-145-5p-dependent gene expression program in breast cancer cells
Source: Cell Death Dis. 2019 Jan 8;10(1):17. doi: 10.1038/s41419-018-1267-5 (PMC6325137; doi:10.1038/s41419-018-1267-5)
Supplement: Supplementary file 1 — Supplementary Figure 1 [file 41419_2018_1267_MOESM1_ESM.pdf]

Bellissimo et al. Supplementary Figure 1

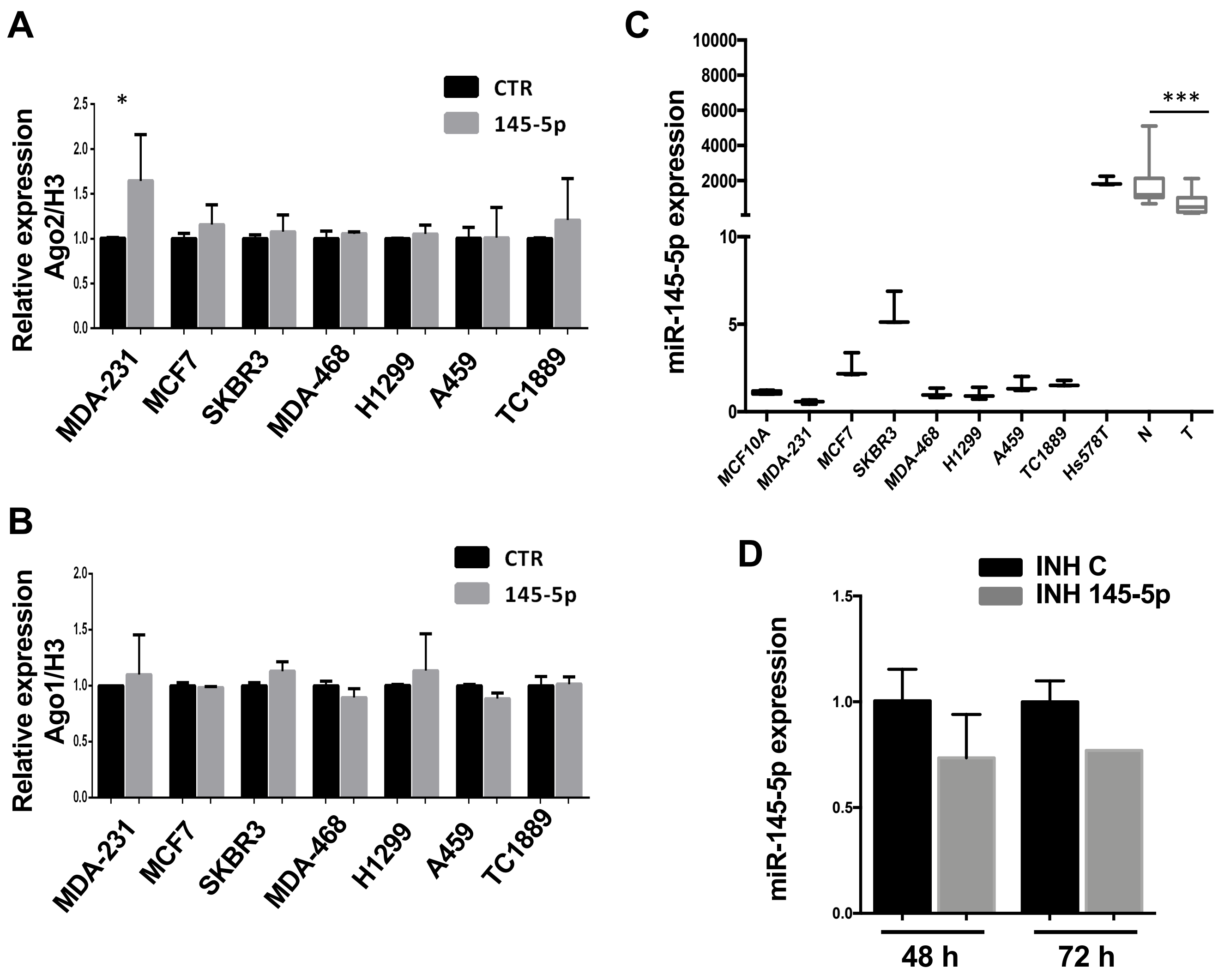

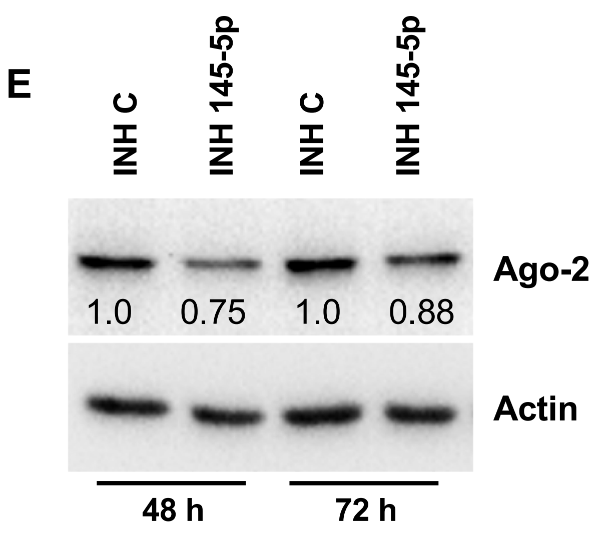

Supplementary Figure 1: A-B) RT-qPCR analysis to evaluate Ago2 **(A)** and Ago1 **(B)** expression in MDA-MB-231, MCF7, SKBR3, MDA-MB-468, H1299, A459 and TC1889 cells upon 48h of miR-145-5p transfection; **C)** Box plot showing the miR-145-5p expression evaluated by RT-qPCR analysis in the indicated cell lines and normal (N) and tumoral (T) breast tissues; **D)** RT-qPCR in Hs578T cell line transfected for 48h or 72h with the inhibitor of miR-145-5p (INH-145-5p) or negative control (INH-C) to evaluate miR-145-5p expression; **E)** Western blot analysis of Ago2 protein from whole cell lysates of Hs578T transfected for 48h or 72h with the inhibitor of miR-145-5p (INH-145-5p) or negative control (INH-C). Numbers indicate the relative quantification normalized on Actin levels.
