# Supplementary material for: Argonaute 2 drives miR-145-5p-dependent gene expression program in breast cancer cells
Source: Cell Death Dis. 2019 Jan 8;10(1):17. doi: 10.1038/s41419-018-1267-5 (PMC6325137; doi:10.1038/s41419-018-1267-5)
Supplement: Supplementary file 2 — Supplementary Figure 2 [file 41419_2018_1267_MOESM2_ESM.pdf]

Bellissimo et al. Supplementary Figure 2

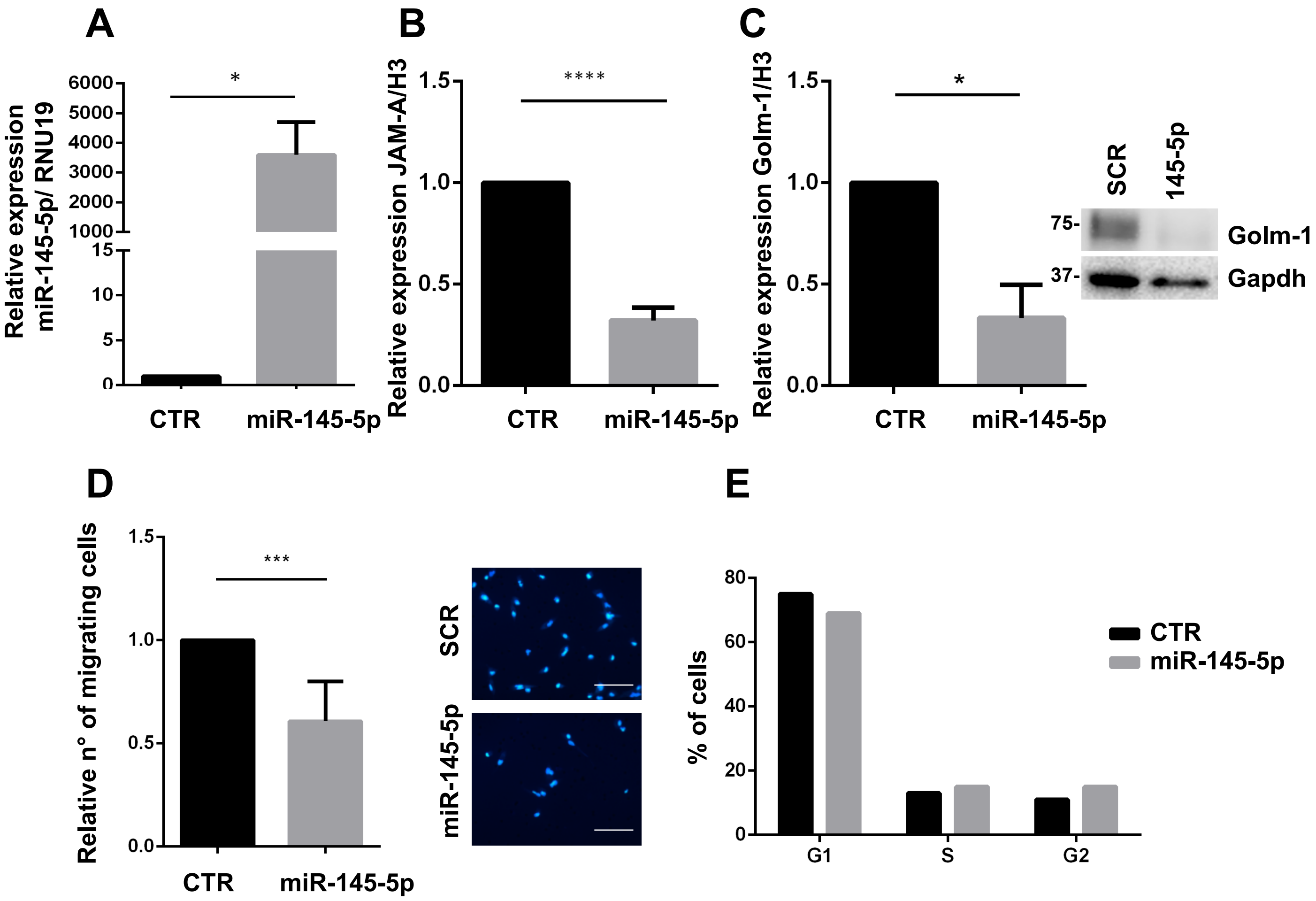

Supplementary Figure 2: miR-145-5p expression impairs cell migration of MDA-MB-231 cells and inhibits Golm-1 protein: RT-qPCR analysis to evaluate miR-145-5p **(A)**, JAM-A **(B)** and Golm-1 **(C)** expression upon 48h of miR-145-5p transfection; **(C)** Right panel shows western blotting to examine Golm-1 protein after 48h of miR-145-5p overexpression; **(D)** Transwell migration assay showing a reduction of migratory properties upon 48h of miR-145-5p overexpression (Scale bars, 100 μm) and **(E)** FACS analysis of cell cycle performed following 48h of miR-145-5p ectopic expression.
