# Supplementary material for: Argonaute 2 drives miR-145-5p-dependent gene expression program in breast cancer cells
Source: Cell Death Dis. 2019 Jan 8;10(1):17. doi: 10.1038/s41419-018-1267-5 (PMC6325137; doi:10.1038/s41419-018-1267-5)
Supplement: Supplementary file 3 — Supplementary Figure 3 [file 41419_2018_1267_MOESM3_ESM.pdf]

Bellissimo et al. Supplementary Figure 3

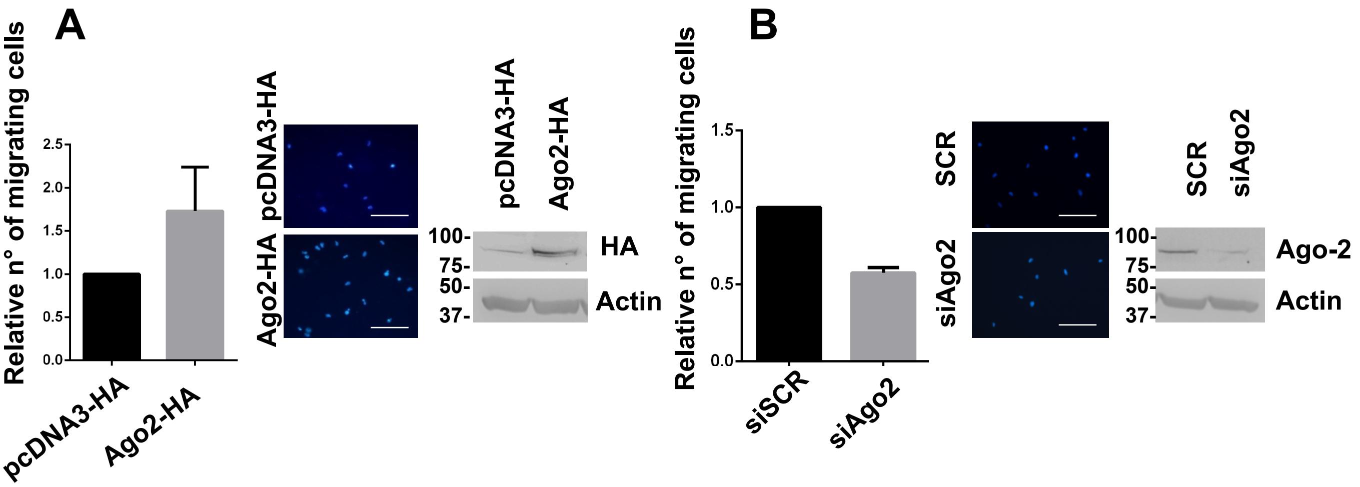

Supplementary Figure 3: miR-145-5p overexpression inhibits migration with the contribution of Ago2: (A) Transwell migration assay showing an increase of MDA-MB-231 migratory properties upon 72h of Ago2-HA overexpression and relative images (left panel) (Scale bars, 100 μm); western blot analysis of Ago2-HA in the same condition (right panel); (B) Transwell assay showing a reduction of migratory properties after Ago2 silencing with relative images (left panel) (Scale bars, 100 μm); western blot analysisis performed upon 72h of Ago2 RNA interference (right panel);
