# Supplementary material for: Argonaute 2 drives miR-145-5p-dependent gene expression program in breast cancer cells
Source: Cell Death Dis. 2019 Jan 8;10(1):17. doi: 10.1038/s41419-018-1267-5 (PMC6325137; doi:10.1038/s41419-018-1267-5)
Supplement: Supplementary file 4 — Supplementary Figure 4 [file 41419_2018_1267_MOESM4_ESM.pdf]

Bellissimo et al. Supplementary Figure 4

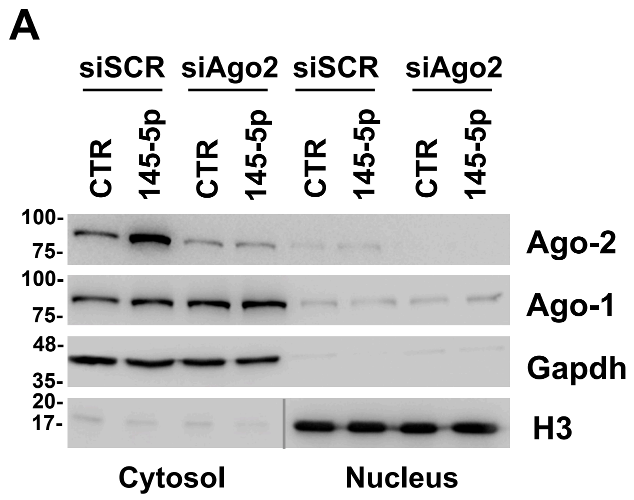

Supplementary Figure 4: Western blot analysis to determine Ago2 and Ago1 localization in MDA-MB-231 cells transfected for 48h with SCR-control mimic, SCR-miR-145-5p, in sample silenced for Ago2 (siAgo2) and in sample overexpressing miR-145-5p and silenced for Ago2 (siAgo2-145-5p). Equal amounts (40µg) of cytoplasmic fractions and nuclear fractions from each sample were analyzed. Gapdh and Histone H3 were used as markers of the cytoplasmic and nuclear fraction, respectively.
