# Supplementary material for: Argonaute 2 drives miR-145-5p-dependent gene expression program in breast cancer cells
Source: Cell Death Dis. 2019 Jan 8;10(1):17. doi: 10.1038/s41419-018-1267-5 (PMC6325137; doi:10.1038/s41419-018-1267-5)
Supplement: Supplementary file 5 — Supplementary Figure 5 [file 41419_2018_1267_MOESM5_ESM.pdf]

Bellissimo et al. Supplementary Figure 5

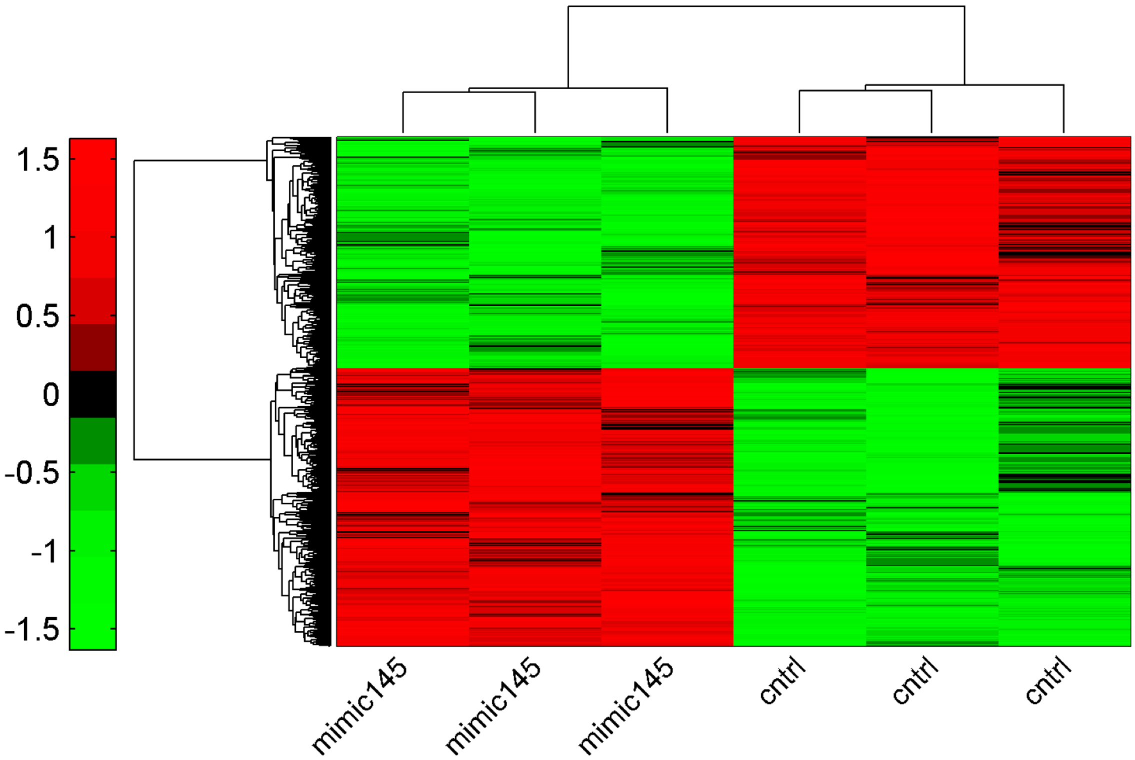

Supplementary Figure 5. Expression matrix of genes modulated by miR-145-5p expression in MDA-MB-231 cells.
