# Supplementary material for: Argonaute 2 drives miR-145-5p-dependent gene expression program in breast cancer cells
Source: Cell Death Dis. 2019 Jan 8;10(1):17. doi: 10.1038/s41419-018-1267-5 (PMC6325137; doi:10.1038/s41419-018-1267-5)
Supplement: Supplementary file 6 — Supplementary Figure 6 [file 41419_2018_1267_MOESM6_ESM.pdf]

Bellissimo et al. Supplementary Figure 6

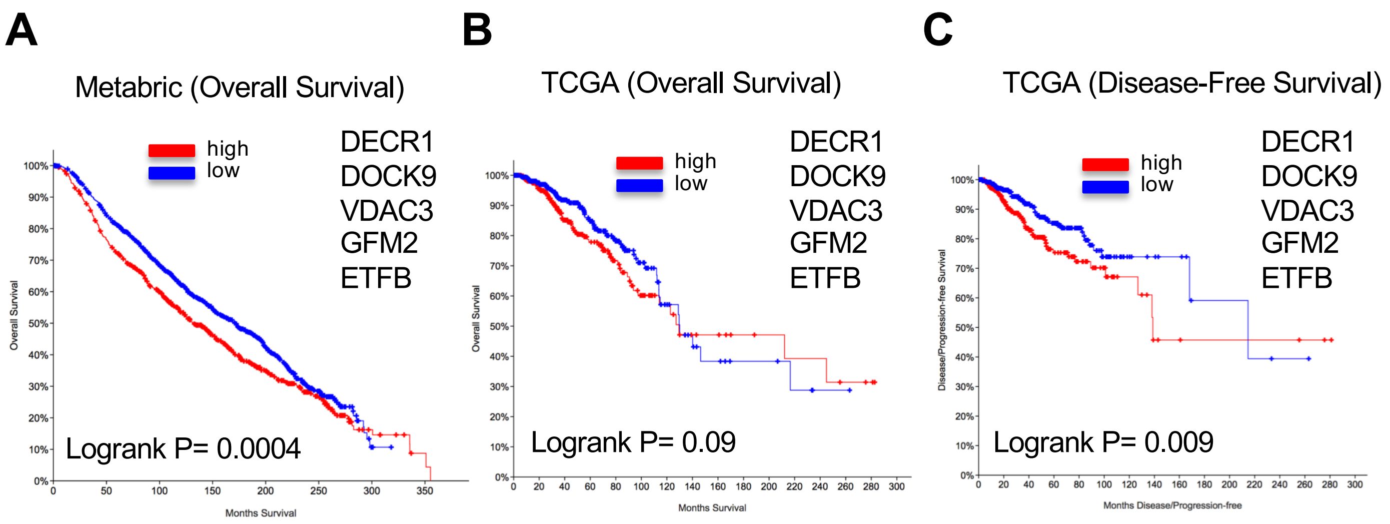

D

5-genes expression (TCGA cohort, RNA-seq data)

Gene A	Gene B	Log Odds Ratio	p-Value	Adjusted p-Value	Tendency
DECR1	VDAC3	1.280	<0.001	<0.001	Co-occurrence
DOCK9	ETFB	1.842	<0.001	0.001	Co-occurrence
DECR1	ETFB	1.119	0.002	0.019	Co-occurrence

Supplementary Figure 6. (A-C) Kaplan Meier analysis to evaluate the predictive power of a signature comprising the 5 top downregulated genes of miR-145-5p (DECR1, GFM2, DOCK9, VDAC3, ETFB) has been performed on the Metabric (A) and the TCGA (B-C) datasets using cBioPortal database. (D) Co-occurrence analysis has been performed considering the overexpression of the 5 top miR-145-5p-dependent downregulated genes on the TCGA dataset using the cBioPortal cancer genomics database.
