# Supplementary material for: Argonaute 2 drives miR-145-5p-dependent gene expression program in breast cancer cells
Source: Cell Death Dis. 2019 Jan 8;10(1):17. doi: 10.1038/s41419-018-1267-5 (PMC6325137; doi:10.1038/s41419-018-1267-5)
Supplement: Supplementary file 7 — Supplementary File 1 [file 41419_2018_1267_MOESM7_ESM.pdf]

entrezID	permutation p	ttest p	FDR	log2fold	mean mimic145	mean cntrl	pval(4Vs3)	log2fold(4Vs3)	pval(3Vs1)	log2fold(3Vs1)	gene	name	locus group	locus type	ensembleID	
64853	1,80966E-14	4,8739E-07	0,006036748	1,0690809	9,744469551	8,675388659	0,008328	0,527222808	0,013484073	0,669452166	AIDA	, dorsalizat	ein-coding	th protein	G00000186063	
92856	5,42819E-06	2,0216E-06	0,012519664	0,6536883	10,87239204	10,21870372	0,413675	-0,09494756	0,019960798	0,355294221	IMP4	nall nucleo	ein-coding	th protein	G00000136718	***mimic145\cntrl***
344558	1,08498E-05	7,9431E-06	0,032794484	0,851205	7,45589905	6,604694058	0,172002	0,213729243	0,206225881	0,167405197	SH3RF3	containing	ein-coding	th protein	G00000172985	
54799	3,87828E-05	1,2347E-05	0,030585766	-0,546362	6,516832109	7,063194001	0,116897	-0,254547105	0,023164232	-0,067187855	MBTD1	main cont	ein-coding	th protein	G00000011258	
11160	6,50576E-05	1,4937E-05	0,030835572	1,106274	8,091065895	6,984791849	0,001276	0,656956972	0,011967376	-0,349357302	ERLIN2	raft assoc	ein-coding	th protein	G00000147475	
122970	6,91305E-05	1,6435E-05	0,029080356	-0,890693	7,848690182	8,73938324	0,35677	-0,207046271	0,01439078	-0,646203723	ACOT4	oA thioeste	ein-coding	th protein	G00000177465	
867	0,000113245	3,081E-05	0,047702048	0,5510886	8,33135129	7,780262652	0,014035	0,286007934	0,001856589	0,3667506	CBL	roto-onco	ein-coding	th protein	G00000110395	
1009	0,000118274	3,1221E-05	0,042966673	1,0745267	9,379078383	8,304551715	0,008513	0,516846657	0,061294095	-0,208969776	CDH11	cadherin 1	ein-coding	th protein	G00000140937	
90411	0,00010648	3,2344E-05	0,040060929	0,9660915	12,65783058	11,69173906	0,003747	0,547741598	0,049752616	0,230262658	MCFD2	ulation fact	ein-coding	th protein	G00000180398	
677781	0,000170735	4,1414E-05	0,04663175	-1,602544	8,392339716	9,994883481	0,091984	-1,304765538	0,044763999	-0,356715131	SCARNA16	body-spec	n-coding R	small nucl	G00000275143	
146691	9,66341E-05	4,4828E-05	0,046270054	0,5901022	7,41568783	6,825585673	0,013894	0,356946771	0,086378133	0,18127576	TOM1L2	membran	ein-coding	th protein	G00000175662	
1666	0,00017749	4,5959E-05	0,043788298	-1,051175	10,0491848	11,10035965	0,342762	-0,117292224	0,169256731	-0,192343415	DECR1	reductase	ein-coding	th protein	G00000104325	
200014	0,000187068	7,0192E-05	0,06209937	-0,803791	6,584800443	7,388591756	0,000484	-0,567035935	0,088038254	-0,113021392	CC2D1B	C2 domair	ein-coding	th protein	G00000154222	
4490	0,00027885	7,2396E-05	0,059779368	-0,860305	13,19954546	14,05985026	0,054713	-0,502111239	0,118173373	-0,140603217	MT1B	allothionei	ein-coding	th protein	G00000169688	
9891	0,00019928	7,2856E-05	0,056399842	-1,437908	6,475776887	7,913684638	0,009987	-0,899260588	0,053488265	-0,329976092	NUAK1	family kin	ein-coding	th protein	G00000074590	
84340	0,000182787	7,9203E-05	0,057706332	-0,70347	7,033423493	7,736893866	0,066762	-0,234845231	0,129354577	-0,17839369	GFM2	factor mit	ein-coding	th protein	G00000164347	
401236	0,000228874	8,1822E-05	0,056302451	1,5767957	3,775102832	2,198307086	0,623548	-0,298032729	0,013493069	1,299852443	STMND1	domain co	ein-coding	th protein	G00000230873	
4697	0,000320095	9,6507E-05	0,062911993	-1,237635	12,6425898	13,88022434	0,003033	-0,865238309	0,054779395	-0,221521128	NDUFA4	ondrial cor	ein-coding	th protein	G00000189043	
4500	0,00035398	0,00011363	0,07037058	-0,809473	12,68050011	13,48997327	0,083062	-0,670008293	0,087691415	-0,302673859	MT1L	in 1L (gene	ein-coding	th protein	G00000260549	
728568	0,00014943	0,00011948	0,067267801	1,0806168	8,758899013	7,678282173	0,001988	0,836105395	0,207225969	0,143986778	C12orf73	2 open re	ein-coding	th protein	G00000204954	
112950	0,000383083	0,00013795	0,074290067	0,6352079	10,65141809	10,0162102	0,003119	0,261432914	0,010453421	0,179334887	MED8	r complex	ein-coding	th protein	G00000159479	
79102	0,000122731	0,00018066	0,089506277	0,624286	13,23179547	12,60750946	0,130032	0,215619484	0,279622575	0,133623835	RNF26	inger prote	ein-coding	th protein	G00000173456	
55604	0,000336934	0,00020182	0,092584545	-0,857946	7,291129226	8,149074799	0,037819	-0,435617487	0,000684637	-0,778703416	CARMIL1	gulator an	ein-coding	th protein	G00000079691	
6322	0,000219327	0,00020668	0,088272448	1,8683433	9,692422078	7,824078786	0,004009	0,929552913	0,081514525	0,337257666	SCML1	nidleg-like	ein-coding	th protein	G00000047634	
11282	4,2861E-05	0,00021189	0,087480563	-1,319132	9,754964283	11,07409617	0,026398	-0,690483514	0,009831913	-0,302069101	MGAT4B	a-1,4-N-ac	ein-coding	th protein	G00000161013	
56925	0,000284311	0,00021428	0,085613882	-1,856245	7,04142623	8,897670828	0,004829	-0,486801451	0,005678941	-0,445257597	LXN	latexin	ein-coding	th protein	G00000079257	
9353	0,000388886	0,00021436	0,082971861	1,0402391	7,038838529	5,998599385	0,002749	0,605526584	0,687731391	0,036936113	SLIT2	uidance lig	ein-coding	th protein	G00000145147	
23348	0,000145369	0,00025259	0,089388272	-0,928181	8,212417404	9,140598041	0,47156	-0,132228974	0,433558265	0,093649038	DOCK9	or of cytok	ein-coding	th protein	G00000088387	
7419	0,000406095	0,00027545	0,092208565	-0,544813	9,464883801	10,00969657	0,391309	-0,098452075	0,609383145	-0,046956507	VDAC3	endent ani	ein-coding	th protein	G00000078668	
5704	0,000459302	0,00030812	0,088754028	0,5363499	10,74757497	10,21122503	0,012908	0,163850138	0,015642404	0,174327821	PSMC4	26S subur	ein-coding	th protein	G00000013275	
22845	0,000367613	0,00030949	0,087121213	0,7327117	11,17956815	10,44685649	0,199537	0,164313928	0,017814951	0,36183591	DOLK	lichol kina	ein-coding	th protein	G00000175283	
340581	0,000610354	0,00031874	0,087731634	-2,162875	3,590916213	5,753791577	0,638283	-0,365571254	0,131929283	-1,501374634						
9526	0,000411024	0,00032186	0,086663851	-0,686286	8,937890158	9,624176169	0,233135	-0,209274095	0,302528672	0,142590374	MPDU1	lichol utili	ein-coding	th protein	G00000129255	
2109	0,000485857	0,0003236	0,08527787	-0,815459	9,554582156	10,37004074	0,14473	-0,168074741	0,302164616	-0,107086535	ETFB	r flavoprot	ein-coding	th protein	G00000105379	
55571	0,000732347	0,0003237	0,083526725	0,6945759	8,844042038	8,14946617	0,41868	0,183095881	0,024344981	0,398474124	CNOT11	cription cor	ein-coding	th protein	G00000158435	
11098	0,00075792	0,00032431	0,081976957	-0,970426	9,380956797	10,35138267	0,412775	-0,14964919	0,035068504	-0,469108945	PRSS23	ease, serin	ein-coding	th protein	G00000150687	
51107	0,000400382	0,0003319	0,082218226	-1,223643	8,402638814	9,626281864	0,020676	-0,656691504	0,41410726	-0,088998667	APH1A	, gamma-s	ein-coding	th protein	G00000117362	
54952	0,000646177	0,00033507	0,081375893	-0,755487	8,462255227	9,217741795	0,064174	-0,375761877	0,065128455	-0,390548718	TRNAU1AP	eine 1 assc	ein-coding	th protein	G00000180098	
283237	0,000669077	0,00034695	0,081080911	0,5831423	10,40103379	9,817891538	0,071845	0,295758947	0,691156379	-0,025241105	TTC9C	otide repea	ein-coding	th protein	G00000162222	
25923	0,000733392	0,00034894	0,080036883	0,6579541	9,864355178	9,206401121	0,004281	0,544793986	0,220711527	-0,115335796	ATL3	astin GTPas	ein-coding	th protein	G00000184743	
8863	0,000449075	0,00035312	0,079522485	-0,51484	6,882687758	7,397527403	0,206783	-0,218341358	0,379840917	0,135839177	PER3	circadian	ein-coding	th protein	G00000049246	
4493	0,000566529	0,00038634	0,085450056	-1,219714	12,64025105	13,85996529	0,073336	-0,685154595	0,588298999	-0,053586048	MT1E	allothionei	ein-coding	th protein	G00000169715	
3281	0,000193673	0,00038832	0,0843806	-1,107757	10,18105528	11,28881251	7,32E-05	-0,318249538	0,002944522	-0,252983882	HSBP1	actor bindi	ein-coding	th protein	G00000230989	
55855	0,000887321	0,00038975	0,083230712	-0,964407	8,481840415	9,446247135	0,000477	-0,813956825	0,03589794	-0,235921888	FAM45BP	milarity 45	seudogen	seudogen	G00000221930	
100506328	0,0007889	0,00042095	0,088369724	2,6023519	2,739951205	0,13759926	0,494653	1,044723952	0,487317116	0,75942877	LINC01127	on-protein	n-coding R	long non-c	G00000281162	
93408	0,000985855	0,00042197	0,087108681	1,0119048	8,129573763	7,117669012	0,155838	0,570039059	0,372995189	-0,107136867	MYL10	in light cha	ein-coding	th protein	G00000106436	
3655	0,000854751	0,00042966	0,087242523	0,8389947	10,58016174	9,741166993	0,098983	0,197255259	0,000311227	0,738633907	ITGA6	n subunit a	ein-coding	th protein	G00000091409	
221908	0,000156296	0,00043588	0,087076403	0,509604	11,1269474	10,6173434	0,594682	0,075657034	0,159632806	0,21771308	PPP1R35	tase 1 regu	ein-coding	th protein	G00000160813	
23286	0,001031572	0,00045434	0,089325259	-0,619053	7,841626872	8,460680205	0,023265	-0,3737894	0,02681468	-0,241456778	WWC1	2 domain c	ein-coding	th protein	G00000113645	
8470	0,000574635	0,00045452	0,087964022	-1,352434	5,918881391	7,271315836	0,137075	-0,411592351	0,000616293	-0,914769127	SORBS2	H3 domain	ein-coding	th protein	G00000154556	
9306	0,001006328	0,00045651	0,086988949	0,7899919	6,92412343	6,134131501	0,06463	0,401314756	0,095771704	0,192176963	SOCS6	of cytokine	ein-coding	th protein	G00000170677	
5613	0,000140004	0,00050864	0,09545437	-1,200563	6,397935427	7,598498591	0,00827	-1,06160157	0,205244705	0,240831855	PRKX	n kinase, X	ein-coding	th protein	G00000183943	
120	0,000925214	0,0005261	0,097257331	-1,266193	6,974664128	8,240857257	0,045253	-0,466665159	0,048816438	-0,285325768	ADD3	adducin 3	ein-coding	th protein	G00000148700	
8850	0,000208503	0,00053457	0,097370316	-0,819066	7,062286358	7,881352004	0,057321	-0,271169906	0,458629588	-0,06151107	KAT2B	cetyltransfe	ein-coding	th protein	G00000114166	
123283	0,001039188	0,00053897	0,096748844	-0,982408	4,486350678	5,468759053	0,04737	-0,570880493	0,304194718	0,19584707	TARSL2	RNA synthe	ein-coding	th protein	G00000185418	
9806	0,000677946	0,00055631	0,098434083	0,5775877	6,274612235	5,69702452	0,330705	0,173278806	0,009643098	0,162665702	SPOCK2	and kazal li	ein-coding	th protein	G00000107742	
81790	0,000532652	0,000562	0,098040216	-0,809338	7,13194596	7,941284328	0,117297	-0,195431676	0,038148035	-0,21095747	RNF170	nger prote	ein-coding	th protein	G00000120925	
822	0,001231734	0,00057163	0,098335783	-0,980259	11,80826358	12,78852246	0,023818	-0,415665067	0,83625439	-0,018975832	CAPG	in protein,	ein-coding	th protein	G00000042493	

23250	0,001048208	0,0005727	0,097170299	-0,854036	7,070970511	7,925006256	0,02074	-0,515006094	0,29206751	-0,130411018	ATP11A	holipid tra	ein-coding	th protein	G00000068650
91664	0,001261808	0,00059319	0,099287233	-0,744632	7,622789131	8,36742102	0,478489	-0,070304809	0,062469045	-0,213798154	ZNF845	nger prote	ein-coding	th protein	G00000213799
116832	0,000692968	0,00060942	0,100644115	-0,999357	9,994132206	10,99348891	0,022965	-0,632084486	0,186320146	0,216949867	RPL39L	nal protein	ein-coding	th protein	G00000163923
79932	0,001305373	0,00061345	0,09997637	-0,743984	7,464016196	8,208000176	0,0145	-0,572075065	0,061927355	-0,191139739	KIAA0319L	IAA0319 lik	ein-coding	th protein	G00000142687
1200	0,00049088	0,00061703	0,097981346	-0,532081	8,219931941	8,752013432	0,202886	0,075063117	0,010023654	-0,292434597	TPP1	tidyl pepti	ein-coding	th protein	G00000166340
6774	0,001318504	0,00062909	0,098631738	0,9242249	9,440853078	8,516628215	0,00918	0,967958218	0,501623572	-0,053547351	STAT3	nd activato	ein-coding	th protein	G00000168610
401509	0,001105087	0,0006465	0,100093273	-0,895802	5,744474259	6,640276416	0,233313	-1,063548271	0,10762045	-0,348521567	ZNF658B	otein 658B	pseudogene	pseudogene	G00000198416
5303	0,001363685	0,00068194	0,104277469	-0,893116	6,673329774	7,566445881	0,039727	-0,730863408	0,091319892	-0,176277602	PIN4	ns isomeras	ein-coding	th protein	G00000102309
92675	0,00071432	0,00068837	0,103977739	-1,838309	8,731795311	10,57010385	0,002487	-1,44294561	0,853936305	-0,017380196	DTD1	l-tRNA dea	ein-coding	th protein	G00000125821
9542	0,000832077	0,00069661	0,1039539	0,8660841	5,667275609	4,801191503	0,154629	0,41659189	0,298378567	0,26273894	NRG2	neuregulin	ein-coding	th protein	G00000158458
3126	0,000506233	0,00070674	0,104210557	-1,120484	9,833442347	10,95392654	0,105383	-0,273565399	0,001553564	-0,76972049	HLA-DRB4	ility compl	ein-coding	th protein	G00000227357
1907	0,000582271	0,00071332	0,103943152	-0,966993	7,496650521	8,463643313	0,100876	-0,323659528	0,007177912	-0,719726752	EDN2	endothelin	ein-coding	th protein	G00000127129
116841	0,001089942	0,00072501	0,10441814	-0,706575	7,631914565	8,338489107	0,560535	-0,199782541	0,387891931	-0,265251748	SNAP47	e associate	ein-coding	th protein	G00000143740
90861	0,00138571	0,000754	0,106125477	-0,573125	9,768687029	10,34181234	0,039148	-0,215749383	0,049028638	-0,180370085	HN1L	neurologic	ein-coding	th protein	G00000206053
9813	0,000607981	0,0007602	0,105796195	0,7337374	9,631493008	8,89775563	0,180489	0,322138086	0,041763278	0,352796125	EFCAB14	ium bindin	ein-coding	th protein	G00000159658
56477	0,000430725	0,00076069	0,10468785	-1,900598	5,524910467	7,425508411	0,097331	-1,028305579	0,011013745	-0,428559278	CCL28	chemokine	ein-coding	th protein	G00000151882
4860	0,001329917	0,00076335	0,103898609	0,7181127	10,49089917	9,772786513	0,002363	0,474870129	0,01766958	0,293445718	PNP	eoside pho	ein-coding	th protein	G00000198805
729515	9,17244E-05	0,00076784	0,10337365	-0,603393	6,74940688	7,352800187	0,039346	-0,607631847	0,765973643	0,033151577	TMEM242	mbrane pr	ein-coding	th protein	G00000215712
23516	0,001296628	0,00080436	0,107127213	1,0166193	8,171146396	7,154527134	0,453855	0,242172401	0,053572842	0,649772335	SLC39A14	r family 39	ein-coding	th protein	G00000104635
283254	0,001325259	0,00083544	0,108923716	0,5774595	7,007928221	6,430468671	0,525679	0,106180858	0,442745407	0,075986223	HARBI1	transposas	ein-coding	th protein	G00000180423
5471	0,001514075	0,00085104	0,108669142	0,6639508	8,924607882	8,26065711	0,037417	0,203716903	0,001606972	0,486559874	PPAT	ophosphat	ein-coding	th protein	G00000128059
4066	0,000826861	0,00085297	0,107804604	0,5515104	7,078331493	6,526821104	0,013004	0,456385787	0,867951014	-0,011173219	LYL1	-loop-helix	ein-coding	th protein	G00000104903
51725	0,000165198	0,00085345	0,106775917	1,7017331	4,504691998	2,802958945	0,521332	-0,344506594	0,047958933	1,40040964	FBXO40	ox protein	ein-coding	th protein	G00000163833
285704	0,001622387	0,00085754	0,106214058	0,7464901	8,816637349	8,070147247	0,545936	0,093017594	0,207139329	0,211563428	RGMB	e molecule	ein-coding	th protein	G00000174136
11212	0,0011631	0,00086602	0,106203095	0,6757301	7,292675485	6,616945417	0,013338	0,455082606	0,034685146	0,235647567	PROSC	transcribe	ein-coding	th protein	G00000147471
638	0,001158344	0,00088145	0,105996582	-1,242488	7,527362322	8,769850305	0,04392	-0,326548436	0,028404477	-0,448880234	BIK	interacting	ein-coding	th protein	G00000100290
149371	0,000241417	0,00090397	0,10765919	-0,523004	7,538306372	8,061310532	0,204774	-0,281600535	0,68877999	0,024292876	EXOC8	omplex con	ein-coding	th protein	G00000116903
26130	0,001693959	0,00090967	0,107305618	0,673947	9,016906962	8,342959925	0,163292	0,199168003	0,087240446	0,252230973	GAPVD1	protein an	ein-coding	th protein	G00000165219
387522	0,001680752	0,00091652	0,107093921	1,5108658	11,82180705	10,31094123	0,018955	1,154912794	0,100971142	0,348651753	M189-UBE	UBE2V1 re	other	eadthroug	G00000124208
5202	0,001285874	0,00092316	0,106862019	0,6315465	13,42583686	12,79429035	0,046523	0,21599465	0,004027864	0,339849835	PFDN2	oldin subu	ein-coding	th protein	G00000143256
84572	0,001668315	0,00096458	0,108611266	-0,583139	9,524709215	10,10784803	0,11445	-0,413169166	0,01112823	-0,244536011	GNPTG	osphate tr	ein-coding	th protein	G00000090581
7694	0,001692389	0,00096547	0,107732258	0,5882839	5,813425866	5,225141997	0,255732	0,774192545	0,70700563	0,045322699	ZNF135	nger prote	ein-coding	th protein	G00000176293
54838	0,001069177	0,00097638	0,107976627	0,8505728	8,811807819	7,961235015	9,99E-05	0,6198888	0,794067654	0,021201453	WBP1L	binding p	ein-coding	th protein	G00000166272
1396	0,000777753	0,00098345	0,107796037	0,5659253	6,095773716	5,529848378	0,06179	0,350091679	0,766583204	-0,03705222	CRIP1	ne rich pro	ein-coding	th protein	G00000213145
152816	0,001487556	0,0009963	0,108246797	-1,919065	6,324771585	8,243836204	0,047723	-0,947807971	0,279618755	-0,188437388	C4orf26	4 open rea	ein-coding	th protein	G00000174792
5919	0,001223639	0,00103155	0,111101844	-1,276343	11,25998198	12,53632535	0,122441	-0,2736705	0,003936052	-0,859122557	RARRES2	d receptor	ein-coding	th protein	G00000106538
100130817	0,000985098	0,00103664	0,110687509	1,036147	5,678501629	4,642354624	0,425466	0,234155156	0,105944562	0,25101647					
85365	0,001938989	0,00103902	0,10999355	0,684382	9,395714342	8,711332354	0,106008	0,288818701	0,085367899	0,297150703	ALG2	/1,6-mann	ein-coding	th protein	G00000119523
8705	0,001770371	0,00104341	0,109521889	-0,699938	9,173933273	9,873871232	0,089232	0,425295904	0,000331901	-1,009059876	B3GALT4	alactosyltr	ein-coding	th protein	G00000235863
5784	0,000816547	0,00105274	0,109572611	1,0076602	9,564816275	8,557156047	0,002322	0,574836904	0,065396509	0,241545426	PTPN14	sphatase, r	ein-coding	th protein	G00000152104
146754	0,001940946	0,0010911	0,112619563	-0,611941	6,754702597	7,366643123	0,235594	-0,190052596	0,016557722	-0,258473617	DNAH2	onemal hea	ein-coding	th protein	G00000183914
150759	0,001631332	0,00110623	0,113237017	0,5559793	9,296218151	8,740238838	0,355595	-0,484157295	0,252967335	-0,072937152	LINC00342	on-protein	n-coding R	long non-c	G00000232931
79089	0,002032158	0,00111827	0,112608481	0,5334749	8,135235971	7,60176107	0,142418	0,300244948	0,306912648	-0,063476028	TMUB2	biquitin lik	ein-coding	th protein	G00000168591
972	0,00111594	0,00112105	0,111978065	-0,98534	10,70799637	11,69333671	0,293869	-0,251340976	0,004069527	-0,591773296	CD74	D74 molec	ein-coding	th protein	G00000019582
837	0,001874309	0,00113631	0,112594114	-0,778746	9,359169071	10,13791486	0,030986	-0,635262867	0,040773274	-0,271564305	CASP4	caspase 4	ein-coding	th protein	G00000196954
54470	0,001681628	0,00113686	0,111755121	0,5748975	8,960264051	8,385366517	0,163341	0,167288914	0,055253949	-0,185470104	ARMCX6	eat contain	ein-coding	th protein	G00000198960
55847	0,001850474	0,00114096	0,111274906	-0,685895	11,91490347	12,60079848	0,061241	-0,179992495	0,040582337	-0,233424829	CISD1	ron sulfur c	ein-coding	th protein	G00000122873
7045	0,001854402	0,00114796	0,111082306	-1,561896	7,636590512	9,198486711	0,136087	-0,536479526	0,045286604	-0,511517479	TGFBI	rowth fact	ein-coding	th protein	G00000120708
83440	0,002218064	0,00119616	0,114849108	-0,972915	6,312540254	7,285455206	0,022614	-0,496213395	0,887389344	0,014890384	ADPGK	endent glu	ein-coding	th protein	G00000159322
79627	0,00165358	0,00120202	0,113649818	-1,224202	10,14410369	11,36830546	0,094407	-0,412753659	0,004999685	-0,69042678	OGFRL1	th factor re	ein-coding	th protein	G00000119900
240	0,00020473	0,00120508	0,11307662	-0,660698	6,376604796	7,037302445	0,233928	-0,180804324	0,04921047	-0,313857946	ALOX5	nate 5-lipo	ein-coding	th protein	G00000012779
4828	0,000502471	0,00122197	0,112949334	-0,759478	10,08709027	10,84656816	0,025526	-0,616755176	0,005784183	-0,527528421	NMB	euromedin	ein-coding	th protein	G00000197696
79956	0,001104756	0,00122845	0,112707376	-0,907352	5,391918064	6,299270512	0,275954	-0,367320337	0,28844226	-0,10589091	ERMP1	iculum me	ein-coding	th protein	G00000099219
8678	0,001203651	0,00122898	0,111927388	0,5643791	8,999926277	8,435547183	0,00307	0,246520703	0,000350614	0,484242861	BECN1	beclin 1	ein-coding	th protein	G00000126581
7105	0,001833415	0,00124427	0,112492541	-0,823571	8,974282055	9,79785313	0,007958	-0,309247942	0,048090918	-0,214401372	TSPAN6	etraspanin	ein-coding	th protein	G00000000003
56548	0,002276016	0,00124979	0,112173112	0,7899793	8,218444375	7,428465028	0,010473	0,431479656	0,142945588	0,176054484	CHST7	ate sulfotr	ein-coding	th protein	G00000147119
10969	0,000523054	0,00126355	0,112591551	0,5167267	12,64295468	12,12622795	0,631209	0,058643704	0,017565055	0,414222431	EBNA1BP2	binding pr	ein-coding	th protein	G00000117395
3431	0,001281189	0,0012893	0,114065859	-0,644749	6,763918434	7,408667121	0,638282	-0,050215511	0,084112617	-0,154653125	SP110	uclear bod	ein-coding	th protein	G00000135899
27102	0,001376522	0,00128935	0,113260959	-1,693802	8,280743225	9,974545501	0,003029	-1,386361419	0,664387371	-0,095963703	EIF2AK1	initiation f	ein-coding	th protein	G00000086232

4499	0,001528575	0,00129566	0,113014156	-1,48416	11,66122347	13,14538366	0,093099	-1,084039392	0,597526709	-0,083757211	MT1M	allothionei	ein-coding	th protein	G00000205364
6208	0,001391563	0,00132443	0,113919096	0,6463373	12,12002134	11,47368406	0,159866	0,185736995	0,036022745	0,352853457	RPS14	omal protei	ein-coding	th protein	G00000164587
4711	0,000236108	0,00132736	0,113383902	-1,019526	9,283464829	10,30299038	0,001056	-0,321269363	0,022969295	-0,128954097	NDUFB5	e oxidored	ein-coding	th protein	G00000136521
29914	0,002338405	0,00134431	0,113268905	0,8214731	10,50930794	9,687834878	0,030135	0,322032625	0,025495508	0,313927432	UBIAD1	ferase don	ein-coding	th protein	G00000120942
57089	0,001457079	0,0013732	0,11415012	0,62393	7,672256754	7,048326716	0,117845	0,278462874	0,007335837	0,484264237	ENTPD7	osphate di	ein-coding	th protein	G00000198018
3241	0,002552746	0,00139161	0,114909122	1,0513557	11,65897356	10,60761786	0,018923	0,369885258	0,129293695	0,228645513	HPCAL1	pocalcin lik	ein-coding	th protein	G00000115756
51506	0,000924327	0,0014013	0,114943275	0,7127438	11,28073851	10,56799475	0,088092	0,592113713	0,01262078	-1,319957673	UFC1	odifier conj	ein-coding	th protein	G00000143222
100130502	0,001767223	0,0014054	0,114521404	-0,597089	7,277177015	7,874266293	0,094615	-0,306823716	0,007920742	-0,258193653					
135114	0,002107626	0,00141918	0,113406107	0,9690546	7,685201971	6,716147333	0,028207	0,485418358	0,072530933	0,271310631	HINT3	ucleotide b	ein-coding	th protein	G00000111911
124808	0,001721689	0,00143052	0,112856079	-1,053314	8,201356479	9,254670955	0,067736	-0,916581544	0,125963361	0,2438426	CCDC43	domain co	ein-coding	th protein	G00000180329
1326	0,002253	0,00147101	0,113874286	-1,486101	5,380099134	6,866199917	0,087389	-0,675608143	0,069749484	-0,571785937	MAP3K8	protein kin	ein-coding	th protein	G00000107968
317	0,002106377	0,00153755	0,11755542	-1,204272	7,059290395	8,263562543	0,038494	-0,738728858	0,007042909	-0,790312279	APAF1	tidase acti	ein-coding	th protein	G00000120868
4580	0,002018245	0,00153816	0,116880935	0,5144416	10,49119601	9,976754377	0,012313	0,305355186	0,179839197	-0,10620811	MTX1	metaxin 1	ein-coding	th protein	G00000173171
11262	0,002806309	0,00154979	0,11633696	-0,899519	5,960483384	6,860002623	0,185581	-0,345478866	0,25350889	-0,133717412	SP140	uclear body	ein-coding	th protein	G00000079263
23229	0,002832174	0,00156609	0,116152949	0,5497177	7,370851823	6,821134102	0,04961	0,381150652	0,540519266	0,036758762	ARHGEF9	ucleotide e	ein-coding	th protein	G00000131089
2498	0,002734077	0,00159122	0,115934021	-1,152387	13,80807299	14,9604597	0,021858	-0,325431588	0,124067404	-0,235147191	FTH1P3	y chain 1 p	oseudogen	oseudogen	G00000213453
2820	0,001541546	0,00159419	0,115470844	-1,132135	6,701840076	7,833975543	0,032572	-0,959883128	0,417484709	0,105051943	GPD2	osphate de	ein-coding	th protein	G00000115159
5281	0,000452661	0,00160301	0,115435352	-1,290056	6,897523924	8,187579806	0,004792	-1,071832403	0,045995244	-0,418381435	PIGF	lycan anch	ein-coding	th protein	G00000151665
51201	0,0021224	0,00161291	0,114812574	-0,971447	8,966512805	9,937959691	0,068856	-0,28586139	0,993930298	0,000599778	ZDHHC2	DHHC-type	ein-coding	th protein	G00000104219
55344	0,002973515	0,00163856	0,115313613	0,7787732	7,26637833	6,487605093	0,004557	0,598170426	0,047477661	0,267423355	PLCXD1	phospholi	ein-coding	th protein	G00000182378
83931	0,001440354	0,00164421	0,115056926	0,9301423	8,575875505	7,645733237	0,450363	0,040578141	0,007139015	0,659365415	STK40	hreonine k	ein-coding	th protein	G00000196182
6612	0,003020577	0,00166335	0,115742973	-0,737523	11,86726288	12,60478581	0,018597	-0,436870498	0,710115901	-0,042907106	SUMO3	quitin-like r	ein-coding	th protein	G00000184900
645843	0,002100286	0,00167264	0,115738996	-0,679531	5,87895985	6,558490875	0,056503	-0,630123295	0,783453547	-0,060040369	TMEM14EP	e protein 1	oseudogen	oseudogen	G00000221962
284099	0,002595735	0,00168654	0,116052606	-1,531482	3,07186411	4,603346451	0,340016	-0,323543336	0,73063779	0,096829341	C17orf78	17 open rea	ein-coding	th protein	G00000278505
6584	0,000652482	0,00168982	0,115000303	-0,77968	6,772168351	7,551848324	0,027186	-0,412464177	0,102785188	-0,15165597	SLC22A5	er family 2	ein-coding	th protein	G00000197375
2019	0,002682502	0,00177475	0,120120398	1,188276	3,63800533	2,449729292	0,212192	1,281303995	0,428245799	0,29061142	EN1	iled home	ein-coding	th protein	G00000163064
10398	0,003187062	0,0017816	0,119928395	1,5587383	12,05335517	10,49461683	0,030679	0,68503475	0,602736547	0,103768887	MYL9	sin light ch	ein-coding	th protein	G00000101335
4501	0,00312554	0,00179983	0,11985312	-0,946501	9,84674773	10,7932484	0,029827	-0,606992881	0,490519145	-0,125841245	MT1X	allothionei	ein-coding	th protein	G00000187193
9693	0,003061605	0,00180609	0,119626671	-0,801599	7,106401578	7,908000725	0,111801	-0,58881361	0,285175775	-0,118847221	RAPGEF2	cleotide ex	ein-coding	th protein	G00000109756
2107	0,002839142	0,00181958	0,119878856	0,8290236	10,38010511	9,551081503	0,077777	0,445004503	0,062795106	0,275833792	ETF1	lation tern	ein-coding	th protein	G00000120705
5087	0,000630491	0,00182774	0,119779258	1,6209099	8,815890713	7,194980845	0,178913	0,536748883	0,748287087	0,071931942	PBX1	X homeobc	ein-coding	th protein	G00000185630
170371	0,002833709	0,00184058	0,119986215	0,9966742	5,037382697	4,040708465	0,097017	1,179461217	0,580606833	-0,282961285	C10orf128	0 open rea	ein-coding	th protein	G00000204161
8882	0,001261982	0,0018976	0,122414405	0,7163108	10,77586616	10,05955531	0,050604	0,216077831	0,048308357	0,254831038	ZPR1	R1 zinc fing	ein-coding	th protein	G00000109917
64061	0,002653135	0,00190284	0,122116692	1,5826954	8,036651499	6,453956093	0,013475	0,852449883	0,02063184	0,741505622	TSPYL2	TSPY like 2	ein-coding	th protein	G00000184205
8880	0,001806094	0,00192966	0,122567585	-0,537015	9,458976357	9,995991587	0,066311	-0,189160048	0,059624003	-0,108083539	FUBP1	element bir	ein-coding	th protein	G00000162613
64145	0,000531072	0,00193165	0,122068114	1,2269446	8,040882812	6,813938204	0,274347	0,374730065	0,100370079	0,346260511	RBSN	osyn, RAB e	ein-coding	th protein	G00000131381
84981	0,002838761	0,00194495	0,122284549	0,7178943	9,332511929	8,614617637	0,042478	0,437764016	0,09396865	-0,170373794	MIR22HG	R22 host ge	n-coding R	long non-c	G00000186594
10522	0,002894193	0,0019664	0,121778866	-0,512544	9,01848812	9,531031796	0,249227	-0,258529331	0,791374225	-0,030043484	DEAF1	transcriptio	ein-coding	th protein	G00000177030
51071	0,00316251	0,00197586	0,121756016	-1,356614	7,056690351	8,413304053	0,008084	-0,650649251	0,802067088	-0,040989204	DERA	e-phospha	ein-coding	th protein	G00000023697
145482	0,003534647	0,0019781	0,12129053	-0,948353	6,197850479	7,146203719	0,127895	-0,550856888	0,150635677	0,224640001	PTGR2	landin red	ein-coding	th protein	G00000140043
5791	0,003575993	0,00200529	0,122351845	0,5898998	8,419779009	7,829879239	0,023464	0,554342901	0,453562194	0,058727472	PTPRE	hosphatas	ein-coding	th protein	G00000132334
6722	0,001217726	0,00202629	0,122427044	0,9720677	9,062762397	8,090694648	0,01721	0,314450256	0,046913185	0,310522264	SRF	n response	ein-coding	th protein	G00000112658
55114	0,002762991	0,00204088	0,122709725	-0,539259	7,278416741	7,817675288	0,068243	-0,324089491	0,355857999	-0,072286714	ARHGAP17	e activating	ein-coding	th protein	G00000140750
81848	0,003598074	0,0020525	0,122222087	0,7206384	9,106009705	8,385371288	0,012074	0,47351574	0,375910063	0,107053376	SPRY4	(signaling a	ein-coding	th protein	G00000187678
221718	8,61102E-05	0,0020636	0,121712939	-4,500612	0	4,500611964	0,309648	-0,889503063	0,023579808	-3,611108902	LINC00518	on-protein	n-coding R	long non-c	G00000183674
131873	0,003446101	0,00206451	0,121188999	2,3590928	2,78163879	0,422546009	0,897997	0,103569114	0,093963996	1,335835898	COL6A6	ype VI alph	ein-coding	th protein	G00000206384
2693	0,001786817	0,00208793	0,121413198	-0,898753	5,402755112	6,301507661	0,223577	-0,234410018	0,012019397	-0,500752346	GHSR	ne secreta	ein-coding	th protein	G00000121853
51397	0,000773125	0,00210982	0,1221126	1,3698672	9,492900853	8,123033613	0,016747	0,652164888	0,090216995	0,369551592	COMMD10	omain cont	ein-coding	th protein	G00000145781
57406	0,000916354	0,00212186	0,122238467	0,6013951	7,556572306	6,955177207	0,015516	0,341825482	0,033551858	0,291819461	ABHD6	e domain c	ein-coding	th protein	G00000163686
7113	0,003711359	0,00214682	0,12310382	-0,82191	6,358985231	7,180895578	0,267756	-0,340572406	0,139768735	-0,182150907	TMPRSS2	rane prote	ein-coding	th protein	G00000184012
51726	0,003590417	0,00215006	0,122721344	0,7910891	11,0796609	10,28857184	0,153994	0,360307751	0,035446693	0,419408599	DNAJB11	ein family	ein-coding	th protein	G00000090520
4615	0,003246583	0,00215218	0,122279146	-0,572657	8,036122459	8,60877961	0,13043	-0,118642056	0,078088494	-0,181881774	MYD88	tiation prin	ein-coding	th protein	G00000172936
79940	0,001196739	0,0021694	0,12213699	-0,694606	9,258418761	9,953024494	0,142014	-0,288417017	0,063864306	-0,404982703	LINC00472	on-protein	n-coding R	long non-c	G00000233237
2760	0,00025661	0,00217238	0,121751176	-0,757642	8,501610435	9,259252353	0,072764	-0,253495131	0,016111011	-0,5598037	GM2A	nglioside a	ein-coding	th protein	G00000196743
285381	0,001192245	0,00220207	0,122859	0,6681324	11,51168955	10,84355712	0,007682	0,393794518	0,421838391	-0,062688696	DPH3	nide biosy	ein-coding	th protein	G00000154813
3162	0,00298897	0,00222564	0,123617406	-1,345728	8,534729913	9,880458109	0,016388	-1,211443861	0,423732432	0,225650835	HMOX1	ne oxygena	ein-coding	th protein	G00000100292
6672	0,00268524	0,0022429	0,123468772	-0,870111	8,023395944	8,893506738	0,060629	-0,287718558	0,958351571	0,006519308	SP100	) nuclear a	ein-coding	th protein	G00000067066
80114	0,003895976	0,00224569	0,123075471	-1,197079	6,575983121	7,773062121	0,102546	-0,295273652	0,071327151	-0,399510069	BICC1	RNA bindir	ein-coding	th protein	G00000122870
2702	0,001976562	0,00226674	0,1226018	1,1460161	3,973434401	2,827418273	0,682117	0,135180556	0,111132982	0,643438402	GJA5	tion protei	ein-coding	th protein	G00000265107

26689	0,000129655	0,00227171	0,122336338	3,1278133	3,127813318	0	0,878669	-0,238831298	0,197323691	2,129181963	OR4D1	amily 4 sub	ein-coding	th protein	G00000141194
51241	0,00179682	0,00227652	0,122064493	-0,760433	10,98154377	11,74197719	0,166954	-0,446597855	0,64020992	-0,051312246	COX16	e c oxidase	ein-coding	th protein	G00000133983
10802	0,003121377	0,00227883	0,121661507	0,5156275	8,722725953	8,207098467	0,155603	0,179078005	0,901980666	-0,010352033	SEC24A	:OPII coat c	ein-coding	th protein	G00000113615
253430	0,000538854	0,00228915	0,12168783	-1,331443	6,169800316	7,501243047	0,054341	-0,717637555	0,111717695	-0,16735948	IPMK	phosphate	ein-coding	th protein	G00000151151
56986	0,003348317	0,0022912	0,121276413	-0,591323	7,025244466	7,616567444	0,029802	-0,671538782	0,849101754	0,016161333	DTWD1	omain cont	ein-coding	th protein	G00000104047
10437	0,002147516	0,0023073	0,121609267	-0,860055	10,08418195	10,94423723	0,194388	-0,208851738	0,072823437	-0,333637323	IFI30	somal thiol	ein-coding	th protein	G00000216490
9855	0,000725647	0,00231312	0,121399029	0,6001976	6,74934008	6,149142467	0,147295	0,245176737	0,039474956	0,325038087	FARP2	and plecks	ein-coding	th protein	G00000006607
64419	0,003470219	0,00232997	0,121255836	-0,545167	7,503141929	8,048308934	0,036878	-0,195681595	0,858169731	-0,012876173	MTMR14	rin related	ein-coding	th protein	G00000163719
51552	0,004001619	0,0023319	0,120848641	-0,90669	9,714026388	10,62071641	0,023031	-0,545084959	0,964871665	0,005695793	RAB14	ber RAS on	ein-coding	th protein	G00000119396
10055	0,003960288	0,00239047	0,122348306	0,5387486	9,92036946	9,381620843	0,487604	0,071462755	0,02848769	0,323785829	SAE1	vating enzy	ein-coding	th protein	G00000142230
7923	0,002789005	0,00239298	0,121972541	-0,626615	7,157994042	7,78460856	0,179059	-0,107087202	0,091040627	-0,185138023	HSD17B8	17-beta de	ein-coding	th protein	G00000204228
64398	0,001742161	0,00239675	0,121664208	-0,810776	7,797100722	8,607876869	0,001988	-0,602240311	0,613959183	-0,050711694	MPP5	palmitoylat	ein-coding	th protein	G00000072415
253827	0,003501692	0,00241546	0,122113473	1,0634802	8,766087364	7,702607165	0,118565	0,41097913	0,036117889	0,479975731	MSRB3	sulfoxide r	ein-coding	th protein	G00000174099
442240	0,003513842	0,00242791	0,121749165	0,5118509	10,08078092	9,568930036	0,226718	0,122977355	0,155335751	0,146347772	ZNF259P1	otein 259 p	seudogen	oseudogen	G00000219565
83734	0,002323018	0,00243436	0,12060757	-0,975035	5,533603723	6,508638425	0,018924	-1,040099685	0,699429323	0,075200561	ATG10	phagy relat	ein-coding	th protein	G00000152348
55283	0,004176305	0,00243843	0,120328022	-1,104187	5,482641467	6,586828859	0,060706	-0,481909321	0,101963912	-0,326826797	MCOLN3	mucolipin 3	ein-coding	th protein	G00000055732
4521	0,004236079	0,00249	0,121901324	-0,908344	9,502556606	10,41090079	0,02123	-0,557176292	0,471323923	0,089423014	NUDT1	lix hydrola	ein-coding	th protein	G00000106268
8853	0,003033275	0,00253253	0,123495556	-0,694683	8,295045955	8,98972894	0,028076	-0,465031748	0,534332975	0,065351488	ASAP2	n, ankyrin	ein-coding	th protein	G00000151693
51199	0,001934766	0,00262936	0,127215584	-0,705769	7,777308037	8,483077206	0,074218	-0,539824509	0,21249756	0,168446451	NIN	ninein	ein-coding	th protein	G00000100503
9189	0,00438263	0,00263312	0,126409644	-0,707585	6,939991826	7,64757648	0,048632	-0,672577124	0,271392177	-0,13308918	ZBED1	BED-type c	ein-coding	th protein	G00000214717
10237	0,001337464	0,0026372	0,126116879	0,7991456	11,72268999	10,92354443	0,025104	0,370361128	0,021975369	0,350309387	SLC35B1	r family 35	ein-coding	th protein	G00000121073
6945	0,004508568	0,00265825	0,126634684	0,5360118	10,13520296	9,5991912	0,00325	0,439831265	0,197789969	0,1095137	MLX	dimerizati	ein-coding	th protein	G00000108788
221687	0,004443159	0,002687	0,126064902	0,7879366	8,403402237	7,615465655	0,047584	0,669055841	0,060953212	0,618409729	RNF182	nger prote	ein-coding	th protein	G00000180537
28973	0,004533619	0,00271265	0,126311074	-0,580133	9,400245751	9,980378579	0,048215	-0,511084782	0,003706807	-0,485447619	MRPS18B	l ribosoma	ein-coding	th protein	G00000204568
5694	0,001582212	0,00273092	0,125743756	0,5478205	12,9689799	12,42115942	0,189617	0,16367938	0,177594777	0,144870427	PSMB6	ome subun	ein-coding	th protein	G00000142507
7039	0,002088801	0,00273608	0,125514649	0,5848125	8,423295278	7,838482732	0,33699	0,141090543	0,395465469	0,062479777	TGFA	ng growth f	ein-coding	th protein	G00000163235
307	0,003857453	0,00274116	0,125283624	-0,558201	9,794141803	10,3523433	0,045031	-0,101258762	0,007890388	-0,472401366	ANXA4	annexin A4	ein-coding	th protein	G00000196975
5530	0,000543706	0,00277519	0,125450201	-1,355349	8,657236134	10,01258484	0,000917	-0,807168895	0,011086096	-0,383839445	PPP3CA	ase 3 catal	ein-coding	th protein	G00000138814
3708	0,004640216	0,00278306	0,124443748	0,5116208	5,333074717	4,821453892	0,617082	-0,062911401	0,009396292	0,34979885	ITPR1	sphosphate	ein-coding	th protein	G00000150995
727936	0,000953287	0,00278453	0,124061469	-1,382547	7,399967828	8,782514716	0,01048	-0,758868187	0,004469419	-0,482703941	GXYLT2	xylosyltra	ein-coding	th protein	G00000172986
80335	0,003892168	0,00278691	0,123722578	0,7541048	9,535320551	8,781215753	0,040804	0,277863488	0,010312838	0,429186661	WDR82	epeat dom	ein-coding	th protein	G00000164091
64116	0,002935437	0,00279253	0,123089595	-1,27896	7,199100983	8,478061349	0,047325	-0,749149137	0,385426781	0,1025179	SLC39A8	er family 3	ein-coding	th protein	G00000138821
2764	0,001644693	0,00279321	0,122682995	-1,838164	7,971589552	9,809753887	0,000928	-1,14719691	0,605724921	-0,116629888	GMFB	uration fac	ein-coding	th protein	G00000197045
91947	0,003788933	0,00282889	0,123374926	0,586807	6,621414523	6,034607542	0,132869	0,403229863	0,676990539	-0,065892116	ARRDC4	domain cor	ein-coding	th protein	G00000140450
1975	0,00176516	0,00286959	0,124711108	-0,543604	10,30739054	10,85099453	0,118039	-0,392107363	0,058638865	-0,180660086	EIF4B	slation init	ein-coding	th protein	G00000063046
55622	0,004507911	0,00289461	0,124921798	0,5646484	8,328616877	7,76396847	0,003973	0,332400084	0,068717661	0,172480738	TTC27	ptide repe	ein-coding	th protein	G00000018699
4502	0,002226943	0,00291648	0,125428413	-0,50019	16,22382433	16,72401386	0,2989	-0,317082379	0,325400891	-0,168099271	MT2A	allothionei	ein-coding	th protein	G00000125148
50486	0,004419572	0,00291901	0,125102998	1,2181301	12,55531515	11,33718509	0,013182	0,807515453	0,054945127	0,419985703	G0S2	)/G1 switch	ein-coding	th protein	G00000123689
19	0,003308048	0,00292084	0,124749655	-1,00204	7,407340726	8,409380441	0,254977	-0,486400637	0,517060029	0,142782483	ABCA1	ette subfa	ein-coding	th protein	G00000165029
29969	0,00368009	0,00296065	0,125584076	-1,878326	6,594182244	8,472508691	0,112269	-1,169477303	0,59352853	-0,097125317	MDFIC	hibitor dor	ein-coding	th protein	G00000135272
7262	0,004906655	0,00296923	0,125518151	0,5884342	13,7238963	13,13546207	0,106173	0,207832844	0,107419534	0,216027172	PHLDA2	like domai	ein-coding	th protein	G00000181649
23456	0,000247399	0,00302113	0,126845916	-0,928796	7,249470509	8,178266349	0,064257	-0,456517809	0,336843913	-0,067490623	ABCB10	ette subfan	ein-coding	th protein	G00000135776
2589	0,001247775	0,00307562	0,128697645	-0,905142	9,059630146	9,964772094	0,005794	-0,313341498	0,012394085	-0,211000917	GALNT1	ylgalactos	ein-coding	th protein	G00000141429
54509	0,004553072	0,00309351	0,129010393	0,9082271	9,09809516	8,189868068	0,105702	0,28598318	0,268223535	0,128976019	RHOF	member F,	ein-coding	th protein	G00000139725
4498	0,003696032	0,00310004	0,128848784	-1,260971	9,5243421	10,78531318	0,1462	-0,484498457	0,313974261	-0,205987685	MT1JP	onein 1J, p	seudogen	oseudogen	G00000255986
933	0,00467805	0,003101	0,128457879	-0,727361	5,807411807	6,534772668	0,126745	-0,390886799	0,445519783	0,102551371	CD22	D22 molecu	ein-coding	th protein	G00000012124
670	0,002703566	0,00312305	0,128511398	-0,981566	7,911871376	8,893437213	0,107082	-0,237686847	0,023675533	-0,577622184	BPHL	nyl hydrola	ein-coding	th protein	G00000137274
65992	0,005180962	0,00316435	0,129779645	0,7300831	10,37282212	9,64273899	0,30959	0,188251616	0,283458036	0,127434748	DDRGK1	domain con	ein-coding	th protein	G00000198171
92659	0,005039784	0,00316598	0,129418117	0,6173227	7,821132641	7,203809981	0,006959	0,424792001	0,165013313	0,152187232	MAFG-AS1	ise RNA 1 (	n-coding R	long non-c	G00000265688
51626	0,005155066	0,00316975	0,129146125	-0,780661	6,530676332	7,311336884	0,23418	-0,327135734	0,019286914	-0,409461463	DYNC2LI1	c 2 light int	ein-coding	th protein	G00000138036
23479	0,003425977	0,00317213	0,128819253	0,8683897	10,76599046	9,897600776	0,017009	0,459009375	0,008205636	0,513009994	ISCU	uster asser	ein-coding	th protein	G00000136003
347	0,003130095	0,00318062	0,128322502	-1,014403	3,972128309	4,986531263	0,329649	-0,625214144	0,582741265	-0,144686276	APOD	olipoprotei	ein-coding	th protein	G00000189058
164	0,005255757	0,00318379	0,128033488	-0,629914	7,514048435	8,143962036	0,071682	-0,394766214	0,204257812	0,130650048	AP1G1	ein comple	ein-coding	th protein	G00000166747
401264	0,00049408	0,00320351	0,128409728	-0,906744	6,542401766	7,449146116	0,04778	-1,018062938	0,033300592	0,077379267	TRAM2-AS1	nse RNA 1 (	n-coding R	long non-c	G00000225791
4052	0,005320051	0,00322597	0,128892565	0,6445763	7,409513807	6,764937467	0,025412	0,380221055	0,236284835	0,144009924	LTBP1	owth factor	ein-coding	th protein	G00000049323
10207	0,003697917	0,00322676	0,12850986	-0,767645	6,781428627	7,549073734	0,064325	-0,601636489	0,103643152	-0,318832058	PATJ	polarity co	ein-coding	th protein	G00000132849
3127	0,004081683	0,0032727	0,129506421	-1,248762	9,235278035	10,48403958	0,0065	-0,426170593	0,006806709	-0,774934036	HLA-DRB5	ility compl	ein-coding	th protein	G00000198502
286827	0,004964618	0,00328025	0,129391762	0,6222111	8,427983164	7,805772042	0,110734	0,311554385	0,196459931	0,145460765	TRIM59	motif cont	ein-coding	th protein	G00000213186
133	0,005393412	0,00328823	0,129294768	-0,960906	7,844022281	8,804928138	0,048374	-0,519708062	0,013816521	-0,700799768	ADM	renomedul	ein-coding	th protein	G00000148926

160518	0,004818863	0,00328983	0,128948484	-0,588805	6,311662759	6,900468208	0,049072	-0,468859595	0,043501413	0,210583405	DENND5B	main contain-coding	th protein	G00000170456
339487	0,001752602	0,00329188	0,128621584	0,661685	10,71021714	10,04853209	0,324834	0,297728371	0,414348982	0,090622136	ZBTB8OS	nain contain-coding	th protein	G00000176261
55152	0,004490756	0,00330279	0,128642373	-0,725441	8,365088375	9,090529264	0,027305	-0,575172146	0,718349192	-0,031347245	DALRD3	binding dorein-coding	th protein	G00000178149
23385	0,003408785	0,00332546	0,128715523	-0,610695	8,469256702	9,07995206	0,970755	-0,003904381	0,344518725	-0,104163568	NCSTN	nicastrin ein-coding	th protein	G00000162736
55304	0,001757607	0,00332735	0,128387805	-1,46094	6,422504788	7,883445051	0,083231	-0,412829065	0,163436353	-0,152480931	SPTLC3	ferase longein-coding	th protein	G00000172296
125962	0,003820337	0,00333509	0,12828669	-1,094788	4,550468506	5,645256761	0,136786	-0,530732381	0,289761207	0,130076738	OR7G1	amily 7 subein-coding	th protein	G00000161807
3295	0,00110326	0,00337185	0,128503564	-0,858939	8,266527749	9,125467003	0,057083	-0,202712865	0,091924825	-0,218691344	HSD17B4	17-beta dein-coding	th protein	G00000133835
358	0,001269929	0,00338114	0,128462319	0,9199979	8,047274628	7,127276724	0,096368	0,568619542	0,203765749	-0,17346836	AQP1	1 (Colton bein-coding	th protein	G00000240583
10672	0,001597009	0,00339556	0,128615448	0,8866075	9,482581313	8,59597378	0,003633	0,525130413	0,900356728	0,007176976	GNA13	in subunit ein-coding	th protein	G00000120063
79711	0,005525091	0,00339791	0,128312096	0,5119428	8,091466104	7,579523351	0,274116	0,087111941	0,024270605	0,267572423	IPO4	importin 4ein-coding	th protein	G00000196497
10668	0,005625787	0,00347104	0,130279336	0,5267345	8,961056263	8,434321793	0,012896	0,433220183	0,385400572	0,075903998	CGRRF1	tor with rinein-coding	th protein	G00000100532
729085	0,000620463	0,00348648	0,13046327	0,7130866	5,497359456	4,784272823	0,283272	0,280856484	0,617564125	-0,101077519	FAM198A	nce similarein-coding	th protein	G00000144649
10449	0,001795756	0,00351175	0,131013412	-0,563191	8,817392082	9,380583551	0,119233	-0,194674804	0,258854725	-0,1072921	ACAA2	oA acyltranein-coding	th protein	G00000167315
84869	0,005076234	0,00353315	0,131022401	0,7394639	8,893377554	8,153913622	0,209672	0,302495678	0,275080514	0,167347184	CBR4	onyl reductein-coding	th protein	G00000145439
58489	0,002048556	0,00353719	0,130780492	-1,915202	7,492661812	9,407864223	0,017826	-1,521501978	0,857264173	-0,067923173	ABHD17C	domain coein-coding	th protein	G00000136379
54602	0,003829099	0,00353847	0,13005137	-1,020701	8,393559783	9,41426123	0,0225	-0,665829663	0,130206375	-0,188234444	NDFIP2	ly interactiein-coding	th protein	G00000102471
55589	0,005698361	0,00358246	0,130506533	0,7661485	9,436096114	8,669947637	0,106135	0,233176894	0,011147583	0,612978433	BMP2K	inducible kein-coding	th protein	G00000138756
128061	0,004944847	0,00358585	0,13024709	-0,555229	7,289107973	7,84433699	0,64756	-0,097224173	0,423280764	0,129125747	C1orf131	l open reacein-coding	th protein	G00000143633
3122	0,003546631	0,00359172	0,130078578	-1,894849	9,530599507	11,42544849	0,022618	-1,296649879	0,012967845	-0,846043873	HLA-DRA	ility compein-coding	th protein	G00000204287
64146	0,004035785	0,00360791	0,130283929	-0,764623	8,946609408	9,711232451	0,055501	-0,332803445	0,048174739	-0,278847026	PDF	rmylase (mein-coding	th protein	G00000258429
6711	0,003839694	0,00360872	0,129934413	-0,940228	10,05081755	10,9910457	0,312565	-0,118968969	0,571968664	-0,086145431	SPTBN1	ta, non-eryein-coding	th protein	G00000115306
6102	0,005150611	0,00362227	0,130044173	1,2813028	9,182414897	7,901112106	0,070468	0,803797443	0,097612356	0,421902108	RP2	itosa 2 (X-liein-coding	th protein	G00000102218
488	0,00190352	0,00362853	0,129892472	0,5867759	10,84104707	10,25427113	0,140123	0,171762618	0,004418964	0,381450329	ATP2A2	plasmic retein-coding	th protein	G00000174437
9412	0,005534501	0,00364754	0,130196641	0,6595835	8,727534043	8,067950585	0,006404	0,367358099	0,360718181	0,085022232	MED21	complex sein-coding	th protein	G00000152944
9848	0,004788161	0,00365532	0,130099553	1,1595988	6,162714468	5,003115703	0,681258	0,200999319	0,282786447	0,532682144	MFAP3L	associatedein-coding	th protein	G00000198948
29099	0,005497377	0,00365955	0,12987692	-0,658209	7,822914895	8,48112399	0,044528	-0,630835799	0,015077963	-0,369914652	COMMD9	lomain conein-coding	th protein	G00000110442
676	0,00028842	0,00368587	0,130065652	-2,120904	0	2,120903955	0,794421	-0,171892225	0,707082202	-0,091543363	BRDT	nain testis ein-coding	th protein	G00000137948
100132474	0,000295294	0,00376623	0,132148442	4,4701725	4,470172504	0	0,866986	0,232363491	0,187222864	2,181875146				
55233	0,002785783	0,00377843	0,132202087	0,6572006	10,74389463	10,08669408	0,030584	0,363089354	0,080253663	0,205548682	MOB1A	inase activein-coding	th protein	G00000114978
139221	0,003008114	0,00380828	0,132497933	1,153817	2,382256389	1,228439428	0,961198	0,050377385	0,241378712	0,435228414	MUM1L1	MUM1 like ein-coding	th protein	G00000157502
9636	0,006035399	0,00382297	0,132636264	1,2709363	13,62610957	12,35517329	0,094397	0,645015288	0,13560201	0,452429457	ISG15	iquitin-likeein-coding	th protein	G00000187608
23180	0,002470588	0,00383135	0,132555595	-0,563326	8,543851325	9,107177787	0,166293	-0,12847817	0,705949911	0,031934924	RFTN1	lipid raft lein-coding	th protein	G00000131378
10720	0,004237699	0,00384156	0,132538697	-0,816625	4,978062427	5,794687569	0,241413	-0,25719367	0,295357813	0,129655481	UGT2B11	insferase fein-coding	th protein	G00000213759
127428	0,005434793	0,00386017	0,132810876	-0,916197	5,637390634	6,553587311	0,364861	-0,471744809	0,677453448	-0,104004587	TCEANC2	N-terminalein-coding	th protein	G00000116205
780	0,006175148	0,00386882	0,132373182	-0,706661	6,785506459	7,49216742	0,060316	-0,256253958	0,159736222	-0,173208844	DDR1	n receptor ein-coding	th protein	G00000204580
148206	0,006076552	0,00388109	0,132427079	-0,77198	7,353680233	8,125660619	0,022971	-1,187943343	0,323193918	-0,165694265	ZNF714	nger proteein-coding	th protein	G00000160352
7374	0,003991056	0,00388989	0,132362562	-1,164224	7,94038771	9,104612136	0,016735	-0,915014197	0,556076742	-0,111208632	UNG	DNA glycoein-coding	th protein	G00000076248
9801	0,005313949	0,00389568	0,132196663	1,1470033	10,09110646	8,944103182	0,03464	0,532659669	0,073395882	0,436478692	MRPL19	al ribosomein-coding	th protein	G00000115364
4751	0,0044099	0,00396879	0,133579422	0,7064218	8,459049122	7,752627289	0,122123	0,292845767	0,006713951	0,43587329	NEK2	related kirein-coding	th protein	G00000117650
9743	0,003671657	0,00398323	0,133702289	-0,881663	7,003809755	7,885472812	0,045737	-0,629230401	0,225441772	0,111376445	ARHGAP32	activatingein-coding	th protein	G00000134909
6631	0,000968532	0,00398628	0,133442893	1,8035898	13,46065345	11,65706361	0,004778	1,525546842	0,785118919	-0,067378735	SNRPC	nucleoprotein-coding	th protein	G00000124562
1832	0,002641777	0,00399007	0,133209795	-1,203085	8,132441528	9,335526448	0,017771	-0,638246499	0,013182887	-1,148462436	DSP	desmoplakiein-coding	th protein	G00000096696
54955	0,006299954	0,00400316	0,13328759	0,6072198	6,908798831	6,301579051	0,040777	0,219220997	0,085988143	0,214380859	C1orf109	l open reacein-coding	th protein	G00000116922
56674	0,000807437	0,00410902	0,136080744	-1,896918	7,068176974	8,965095023	0,025514	-1,140702793	0,404714529	-0,048099053	TMEM9B	main familyein-coding	th protein	G00000175348
25801	0,001338206	0,0041463	0,136222412	-1,246682	7,06048739	8,307169671	0,00567	-0,697220555	0,267174758	-0,140460448	GCA	grancalcin ein-coding	th protein	G00000115271
343521	0,006359168	0,00414942	0,135964308	0,6018794	7,735395696	7,133516306	0,316126	0,463381068	0,380884546	0,086939035	TCTEX1D4	omain conein-coding	th protein	G00000188396
4722	0,001360653	0,00417295	0,136374532	0,5310497	12,0016332	11,47058345	0,105347	0,330397489	0,767246383	-0,045152831	NDUFS3	oxidoreductein-coding	th protein	G00000213619
6453	0,000995256	0,00420416	0,136673413	-0,549119	7,795388223	8,344507247	0,022422	-0,170643101	0,870865073	0,01028727	ITSN1	ntersectin ein-coding	th protein	G00000205726
118429	0,00577444	0,00422825	0,137096784	0,7194858	8,818364937	8,09887916	0,067078	0,423269664	0,044887734	0,271163203	ANTXR2	x toxin receein-coding	th protein	G00000163297
55751	0,00352988	0,00423905	0,137087887	-0,625005	7,290523143	7,91552817	0,196423	-0,381403205	0,443169488	0,079357281	TMEM184C	mbrane proein-coding	th protein	G00000164168
100131174	0,001581775	0,00424208	0,136828708	2,8818099	3,172030098	0,290220209	0,489432	-1,06412289	0,004854766	3,178992975				
56940	0,006651194	0,00429832	0,137567861	0,6252521	7,934446048	7,309193992	0,002762	0,39466128	0,373268104	0,086508816	DUSP22	ficity phospein-coding	th protein	G00000112679
81853	0,002938279	0,00429959	0,137254032	-0,791069	5,366479976	6,15754857	0,05817	-0,436060296	0,581277379	0,076061181	TMEM14B	mbrane proein-coding	th protein	G00000137210
51258	0,00039513	0,00432703	0,137775016	-1,079133	12,54849572	13,62762844	0,189466	-0,169777141	0,919686821	0,008992701	MRPL51	al ribosomein-coding	th protein	G00000111639
55905	0,003854779	0,00433212	0,1375834	0,7343023	10,62974943	9,895447121	0,044422	0,316531255	0,186955453	0,104538889	RNF114	nger proteein-coding	th protein	G00000124226
148641	0,006768045	0,00438326	0,138851473	-0,818531	7,942198242	8,760729716	0,035305	-0,53145384	0,520084414	-0,100799957	SLC35F3	er family 35ein-coding	th protein	G00000183780
11230	0,006589573	0,00438401	0,138520923	0,766856	8,878278231	8,111422277	0,039524	0,680738183	0,445729086	0,110966521	PRAF2	main family ein-coding	th protein	G00000243279
10491	0,005908801	0,0044316	0,138961193	-0,618303	10,04186187	10,66016526	0,149011	-0,36380011	0,306870001	-0,124972151	CRTAP	associateein-coding	th protein	G00000170275
254359	0,003163553	0,00444104	0,138905473	0,6332858	10,58221721	9,94893139	0,016326	0,296158634	0,005067004	0,356781672	ZDHHC24	HHC-type cein-coding	th protein	G00000174165

8508	0,006436712	0,00444599	0,138709849	-0,684631	6,380529134	7,065160513	0,199125	-0,145508242	0,143965216	-0,195199008	NIPSNAP1	omolog 1 (	ein-coding	th protein	G00000184117
10810	0,001825917	0,00444894	0,138453165	0,6015543	7,010005093	6,408450801	0,575489	0,133981756	0,016712324	0,455585125	WASF3	ein family	ein-coding	th protein	G00000132970
401409	0,004019858	0,00445274	0,13822433	2,0096785	3,342488389	1,33280987	0,316675	0,912923308	0,342807189	0,52438373	RAB19	per RAS on	ein-coding	th protein	G00000146955
10534	0,002526188	0,00447537	0,13857949	0,6878125	11,77074073	11,08292819	0,073397	0,135848542	0,090124106	0,116015673	SSSCA1	e/scleroder	ein-coding	th protein	G00000173465
5727	0,006666905	0,00447653	0,138269599	-0,660826	6,180293304	6,841119565	0,06203	-0,331529865	0,071744331	-0,255308488	PTCH1	patched 1	ein-coding	th protein	G00000185920
414777	0,006791255	0,00448504	0,138187845	0,6103017	10,17817717	9,567875431	0,322811	0,154137905	0,071030077	0,249540337	HCG18	oup 18 (nor	n-coding R	long non-c	G00000231074
10007	0,006808648	0,00448762	0,137924182	-0,646862	8,817791812	9,46465384	0,338235	-0,144276144	0,575978711	-0,060836612	GNPDA1	-phosphat	ein-coding	th protein	G00000113552
55752	0,005892674	0,00450045	0,137976216	-0,797726	10,4021514	11,19987749	0,166808	-0,247951283	0,891203893	0,016917526	SEPT11	septin 11	ein-coding	th protein	G00000138758
79140	0,001406798	0,00450441	0,137417204	1,120852	9,100854053	7,980002024	0,025199	0,46495395	0,176705567	0,220737592	CCDC28B	domain cor	ein-coding	th protein	G00000160050
10663	0,002317129	0,00452971	0,137849594	-1,139872	3,628718397	4,768590274	0,446979	-0,376150389	0,165938126	-0,74408393	CXCR6	chemokin	ein-coding	th protein	G00000172215
8407	0,005055465	0,00453692	0,137730575	-1,491765	10,83817858	12,32994405	0,029599	-0,690103056	0,200523327	-0,242243002	TAGLN2	transgelin 2	ein-coding	th protein	G00000158710
1368	0,003877769	0,00454299	0,13757782	0,6237977	5,502805814	4,879008151	0,425435	0,221117786	0,460119758	0,200226722	CPM	oxypeptida	ein-coding	th protein	G00000135678
100507431	0,003386655	0,00454703	0,137364177	-1,585151	3,717793778	5,302944877	0,236959	-0,44137407	0,014986674	-0,703598529					
153577	0,00161015	0,00454714	0,137033315	4,1888364	4,613864594	0,425028182	0,803555	0,212686582	0,002092634	4,105434068					
389084	0,002920585	0,00456306	0,137179438	1,0890516	7,250173153	6,161121523	0,023777	0,966165243	0,358710681	-0,248292574	C2orf82	2 open rea	ein-coding	th protein	G00000182600
79644	0,006996176	0,00459114	0,137356575	-0,697214	8,815417262	9,512631676	0,739212	-0,094988122	0,034648417	-0,416740887	SRD5A3	5 alpha-red	ein-coding	th protein	G00000128039
8702	0,005526951	0,00460981	0,136923197	0,8384068	7,155101875	6,316695107	0,042619	0,659622013	0,333329263	0,149259749	B4GALT4	alactosyltr	ein-coding	th protein	G00000121578
10576	0,006500346	0,00464029	0,137498771	0,5645474	12,32392948	11,75938205	0,035953	0,200406971	0,030616842	0,260611816	CCT2	ontaining T	ein-coding	th protein	G00000166226
55230	0,002808855	0,00469478	0,138450764	-0,893691	7,024577964	7,918268584	0,091622	-0,329126542	0,397501444	-0,149422395	USP40	specific pe	ein-coding	th protein	G00000085982
50848	0,000793165	0,00469824	0,138223645	-1,24871	6,793348514	8,042058305	0,015886	-0,506761069	0,010787129	-0,772504	F11R	11 recepto	ein-coding	th protein	G00000158769
63926	0,006236044	0,00470471	0,138086201	-0,698007	5,745102626	6,443109784	0,148352	-0,67063302	0,714388773	0,040940944	ANKEF1	EF-hand d	ein-coding	th protein	G00000132623
8774	0,004207917	0,0047166	0,138107956	1,0670575	9,874902919	8,807845401	0,059832	0,486256502	0,194604834	0,256830503	NAPG	ment prot	ein-coding	th protein	G00000134265
6291	0,006861791	0,00471999	0,137881156	-1,629591	6,675286723	8,304877541	0,235553	-0,500627673	0,55263404	-0,188504407	SAA4	yloid A4, c	ein-coding	th protein	G00000148965
404636	0,000623183	0,00476941	0,138670603	-1,660496	10,14428931	11,80478569	0,013813	-1,909659465	0,15420164	-0,191214311	FAM45A	ence simila	ein-coding	th protein	G00000119979
54865	0,002232348	0,00476992	0,138360731	0,7385256	10,53406986	9,795544276	0,452885	0,167003306	0,366883305	0,189109808	GPATCH4	domain cor	ein-coding	th protein	G00000160818
84959	0,003734674	0,00478937	0,138600237	1,1086378	8,322485582	7,21384779	0,023595	0,578895908	0,013929505	0,652338684	UBASH3B	l and SH3 c	ein-coding	th protein	G00000154127
653650	0,000437547	0,00481392	0,138662866	0,6521018	8,01989816	7,367796409	0,00168	0,619397057	0,090687754	0,100716405	PDPK2P	ndent prot	seudogen	seudogen	G00000205918
3956	0,001708221	0,00482008	0,138518229	0,5573049	15,65088224	15,09357739	0,512823	0,098861119	0,677930497	0,029118574	LGALS1	galectin 1	ein-coding	th protein	G00000100097
677844	0,007435284	0,00485456	0,138864707	0,54971	12,74768647	12,19797647	0,406168	0,061206524	0,011576501	0,598469245	SNORA78	lar RNA, H	n-coding R	small nucl	G00000273587
25983	0,005318715	0,0048758	0,139150814	0,5641423	10,02968657	9,46554426	0,159395	0,211002086	0,25119798	0,16906627	NGDN	neuroguidi	ein-coding	th protein	G00000129460
9839	0,004350224	0,00490326	0,139292682	0,7760401	6,52635307	5,75031294	0,031621	0,447410373	0,215901787	0,184310753	ZEB2	box binding	ein-coding	th protein	G00000169554
100528032	0,005134035	0,00490932	0,13914551	-1,124463	3,965299586	5,089763049	0,478121	-0,256389056	0,131564702	-0,682520824	LRC4-KLRK	KLRK1 read	other	eadthroug	G00000255819
4154	0,004930529	0,00492813	0,139359961	0,8882328	9,66616328	8,777930472	0,152722	0,202008179	0,284819112	0,17212746	MBNL1	like splicin	ein-coding	th protein	G00000152601
64393	0,006830443	0,00492952	0,139081767	0,7156247	6,358189277	5,642564598	0,018736	0,366528067	0,033105726	0,392859197	ZMAT3	ger matrin	ein-coding	th protein	G00000172667
9686	0,00759616	0,00495908	0,139281057	-0,566174	7,412796245	7,97897042	0,712526	-0,083911262	0,019505685	-0,361491018	VGLL4	ike family r	ein-coding	th protein	G00000144560
56951	0,003090161	0,00499394	0,139312473	-1,021016	7,7883497	8,809365414	0,001705	-0,84255302	0,498834351	0,110700887	C5orf15	5 open rea	ein-coding	th protein	G00000113583
51280	0,001142767	0,005039	0,139939142	-1,394748	8,660078281	10,05482676	0,010962	-0,549832498	0,550526426	-0,098528795	GOLM1	embrane p	ein-coding	th protein	G00000135052
1808	0,003699337	0,00505133	0,139967681	-0,874538	8,539023051	9,41356059	0,160729	-0,219931779	0,026655428	-0,482630039	DPYSL2	pyrimidina	ein-coding	th protein	G00000092964
8611	0,001148729	0,00511022	0,140033274	0,8352857	10,00022661	9,164940923	0,013002	0,609016435	0,23473522	-0,148273683	PLPP1	lipid phosp	ein-coding	th protein	G00000067113
114876	0,000848241	0,00514493	0,140673037	-0,5949	7,593904567	8,18880428	0,108053	-0,269175326	0,001577669	-0,152516514	OSBPL1A	inding pro	ein-coding	th protein	G00000141447
9804	0,006767615	0,00514599	0,140392181	-0,572725	9,023505675	9,596230227	0,000379	-0,421918432	0,172396039	-0,159699195	TOMM20	r mitochon	ein-coding	th protein	G00000173726
9572	0,007440647	0,00514912	0,140168797	0,6113144	7,677021309	7,065706882	0,017045	0,555197571	0,370293323	0,082811573	NR1D1	ubfamily 1	ein-coding	th protein	G00000126368
10950	0,006482932	0,00518445	0,140821154	0,8788626	9,336660607	8,45779802	0,005245	0,666940228	0,111691422	0,299144643	BTG3	proliferatio	ein-coding	th protein	G00000154640
2768	0,007806595	0,00518941	0,140647225	0,557275	9,347143991	8,789868979	0,214575	0,274311603	0,815459917	0,023496287	GNA12	in subunit a	ein-coding	th protein	G00000146535
5127	0,003701116	0,00519918	0,14060434	-0,56955	9,536170076	10,10571994	0,010045	-0,271647077	0,054006227	0,241948017	CDK16	ependent k	ein-coding	th protein	G00000102225
115704	0,006317271	0,00521166	0,140634809	0,5650461	7,098115824	6,533069733	0,026697	0,205372431	0,031773128	0,236090933	EVI5L	al integrati	ein-coding	th protein	G00000142459
10093	0,007613349	0,00522748	0,140145788	0,7164635	8,131632123	7,415168636	0,022134	0,25129354	0,116939204	0,210722422	ARPC4	tein 2/3 c	ein-coding	th protein	G00000241553
10329	0,005876466	0,00523627	0,140078277	-0,766853	7,758994618	8,525847658	0,003293	-0,394965425	0,023303858	0,35244251	TMEM5	embrane p	ein-coding	th protein	G00000118600
344657	0,007373246	0,00525405	0,140250921	2,5058926	3,055431894	0,549539285	0,941234	0,115068532	0,291843257	1,837007078	LRRIQ4	ats and IQ r	ein-coding	th protein	G00000188306
92292	0,007813484	0,00527165	0,14041808	-1,277287	3,693082673	4,970369671	0,41416	-0,614189794	0,263142479	-0,334290335	GLYATL1	acyltransfe	ein-coding	th protein	G00000166840
1948	0,002885086	0,00527498	0,140205377	0,7234553	8,329308299	7,605853009	0,721753	-0,037151721	0,200028876	-0,158086432	EFNB2	ephrin B2	ein-coding	th protein	G00000125266
8698	0,001606695	0,00531076	0,140253336	-0,770965	6,07931693	6,850281718	0,16339	-0,33625172	0,025058537	-0,381888351	S1PR4	1-phospha	ein-coding	th protein	G00000125910
205	0,002279138	0,00533064	0,140180631	-0,672631	9,146781986	9,819412549	0,446547	-0,137445393	0,262841278	-0,207810818	AK4	nylate kina	ein-coding	th protein	G00000162433
9673	0,00800954	0,00534354	0,140222101	0,533053	7,543588376	7,010535382	0,014599	0,362403312	0,040910614	0,271551574	SLC25A44	r family 25	ein-coding	th protein	G00000160785
94081	0,006688728	0,00536012	0,140063675	0,5069162	10,22998606	9,723069847	0,10641	0,229322496	0,0305038	0,338556564	SFXN1	ideroflexin	ein-coding	th protein	G00000164466
10047	0,003623555	0,00537063	0,139748701	1,1505844	4,248130289	3,097545855	0,113191	0,841805896	0,544559548	0,164777353	CST8	cystatin 8	ein-coding	th protein	G00000125815
170392	0,006021525	0,00544136	0,140996856	0,5185166	5,095141182	4,576624607	0,88182	0,047178395	0,427237436	0,272907374	OIT3	n induced	ein-coding	th protein	G00000138315
390667	0,004144726	0,0054724	0,141210388	0,7513402	5,348853774	4,597513564	0,015563	0,572246816	0,687255727	0,060099214	PTX4	pentraxin 4	ein-coding	th protein	G00000251692
100130342	0,002410924	0,00549329	0,141161249	0,6105432	4,671113933	4,060570697	0,986316	0,006904004	0,181386791	0,402813613	SCGB2B3P	ly 2B mem	seudogen	seudogen	G00000269811

3251	0,001066102	0,00550614	0,141198342	-0,914354	9,457793629	10,37214775	0,086278	-0,202416734	0,930035486	-0,006982087	HPRT1	hosphoribc	ein-coding	th protein	G00000165704
100170229	0,008254114	0,00552052	0,140983284	0,6589281	8,22239998	7,563471901	0,334962	0,14345484	0,487710268	0,086685332	SRRM5	iine repetit	ein-coding	th protein	G00000226763
25850	0,004628251	0,00553322	0,141017085	-0,624521	6,212993733	6,837515009	0,021625	-0,418715445	0,41998148	-0,082028017	ZNF345	nger prote	ein-coding	th protein	G00000251247
100130713	0,008358682	0,00556745	0,141597981	0,6955523	6,790312675	6,09476042	0,020145	0,663731198	0,732443141	-0,043903954					
6364	0,005482446	0,00560952	0,141794386	1,1704226	5,854365317	4,683942751	0,586326	0,185079483	0,23360878	0,386727958	CCL20	chemokine	ein-coding	th protein	G00000115009
3017	0,004615595	0,00565982	0,142774654	-1,67897	9,964108771	11,64307876	0,396982	-0,405218076	0,008246209	-1,41200835	HIST1H2BD	r 1 H2B far	ein-coding	th protein	G00000158373
83876	0,004792719	0,00568103	0,142728113	0,660654	5,068158574	4,407504527	0,135412	0,412147072	0,775901568	0,062261267	MRO	maestro	ein-coding	th protein	G00000134042
23075	0,000750191	0,00568551	0,142551515	-1,415029	7,507609912	8,922639145	0,001379	-1,255891143	0,866942949	-0,0173781	SWAP70	-cell comp	ein-coding	th protein	G00000133789
6809	0,005463397	0,00569275	0,14244484	-0,553736	9,42373724	9,977473679	0,053758	-0,304533051	0,068315092	-0,149336197	STX3	syntaxin 3	ein-coding	th protein	G00000166900
100129405	0,007107237	0,00569672	0,141970436	0,5722862	9,160985097	8,588698937	0,019355	0,315938845	0,015985746	0,318006174	MSTO2P	member 2	seudogen	seudogen	G00000203761
89765	0,008386574	0,00570138	0,141801326	0,6616269	4,813917887	4,152290949	0,150448	0,303410141	0,191184165	0,214769515	RSPH1	oke head 1	ein-coding	th protein	G00000160188
256987	0,001049577	0,00573053	0,142240762	-0,99965	6,279219674	7,278870124	0,002012	-0,686417016	0,531767773	0,077314422	SERINC5	e incorpora	ein-coding	th protein	G00000164300
152302	0,006649837	0,0057386	0,142156116	1,6591657	9,290151848	7,630986173	0,002332	1,117532542	0,201349009	0,306056475	CIDECP	)FFA-like e	seudogen	seudogen	G00000186162
740	0,007089868	0,00574358	0,141995442	0,6380823	8,54612725	7,908044987	0,989828	-0,002718444	0,14588202	0,197376461	MRPL49	al ribosoma	ein-coding	th protein	G00000149792
6464	0,008598153	0,00576622	0,142271342	0,6865836	9,366772159	8,680188601	0,009523	0,380936399	0,131698057	0,209777103	SHC1	daptor pro	ein-coding	th protein	G00000160691
128486	0,005526199	0,00581095	0,142523106	0,6001738	8,320109594	7,719935797	0,067857	0,221646178	0,563715444	0,067761409	FITM2	ng transme	ein-coding	th protein	G00000197296
84447	0,003841302	0,00583738	0,142888495	0,6461364	9,480778939	8,834642524	0,005143	0,569263694	0,166838474	0,189397635	SYVN1	synoviolin :	ein-coding	th protein	G00000162298
1073	0,006239627	0,0058418	0,142714541	0,6824006	9,201702434	8,519301863	0,154892	0,471806472	0,619437521	-0,060823475	CFL2	cofilin 2	ein-coding	th protein	G00000165410
138199	0,00869161	0,00584785	0,14258129	-0,687791	7,158073559	7,845864689	0,250109	-0,611570577	0,019350858	-0,643408618	CARNMT1	N-methyltr	ein-coding	th protein	G00000156017
100130111	0,008678634	0,00586681	0,142762421	-0,581009	7,251006543	7,832015174	0,550074	-0,107516557	0,087729347	-0,292105428					
3115	0,001921685	0,00593784	0,143925388	-0,993588	10,40223089	11,39581923	0,11555	-0,232145519	0,009859543	-0,661568616	HLA-DPB1	ility compl	ein-coding	th protein	G00000223865
1054	0,005610151	0,00597695	0,144590258	0,638619	9,725023683	9,086404707	0,155876	0,275453226	0,0504586	0,365116565	CEBPG	er binding	ein-coding	th protein	G00000153879
6941	0,001137392	0,00602116	0,144811351	-0,738476	6,898479532	7,636955801	0,007285	-0,711505075	0,280186272	-0,142062256	TCF19	cription fac	ein-coding	th protein	G00000137310
283692	0,008497189	0,00603132	0,144774714	4,4411478	4,976439013	0,535291189	0,433936	1,371764887	0,608821426	0,875155558	CPEB1-AS1	antisense	n-coding R	long non-c	G00000259462
374654	0,003750686	0,00604377	0,144792866	0,7181609	5,587157136	4,868996241	0,612129	0,082867094	0,014910999	0,639141356	KIF7	family me	ein-coding	th protein	G00000166813
8220	0,002154193	0,00605298	0,144733492	0,8622033	7,304435618	6,442232341	0,042914	0,222707136	0,086979143	0,302709972	DGCR14	ome critica	ein-coding	th protein	G00000100056
27090	0,008679049	0,0060654	0,144751047	0,7156537	9,195756244	8,480102507	0,008984	0,280617816	0,089707523	0,280558192	T6GALNAC	inide alph	ein-coding	th protein	G00000136840
22949	0,007343174	0,00607533	0,144709172	-0,66212	10,24856896	10,91068895	0,025984	-0,454304907	0,004625852	-0,73034244	PTGR1	landin red	ein-coding	th protein	G00000106853
729830	0,005559754	0,00610234	0,144795684	-0,616875	5,674614442	6,291489626	0,023244	-0,366535561	0,263672858	-0,106072076	FAM160A1	nce similari	ein-coding	th protein	G00000164142
11217	0,009031077	0,00611171	0,144740608	0,6838494	8,435974187	7,752124814	0,063605	0,293604203	0,231799223	0,153143514	AKAP2	anchoring	ein-coding	th protein	G00000241978
55300	0,003390102	0,0061372	0,144515505	-0,803557	7,723615593	8,52717215	0,056916	-0,587664158	0,157052401	0,256859525	PI4K2B	ositol 4-kin	ein-coding	th protein	G00000038210
55272	0,004390678	0,00614829	0,144501759	-0,853748	11,16348438	12,01723225	0,010627	-0,720994196	0,34764061	0,136037437	IMP3	nucleolar ri	ein-coding	th protein	G00000177971
5395	0,005966191	0,0061539	0,144086986	-0,609381	6,488169448	7,097550099	0,290806	-0,384109555	0,633966382	0,060391576	PMS2	match rep	ein-coding	th protein	G00000122512
153527	0,007764632	0,00616283	0,144023777	-0,544572	9,529078061	10,07365047	0,169837	-0,345421878	0,140528625	-0,382891044	ZMAT2	ger matrin	ein-coding	th protein	G00000146007
93082	0,004553447	0,00617683	0,144078981	-0,721134	6,373881474	7,095014999	0,973242	-0,005952502	0,007556583	-0,348421911	NEURL3	ubiquitin p	ein-coding	th protein	G00000163121
2896	0,003291176	0,00621871	0,144240942	-0,62569	10,56365407	11,18934396	0,194829	-0,160399929	0,006059288	-0,41287614	GRN	granulin	ein-coding	th protein	G00000030582
154141	0,00824172	0,00626594	0,144794091	-1,858534	3,595199161	5,453732857	0,065028	-0,917850407	0,393661685	0,228472029	MBOAT1	yltransfera	ein-coding	th protein	G00000172197
5218	0,006191117	0,00631297	0,145338752	0,5496307	6,374941831	5,825311161	0,26653	0,157185107	0,01275405	0,482524513	CDK14	ependent k	ein-coding	th protein	G00000058091
8898	0,00929392	0,00632599	0,145368183	0,6299512	9,389311851	8,759360675	0,27636	0,14978925	0,123741321	0,216188943	MTMR2	arin relate	ein-coding	th protein	G00000087053
390321	0,006443088	0,00635205	0,145427395	1,9289341	4,949582852	3,020648794	0,091954	1,369598504	0,370351194	0,525836506	OR6C1	amily 6 sub	ein-coding	th protein	G00000205330
9517	0,0026409	0,00637018	0,145305244	-0,883124	7,102087386	7,985211201	0,002049	-0,404320087	0,588099642	-0,068500593	SPTLC2	ferase long	ein-coding	th protein	G00000100596
136227	0,003792415	0,00638396	0,145085285	0,5573085	5,949457243	5,392148758	0,127233	0,387240073	0,837806217	-0,037177422	COL26A1	pe XXVI alp	ein-coding	th protein	G00000160963
5251	0,007967373	0,00639627	0,144833639	1,5054767	3,919049982	2,413573275	0,21366	0,311532898	0,002680378	1,471132635	PHEX	endopeptic	ein-coding	th protein	G00000102174
100132163	0,008367082	0,0063976	0,144599403	-1,609499	3,005631244	4,615130183	0,289966	-1,173632484	0,772176712	-0,247999918	PHKA2-AS1	antisense	n-coding R	long non-c	G00000237836
3052	0,000946809	0,00641528	0,144734792	0,8496356	10,06903313	9,219397483	0,130684	0,299868719	0,002220302	0,365341574	HCCS	ochrome c	ein-coding	th protein	G00000004961
26658	0,009243481	0,00641983	0,144574209	-0,704836	5,568383688	6,273219807	0,278806	-0,464238037	0,784332207	-0,055479537	OR7C2	amily 7 sub	ein-coding	th protein	G00000127529
51147	0,007132659	0,00643334	0,144615444	-0,606894	6,980309207	7,587202912	0,166188	-0,206837968	0,014609846	-0,381799195	ING4	rowth fam	ein-coding	th protein	G00000111653
221	0,001097003	0,00646242	0,144743625	-0,708266	6,734722256	7,442988308	0,73308	-0,058819147	0,015813814	-0,34420378	ALDH3B1	ogenase 3 f	ein-coding	th protein	G00000006534
2869	0,009433371	0,0065052	0,144915772	0,6457434	7,381755708	6,736012284	0,005794	0,304723822	0,061970845	0,305481525	GRK5	upled rece	ein-coding	th protein	G00000198873
3035	0,002065287	0,00650677	0,144690476	0,543897	10,45334822	9,909451257	0,148408	0,118165051	0,073667516	0,184352992	HARS	l-tRNA synt	ein-coding	th protein	G00000170445
403150	0,009184298	0,00654145	0,145200903	0,7763876	4,466152541	3,689764922	0,474907	0,296648842	0,508674215	-0,234933517					
390429	0,000663056	0,00657378	0,145397519	-2,652345	0	2,652344719	0,42265	-1,822327021	0,694302132	-0,830017698	OR4N2	amily 4 sub	ein-coding	th protein	G00000176294
140606	0,007263317	0,00659836	0,145421687	1,1490491	9,303493914	8,154444776	0,004562	0,521972411	0,059258452	0,391825913	SELENOM	selenoprotein M(SELENOM)			
5594	0,008423308	0,00664531	0,145421414	0,6025588	9,783462879	9,18090411	0,002904	0,496811009	0,406122826	0,094888783	MAPK1	ivated prot	ein-coding	th protein	G00000100030
2805	0,004335437	0,00666952	0,14543742	0,8028496	9,883023361	9,080173732	0,001814	0,421359101	0,03250208	0,482654172	GOT1	loacetic tra	ein-coding	th protein	G00000120053
29937	0,001839427	0,00670089	0,145608595	-0,961372	11,21185395	12,17322642	0,002642	-0,543627402	0,10527895	-0,141816806	NENF	neurotrop	ein-coding	th protein	G00000117691
91801	0,009639679	0,00673918	0,146184236	0,7991086	7,081659474	6,282550829	0,775804	0,0843382	0,122784323	0,356587991	ALKBH8	3, tRNA me	ein-coding	th protein	G00000137760
388948	0,007476352	0,00677856	0,14678139	0,8220594	5,617390962	4,795331543	0,100603	0,572910417	0,721275471	0,089640697	LINC01118	on-protein	n-coding R	long non-c	G00000222005
83478	0,009462843	0,00682068	0,147435713	-0,931646	5,268784534	6,200430418	0,135315	-0,604853713	0,974076478	-0,004988394	ARHGAP24	e activating	ein-coding	th protein	G00000138639

7633	0,008468524	0,00682787	0,147334069	0,7102148	6,779741681	6,069526927	0,428394	0,101408312	0,26169051	0,160129993	ZNF79	inger prot	ein-coding	th protein	G00000196152
80183	0,009371624	0,00683977	0,147078296	0,7234303	4,533914155	3,810483874	0,088662	0,413120133	0,146738925	0,236360811	RUBCNL	containing	ein-coding	th protein	G00000102445
84830	0,009793684	0,00684208	0,146873105	-0,606451	5,507091239	6,113541774	0,284072	0,318178848	0,145952783	-0,475878304	ADTRP	dent TFPI r	ein-coding	th protein	G00000111863
84879	0,005700431	0,00686539	0,147118499	0,9120543	7,511558688	6,599504355	0,023783	0,48481298	0,009281131	0,466578823	MFSD2A	erfamily d	ein-coding	th protein	G00000168389
6450	0,008837552	0,00690695	0,147753401	0,7553897	7,648159829	6,892770139	0,0165	0,393507929	0,238363302	-0,182046624	SH3BGR	ding glutan	ein-coding	th protein	G00000185437
90637	0,008493424	0,00694366	0,148282492	0,8717221	10,29489661	9,423174471	0,0404	0,575717143	0,102118645	0,276027539	ZFAND2A	N1-type c	ein-coding	th protein	G00000178381
92558	0,006042921	0,00695044	0,147917253	0,5557157	5,527428594	4,971712893	0,241366	0,49251825	0,934353315	-0,027398354	BICDL1	y like carg	ein-coding	th protein	G00000135127
151987	0,003923002	0,00701031	0,147920559	-0,835289	8,94764642	9,782935806	0,175827	-0,438944466	0,929410346	-0,015046392	PPP4R2	tase 4 reg	ein-coding	th protein	G00000163605
55033	0,009977153	0,00701752	0,147820909	0,8622836	9,368864198	8,506580642	0,079101	0,552860989	0,455307877	0,113210252	FKBP14	binding pr	ein-coding	th protein	G00000106080
23259	0,005897763	0,0070206	0,147634695	-0,723375	7,107827413	7,831202396	0,153962	-0,401312833	0,02971965	-0,306892445	DDHD2	omain con	ein-coding	th protein	G00000085788
339221	0,008114471	0,00706717	0,148111183	0,6831007	5,941725255	5,258624567	0,151684	-0,284211369	0,159316361	0,242490707	ENPP7	nosphatase	ein-coding	th protein	G00000182156
10901	0,005200757	0,00708526	0,147989373	-0,886342	8,207140483	9,093482974	0,351767	0,179228107	0,143284252	-0,339066943	DHRS4	genase/rec	ein-coding	th protein	G00000157326
114294	0,006483582	0,00708902	0,14781882	-0,579976	8,507857949	9,087833686	0,077815	-0,324976732	0,133351682	-0,263047092	LACTB	ctamase be	ein-coding	th protein	G00000103642
5476	0,00567457	0,00710828	0,147971183	-0,551391	10,69570751	11,24709826	0,01724	-0,260321158	0,20018843	-0,146079405	CTSA	cathepsin A	ein-coding	th protein	G00000064601
900	0,005364736	0,00716557	0,148913558	-0,581704	8,114902526	8,696606654	0,088496	-0,627469564	0,939787858	0,008281915	CCNG1	cyclin G1	ein-coding	th protein	G00000113328
728730	0,008961995	0,00719152	0,148953019	-1,281429	5,486281325	6,767710672	0,201646	-0,617592755	0,091184607	-0,531568437					
2068	0,009071043	0,00720605	0,149004751	0,507048	8,998706939	8,491658963	0,100786	0,316048914	0,442011953	0,101693521	ERCC2	FIIH core c	ein-coding	th protein	G00000104884
219981	0,006707649	0,00720931	0,1488238	0,948232	5,191573337	4,243341331	0,319211	0,309964023	0,27824539	0,292712301	OR5A2	amily 5 sub	ein-coding	th protein	G00000172324
440275	0,010168662	0,00721164	0,14862409	-0,715297	8,043569754	8,758867188	0,037178	-0,312181603	0,27506118	0,140701775	EIF2AK4	initiation f	ein-coding	th protein	G00000128829
360132	0,005306743	0,00721273	0,148153629	-0,86637	7,39590795	8,262277674	0,043696	-0,549233075	0,033233005	-0,276674266	FKBP9P1	; protein 9	pseudogene	pseudogene	G00000176826
285282	0,010190579	0,00724163	0,148255408	-0,592065	7,118538712	7,710603937	0,329099	-0,23125257	0,279625004	-0,173495853	RABL3	RAS oncog	ein-coding	th protein	G00000144840
5962	0,008811256	0,00725429	0,148269609	0,6517482	10,11594533	9,464197152	0,006914	0,5607128	0,816340899	0,031546771	RDX	radixin	ein-coding	th protein	G00000137710
554249	0,006216419	0,00729116	0,148289069	0,6202575	9,083133868	8,462876404	0,03747	0,471530432	0,081625907	0,257932801					
152189	0,001271701	0,00732223	0,148676831	-1,123935	7,614288982	8,738223677	0,040947	-0,522796995	0,352815301	-0,058535653	CMTM8	smembran	ein-coding	th protein	G00000170293
53838	0,007994562	0,00732265	0,148441999	0,6113624	9,211709807	8,600347456	0,396198	0,189457795	0,062088283	-0,524623882	C11orf24	11 open re	ein-coding	th protein	G00000171067
140849	0,001464393	0,00732392	0,147983244	0,5664628	8,45626991	7,889807135	0,272123	0,694617983	0,323157236	-0,604759094	INC00266-	on-protein	n-coding R	long non-c	G00000149656
2067	0,008375525	0,007355	0,148369275	0,7022341	11,16276819	10,46053406	0,21191	0,131897207	0,412787357	0,089631174	ERCC1	endonuclea	ein-coding	th protein	G00000012061
6596	0,002120243	0,00735753	0,148178932	-1,224764	7,03127651	8,256040102	0,066746	-0,948430043	0,961032183	-0,007643903	HLTF	e transcrip	ein-coding	th protein	G00000071794
55856	0,010610828	0,00736038	0,147995839	-0,535255	10,77055334	11,3058087	0,019413	-0,214027471	0,198217297	-0,141167295	ACOT13	A thioeste	ein-coding	th protein	G00000112304
338620	0,003044293	0,00736399	0,147828274	0,7514781	8,030180964	7,278702908	0,102735	0,299912848	0,145312048	0,245145452					
55062	0,010633289	0,00737872	0,147884388	0,8768044	9,280736616	8,403932212	0,001449	0,612451954	0,026717527	0,602163778	WIPI1	phosphoin	ein-coding	th protein	G00000070540
794	0,004443952	0,00740145	0,147623333	-0,562391	11,17873401	11,74112494	0,483982	-0,128683775	0,14237627	-0,15715078	CALB2	calbindin 2	ein-coding	th protein	G00000172137
6595	0,005630697	0,00747142	0,148540388	-0,59592	6,868644345	7,464563924	0,046293	-0,395758671	0,735766599	0,039143792	SMARCA2	pendent re	ein-coding	th protein	G00000080503
51776	0,007236768	0,00748551	0,148344437	0,5436764	8,49293915	7,94926272	0,042228	0,214898074	0,381934243	0,096541238	ZAK	sterile alpha motif and leucine zipper containing kinase AZK(ZAK)			
51441	0,002192585	0,00749216	0,148238992	-1,283265	8,94205328	10,22531823	0,004568	-0,730860792	0,048160395	-0,523891192	YTHDF2	nosine RN	ein-coding	th protein	G00000198492
8776	0,007509955	0,00755087	0,148688034	-0,514441	7,646337525	8,160778098	0,009694	-0,246568254	0,888686537	-0,012964708	MTMR1	arin relate	ein-coding	th protein	G00000063601
55246	0,001573139	0,00763674	0,148958054	-1,125218	8,713620163	9,838837727	0,038198	-0,495653373	0,247770168	-0,222894666	CCDC25	domain co	ein-coding	th protein	G00000147419
23212	0,009270659	0,00766217	0,148984922	0,808666	11,52530366	10,71663767	0,175126	0,226839279	0,407224823	0,146318222	RRS1	enesis reg	ein-coding	th protein	G00000179041
374286	0,010505666	0,00769101	0,148612394	-0,641922	8,401801524	9,043723299	0,0093	-0,410707726	0,013873914	-0,459244705	CDRT1	cated regio	ein-coding	th protein	G00000241322
141	0,0104027	0,00772718	0,149078684	-1,421275	4,043569378	5,464844269	0,11339	-0,758981002	0,438937872	-0,304722777	ADPRH	sylarginine	ein-coding	th protein	G00000144843
51114	0,009769303	0,00772762	0,148855313	-0,590981	7,608658973	8,199639634	0,009925	-0,393043558	0,510020753	0,07669544	ZDHHC9	DHHC-type	ein-coding	th protein	G00000188706
51569	0,006565953	0,00777329	0,149270844	0,5114839	9,648969495	9,137485615	0,063032	0,27472269	0,306440134	0,061845345	UFM1	tin fold mo	ein-coding	th protein	G00000120686
116154	0,00573341	0,00780141	0,149347645	0,5617249	4,932741082	4,371016188	0,121759	0,69908849	0,418808248	-0,291131297	PHACTR3	e and actin	ein-coding	th protein	G00000087495
131870	0,008955224	0,00781332	0,149114659	0,5415745	8,484859164	7,943284641	0,040093	0,438921061	0,888458034	0,014543153	NUDT16	ix hydrolas	ein-coding	th protein	G00000198585
6728	0,005110999	0,00783721	0,149111207	0,541109	11,32591295	10,78480399	0,521044	0,126568461	0,077746005	0,224974385	SRP19	cognition p	ein-coding	th protein	G00000153037
84740	0,010590174	0,00784848	0,149096538	0,5317144	8,179052058	7,647337612	0,630152	0,053083998	0,019389121	0,39319911	AFAP1-AS1	antisense	n-coding R	long non-c	G00000272620
5908	0,003627258	0,00789476	0,149517116	0,9948661	10,22950331	9,234637255	0,011113	0,455640036	0,070171446	0,398503811	RAP1B	er of RAS o	ein-coding	th protein	G00000127314
291	0,009776144	0,00790129	0,149184603	0,6386712	12,54785188	11,90918071	0,346113	0,204129479	0,097787846	0,300254741	SLC25A4	er family 2	ein-coding	th protein	G00000151729
23102	0,01109163	0,00793411	0,149576237	-0,504509	6,136354852	6,640863971	0,193615	-0,282451805	0,40790894	-0,114621695	TBC1D2B	ain family r	ein-coding	th protein	G00000167202
9945	0,007748649	0,0080009	0,15014976	0,9502417	7,897689539	6,947447843	0,155447	0,301721719	0,014891371	0,580898916	GFPT2	e-6-phosph	ein-coding	th protein	G00000131459
2786	0,008693566	0,00804339	0,150491141	-0,673965	6,07953735	6,75350255	0,559452	-0,147714675	0,209383067	-0,128902872	GNG4	n subunit g	ein-coding	th protein	G00000168243
401491	0,001024984	0,00808594	0,150831502	0,784615	6,899812716	6,11519773	0,055171	0,49888808	0,06616256	0,412255744	VLDLR-AS1	antisense	n-coding R	long non-c	G00000236404
55166	0,011462199	0,00810039	0,150873859	-0,530245	7,417186015	7,947431375	0,332499	-0,382027356	0,571581528	-0,081516274	CENPQ	omere prot	ein-coding	th protein	G00000031691
1021	0,009548159	0,00813081	0,150986388	-0,578251	8,93227264	9,510523176	0,033921	-0,385998359	0,263126261	0,139051464	CDK6	ependent k	ein-coding	th protein	G00000105810
100133190	0,00428594	0,00820192	0,151398886	0,6006858	6,550683445	5,949997651	0,828321	-0,064223889	0,046150832	0,297288513	ADIRF-AS1	antisense	n-coding R	long non-c	G00000272734
51026	0,00672381	0,00821479	0,151185823	0,6467256	9,628383802	8,981658223	0,088736	0,268826646	0,027226954	0,459467659	GOLT1B	gi transpor	ein-coding	th protein	G00000111711
63932	0,004187131	0,00822571	0,151162198	1,284824	8,405667701	7,12084367	0,070505	0,659846807	0,228794323	0,2902718	CXorf56	X open rea	ein-coding	th protein	G00000018610
2012	0,009947297	0,00826511	0,151436856	-0,879361	9,223099179	10,10246009	0,162741	-0,4523104	0,21008647	-0,243328061	EMP1	membrane	ein-coding	th protein	G00000134531
221264	0,009563528	0,00828763	0,151178509	-1,108745	3,622097152	4,73084226	0,163308	-0,561835157	0,856029606	-0,04904188	AK9	nylate kina	ein-coding	th protein	G00000155085

729220	0,004678147	0,00834084	0,151702349	0,744995	5,335368635	4,590373586	0,004228	0,253457532	0,023935265	0,587482082					
3337	0,001181909	0,00834188	0,151498755	0,6903516	10,63490131	9,944549693	0,1276	0,327406091	0,226181611	0,232942107	DNAJB1	tein family	ein-coding	th protein	G00000132002
84450	0,008605607	0,0083494	0,151413331	-0,712783	6,588250037	7,301033203	0,11165	-0,806682906	0,343500031	-0,149496935	ZNF512	nger prote	ein-coding	th protein	G00000243943
3484	0,001549225	0,00834952	0,151194207	2,025348	10,14225907	8,116911051	0,00342	1,05760526	0,287181074	0,307936691	IGFBP1	th factor b	ein-coding	th protein	G00000146678
1723	0,004042607	0,00835743	0,15111648	0,657251	7,120501613	6,463250647	0,049895	-0,293245047	0,024416614	0,416582526	DHODH	dehydrog	ein-coding	th protein	G00000102967
93273	0,009865221	0,00839578	0,151147886	1,0589967	4,968606653	3,909609973	0,474504	0,343815719	0,184203018	0,542597934	LEMD1	main cont	ein-coding	th protein	G00000186007
348094	0,011632711	0,00841342	0,150807973	0,8265209	5,384987464	4,558466575	0,311003	0,210278157	0,108584106	0,374574067	ANKDD1A	death dor	ein-coding	th protein	G00000166839
285987	0,011801786	0,00842824	0,150420474	-2,188097	0,324904283	2,513001471	0,885327	-0,138587909	0,412777068	-0,764766757	DLX6-AS1	antisense	In-coding R	long non-c	G00000231764
112858	0,009304981	0,00850256	0,151310899	0,8028127	6,647773067	5,844960385	0,975839	0,01155649	0,109756142	0,421619151	TP53RK	regulating	ein-coding	th protein	G00000172315
387720	0,01194929	0,00854891	0,151050662	0,9352746	4,764856695	3,829582048	0,19783	0,67359322	0,361805509	0,16664485					
154807	0,011570409	0,00856055	0,150825906	0,5832255	9,940974418	9,357748947	0,013197	0,325829637	0,008953045	0,586709946	VKORC1L1	uctase co	ein-coding	th protein	G00000196715
389493	0,008908679	0,0085664	0,150714567	0,6113714	8,052691287	7,441319886	0,804463	-0,111256711	0,860657164	0,040082675	NUPR2	2, transcrip	ein-coding	th protein	G00000185290
150483	0,008812141	0,00857734	0,150479615	0,5398664	6,61982676	6,079960324	0,059546	0,458341543	0,649377	-0,060452299	TEKT4	tektin 4	ein-coding	th protein	G00000163060
51013	0,009160276	0,00860259	0,150709087	0,703689	10,97984456	10,27615552	0,118	0,275750787	0,656243259	-0,06490695	EXOSC1	me compo	ein-coding	th protein	G00000171311
445815	0,01083679	0,00862634	0,150698964	0,500674	6,844077191	6,343403167	0,043375	0,690217688	0,342310054	-0,119165243	ALM2-AKA	AKAP2 reac	other	eadthroug	G00000157654
6167	0,006850331	0,00863296	0,15060219	-0,531621	10,24638654	10,77800789	0,026306	-0,21731447	0,003060362	-0,401857114	RPL37	omal prote	ein-coding	th protein	G00000145592
1002	0,007302277	0,00864046	0,150521017	1,036826	7,629135557	6,592309602	0,050015	0,611262235	0,115898291	0,390985976	CDH4	cadherin 4	ein-coding	th protein	G00000179242
55296	0,00179054	0,0086457	0,150189735	-0,619196	5,76437358	6,38356989	0,110135	-0,428832255	0,130974879	-0,398228634	TBC1D19	ain family r	ein-coding	th protein	G00000109680
348303	0,008987895	0,00865036	0,150060254	2,7026123	3,270928847	0,56831658	0,763367	0,28685169	0,073484982	2,515337073	SELENOV	selenoprotein V(SELENOV)			
9263	0,011110242	0,00865643	0,149955545	0,6249478	8,88678182	8,261834053	0,0139	0,181469752	0,182107617	0,155920736	STK17A	reonine ki	ein-coding	th protein	G00000164543
6372	0,001384361	0,00866319	0,149863037	0,7323497	4,192390525	3,460040867	0,386161	0,40946501	0,559772801	0,254772907	CXCL6	if chemoki	ein-coding	th protein	G00000124875
51141	0,006090857	0,00867535	0,149864034	-0,509144	9,828276569	10,33742042	0,941665	0,007128283	0,014000107	-0,294935994	INSIG2	n induced g	ein-coding	th protein	G00000125629
642311	0,002541382	0,00870553	0,150175927	0,5168915	11,15648354	10,63959202	0,044431	0,302321385	0,001733056	0,213317988	BMS1P15	ogenesis fa	seudogeno	seudogen	G00000258780
56919	0,004558334	0,00870665	0,149986388	0,686201	8,324628083	7,638427075	0,863148	0,0205823	0,099277436	0,278195484	DHX33	-box helica	ein-coding	th protein	G00000005100
1193	0,011711373	0,00871601	0,149731074	-0,915146	4,15609838	5,071244499	0,286656	-0,604394046	0,199793936	-0,3966902	CLIC2	ntracellular	ein-coding	th protein	G00000155962
28989	0,005819849	0,00873167	0,1497924	0,591377	10,39077414	9,799397175	0,287083	0,13046698	0,0248282	0,4100052	NTMT1	o-Lys N-me	ein-coding	th protein	G00000148335
163	0,004091902	0,00879123	0,150397468	-0,934281	9,693934218	10,62821567	0,045682	-0,479225212	0,217546125	-0,220617459	AP2B1	tein comp	ein-coding	th protein	G00000006125
119559	0,008510503	0,00882021	0,15047772	0,6651577	8,873166473	8,208008786	0,126068	0,233518989	0,114337743	0,257479274	SFXN4	ideroflexin	ein-coding	th protein	G00000183605
58476	0,007227844	0,00882787	0,150401125	0,6813437	8,847097381	8,165753695	0,046904	0,631817989	0,360061661	0,147872141	TP53INP2	3 inducible	ein-coding	th protein	G00000078804
8817	0,010514161	0,00883924	0,150181751	1,0467487	5,843961836	4,797213135	0,075823	0,474433293	0,091171204	0,502811617	FGF18	st growth f	ein-coding	th protein	G00000156427
55314	0,010677649	0,0088393	0,149977079	-0,739689	5,84656453	6,586253719	0,15718	-0,419500293	0,039441181	-0,469033206	TMEM144	mbrane pr	ein-coding	th protein	G00000164124
64792	0,006322053	0,00888134	0,150278592	0,5006513	8,828168618	8,327517276	0,025161	0,287689891	0,983559462	-0,002128855	IFT22	gellar trans	ein-coding	th protein	G00000128581
7416	0,00398915	0,00892756	0,150444094	-0,553907	11,77226569	12,32617251	0,121143	-0,347026239	0,470152495	-0,077747368	VDAC1	endent ani	ein-coding	th protein	G00000213585
3140	0,00510052	0,00893286	0,150328838	-0,791041	6,592830183	7,383871537	0,327206	0,134535747	0,010313007	-0,682006529	MR1	ibility com	ein-coding	th protein	G00000153029
284632	0,001079441	0,00895182	0,150036328	3,098813	3,09881303	0	0,046717	2,63372147	0,422649731	0,616410896					
3482	0,012392169	0,00896617	0,149871303	-0,632002	8,988477325	9,620479221	0,029453	-0,426811843	0,799417512	-0,034275821	IGF2R	rowth fact	ein-coding	th protein	G00000197081
79628	0,011316967	0,00897818	0,14986981	-0,670819	6,199581943	6,870400936	0,029606	-0,293040464	0,199155053	-0,161323531	SH3TC2	tetratrico	ein-coding	th protein	G00000169247
148930	0,010049615	0,00898472	0,149777112	0,6436623	5,768036574	5,124374316	0,068203	0,66653706	0,507286825	0,089775764	KNCN	kinocilin	ein-coding	th protein	G00000162456
57761	0,012211887	0,0090216	0,149988155	0,8846791	9,122109471	8,237430363	0,008132	0,63210085	0,131162751	0,290507755	TRIB3	s pseudoki	ein-coding	th protein	G00000101255
5796	0,010906232	0,00905142	0,150080976	-0,813367	8,193253195	9,00662052	0,113009	-0,404498337	0,228326504	-0,210890981	PTPRK	hosphatas	ein-coding	th protein	G00000152894
3073	0,007897933	0,00905267	0,14990104	-0,761981	9,333153392	10,09513449	0,006937	-0,379783031	0,199294494	-0,219620357	HEXA	nidase sub	ein-coding	th protein	G00000213614
10255	0,004639711	0,00908204	0,15018662	0,7168715	6,444227789	5,72735626	0,109529	0,439805803	0,981808215	-0,001329662	HCG9	oup 9 (non	n-coding R	long non-c	G00000204625
119395	0,00240464	0,00911289	0,149896272	-0,568562	9,342895617	9,911457262	0,517899	-0,196204999	0,370127928	-0,279959456	CALHM3	neostasis r	ein-coding	th protein	G00000183128
4494	0,002332429	0,00913931	0,150131448	-1,684571	8,522713028	10,20728434	0,082188	-0,932308347	0,755027595	-0,100075146	MT1F	allothionei	ein-coding	th protein	G00000198417
390439	0,001122998	0,00914181	0,149973594	2,8466812	2,846681237	0	0,42265	1,487741181		0	OR11G2	mily 11 su	ein-coding	th protein	G00000196832
221710	0,003564931	0,00918289	0,150448296	0,8989355	9,466404625	8,56746909	0,865317	0,052881023	0,765975306	-0,038299079	SMIM13	l membrar	ein-coding	th protein	G00000224531
100652762	0,008461317	0,00921544	0,150583209	-1,611041	7,675654257	9,286694869	0,035095	-0,840313838	0,086902616	-0,588148028					
79947	0,011599331	0,00921652	0,150402372	0,6082675	8,497480495	7,889213027	0,454927	0,147218054	0,269102381	0,167887746	DHDDS	iphosphat	ein-coding	th protein	G00000117682
51676	0,002963847	0,00923063	0,150039662	-1,137747	6,709467341	7,847214169	0,963889	0,009219794	0,067792026	-0,345681246	ASB2	and SOCS b	ein-coding	th protein	G00000100628
58515	0,011120281	0,00933544	0,150754062	0,6093743	12,86757384	12,25819949	0,127636	0,468149239	0,135976159	0,225326643	SELENOK	selenoprotein K(SELENOK)			
283554	0,012403228	0,00933748	0,150590724	-1,840433	2,816181526	4,656614649	0,123464	-2,029484651	0,062294428	0,944653492	GPR137C	oupled rec	ein-coding	th protein	G00000180998
88745	0,00353299	0,00934002	0,150435777	0,8026664	11,61268115	10,81001473	0,141841	0,200346881	0,024903354	0,487526464	RRP36	l RNA proc	ein-coding	th protein	G00000124541
377677	0,002560385	0,00934416	0,150307032	-0,700637	7,018691065	7,719327824	0,001546	-0,48552906	0,181172536	0,165950701	CA13	nic anhydr	ein-coding	th protein	G00000185015
127495	0,002878865	0,0093487	0,150184989	-1,899232	1,351016182	3,250248289	0,917923	0,068652584	0,031259771	-1,073969619	LRRC39	n repeat co	ein-coding	th protein	G00000122477
229	0,007833042	0,00937612	0,150430371	0,7185767	4,711947359	3,993370662	0,552181	0,175570985	0,411887886	0,17414101	ALDOB	uctose-bisp	ein-coding	th protein	G00000136872
23503	0,006718787	0,00939808	0,150587574	-0,504808	6,694014937	7,198822551	0,0627	-0,374046428	0,680770434	-0,039540801	ZFYVE26	YVE-type c	ein-coding	th protein	G00000072121
56479	0,002251487	0,00942098	0,150565094	0,6245181	7,824027145	7,199509013	0,059646	0,344672635	0,329494819	0,13112356	KCNQ5	ed channel	ein-coding	th protein	G00000185760
284422	0,013005036	0,00942848	0,150490762	0,5068473	6,094597896	5,587750599	0,011673	0,626515063	0,409964186	-0,122291148	SMIM24	l membrar	ein-coding	th protein	G00000095932
10002	0,012878043	0,00944172	0,150508024	0,5178621	4,900622285	4,382760151	0,250915	0,365441573	0,384405006	0,097890914	NR2E3	ubfamily 2	ein-coding	th protein	G00000278570

3113	0,00159057	0,00947228	0,150607602	-0,962089	9,809062878	10,77115212	0,253523	-0,071054052	0,008664856	-0,787752696	HLA-DPA1	ility comple	ein-coding	th protein	G00000231389
121053	0,011808107	0,00951505	0,150707126	0,7071294	10,29950118	9,592371822	0,036652	0,245609754	0,081507037	0,334197223	C12orf45	l2 open rea	ein-coding	th protein	G00000151131
29087	0,001533326	0,00955812	0,151195963	0,5371988	9,266126231	8,728927457	0,282162	-0,070060885	0,447271526	-0,062701618	THYN1	te nuclear	ein-coding	th protein	G00000151500
100507436	0,009267306	0,00958782	0,151472352	0,6547563	12,39786015	11,74310389	0,018303	0,315478009	0,118024211	0,167690703	MICA	peptide-rel	ein-coding	th protein	G00000204520
65258	0,006699404	0,00962553	0,151681171	0,5958096	5,968279711	5,372470081	0,043033	0,270627399	0,98912688	-0,001402278	MPPE1	phosphoes	ein-coding	th protein	G00000154889
79714	0,012683692	0,00967145	0,151441446	0,6305092	7,259633603	6,62912441	0,526068	-0,105153497	0,07972953	0,320612314	CCDC51	domain co	ein-coding	th protein	G00000164051
9792	0,013146111	0,00977586	0,152306257	0,5712314	10,16443191	9,593200512	0,030601	0,326858456	0,67040281	0,04781777	SERTAD2	omain con	ein-coding	th protein	G00000179833
27324	0,011941304	0,00980674	0,152403932	1,4422962	4,559430873	3,117134713	0,024482	1,382545589	0,676177943	-0,148168581	TOX3	group box	ein-coding	th protein	G00000103460
10419	0,002622854	0,00984919	0,152680379	0,6395111	9,717478724	9,077967619	0,319654	0,125085742	0,162026142	0,163500474	PRMT5	ine methyl	ein-coding	th protein	G00000100462
80017	0,002350858	0,00992743	0,152936274	-0,716542	6,742230811	7,458772855	0,001512	-0,291410029	0,001304013	-0,297447971	C14orf159	4 open rea	ein-coding	th protein	G00000133943
4358	0,009950884	0,00993965	0,152934244	-0,60651	8,556211874	9,162722274	0,116038	-0,456491597	0,291213147	-0,168113495	MPV17	drial inner	ein-coding	th protein	G00000115204
55762	0,013568426	0,00998786	0,152916308	-0,886904	7,340196866	8,227100404	0,328973	-0,752621401	0,236506363	-0,502376285	ZNF701	nger prote	ein-coding	th protein	G00000167562
4893	0,003627394	0,00999522	0,152840014	-0,567675	9,442371994	10,01004732	0,000877	-0,600802883	0,154311952	0,162783454	NRAS	RAS viral on	ein-coding	th protein	G00000213281
83861	0,011789421	0,01006206	0,153106005	-0,784595	6,090114387	6,874709309	0,28523	-0,136769693	0,050063691	-0,45927165	RSPH3	spoke 3 ho	ein-coding	th protein	G00000130363
134548	0,012015065	0,01006849	0,153015835	0,7159698	6,651587322	5,935617557	0,122902	0,382965232	0,495104339	0,105318008	SOWAHA	repeat don	ein-coding	th protein	G00000198944
55035	0,010843892	0,01007604	0,152942897	0,5300386	8,9339065	8,403867859	0,915732	0,021659967	0,185594232	0,172725256	NOL8	eolar prote	ein-coding	th protein	G00000198000
81894	0,013407388	0,01011283	0,153313471	0,5813251	7,973311662	7,391986595	0,203836	0,235634063	0,028100296	0,366666221	SLC25A28	r family 25	ein-coding	th protein	G00000155287
23223	0,01202147	0,0102259	0,154649045	0,5984214	7,952045437	7,353624038	0,428785	0,16730971	0,462426057	0,131671611	RRP12	A processir	ein-coding	th protein	G00000052749
10939	0,002798754	0,01030244	0,154861162	-0,537841	8,616494141	9,154334885	0,096072	-0,31414414	0,433789792	0,104543905	AFG3L2	x AAA pept	ein-coding	th protein	G00000141385
3486	0,012104972	0,01034915	0,155186606	0,7705508	8,463142669	7,692591852	0,025925	0,60187054	0,724753545	-0,062106333	IGFBP3	th factor b	ein-coding	th protein	G00000146674
26227	0,007109191	0,01040164	0,155409315	1,0158204	5,707125088	4,691304681	0,815571	0,066137981	0,007841038	0,741539484	PHGDH	cerate deh	ein-coding	th protein	G00000092621
3875	0,008602701	0,0104489	0,155927364	0,6180708	12,92919937	12,31112856	0,66213	0,083297317	0,252608159	-0,213342272	KRT18	keratin 18	ein-coding	th protein	G00000111057
27238	0,006565546	0,01046394	0,155963894	0,5786153	9,577808353	8,99919306	0,030784	0,344010198	0,798687194	0,02714695	GPKOW	main and K	ein-coding	th protein	G00000068394
4775	0,013409641	0,01048095	0,155842234	0,5257781	7,155042638	6,629264583	0,307158	0,178319553	0,243913757	0,166534235	NFATC3	or of activa	ein-coding	th protein	G00000072736
8291	0,010137635	0,01054191	0,156186285	-0,579053	6,969977803	7,549030727	0,948769	0,005510588	0,032173057	-0,273660054	DYSF	dysferlin	ein-coding	th protein	G00000135636
23753	0,009316358	0,01057648	0,156324402	1,1756366	12,03772009	10,86208349	0,023535	0,677103599	0,099498956	0,508781514	SDF2L1	derived fa	ein-coding	th protein	G00000128228
9764	0,008801532	0,01061482	0,156331444	0,8533803	6,935711024	6,082330757	0,003221	0,575237512	0,54659438	0,103836235	KIAA0513	KIAA0513	ein-coding	th protein	G00000135709
360205	0,010506008	0,01063339	0,156048292	-0,500331	6,631278361	7,131609428	0,599309	0,085987906	0,007808467	-0,360311692	PRAC2	susceptibi	ein-coding	th protein	G00000229637
222223	0,014048256	0,01065644	0,156201528	-0,910526	4,135777167	5,046303095	0,968934	-0,010458878	0,0176244	-0,72320041	KIAA1324L	IAA1324 lik	ein-coding	th protein	G00000164659
55068	0,009934967	0,01068623	0,156268244	0,5733862	5,502468902	4,929082732	0,008041	-0,350754098	0,011867923	0,570279436	ENOX1	ulfide-thio	ein-coding	th protein	G00000120658
10687	0,009177841	0,01069563	0,156221325	-0,954545	6,143679436	7,098224711	0,036015	-0,797035075	0,21938854	0,173505053	PNMA2	plastic Ma	ein-coding	th protein	G00000240694
25874	0,012722912	0,0107236	0,156077718	0,5289416	11,87651204	11,34757044	0,056634	0,343968007	0,343775369	-0,145374494	MPC2	rial pyruva	ein-coding	th protein	G00000143158
414328	0,013598395	0,01074001	0,15595005	-0,554815	7,663214379	8,218029162	0,465349	-0,139322703	0,019031585	-0,424334928	IDNK	, gluconok	ein-coding	th protein	G00000148057
348645	0,013816228	0,01074564	0,155849028	1,1059257	4,759091371	3,653165649	0,611751	0,248043035	0,862864539	0,07565259	C22orf34	22 open rea	other	unknown	G00000188511
65977	0,01316446	0,01075757	0,155839527	0,7122439	9,858045237	9,145801343	0,060282	0,332666942	0,239776006	0,206499148	PLEKHA3	logy doma	ein-coding	th protein	G00000116095
5271	0,01300658	0,01080965	0,156228551	0,7010169	8,545670865	7,844654	0,318439	0,333813625	0,315811755	0,228370801	SERPINB8	amily B me	ein-coding	th protein	G00000166401
645460	0,008951939	0,01086743	0,156880543	-0,724923	8,934938587	9,659861684	0,304337	-0,305406319	0,736600158	0,05649163					
2308	0,014608642	0,01088361	0,15674875	0,7811364	7,9535506	7,172414228	0,135645	0,317231216	0,118457273	0,307646903	FOXO1	khead box	ein-coding	th protein	G00000150907
1846	0,007556123	0,01088497	0,15658636	0,5686577	9,099307857	8,530650136	0,240967	0,198275168	0,158778241	-0,192827633	DUSP4	ificity phos	ein-coding	th protein	G00000120875
4504	0,003729232	0,01093976	0,157009802	-1,369133	10,24874054	11,61787351	0,098809	-0,724881032	0,003995122	-0,55894594	MT3	tallothione	ein-coding	th protein	G00000087250
353144	0,013045189	0,01099434	0,157428233	0,906893	4,320374117	3,413481096	0,563695	0,507575168	0,983119736	0,014221923	LCE3C	nified enve	ein-coding	th protein	G00000244057
8460	0,007585644	0,01100365	0,157198132	0,5016816	9,139339775	8,637658206	0,019081	0,49288989	0,078641255	0,265774849	TPST1	ein sulfotr	ein-coding	th protein	G00000169902
144165	0,010686627	0,01105989	0,157637927	-0,848593	6,866562135	7,715155526	0,407322	-0,17399403	0,065412313	-0,523734389	PRICKLE1	r cell polar	ein-coding	th protein	G00000139174
10785	0,01399278	0,01106328	0,157505016	0,5859607	7,766847189	7,180886509	0,361771	0,129502889	0,571845184	0,084059289	WDR4	epeat dom	ein-coding	th protein	G00000160193
5094	0,01394172	0,01109849	0,157824902	-0,62997	11,70392151	12,33389158	0,165838	-0,232946489	0,481281273	-0,112360801	PCBP2	) binding p	ein-coding	th protein	G00000197111
10953	0,009610902	0,01110727	0,157768608	-0,69835	8,802379372	9,500728969	0,080892	-0,525199649	0,261413631	0,106916149	TOMM34	r mitochon	ein-coding	th protein	G00000025772
2519	0,00344717	0,01112629	0,157857717	-1,36804	8,925332768	10,29337269	0,047058	-0,415328351	0,069478847	0,19995521	FUCA2	e, alpha-L-	ein-coding	th protein	G00000001036
164592	0,005056455	0,01115699	0,15775113	1,0133939	5,188613929	4,175220031	0,000476	1,215520002	0,850868222	0,033792219	CCDC116	domain cor	ein-coding	th protein	G00000161180
283911	0,013977834	0,01118786	0,157827306	1,9909152	3,843116389	1,85220123	0,77947	-0,242610961	0,217804996	1,291005922					
3624	0,005445681	0,01127726	0,158187634	0,6511792	6,32935497	5,678175759	0,190691	0,447698652	0,656655005	-0,12736126	INHBA	n beta A su	ein-coding	th protein	G00000122641
84293	0,006048812	0,01128529	0,158121203	-0,996616	9,122525869	10,11914184	0,06725	-0,299942592	0,227161983	-0,114406798	FAM213A	nce similar	ein-coding	th protein	G00000122378
100129069	0,002041097	0,01129548	0,157906728	0,5825042	7,190669223	6,608165019	0,276104	0,231367696	0,117906157	0,316846255					
171391	0,004935836	0,01130525	0,157687372	-0,830639	5,238264465	6,068903107	0,227979	-0,718600731	0,055584667	0,159140106					
6856	0,009053568	0,01134106	0,157831436	0,6566305	11,00680104	10,35017051	0,093697	0,417702185	0,077525312	0,346230122	SYPL1	ptophysin l	ein-coding	th protein	G00000008282
554251	0,006482928	0,01136791	0,158027471	-1,221164	5,666676475	6,887840256	0,009498	-0,796497589	0,042871477	0,348960349	FBXO48	ox protein	ein-coding	th protein	G00000204923
3606	0,009843793	0,01136811	0,157853056	-1,170071	6,84808188	8,018152698	0,06964	-0,867652209	0,059125525	-0,390379006	IL18	nterleukin 1	ein-coding	th protein	G00000150782
23157	0,011612965	0,01137877	0,157824204	-0,522886	7,307100919	7,829986566	0,203626	-0,363395837	0,111215882	-0,259259481	SEPT6	septin 6	ein-coding	th protein	G00000125354
11345	0,010519692	0,01146718	0,158164813	-1,309256	9,416227283	10,72548374	0,016608	-0,857892899	0,596890628	-0,123551499	GABARAPL	ptor associ	ein-coding	th protein	G00000034713
55529	0,01495706	0,01147313	0,158070961	0,5381672	9,53438817	8,996220923	0,012222	0,285970662	0,373967976	-0,097182159	TMEM55A	nbrane pr	ein-coding	th protein	G00000155099

80146	0,00753796	0,01149451	0,157838748	-0,976146	8,549951851	9,526098249	0,008737	-0,511303549	0,406049047	-0,162968868	UXS1	onate deca	ein-coding	th protein	G00000115652
118812	0,012115851	0,0114961	0,157685755	0,5914211	7,185004376	6,593583257	0,382851	0,122963098	0,233843621	0,1893228	MORN4	epeat cont	ein-coding	th protein	G00000171160
221079	0,013704338	0,01149974	0,157561162	-0,639077	7,547526632	8,186603733	0,056076	-0,66391087	0,842328959	-0,021222417	ARL5B	ion factor l	ein-coding	th protein	G00000165997
286480	0,004863925	0,01150657	0,157480597	1,1954476	11,72531681	10,52986921	0,026449	0,693336181	0,406364527	-0,213591491	UBE2E4P	ng enzyme	seudogen	seudogene	
284865	0,012858428	0,01150761	0,157320997	2,0007859	2,251297787	0,250511894	0,497808	0,791975157	0,114899091	0,82608913					
3137	0,010663107	0,01150766	0,157148266	-0,751481	14,19326573	14,94474675	0,227674	-0,307067183	0,104937066	-0,338127172	HLA-J	y complex	seudogen	seudogen	G00000204622
56990	0,005438129	0,01151756	0,157110116	0,9395942	8,974362551	8,034768385	0,051974	0,630600974	0,202918426	0,252908519	CDC42SE2	small effe	in-coding	th protein	G00000158985
51251	0,014692969	0,01154543	0,15731712	-0,61696	8,309023389	8,925983563	0,004866	-0,554960135	0,580845795	0,075738901	NT5C3A	tidase, cyt	ein-coding	th protein	G00000122643
865	0,003663399	0,01157054	0,15748605	-0,885897	7,919643202	8,805539844	0,067749	-0,441955103	0,097254702	0,328978497	CBFB	g factor b	ein-coding	th protein	G00000067955
4232	0,002593292	0,01157624	0,157390574	-0,869265	6,039913331	6,909178664	0,002503	-0,795661101	0,072063463	0,361874173	MEST	m specific t	ein-coding	th protein	G00000106484
7700	0,012362702	0,01160025	0,157199407	-0,548696	6,931682304	7,480378698	0,023099	-0,68382607	0,275196064	0,224624516	ZNF141	nger prote	ein-coding	th protein	G00000131127
100129110	0,001842868	0,01160226	0,157054769	-2,53007	2,864149713	5,394220184	0,000133	-5,608084664	0,059494385	0,21386448					
125963	0,00994505	0,01161511	0,157057035	0,6215591	6,390150467	5,768591407	0,046109	0,848892782	0,792060015	-0,060645598	OR1M1	amily 1 sub	ein-coding	th protein	G00000170929
100128942	0,015306397	0,01162592	0,15669005	0,7936601	6,058346494	5,264686417	0,047634	0,708815675	0,979346987	-0,006388035					
728113	0,015389488	0,01165508	0,156912358	-0,797666	7,066560769	7,864227123	0,360371	-0,207403754	0,197746815	-0,340473169	ANXA8L1	nexin A8-lik	ein-coding	th protein	G00000264230
65078	0,015485118	0,01165883	0,156622392	0,9020841	10,25887515	9,356791046	0,067564	1,014841615	0,357899028	-0,179492815	RTN4R	ulon 4 rece	ein-coding	th protein	G00000040608
10456	0,015223436	0,01168654	0,156654838	0,5542033	11,46767979	10,91347651	0,118485	0,263595333	0,184106662	0,164393044	HAX1	sociated pr	ein-coding	th protein	G00000143575
285150	0,001567844	0,01169668	0,156621251	3,3113482	3,311348196	0	0,782933	0,427780647	0,186276581	1,759578356					
283464	0,007014161	0,01170969	0,15662607	-0,994123	6,143054326	7,137177231	0,215667	-0,519253377	0,554655997	0,153872929	GXYLT1	xylosyltra	ein-coding	th protein	G00000151233
386685	0,010824448	0,01174281	0,156561883	0,6025899	6,730813767	6,128223829	0,94896	0,010842569	0,101957279	0,312650857	RTAP10-1	ociated pr	ein-coding	th protein	G00000189169
63939	0,012120297	0,0118115	0,15629993	-0,535671	7,271807612	7,80747803	0,19003	-0,74353007	0,114545044	-0,475172789	FAM217B	nce simila	ein-coding	th protein	G00000196227
80256	0,01440338	0,01183692	0,156135859	0,5992348	6,613434346	6,014199514	0,138407	0,360281736	0,90063774	0,023799429	FAM214B	nce simila	ein-coding	th protein	G00000005238
978	0,002001391	0,01185906	0,156261483	-1,000974	11,24852202	12,2494963	0,011048	-0,522560259	0,015973592	-0,640452737	CDA	line deami	ein-coding	th protein	G00000158825
90580	0,013268286	0,01186583	0,156018762	0,757611	7,83971514	7,082104091	0,864225	0,026290408	0,024373222	0,572557799	C19orf52	l9 open re	ein-coding	th protein	G00000142444
124801	0,014119218	0,01186858	0,1558895	0,6260923	10,79223502	10,1661427	0,308096	0,150119853	0,164842269	0,230866415	LSM12	M12 homo	ein-coding	th protein	G00000161654
55527	0,010234754	0,01186931	0,15573389	0,6106255	7,114166675	6,503541193	0,169962	0,145350749	0,101188119	0,167063689	FEM1A	-1 homolo	ein-coding	th protein	G00000141965
5049	0,008236383	0,01189345	0,155720697	-1,305916	9,645016806	10,95093325	0,016187	-0,905411486	0,897780276	-0,036626657	PAFAH1B2	cetylhydro	ein-coding	th protein	G00000168092
84691	0,0134263	0,0119347	0,15609586	0,7806374	6,277699631	5,497062201	0,003187	0,819596822	0,774525741	-0,049486588	FAM71F1	nce simila	ein-coding	th protein	G00000135248
23642	0,010828643	0,01195582	0,155878183	0,8078164	9,932425835	9,124609447	0,234307	0,158224599	0,376275226	0,160973526	SNHG1	olar RNA h	n-coding R	long non-c	G00000255717
7754	0,012635294	0,01196682	0,155857601	-0,7919	6,462629392	7,254529808	0,634015	-0,219640083	0,028245226	-0,939563157	ZNF204P	otein 204,	seudogen	seudogen	G00000204789
23408	0,008115685	0,01198597	0,15594297	-0,901897	6,671734347	7,57363115	0,077061	-0,339112063	0,944215439	-0,006860998	SIRT5	sirtuin 5	ein-coding	th protein	G00000124523
644246	0,013788125	0,01201601	0,155842791	-1,100154	7,86490774	8,965062171	0,14131	-0,377395323	0,079154821	-0,42811782	ANSL1-AS1	antisense	n-coding R	long non-c	G00000214401
11118	0,013027023	0,01204682	0,155753094	-0,855423	6,45415404	7,309577372	0,226252	-0,597930591	0,154031123	-0,453547909	BTN3A2	subfamily	ein-coding	th protein	G00000186470
255626	0,015608197	0,01205249	0,155663864	2,214288	5,383015108	3,168727154	0,913706	0,065786996	0,086220515	1,281465967	HIST1H2BA	1 H2B fa	ein-coding	th protein	G00000146047
26958	0,002296647	0,01206364	0,155645562	-0,759909	7,639420343	8,399329529	0,045963	-0,401219543	0,380826183	-0,101872476	COPG2	complex	ein-coding	th protein	G00000158623
51531	0,011656814	0,01207565	0,155638379	-0,553933	6,913079954	7,467012506	0,217103	-0,403073689	0,580432644	0,045854625	TRMO	ethyltrans	ein-coding	th protein	G00000136932
1901	0,014735115	0,01207776	0,155503757	-0,837539	6,259021767	7,096560355	0,227601	-0,418600166	0,883156755	-0,025082775	S1PR1	1-phospha	ein-coding	th protein	G00000170989
51014	0,014962446	0,01208076	0,155380972	0,6300015	11,08501856	10,45501707	0,167836	0,191739379	0,050058272	0,330566644	TMED7	e p24 traff	ein-coding	th protein	G00000134970
23215	0,005085213	0,01211303	0,155634396	0,6043204	10,8071396	10,20281917	0,001846	0,528312927	0,543246968	0,066611848	PRRC2C	rich coiled	ein-coding	th protein	G00000117523
5092	0,01278192	0,01215882	0,155899206	-0,691356	9,43830833	10,12966411	0,13287	-0,47748461	0,877226823	-0,015861637	PCBD1	rbinolami	ein-coding	th protein	G00000166228
347902	0,00689156	0,01217299	0,155919505	-0,717041	9,067996386	9,785037004	0,579865	-0,131621619	0,14931985	-0,426217103	AMIGO2	cule with l	ein-coding	th protein	G00000139211
349114	0,016184548	0,01221353	0,15611591	-0,858436	10,61150081	11,46993669	0,002805	-0,671094047	0,517603335	0,110909373	LINC00265	on-protein	n-coding R	long non-c	G00000188185
90417	0,004393291	0,01222345	0,155920856	0,6355912	9,015546624	8,379955427	0,157772	0,206485408	0,051991913	0,356426237	KNSTRN	d astrin/SP	ein-coding	th protein	G00000128944
1535	0,012327348	0,01228532	0,15606714	-0,650121	11,47156915	12,1216902	0,144847	-0,533553491	0,174999243	-0,229731871	CYBA	ne b-245 al	ein-coding	th protein	G00000051523
6041	0,00350478	0,01230139	0,156111208	-2,094962	3,612211937	5,707173489	0,233472	-1,373243991	0,586956871	-0,067991394	RNASEL	onuclease	ein-coding	th protein	G00000135828
1317	0,012520984	0,0123474	0,156214976	0,6955281	8,521387733	7,825859654	0,388312	0,167524128	0,01944771	0,667209933	SLC31A1	er family 3	ein-coding	th protein	G00000136868
64837	0,00318666	0,01235731	0,156021618	0,945211	8,598426398	7,653215401	0,029754	0,27875435	0,004869783	0,345381628	KLC2	sin light ch	ein-coding	th protein	G00000174996
94121	0,007220727	0,01246031	0,156365672	-0,590917	6,293070358	6,883987028	0,228862	-0,414090606	0,29706222	0,144941384	SYTL4	otagmin	ein-coding	th protein	G00000102362
253558	0,005834948	0,01247482	0,156389291	0,8185131	8,692591991	7,874078921	0,153202	0,193435711	0,032934593	0,516727414	LCLAT1	ipin acyltr	ein-coding	th protein	G00000172954
440766	0,014782142	0,01247791	0,156269869	0,5953421	6,718597216	6,123255096	0,621423	0,269444782	0,492555802	0,373831107					
685	0,015568905	0,01249747	0,156356701	-0,758768	5,294002679	6,052770632	0,314695	-0,86925969	0,387589104	0,159199454	BTC	etacellulin	ein-coding	th protein	G00000174808
7594	0,016148143	0,01253029	0,156293792	-0,902584	6,951229056	7,853813193	0,086561	-0,758665949	0,395415438	0,16588094	ZNF43	inger prot	ein-coding	th protein	G00000198521
100130889	0,01568718	0,01253252	0,156164277	0,8128296	7,544898142	6,732068532	0,153253	0,324587859	0,268916327	0,265855839	PSORS1C3	candidate	n-coding R	long non-c	G00000204528
51540	0,006100639	0,01255896	0,156179487	0,6708222	7,889216487	7,218394328	0,111856	0,150941251	0,027252452	0,458295093	SCLY	ocysteine	ein-coding	th protein	G00000132330
127933	0,008278216	0,01256903	0,155991482	-0,756802	7,948151358	8,70495319	0,028446	-0,508568422	0,49572383	0,120900191	UHMK1	ology mot	ein-coding	th protein	G00000152332
388730	0,015451198	0,01260737	0,155998408	2,7059368	3,180063788	0,47412701	0,756229	0,3678838	0,091677292	2,487197612	TMEM81	mbrane pr	ein-coding	th protein	G00000174529
6624	0,006342657	0,01262033	0,156002866	-1,605285	5,528738673	7,134023389	0,033502	-0,992980824	0,166991133	-0,277657381	FSCN1	n-bundling	ein-coding	th protein	G00000075618
375057	0,014353159	0,01265128	0,156229522	0,6387443	5,50390889	4,865164562	0,746024	0,128109755	0,626585178	-0,057302244	STUM	y transduct	ein-coding	th protein	G00000203685
51390	0,004723592	0,01266923	0,15598473	-1,104963	8,7982292	9,903192018	0,03226	-0,367170829	0,025334377	-0,310199653	AIG1	ogen induc	ein-coding	th protein	G00000146416

23678	0,003620953	0,01270697	0,156138966	-1,010139	5,361118229	6,371256942	0,268894	-0,495082017	0,282081079	0,197698622	SGK3	egulated kain-coding	th protein	G00000104205
27336	0,008180005	0,01273067	0,156275099	-0,604745	8,295774062	8,900518942	0,260789	-0,552464302	0,391272043	0,068138978	HTATSF1	at specific ein-coding	th protein	G00000102241
148046	0,013978063	0,01275401	0,156406612	2,1147799	5,567093259	3,452313317	0,532567	0,30874359	0,077084753	1,182384024	CIRBP-AS1	antisense n-coding R	long non-c	G00000267493
5029	0,009051198	0,01276602	0,156399068	0,6932464	6,307509212	5,614262821	0,18612	0,283883587	0,021742711	0,291446148	P2RY2	gic receptein-coding	th protein	G00000175591
79683	0,011856979	0,01278595	0,156488456	0,6498963	6,648775436	5,998879111	0,762483	0,032643701	0,040795329	0,346756571	ZDHHC14	HHC-type cein-coding	th protein	G00000175048
1855	0,007259458	0,01279313	0,156421715	0,6655909	8,748179968	8,082589086	0,39332	0,126452105	0,121099671	0,256735706	DVL1	gment polaein-coding	th protein	G00000107404
3937	0,016121542	0,01290336	0,156994595	0,5374623	5,530862281	4,993400009	0,360464	0,257967016	0,412742615	0,126078067	LCP2	te cytosolicein-coding	th protein	G00000043462
1427	0,005351003	0,01293663	0,15693691	-1,115145	4,866505047	5,981649973	0,306849	-0,112633463	0,602364919	0,058777887	CRYGS	tallin gamrein-coding	th protein	G00000213139
136853	0,016987656	0,01295219	0,156665368	0,5714197	6,426331721	5,854912058	0,004638	0,742455949	0,281170742	-0,1660576	SSC4D	e rich famiein-coding	th protein	G00000146700
728591	0,003260834	0,01296324	0,156646022	-0,914585	4,978915326	5,893500437	0,383002	-0,297448309	0,705974779	0,074466038	CCDC169	domain corein-coding	th protein	G00000242715
57486	0,003107093	0,01297212	0,156600527	-1,11406	7,302112006	8,416172263	0,032053	-0,894700733	0,491771328	0,121617928	NLN	neurolysinein-coding	th protein	G00000123213
11020	0,016795651	0,01297753	0,156513312	-0,588962	7,551093723	8,140055295	0,695515	-0,053806525	0,449759835	0,120612957	IFT27	gellar transein-coding	th protein	G00000100360
7057	0,005591929	0,01298012	0,156392316	-1,093744	11,31318261	12,40692624	0,002435	-0,486673577	0,08244954	-0,512324317	THBS1	mbosponcein-coding	th protein	G00000137801
728431	0,002883698	0,01301209	0,156625089	-1,830403	5,674868144	7,505271355	0,003322	-1,432522906	0,119079699	-0,396169941	LINC01137	on-protein n-coding R	long non-c	G00000233621
2870	0,011517358	0,01307753	0,15695523	0,6890148	8,957661375	8,268646611	0,546751	-0,031745103	0,130821695	0,285779376	GRK6	upled receein-coding	th protein	G00000198055
9419	0,00809594	0,0131394	0,157240693	0,6412969	10,21749473	9,576197875	0,270076	0,285151511	0,166608342	0,15060648	CRIPT	ning interaein-coding	th protein	G00000119878
58525	0,016452048	0,01314887	0,157202079	0,5233311	6,819501185	6,296170099	0,495769	-0,263457197	0,326797866	0,403323558	WIZ	paced zinc ein-coding	th protein	G00000011451
158801	0,010715403	0,01319093	0,157401083	-0,522533	6,28595321	6,808486548	0,996853	0,002293041	0,26798743	-0,676265897	NKAPP1	ng protein pseudogen	oseudogen	G00000233382
27436	0,017139226	0,01319569	0,157306371	0,5046657	8,925128768	8,420463113	0,041441	0,289648705	0,028639166	0,348577074	EML4	ubule assoein-coding	th protein	G00000143924
7294	0,01647002	0,01321049	0,157331393	0,6986855	5,079884685	4,381199137	0,233124	0,293090973	0,056374125	0,361981697	TXK	tyrosine kiein-coding	th protein	G00000074966
26002	0,013714065	0,01327684	0,157666731	-0,765993	4,288797954	5,054790744	0,683412	-0,088699743	0,226573122	-0,307164229	MOXD1	ygenase DBein-coding	th protein	G00000079931
400221	0,015581195	0,01335096	0,158243525	-0,935766	6,562126519	7,497892125	0,11085	-0,503069057	0,018750337	-0,839883996				
114883	0,017505546	0,01335951	0,158193487	0,57686	9,302442104	8,725582082	0,052892	0,394762516	0,199104727	0,181854617	OSBPL9	binding proein-coding	th protein	G00000117859
128338	0,002960713	0,01343989	0,158841607	-0,520646	8,378492957	8,899139429	0,397084	-0,208930874	0,105330431	-0,173974013	DRAM2	lated autopein-coding	th protein	G00000156171
10905	0,017293511	0,01345808	0,158753577	0,6699539	9,507684209	8,837730317	0,244253	0,277292259	0,059905634	0,392357308	MAN1A2	alpha classein-coding	th protein	G00000198162
10011	0,010744467	0,01348408	0,158908879	0,6442399	10,72423767	10,07999777	0,046924	0,298365468	0,205881325	0,206708366	SRA1	eptor RNA ein-coding	th protein	G00000213523
55157	0,004032579	0,01349049	0,158833334	-0,766474	7,134699464	7,901173163	0,074212	-0,396717058	0,004732913	-0,525446708	DARS2	ynthetase 2ein-coding	th protein	G00000117593
4153	0,013235799	0,01350352	0,158835771	0,7121364	5,096325945	4,384189496	0,010467	0,667344418	0,673235718	-0,076018117	MBL2	se binding ein-coding	th protein	G00000165471
57834	0,010594988	0,01350941	0,158754261	0,5870904	6,2671616	5,680071153	0,083795	0,663564583	0,901957634	-0,021885191	CYP4F11	mily 4 subein-coding	th protein	G00000171903
171546	0,01155955	0,01352908	0,158834695	0,5535114	11,01005158	10,45654021	0,013298	0,406457437	0,054421316	0,213911805	SPTSSA	transferaseein-coding	th protein	G00000165389
55556	0,013794966	0,01362763	0,159086934	-0,51417	7,900267195	8,414437561	0,083929	-0,310835976	0,546299651	0,076651332	ENOSF1	uperfamily ein-coding	th protein	G00000132199
11031	0,005762312	0,0136337	0,159008028	-0,564241	10,18847991	10,75272139	0,015005	-0,407393871	0,054687682	-0,137675419	RAB31	ber RAS onein-coding	th protein	G00000168461
440712	0,01635884	0,01363973	0,158928622	0,8164892	7,908283797	7,091794605	0,093473	0,39617675	0,430224461	0,180503156	C1orf186	l open reaein-coding	th protein	G00000263961
1845	0,017614178	0,01367191	0,158855316	-0,524256	9,032116165	9,556371678	0,044563	-0,330017748	0,238529831	-0,151818629	DUSP3	ificity phosein-coding	th protein	G00000108861
51335	0,002313606	0,0137223	0,159142242	0,8155655	9,378933508	8,563368053	0,188859	0,133572036	0,070373139	0,309371853	NGRN	ite outgrovein-coding	th protein	G00000182768
116151	0,011006186	0,01378907	0,159766939	-0,583219	8,273816382	8,857035657	0,052763	-0,67875384	0,605884419	-0,082197778	FAM210B	nce similarein-coding	th protein	G00000124098
5817	0,004030767	0,01381106	0,159722926	0,5811618	9,276002868	8,694841088	0,117425	0,217046771	0,142917233	0,185395383	PVR	ovirus receein-coding	th protein	G00000073008
54979	0,016168923	0,01386693	0,160070106	0,5546514	6,553018727	5,998367302	0,010201	0,277507244	0,592833259	-0,050290848	HRASLS2	like supprein-coding	th protein	G00000133328
23345	0,009115917	0,01388029	0,160075181	0,5001103	4,711283168	4,211172842	0,005682	0,472413755	0,226996973	-0,150451593	SYNE1	ining nucleein-coding	th protein	G00000131018
1586	0,018197221	0,01388725	0,160006456	0,5519707	5,468436853	4,916466183	0,096525	0,493349277	0,484849516	-0,101590291	CYP17A1	mily 17 subein-coding	th protein	G00000148795
53940	0,014483631	0,01390758	0,159943079	-1,174639	13,39730703	14,57194586	0,227828	-0,612512314	0,333196904	-0,44245561	FTHL17	heavy chaiein-coding	th protein	G00000132446
27077	0,002375802	0,01390871	0,159807742	1,0815563	9,505252733	8,423696392	0,316511	0,286967272	0,197343426	0,248875386	B9D1	main contaein-coding	th protein	G00000108641
340260	0,011393387	0,0139093	0,159666414	0,7190645	7,254679082	6,535614574	0,075368	0,714640557	0,392585382	-0,182661013	UNCX	IC homeobein-coding	th protein	G00000164853
11014	0,00249555	0,01391078	0,159535479	0,9130691	12,0593366	11,14626746	0,002655	0,549565433	0,039136617	0,453252101	KDELR2	ulum proteinein-coding	th protein	G00000136240
64854	0,01525974	0,01391742	0,159463975	-1,064367	6,577127649	7,641494228	0,067564	-0,84237259	0,188495321	0,276004402	USP46	specific pepein-coding	th protein	G00000109189
3914	0,017360295	0,01393675	0,159537921	0,5127557	10,23127123	9,718515567	0,423959	0,07962164	0,271103654	-0,11914961	LAMB3	in subunit bein-coding	th protein	G00000196878
6574	0,016313721	0,01402158	0,159477303	0,7411476	11,9286268	11,18747915	0,118633	0,384551705	0,054516101	0,46736063	SLC20A1	er family 2ein-coding	th protein	G00000144136
83647	0,017917921	0,01407802	0,159825703	0,6788536	4,015417407	3,336563838	0,045368	1,114764587	0,440115202	-0,281574588	LINC00529	on-protein n-coding R	long non-c	G00000236827
22841	0,00243671	0,01411817	0,159841966	0,6956856	9,104410077	8,408724514	0,179508	0,206725842	0,137909486	0,251680528	RAB11FIP2	ly interactiein-coding	th protein	G00000107560
80760	0,0168668	0,01412132	0,159731642	0,7633878	3,977292487	3,213904724	0,066245	1,02777322	0,605280214	-0,197682359	ITIH5	bitor heavyein-coding	th protein	G00000123243
55175	0,003978091	0,0142085	0,160132829	0,862415	7,957764407	7,095349444	0,027848	0,352153038	0,070113101	0,381630248	KLHL11	e family meein-coding	th protein	G00000178502
280636	0,003313586	0,01421532	0,160063973	-0,711583	11,43781655	12,14939999	0,002402	-0,304731419	0,027256241	-0,147036052	C11orf31	11 open reein-coding	th protein	G00000211450
6373	0,01793685	0,01423224	0,160108909	2,9992695	5,554773619	2,555504107	0,228983	1,718300115	0,40211026	0,999057082	CXCL11	f chemokinein-coding	th protein	G00000169248
391211	0,002232647	0,01426329	0,160312603	3,4377935	3,43779355	0	0,186972	2,092415159		0	OR2G6	amily 2 subein-coding	th protein	G00000188558
84449	0,016177164	0,01430641	0,160506036	0,5684469	7,17718126	6,60873433	0,005373	0,199949376	0,572474935	0,070722853	ZNF333	nger proteiein-coding	th protein	G00000160961
2296	0,003328573	0,01431194	0,160422702	0,8429199	9,059012644	8,21609276	0,075528	0,314760934	0,040685165	0,444337239	FOXC1	khead boxein-coding	th protein	G00000054598
9732	0,012271316	0,01439001	0,160716094	0,6383265	7,816147123	7,177820586	0,425634	0,144492866	0,123676198	0,331802743	DOCK4	or of cytokein-coding	th protein	G00000128512
375287	0,004590268	0,01443067	0,161024994	-0,794028	7,679172516	8,473200304	0,980654	-0,006747632	0,053639933	-0,526092232	RBM43	ing motif pein-coding	th protein	G00000184898
646268	0,008392847	0,01444432	0,160887379	1,865764	3,347704101	1,481940071	0,207579	2,153538231	0,837559603	-0,284039951				

23576	0,006939298	0,01455103	0,161639872	0,7057346	8,97156617	8,265831556	0,212159	0,332861405	0,106083751	0,306910591	DDAH1	dimethyla	ein-coding	th protein	G00000153904
286234	0,003734572	0,01457239	0,161732156	0,9988398	5,249952253	4,251112485	0,010045	1,12022849	0,388234732	-0,198542184	SPATA31E1	ubfamily E	ein-coding	th protein	G00000177992
54922	0,004241217	0,01457621	0,161629688	-0,646939	5,331927579	5,978866861	0,664393	-0,100455937	0,09374841	-0,269948342	RASIP1	eracting pr	ein-coding	th protein	G00000105538
346653	0,004596157	0,01464832	0,161994213	0,6447976	6,608636602	5,963838975	0,494341	-0,180171653	0,593999215	0,094750015	FAM71F2	nce similar	ein-coding	th protein	G00000205085
100129427	0,014503978	0,01465641	0,161939046	1,4110535	5,323511214	3,912457759	0,23902	0,470899704	0,038061968	0,922703958	DDC-AS1	antisense R	n-coding R	long non-c	G00000226122
493856	0,019053506	0,01466235	0,161860381	-1,007401	8,886548018	9,893948831	0,233841	-0,632425824	0,941593135	0,022197936	CISD2	ron sulfur c	ein-coding	th protein	G00000145354
6734	0,005043715	0,01468342	0,161804472	0,7547122	10,04389048	9,289178238	0,025708	0,3180602	0,060060478	0,397286954	SRPRA	eptor alpha	ein-coding	th protein	G00000182934
3164	0,014216015	0,01468407	0,161667813	0,8084938	6,365393631	5,556899793	0,057317	0,474750739	0,863633491	-0,025931778	NR4A1	ubfamily 4	ein-coding	th protein	G00000123358
10139	0,018093782	0,01470772	0,161497534	0,5043174	9,323145309	8,818827955	0,152778	0,244110622	0,865428352	0,024786511	ARFRP1	on factor re	ein-coding	th protein	G00000101246
5272	0,018649639	0,0147105	0,161384955	0,6299462	8,035889666	7,405943431	0,502984	0,272855797	0,60789994	0,118190466	SERPINB9	amily B me	ein-coding	th protein	G00000170542
4670	0,002399064	0,01471598	0,161302275	0,5121329	10,3225268	9,810393883	0,125815	0,205454884	0,007341962	0,257234539	HNRNPM	uclear ribo	ein-coding	th protein	G00000099783
128602	0,010874444	0,01480957	0,161898246	0,5951509	5,397067541	4,801916634	0,268453	0,497840325	0,108662908	0,285576029	C20orf85	20 open re	ein-coding	th protein	G00000124237
26038	0,00783536	0,01485671	0,162127428	0,5478656	5,997929498	5,450063889	0,113031	0,418116071	0,578772085	0,063988866	CHD5	elicase DNA	ein-coding	th protein	G00000116254
283143	0,019196481	0,01487027	0,162132579	0,6959085	8,29873343	7,602824969	0,148182	0,575873772	0,627289493	0,071855456	LINC00900	on-protein	n-coding R	long non-c	G00000246100
84275	0,002746893	0,01489424	0,162251025	0,7016808	8,471540161	7,769859385	0,012903	0,37894869	0,0252593	0,384912856	SLC25A33	r family 25	ein-coding	th protein	G00000171612
7094	0,01828523	0,01490938	0,162130846	0,5575577	9,584051268	9,026493571	0,178874	0,234239431	0,287004997	0,193806614	TLN1	talin 1	ein-coding	th protein	G00000137076
5806	0,00587069	0,01492136	0,162118753	0,9887832	7,364125773	6,375342593	0,869808	0,041991684	0,455823395	0,176256898	PTX3	pentraxin 3	ein-coding	th protein	G00000163661
6456	0,014771351	0,014935	0,162124733	0,6698036	4,923712825	4,253909258	0,81077	0,117287532	0,060352689	0,421712327	SH3GL2	ning GRB2 l	ein-coding	th protein	G00000107295
55171	0,007492829	0,01497149	0,162236424	-0,514041	6,62502263	7,13906404	0,352461	-0,256274451	0,341519375	0,156133935	TBCCD1	omain cont	ein-coding	th protein	G00000113838
47	0,004779369	0,01500202	0,162000335	0,9162086	10,0478432	9,131634549	0,008046	0,416731271	0,189879315	0,253766101	ACLY	P citrate ly	ein-coding	th protein	G00000131473
29968	0,014418133	0,01500779	0,161921459	0,7817719	8,528133883	7,746362022	0,185577	-0,169294568	0,023876516	0,839668094	PSAT1	ine aminot	ein-coding	th protein	G00000135069
5680	0,018026498	0,01504768	0,162210623	-1,898226	4,728686727	6,62691243	0,200977	-0,535941848	0,203639166	-0,686222763	PSG11	ific beta-1-	ein-coding	th protein	G00000243130
57584	0,01942162	0,01505223	0,162118498	-0,802432	8,838910214	9,641342316	0,064628	-0,575627352	0,469278464	-0,127242605	ARHGAP21	e activating	ein-coding	th protein	G00000107863
55924	0,019375676	0,01507079	0,162036591	0,789639	5,991170936	5,201531907	0,003673	0,710132793	0,467958955	0,133209158	FAM212B	nce similar	ein-coding	th protein	G00000197852
403341	0,019251781	0,01511195	0,162197614	0,5345721	9,199266998	8,664694862	0,015422	0,515080489	0,320561727	-0,148809249	ZBTB34	BTB domai	ein-coding	th protein	G00000177125
29766	0,007009191	0,01515331	0,162219815	-1,402704	9,046821422	10,44952587	0,000891	-1,180731894	0,691249227	-0,107681673	TMOD3	opomoduli	ein-coding	th protein	G00000138594
259232	0,01879203	0,01515491	0,162096829	0,549295	4,914143551	4,364848559	0,16074	0,400035375	0,688126213	0,08005163	NALCN	channel, n	ein-coding	th protein	G00000102452
7525	0,017993639	0,01516219	0,162034803	-0,842443	7,992935304	8,835378542	0,035893	-0,669098156	0,215509174	-0,231723019	YES1	e 1, Src far	ein-coding	th protein	G00000176105
23478	0,009808937	0,01518911	0,162182556	-0,806555	10,65088234	11,45743712	0,147192	-0,361390298	0,364796842	-0,222307706	SEC11A	gnal peptid	ein-coding	th protein	G00000140612
55163	0,008764989	0,01521373	0,162026359	-0,602104	8,340211855	8,942315375	0,213052	-0,133559707	0,93606583	0,010428157	PNPO	e 5'-phosp	ein-coding	th protein	G00000108439
23630	0,009242933	0,01523208	0,161804422	1,1394251	8,185300369	7,045875238	0,036418	0,424438336	0,217535592	0,343446919	KCNE5	annel subf	ein-coding	th protein	G00000176076
3196	0,01730151	0,01530798	0,162332235	0,614853	6,843438751	6,228585753	0,777827	0,113843859	0,329912615	0,363042263	TLX2	ukemia hon	ein-coding	th protein	G00000115297
100289388	0,019795565	0,01539903	0,162879357	-0,812811	5,93498179	6,747792689	0,031949	-2,339262011	0,228053116	0,352495975	KCTD21-AS1	1 antisense	n-coding R	long non-c	G00000246174
7428	0,011279866	0,01541733	0,162933818	0,5688872	9,255436155	8,686548924	0,340836	0,098163724	0,245079465	0,16436317	VHL	indau tum	ein-coding	th protein	G00000134086
115509	0,014618407	0,01547505	0,163404386	0,7121229	8,269604652	7,557481714	0,340053	0,188565021	0,354472951	0,19029902	ZNF689	nger prote	ein-coding	th protein	G00000156853
91319	0,019153226	0,01549014	0,163424463	0,6551977	7,718540069	7,063342414	0,036592	0,664165749	0,919978568	-0,01273343	DERL3	derlin 3	ein-coding	th protein	G00000099958
2118	0,018779733	0,01549423	0,163328396	0,7734492	8,264864338	7,491415142	0,030454	0,488740469	0,097330684	0,386471475	ETV4	TS variant	ein-coding	th protein	G00000175832
51182	0,005379853	0,01551109	0,163367091	-0,701695	8,968329528	9,670024266	0,012883	-0,404152201	0,086063689	0,18041039	HSPA14	family A (	ein-coding	th protein	G00000187522
50854	0,019357318	0,01552713	0,163397151	0,8159474	12,19365158	11,37770416	0,119669	0,389181554	0,502303395	-0,116462069	C6orf48	6 open rea	ein-coding	th protein	G00000204387
10866	0,0083462	0,01552975	0,163285952	-0,561762	9,211611008	9,773373052	0,633469	-0,072425007	0,243353514	-0,123846281	HCP5	P5 (non-pr	n-coding R	long non-c	G00000206337
28964	0,01213054	0,01561306	0,163883725	-0,56392	7,380590192	7,944510299	0,135886	-0,382766997	0,904210314	-0,022352793	GIT1	GIT ArfGAP	ein-coding	th protein	G00000108262
826	0,016651479	0,01564927	0,163847199	-0,520377	12,2509983	12,77137482	0,25227	-0,147188016	0,740517894	-0,044917563	CAPNS1	n small sub	ein-coding	th protein	G00000126247
80129	0,020040464	0,0156542	0,163622145	1,4019488	3,7725583	2,370609461	0,010343	0,683164932	0,080783563	0,746318083	CCDC170	domain cor	ein-coding	th protein	G00000120262
11328	0,007285532	0,01565698	0,16337549	-0,68447	8,359980589	9,044450843	0,083873	-0,254226184	0,174281918	-0,238284107	FKBP9	binding pr	ein-coding	th protein	G00000122642
84100	0,017335272	0,01567574	0,163296158	0,9175924	7,20558061	6,287988188	0,490711	0,286832587	0,314754229	0,22213798	ARL6	tion factor	ein-coding	th protein	G00000113966
26686	0,011530837	0,01568826	0,16315213	1,5845005	4,213414581	2,628914064	0,142018	1,032323905	0,942387774	-0,041689636	OR4E2	amily 4 sub	ein-coding	th protein	G00000221977
728763	0,019132279	0,01569215	0,163055662	0,675544	5,575641668	4,900097718	0,729958	0,080106129	0,524082315	0,101717541	CROCC2	coil, rootle	ein-coding	th protein	G00000226321
152503	0,003062304	0,01571454	0,163151453	-0,740419	8,228813604	8,969232224	0,084713	-0,252081152	0,560334002	-0,069863089	SH3D19	main conta	ein-coding	th protein	G00000109686
121512	0,016450829	0,01573953	0,16327399	-0,959242	4,550310135	5,509551792	0,038246	-0,668710543	0,680221354	-0,09091582	FGD4	nd PH dom	ein-coding	th protein	G00000139132
1829	0,014227579	0,01579421	0,163567239	-1,208435	7,37112533	8,579560069	0,027138	-0,72384799	0,461752223	-0,217587983	DSG2	esmoglein	ein-coding	th protein	G00000046604
2230	0,018524309	0,01588999	0,164284482	-0,622202	8,895739603	9,517941866	0,019825	-0,47179552	0,505692672	-0,094931912	FDX1	erredoxin	ein-coding	th protein	G00000137714
7407	0,006132549	0,01590579	0,164310668	0,6874105	10,00855012	9,321139653	0,071175	0,259154921	0,130537629	0,264158119	VARS	tRNA synth	ein-coding	th protein	G00000204394
1509	0,009703387	0,01599207	0,164926823	-0,527492	12,64102739	13,16851928	0,224774	-0,160941251	0,078047187	-0,273303306	CTSD	cathepsin D	ein-coding	th protein	G00000117984
729967	0,018495088	0,01609028	0,165801591	-0,925565	8,275895855	9,20146131	0,283729	-0,394761583	0,260371537	-0,438570412	MORN2	epeat cont	ein-coding	th protein	G00000188010
8687	0,018842704	0,01610384	0,165665671	1,66014	4,135741339	2,47560137	0,037048	1,140669704	0,108139821	0,781745871	KRT38	keratin 38	ein-coding	th protein	G00000171360
144100	0,011566678	0,01610639	0,165554428	-0,694224	6,711384975	7,405608658	0,580184	-0,084013309	0,059587498	-0,350874374	PLEKHA7	logy doma	ein-coding	th protein	G00000166689
6349	0,017214067	0,0161715	0,165810932	0,5264754	7,145603306	6,619127875	0,122743	0,619767968	0,51101488	-0,124831436	CCL3L1	emokine li	ein-coding	th protein	product
91768	0,004031681	0,01618734	0,165836067	-0,815699	6,776205159	7,591904324	0,159038	-0,459699691	0,984571467	-0,003323856	CABLES1	bl enzyme	ein-coding	th protein	G00000134508
387882	0,006922047	0,01619753	0,165803326	0,7464955	9,045472622	8,298977125	0,061475	0,521240013	0,33608389	0,186621095	C12orf75	12 open re	ein-coding	th protein	G00000235162

4295	0,01970593	0,01626118	0,166180167	0,7747323	6,002275842	5,227543576	0,071116	0,377619662	0,158679797	0,262319507	MLN	motilin	ein-coding	th protein	G00000096395
2271	0,018548735	0,01626376	0,166069485	0,7019243	10,76139043	10,05946613	0,986601	0,003387764	0,085530847	0,381298426	FH	arate hydra	ein-coding	th protein	G00000091483
10069	0,019551666	0,01630576	0,166087617	-0,509989	7,782946858	8,292935724	0,193675	-0,121758893	0,181958016	0,192575363	RWDD2B	main conta	ein-coding	th protein	G00000156253
399818	0,004833242	0,01630645	0,165958107	-0,64213	8,04872548	8,690855711	0,145422	-0,310349577	0,64826302	0,058375243	METTL10	transferase	ein-coding	th protein	G00000203791
56204	0,02030969	0,01632617	0,165886184	-0,750348	5,915276784	6,665625237	0,274042	-0,339328946	0,592208323	-0,079808449	FAM214A	nce similar	ein-coding	th protein	G00000047346
55663	0,006522509	0,01632864	0,165775288	0,5771398	7,023755348	6,446615565	0,490059	0,138145742	0,150574855	0,242162202	ZNF446	nger prote	ein-coding	th protein	G00000083838
254295	0,020475307	0,0163442	0,165526272	-0,671977	9,215484531	9,887461505	0,415548	-0,261059971	0,151420561	-0,295143694	PHYHD1	ygenase d	ein-coding	th protein	G00000175287
98	0,004108558	0,01634442	0,165393264	1,3297797	10,69705826	9,367278533	0,012811	0,566162792	0,140308637	0,426542147	ACYP2	phosphata	ein-coding	th protein	G00000170634
2512	0,005817912	0,01634857	0,165165359	-0,529547	15,90342034	16,43296732	0,666179	-0,042549442	0,003911957	-0,482951282	FTL	itin light c	ein-coding	th protein	G00000087086
231	0,019361691	0,01645985	0,165883638	-1,325125	11,91331559	13,23844084	0,063986	-0,755139391	0,497030954	-0,188623248	AKR1B1	ictase fami	ein-coding	th protein	G00000085662
23228	0,006065475	0,01651893	0,165804558	-2,418074	4,661966499	7,080040038	0,055675	-1,903821112	0,627737558	0,21670803	PLCL2	pholipase C	ein-coding	th protein	G00000154822
3726	0,006618497	0,01657315	0,166214057	0,5345978	8,712087817	8,177490025	0,103327	0,364727152	0,249166304	0,220110394	JUNB	AP-1 trans	ein-coding	th protein	G00000171223
11199	0,004134609	0,0165888	0,166236466	0,674213	5,576194525	4,901981546	0,258104	0,4272822	0,238202796	-0,448738322	ANXA10	nnexin A1	ein-coding	th protein	G00000109511
9448	0,007852171	0,01659564	0,166170502	-1,086561	9,335012712	10,42157378	0,033097	-0,492042031	0,909172328	-0,025241778	MAP4K4	tein kinase	ein-coding	th protein	G00000071054
92162	0,020901425	0,01661159	0,166061775	0,6829544	7,292628039	6,609673599	0,014285	0,536897528	0,491093818	0,116389811	TMEM88	mbrane pr	ein-coding	th protein	G00000167874
55454	0,019322677	0,01667844	0,166193531	0,7457233	8,5261425	7,780419249	0,278373	0,217070554	0,08019108	0,442914032	SGALNACT	cetylgalac	ein-coding	th protein	G00000169826
59284	0,019097294	0,01673402	0,166613316	0,7760293	5,789302518	5,013273171	0,093132	0,49022893	0,254264198	0,274132093	CACNG7	channel au	ein-coding	th protein	G00000105605
55916	0,00473081	0,01674183	0,166557159	-0,506234	6,855947683	7,36218173	0,23489	-0,140129736	0,223839079	-0,140979292	NXT2	factor 2 lil	ein-coding	th protein	G00000101888
10863	0,008637712	0,0167934	0,166668621	-1,102139	5,422623488	6,524762834	0,080617	-0,742590953	0,055352874	-0,863652356	ADAM28	llopeptidas	ein-coding	th protein	G00000042980
1437	0,019434946	0,01679894	0,166590062	-0,962778	9,510707445	10,47348565	0,085495	-0,477089265	0,477083957	0,174520332	CSF2	timulating	ein-coding	th protein	G00000164400
7980	0,004236914	0,01680554	0,166522178	0,8207677	12,24745716	11,42668946	0,003422	0,593430032	0,637782936	-0,071211281	TFPI2	or pathway	ein-coding	th protein	G00000105825
10383	0,017796291	0,01681062	0,166439374	0,5476248	11,98362553	11,43600074	0,848979	0,038208157	0,140916508	0,247170021	TUBB4B	beta 4B cl	ein-coding	th protein	G00000188229
3777	0,007232524	0,01687511	0,166811243	0,5020032	7,016929794	6,514926556	0,01765	0,638418949	0,235939848	-0,137508246	KCNK3	ain chann	ein-coding	th protein	G00000171303
120863	0,018179853	0,01689064	0,166698608	-0,868847	3,192392257	4,061238994	0,228958	-1,123934054	0,17253883	0,41004708	DEPDC4	main conta	ein-coding	th protein	G00000166153
1519	0,012193911	0,01705933	0,16809561	-1,821356	4,630450758	6,451806782	0,108826	-0,899915935	0,067149507	-1,171044145	CTSO	cathepsin C	ein-coding	th protein	G00000256043
401505	0,020674127	0,01706919	0,16805907	0,7026523	8,440106855	7,737454566	0,240051	0,16767977	0,462973125	0,136910292	TOMM5	er mitocho	ein-coding	th protein	G00000175768
81606	0,017028846	0,01708161	0,168047778	0,5569567	7,563354397	7,006397649	0,057703	0,417066399	0,34586895	0,110206688	LBH	d heart de	ein-coding	th protein	G00000213626
4883	0,018932918	0,01708573	0,16795489	1,890183	3,521993159	1,631810197	0,137995	2,135035943	0,546189537	-0,688998163	NPR3	c peptide r	ein-coding	th protein	G00000113389
400673	0,014030521	0,0171346	0,167769522	-0,748255	6,340861061	7,089115933	0,015669	-0,970300478	0,830056637	-0,022138224	VMAC	filament a	ein-coding	th protein	G00000187650
11009	0,018352872	0,01713875	0,167545327	0,8404696	7,529952949	6,689483318	0,110614	0,870033825	0,304384671	0,227752542	IL24	terleukin 2	ein-coding	th protein	G00000162892
10236	0,01983324	0,01715586	0,167580312	0,6587242	11,04786422	10,38914	0,805351	0,035954085	0,150621639	0,239104134	HNRNPR	uclear ribo	ein-coding	th protein	G00000125944
8892	0,007533825	0,01717368	0,167622154	0,597914	10,26532852	9,66741449	0,05183	0,303450521	0,291413469	0,143784923	EIF2B2	initiation f	ein-coding	th protein	G00000119718
221442	0,00517695	0,01718261	0,167577295	1,3066888	5,656833353	4,350144522	0,981577	-0,006304067	0,040256744	0,765591989	ADCY10P1	e 10, solub	seudogeno	seudogen	G00000161912
5132	0,007263539	0,01721162	0,167728157	1,1600986	5,337804801	4,177706213	0,126315	0,563014691	0,503356606	-0,188392599	PDC	phosducin	ein-coding	th protein	G00000116703
401285	0,009034616	0,01721996	0,167677438	0,5596798	5,458543419	4,898863636	0,074534	0,426881908	0,821411982	0,029427933	TCP10L2	mplex 10-li	ein-coding	th protein	G00000166984
84769	0,020492389	0,01722521	0,167596816	0,7150866	8,834053077	8,11896645	0,021441	0,432756669	0,266220261	0,216673605	MPV17L2	inner me	ein-coding	th protein	G00000254858
64940	0,003824079	0,01725069	0,167581438	0,8680085	8,321054964	7,453046428	0,110429	0,245610269	0,074425027	0,368869348	STAG3L4	en 3-like 4	seudogeno	seudogen	G00000106610
30061	0,01943962	0,0173149	0,167941803	-1,346539	3,848702558	5,195241351	0,189937	-0,655056396	0,338889207	0,268305776	SLC40A1	er family 4	ein-coding	th protein	G00000138449
246184	0,006453853	0,01733214	0,167977478	-0,900191	9,286015723	10,18620672	0,252515	-0,306071961	0,816052441	-0,036371729	CDC26	division cyc	ein-coding	th protein	G00000176386
27129	0,013859716	0,01734092	0,167931147	0,6950786	4,785489232	4,090410667	0,16592	0,667210991	0,537958209	-0,23704825	HSPB7	n family B	ein-coding	th protein	G00000173641
79633	0,020652744	0,01734815	0,167869908	-0,9351	7,321038203	8,256138181	0,138876	-0,432435515	0,023733404	-0,70886701	FAT4	ypical cadh	ein-coding	th protein	G00000196159
647024	0,01668691	0,01743304	0,168428184	0,5496178	8,738582667	8,188964884	0,137795	0,34843284	0,466798624	0,095987412	C6orf132	open rea	ein-coding	th protein	G00000188112
151963	0,009855068	0,01746069	0,168563998	-0,849141	5,760341573	6,609482277	0,168317	-0,425034039	0,925438688	0,022175184	MB21D2	omain con	ein-coding	th protein	G00000180611
85315	0,010390514	0,01748567	0,168542243	-0,746007	4,761804469	5,507811154	0,277608	-0,237155829	0,674946012	0,057773458	PAQR8	oQ recept	ein-coding	th protein	G00000170915
27131	0,019061506	0,01756545	0,169179638	-0,570459	9,442967065	10,01342578	0,301833	-0,184709961	0,838576684	-0,0373525	SNX5	rting nexin	ein-coding	th protein	G00000089006
51076	0,021754231	0,01757691	0,169158465	0,5758315	9,215877619	8,640046105	0,029699	0,276308563	0,071027593	0,322481654	CUTC	opper tran	ein-coding	th protein	G00000119929
400684	0,003229335	0,01760118	0,169129202	0,8742399	7,39905062	6,524810684	0,514738	-0,137727448	0,026729031	0,551737404					
55164	0,021770862	0,01773644	0,169770364	-0,631657	6,363179734	6,994837159	0,200171	-0,506055427	0,127830783	0,261041818	SHQ1	ucleoprot	ein-coding	th protein	G00000144736
553	0,003375427	0,01779048	0,169893755	0,5487457	5,24413035	4,695384684	0,041111	0,785474793	0,38745883	-0,204496485	AVPR1B	sopressin r	ein-coding	th protein	G00000198049
5420	0,017959561	0,01783664	0,170203307	-0,618014	8,994443733	9,612457425	0,006423	-0,504069646	0,572974333	0,06715902	PODXL	docalyxin l	ein-coding	th protein	G00000128567
29916	0,00843029	0,01784464	0,17014859	0,5674271	7,319245244	6,751818138	0,938907	0,007535017	0,030447803	0,17885233	SNX11	rting nexin	ein-coding	th protein	G00000002919
650368	0,01777073	0,01784609	0,170031526	0,5050894	9,213011419	8,707922024	0,156159	0,190499363	0,315535419	0,151819162	TSSC2	ansferable	seudogeno	seudogen	G00000223756
54885	0,021970654	0,01795014	0,170760185	-0,977294	4,755094973	5,732388909	0,185555	-0,649066112	0,447173076	-0,20562505	TBC1D8B	ain family r	ein-coding	th protein	G00000133138
3658	0,007535797	0,01798014	0,170783249	-0,968313	7,071786073	8,040099565	0,038775	-0,653714923	0,870851945	-0,03036911	IREB2	element b	ein-coding	th protein	G00000136381
5861	0,003814321	0,01802311	0,170929211	0,5433278	10,93727537	10,39387537	0,259103	0,187686551	0,097174783	0,212494604	RAB1A	ber RAS on	ein-coding	th protein	G00000138069
100128364	0,02204585	0,01812549	0,171637268	-0,787853	9,122578011	9,910430739	0,514186	-0,066984403	0,037671651	-0,550459107					
6482	0,016121751	0,01812683	0,171518866	0,5402628	7,503940292	6,96367751	0,242044	0,167429051	0,616926842	-0,070402117	ST3GAL1	e alpha-2,	ein-coding	th protein	G00000008513
152559	0,007229716	0,01820588	0,171742004	0,6266579	7,077312855	6,450654983	0,397841	0,146062592	0,000830767	0,459725478	PAQR3	oQ recept	ein-coding	th protein	G00000163291
113246	0,005120161	0,01823675	0,171902366	0,6274336	11,78895821	11,16152459	0,017086	0,567856023	0,849232544	0,02961681	C12orf57	2 open rea	ein-coding	th protein	G00000111678

2819	0,022352865	0,01827872	0,172036066	0,686434	4,837106311	4,150672356	0,814441	-0,047045517	0,045577544	0,591861123	GPD1	sphate del	ein-coding	th protein	G00000167588
64374	0,022143288	0,01828507	0,171965154	0,5355834	9,65440222	9,118818783	0,07094	0,199450229	0,125958044	0,244659299	SIL1	otide excha	ein-coding	th protein	G00000120725
9023	0,005685877	0,01833556	0,17230919	1,3648873	8,347045412	6,982158161	0,012098	1,283851421	0,570122436	0,189758564	CH25H	rol 25-hyd	ein-coding	th protein	G00000138135
9823	0,008567654	0,0183553	0,172363939	0,7391904	6,670318691	5,931128333	0,019045	0,466694689	0,19289348	-0,117324361	ARMCX2	eat contain	ein-coding	th protein	G00000184867
6422	0,022121677	0,01837098	0,172380448	0,6999122	4,395650586	3,695738393	0,641119	0,143265313	0,221362907	0,35762048	SFRP1	zzled relat	ein-coding	th protein	G00000104332
84223	0,017760357	0,01840711	0,172458201	-0,797956	5,221368287	6,019324136	0,038664	-0,630091336	0,426435787	-0,171999672	IQCG	otif contair	ein-coding	th protein	G00000114473
144608	0,022121688	0,01854588	0,172973277	-0,907296	4,351931878	5,259228144	0,186905	-0,645134462	0,806447092	0,075224512	C12orf60	12 open rea	ein-coding	th protein	G00000182993
4673	0,011589828	0,01859079	0,173261685	-0,632522	11,87653183	12,5090539	0,051036	-0,620900426	0,067798711	-0,267317459	NAP1L1	assembly p	ein-coding	th protein	G00000187109
3535	0,015230623	0,01860031	0,173220103	2,7667993	3,131535833	0,364736518	0,294819	1,567795632	0,262562248	1,706088466	IGL	lobulin lam	ein-coding	th protein	product
833	0,006162063	0,01867198	0,173366087	0,5158682	9,393011114	8,877142868	0,004709	0,327137986	0,165333709	0,172252762	CARS	l-tRNA syn	ein-coding	th protein	G00000110619
6038	0,015658356	0,01871751	0,17365869	0,823305	8,510614041	7,687309049	0,062035	0,728800585	0,358291729	0,192915143	RNASE4	se A family	ein-coding	th protein	G00000258818
54790	0,022502728	0,01872678	0,173484744	-0,90717	5,592199776	6,499369973	0,098499	-0,548249253	0,84431087	-0,037495629	TET2	ytosine dic	ein-coding	th protein	G00000168769
85009	0,014331647	0,01880599	0,174088382	-3,533742	0,685101087	4,218843166	0,177599	-2,318468566	0,429738612	0,38206441					
310	0,023093006	0,01893558	0,174505506	0,5642618	10,29354941	9,729287606	0,158802	0,22506603	0,124495179	0,252008198	ANXA7	annexin A7	ein-coding	th protein	G00000138279
116512	0,009726172	0,01899204	0,174635996	1,086395	4,963070321	3,876675273	0,661884	0,081896113	0,114374186	0,51095999	MRGPRD	GPR famil	ein-coding	th protein	G00000172938
84191	0,022605121	0,019034	0,17476232	-0,769409	10,09901637	10,86842576	0,187352	-0,376219691	0,0404768	-0,751247884	FAM96A	ence simila	ein-coding	th protein	G00000166797
836	0,022701207	0,01904413	0,174725783	-0,794274	6,443815287	7,238089102	0,279705	-0,596848147	0,755229928	0,056538294	CASP3	caspase 3	ein-coding	th protein	G00000164305
29925	0,01024099	0,01907014	0,174834967	0,7569017	9,081327192	8,324425461	0,013677	0,494273013	0,078641631	0,406761799	GMPPB	se pyropho	ein-coding	th protein	G00000173540
137392	0,01498562	0,01912703	0,175097305	-0,535214	8,4611445	8,996358019	0,141377	-0,2608058	0,54614647	-0,103452062	FAM92A1	nce similar	ein-coding	th protein	G00000188343
445328	0,023163418	0,01916697	0,17533338	-1,077185	7,527947872	8,605132991	0,033712	-0,55328866	0,071636618	-0,59950417	ARHGEF35	leotide exe	in-coding	th protein	G00000213214
8503	0,017118668	0,01920997	0,175467478	0,8955837	6,172539472	5,276955787	0,380046	-0,616579092	0,007952928	0,770205093	PIK3R3	3-kinase re	in-coding	th protein	G00000117461
55660	0,008039765	0,01923399	0,17555743	-0,823082	10,22976049	11,05284282	0,09255	-0,314662213	0,853378626	-0,034987904	PRPF40A	essing facto	ein-coding	th protein	G00000196504
5373	0,009250695	0,01925784	0,17538737	1,0354912	9,411393566	8,375902379	0,109399	0,462162175	0,019015214	0,458779063	PMM2	omannom	ein-coding	th protein	G00000140650
6396	0,020477928	0,01928715	0,175396405	0,5563931	10,67063042	10,11423731	0,653682	0,054550078	0,097645094	0,301855157	SEC13	re and COF	ein-coding	th protein	G00000157020
5368	0,02195694	0,019307	0,175448108	0,5393076	5,058136212	4,518828619	0,392595	0,451799066	0,894752891	0,021155446	PNOC	pronocicep	in-coding	th protein	G00000168081
100216546	0,023484027	0,01937439	0,175931408	-0,662434	5,4169089	6,07934329	0,033228	-0,351047607	0,350271944	0,152810293	LINC01004	on-protein	n-coding R	long non-c	G00000228393
6549	0,016706894	0,01937952	0,175849063	1,2607406	4,145260905	2,884520259	0,059431	0,580383827	0,027647488	0,665167333	SLC9A2	er family 9	ein-coding	th protein	G00000115616
3868	0,014145832	0,01946693	0,176126099	0,57953	6,165598015	5,586068063	0,177737	0,332316108	0,799383171	-0,05045474	KRT16	keratin 16	ein-coding	th protein	G00000186832
56950	0,004586459	0,01950424	0,176077775	-0,675825	8,754406062	9,430230887	0,036185	-0,396070744	0,033802432	-0,407123391	SMYD2	ND domain	ein-coding	th protein	G00000143499
57447	0,013494397	0,01952358	0,175995843	-0,645598	5,722167844	6,367766086	0,185221	-0,220427294	0,182935681	-0,164095503	NDRG2	family mer	ein-coding	th protein	G00000165795
55319	0,023394147	0,01954183	0,175776559	0,5328949	9,370315425	8,837420526	0,021815	0,334626365	0,133661625	0,225915661	TMA16	nery assoc	ein-coding	th protein	G00000198498
113675	0,018722347	0,01955215	0,1757418	0,5265349	8,215835507	7,689300558	0,156206	0,168727026	0,121765346	0,258505854	SDSL	dehydrata	ein-coding	th protein	G00000139410
3151	0,013321303	0,01955536	0,175643129	-0,565731	12,37921924	12,94494984	0,118004	-1,118035233	0,201787179	-0,230130427	HMGN2	nucleosom	ein-coding	th protein	G00000198830
1459	0,011362057	0,0196034	0,1756924	0,5440887	9,19786224	8,65377351	0,2601	0,101827906	0,507973072	-0,08185725	CSNK2A2	kinase 2 a	ein-coding	th protein	G00000070770
1048	0,023790971	0,0196173	0,175436182	0,5010974	5,042057868	4,540960478	0,284189	0,59200565	0,931814484	-0,037230589	CEACAM5	en related	ein-coding	th protein	G00000105388
2218	0,020738108	0,01964166	0,175527289	-0,703796	6,866710419	7,570506598	0,09363	-0,43586231	0,410329513	-0,153632512	FKTN	fukutin	ein-coding	th protein	G00000106692
347730	0,006855877	0,019666	0,17561813	0,556645	6,101443166	5,544798172	0,561338	0,203541754	0,762934121	0,101695196	LRRTM1	t transmem	ein-coding	th protein	G00000162951
4653	0,023086727	0,01971892	0,175584315	1,5216995	3,446682016	1,924982565	0,265452	1,153922938	0,293127035	-0,405112305	MYOC	myocilin	ein-coding	th protein	G00000034971
6183	0,022198466	0,01973907	0,17551137	0,5286145	8,514203148	7,985588654	0,821996	0,021924662	0,052219782	0,325976893	MRPS12	l ribosoma	ein-coding	th protein	G00000128626
284029	0,018753956	0,01976119	0,175582047	0,8996187	6,48298436	5,583365612	0,009038	0,718855506	0,896082473	0,022846688	LINC00324	on-protein	n-coding R	long non-c	G00000178977
2115	0,01104063	0,01976806	0,175517106	0,6125392	7,421770794	6,809231581	0,030807	0,406446756	0,328024975	0,125093388	ETV1	TS variant	ein-coding	th protein	G00000006468
127833	0,012784638	0,01979738	0,175651554	0,6036322	6,459051595	5,85541936	0,659273	0,197531651	0,286876078	0,190773202	SYT2	aptotagmi	ein-coding	th protein	G00000143858
1310	0,007049154	0,01990017	0,176184912	0,8997939	4,493305212	3,593511358	0,415955	0,150253562	0,089259537	0,415492661	COL19A1	ype XIX alp	ein-coding	th protein	G00000082293
63036	0,02008732	0,01990499	0,176101699	0,5807456	6,108050905	5,527305291	0,028301	0,427412088	0,596279055	-0,069658226	CELA2A	elastase f	ein-coding	th protein	G00000142615
92086	0,006294467	0,01993849	0,17627218	1,2352077	4,558852552	3,323644821	0,791569	-0,241111593	0,290731247	1,124246489	GGTLC1	yltransfera	ein-coding	th protein	G00000149435
254170	0,013080147	0,01998787	0,176331145	0,6193897	8,640607771	8,021218035	0,111016	0,305971051	0,024740757	0,280000181	FBXO33	ox protein	ein-coding	th protein	G00000165355
202051	0,010741656	0,02000547	0,17636079	0,5439368	7,479306688	6,935369883	0,11051	0,123385341	0,699809712	-0,046653828	SPATA24	genesis ass	ein-coding	th protein	G00000170469
58189	0,021211224	0,02003526	0,176372399	0,7146499	5,208786246	4,494136352	0,347901	-0,414129784	0,169677148	0,690300925	WFDC1	isulfide co	ein-coding	th protein	G00000103175
29098	0,00567706	0,0200642	0,176376409	0,6098581	10,57593725	9,966079153	0,11702	0,21726888	0,417235965	0,116965448	RANGRF	nucleotide	ein-coding	th protein	G00000108961
401233	0,022093243	0,02009904	0,17643227	-0,65741	8,360332108	9,017741632	0,205588	-0,211480026	0,718630793	0,067458279	HTATSF1P2	fic factor 1	pseudogen	oseudogen	G00000271361
9854	0,004664863	0,02012835	0,1765644	0,5792587	7,554103854	6,974845123	0,005378	0,377349815	0,113011353	0,218216306	C2CD2L	C2CD2 like	ein-coding	th protein	G00000172375
219537	0,011091599	0,02016211	0,176735364	0,7951777	5,40208615	4,606908499	0,346379	0,217231534	0,204953428	0,27253067	SMTNL1	oothelin lik	ein-coding	th protein	G00000214872
55536	0,011250463	0,02016599	0,176644379	-0,719127	8,731732198	9,450859622	0,034367	-0,46535952	0,343985386	-0,085489239	CDCA7L	cycle asso	ein-coding	th protein	G00000164649
51605	0,024003384	0,02019122	0,176740404	0,5732383	9,367982505	8,794744188	0,023651	0,382148367	0,174770232	0,213188129	TRMT6	ethyltrans	ein-coding	th protein	G00000089195
648691	0,022706813	0,02019264	0,176627995	-0,637705	5,381135915	6,018840799	0,882907	-0,072071756	0,196775549	-0,505423907					
644656	0,018842436	0,02019378	0,176388792	0,7853134	8,295849649	7,510536289	0,955118	0,009268647	0,146010893	0,366552468					
115273	0,004893178	0,02022753	0,176434799	-0,738851	6,626720745	7,365571451	0,886848	-0,022222392	0,027054862	0,22984433	RAB42	ber RAS onc	ein-coding	th protein	G00000188060
644975	0,023536358	0,02029333	0,176387619	0,7077278	7,277331109	6,569603292	0,209103	0,463567013	0,708009125	0,076492812					
64087	0,015996591	0,02030063	0,176327348	-0,63857	7,262597412	7,901167808	0,023359	-0,43727746	0,127323119	0,311745456	MCCC2	noyl-CoA c	ein-coding	th protein	G00000131844

751071	0,015650762	0,0203275	0,176313466	0,7579277	8,005809063	7,247881331	0,041411	0,598739341	0,441013933	0,137808356	METTL12	transferase	ein-coding	th protein	G00000214756
9245	0,014306313	0,02036265	0,176125	0,5051132	4,821826464	4,316713286	0,553963	0,396064158	0,340604222	-0,533114237	GCNT3	etyl) transf	ein-coding	th protein	G00000140297
1413	0,008691863	0,02047914	0,176762228	0,9990251	4,183270164	3,184245061	0,305675	0,672527524	0,900098892	0,068327865	CRYBA4	stallin beta	ein-coding	th protein	G00000196431
253782	0,01295945	0,02048183	0,176662359	-0,608407	7,074287685	7,682694515	0,049775	-0,520676487	0,049052463	0,23324743	CERS6	mide synth	ein-coding	th protein	G00000172292
23596	0,007592884	0,02050788	0,176763956	-0,849816	6,618518189	7,468334672	0,620293	-0,081263444	0,002867475	-0,642122486	OPN3	opsin 3	ein-coding	th protein	G00000054277
57494	0,011731817	0,02052675	0,176557959	-0,517293	5,650283068	6,167576387	0,107676	-0,608220565	0,092568287	0,270788394	RIMKLB	protein rin	ein-coding	th protein	G00000166532
7576	0,024308868	0,02054599	0,176600809	-1,210331	5,259392838	6,46972406	0,167156	-0,243155783	0,374652377	-0,243927566	ZNF28	inger prote	ein-coding	th protein	G00000198538
4338	0,005952847	0,02054865	0,176501197	-0,587943	9,220770014	9,808712755	0,088347	-0,245996204	0,662926334	0,02380756	MOCS2	m cofactor	ein-coding	th protein	G00000164172
4189	0,024766322	0,02059744	0,176552979	1,4255543	9,520390324	8,094836002	0,054605	0,960781834	0,294498938	0,376424366	DNAJB9	tein family	ein-coding	th protein	G00000128590
23262	0,009729482	0,02062354	0,176410464	-0,739001	7,907152659	8,646153258	0,046875	-0,371038945	0,496619126	-0,112656917	PPIP5K2	pentakisp	ein-coding	th protein	G00000145725
3884	0,025005509	0,02064423	0,176465555	0,7105765	5,834949352	5,124372802	0,768328	0,072000635	0,08575385	0,424059733	KRT33B	keratin 33E	ein-coding	th protein	G00000131738
285958	0,007112308	0,02078902	0,177336103	0,6567143	10,82107813	10,16436383	0,553889	0,171038275	0,227242879	0,190071878	SNHG15	olar RNA h	n-coding R	long non-c	G00000232956
54863	0,024806974	0,0208672	0,177880492	0,5559943	8,759457019	8,203462678	0,588473	0,181898119	0,326964337	0,130540544	TOR4A	amily 4 me	ein-coding	th protein	G00000198113
6500	0,023938098	0,02089548	0,177999066	-1,246775	9,423293891	10,67006895	0,319906	-0,529278346	0,955932361	-0,025122474	SKP1	se-associat	ein-coding	th protein	G00000113558
56652	0,025230817	0,02091991	0,178084621	0,5102827	7,715020012	7,204737345	0,458674	0,087606069	0,246834913	0,15892582	C10orf2	10 open re	ein-coding	th protein	G00000107815
353141	0,018219316	0,02093971	0,178130789	0,9521342	5,859347008	4,907212768	0,01529	1,025434506	0,778321095	-0,08228521	LCE2D	nified enve	ein-coding	th protein	G00000187223
129401	0,019782954	0,02098816	0,17842043	0,6927876	8,27777934	7,584991758	0,016129	0,276918881	0,033144189	0,385687135	NUP35	ucleoporin	ein-coding	th protein	G00000163002
5578	0,017328081	0,02101099	0,178247481	0,6396873	9,01008943	8,370402086	0,09036	0,305416541	0,22507291	0,245900966	PRKCA	in kinase C	ein-coding	th protein	G00000154229
8535	0,024398757	0,0211484	0,179045321	0,8653781	7,412703957	6,547325852	0,007815	0,421570658	0,404374434	0,156441855	CBX4	hromobox	ein-coding	th protein	G00000141582
10455	0,004857701	0,02120241	0,179135179	-0,77426	10,68300052	11,45726071	0,072023	-0,317949022	0,115382806	-0,097855341	ECI2	A delta iso	ein-coding	th protein	G00000198721
202865	0,022173233	0,02121836	0,17902573	0,9350351	4,08181543	3,146780325	0,223627	0,723665993	0,30424676	0,578505257	C7orf33	7 open rea	ein-coding	th protein	G00000170279
2274	0,008836525	0,02127129	0,17935012	0,9707414	8,849264021	7,878522611	0,257554	0,341408359	0,950187556	-0,014285262	FHL2	a half LIM c	ein-coding	th protein	G00000115641
65083	0,023881192	0,02130039	0,179229445	0,5795949	8,20108189	7,621486984	0,338443	0,199463938	0,517920838	0,103889373	NOL6	eolar prote	ein-coding	th protein	G00000165271
219347	0,019315026	0,02131538	0,179112282	-0,842073	4,32193115	5,16400368	0,244866	-0,854707596	0,137506533	-0,398924361	MEM254-A	54 antisens	n-coding R	long non-c	G00000230091
360	0,021372535	0,02132505	0,179072043	0,7629964	7,062738676	6,299742311	0,206103	0,456624047	0,101969602	-0,422104634	AQP3	3 (Gill blo	ein-coding	th protein	G00000165272
126205	0,018614259	0,02134925	0,179032445	1,8077241	2,71864589	0,910921802	0,9122	-0,158832914	0,403995843	1,199557767	NLRP8	rin domair	ein-coding	th protein	G00000179709
5818	0,007447791	0,02142649	0,179437207	0,8844969	7,949709155	7,065212243	0,086647	0,318012557	0,371841943	0,172810363	NECTIN1	adhesion r	ein-coding	th protein	G00000110400
84909	0,009390644	0,0214576	0,179576374	0,644439	7,634342309	6,989903304	0,669795	0,116855322	0,900315712	-0,020378214	C9orf3	9 open rea	ein-coding	th protein	G00000148120
645249	0,02104269	0,02147861	0,179630801	0,6966175	6,66238983	5,965772311	0,790046	0,049764921	0,215107408	0,260997624	MNX1-AS1	se RNA 1 (	n-coding R	long non-c	G00000243479
5567	0,02402144	0,02150091	0,179695938	-0,861316	6,968130167	7,829446351	0,177116	-0,520552178	0,4937541	-0,19361349	PRKACB	-activated	ein-coding	th protein	G00000142875
79963	0,008593988	0,02151568	0,179698158	-0,671229	6,49660858	7,167837308	0,383506	-0,131426283	0,003107112	-0,343664507	ABCA11P	bfamily A n	pseudogen	pseudogen	G00000251595
10247	0,019379063	0,02152847	0,179562786	-0,624329	7,842615355	8,466944193	0,486123	-0,095790762	0,239772916	-0,140867216	RIDA	te imine de	ein-coding	th protein	G00000132541
6683	0,019766703	0,0217357	0,180804223	-0,767597	6,478984815	7,246581352	0,156374	-0,589382135	0,149437254	-0,248597922	SPAST	spastin	ein-coding	th protein	G00000021574
8445	0,017264243	0,02174866	0,180427328	0,6365829	8,210311764	7,573728831	0,002315	0,752231878	0,959986878	-0,007898098	DYRK2	phosphory	ein-coding	th protein	G00000127334
162963	0,022448851	0,02176639	0,180453605	-0,555806	4,966587552	5,522393941	0,735705	0,021736518	0,634856737	-0,068337578	ZNF610	nger prote	ein-coding	th protein	G00000167554
56265	0,006270414	0,02179398	0,180561479	0,5505595	5,667549489	5,116990007	0,150978	0,488077876	0,959320028	0,006246086	CPXM1	se X, M14 f	ein-coding	th protein	G00000088882
5676	0,02595805	0,02182167	0,180549349	-2,012649	5,555343097	7,567991809	0,443054	-0,264606812	0,284236414	-0,607225382	PSG7	L-glycoprot	ein-coding	th protein	G00000221878
6772	0,006032624	0,02205816	0,181174695	-0,612027	8,146640044	8,758667122	0,443862	-0,09981316	0,028766104	-0,49096217	STAT1	nd activatc	ein-coding	th protein	G00000115415
55012	0,007972764	0,02206912	0,181144672	0,6884143	9,907611668	9,219197408	0,066713	0,230157207	0,245427	0,196587582	PPP2R3C	2 regulato	ein-coding	th protein	G00000092020
89894	0,020946889	0,02213419	0,181198409	-0,618255	6,849995597	7,468250291	0,953079	0,007852286	0,966340349	-0,00621246	TMEM116	mbrane pr	ein-coding	th protein	G00000198270
1499	0,017252497	0,02226584	0,182035498	0,9348765	10,60835732	9,673480775	0,045131	0,450026235	0,474878912	0,168239267	CTNNB1	atenin beta	ein-coding	th protein	G00000168036
152789	0,013321118	0,0222792	0,182024637	0,6845521	3,849722035	3,165169912	0,469602	0,23726166	0,055440123	0,586736749	JAKMIP1	crotubule i	ein-coding	th protein	G00000152969
259	0,012935391	0,02228297	0,181935359	0,6879264	5,294727819	4,606801443	0,597618	0,159588313	0,361397773	0,288899149	AMBP	globulin/bik	ein-coding	th protein	G00000106927
912	0,00479068	0,02233865	0,182149886	1,9103273	4,065996413	2,155669139	0,177362	1,189765804	0,791743017	0,188318651	CD1D	D1d molecu	ein-coding	th protein	G00000158473
5651	0,023094214	0,02240424	0,182564456	1,4500227	1,867388639	0,417365896	0,082359	1,671452149	0,79079347	0,181922433	TMPRSS15	ane protea	ein-coding	th protein	G00000154646
389123	0,020573524	0,02241705	0,182548796	1,0339074	3,495635212	2,46172785	0,230217	1,340427808	0,384701022	-0,877657932	IQCF2	otif contain	ein-coding	th protein	G00000184345
85025	0,008028975	0,02244683	0,182551234	-0,681085	8,144599503	8,825684307	0,172595	-0,259987121	0,024626003	0,237477238	TMEM60	mbrane pr	ein-coding	th protein	G00000135211
9240	0,011358114	0,02245889	0,182529468	0,753571	9,807707025	9,054135989	0,508938	0,156762816	0,07908072	0,426471027	PNMA1	plastic Ma	ein-coding	th protein	G00000176903
100528007	0,005461564	0,02245938	0,182413753	-0,55423	5,599223864	6,153454339	0,227695	-0,24127124	0,528446035	-0,073578652	ORCS7-ASN	adthrough	other	eadthroug	G00000270316
284293	0,023730923	0,0224607	0,182304908	0,5711292	8,647084277	8,075955064	0,048444	0,257500716	0,33260443	-0,126449681	HMSD	minor serpi	ein-coding	th protein	G00000221887
160140	0,00561259	0,02256201	0,1827679	-0,967316	3,91424612	4,881562002	0,065859	-0,335643296	0,568393793	0,049508394	C11orf65	11 open rea	ein-coding	th protein	G00000166323
448834	0,006159214	0,02257118	0,182722746	2,388912	3,815373525	1,426461477	0,063284	2,045228566	0,797499235	0,177720447	KPRP	te proline r	ein-coding	th protein	G00000203786
279	0,025196241	0,02257125	0,182603956	-0,848799	5,048876554	5,897675517	0,037546	-0,515759957	0,445456361	-0,186450222	AMY2A	alpha 2A (p	ein-coding	th protein	G00000243480
11146	0,020227043	0,02261512	0,182601018	-0,598594	7,85462903	8,453222993	0,021795	-0,479414862	0,474111262	-0,093773072	GLMN	KBP associ	ein-coding	th protein	G00000174842
116984	0,026833447	0,02266291	0,182748652	-0,85678	4,638996272	5,495776412	0,057564	-0,656204124	0,659895478	0,103321647	ARAP2	ain, ankyri	ein-coding	th protein	G00000047365
497258	0,015097052	0,02267432	0,18260286	-1,212068	3,857796956	5,069865407	0,242513	-0,68252326	0,690837633	-0,063074352	BDNF-AS	F antisense	n-coding R	long non-c	G00000245573
23240	0,025749571	0,02268058	0,182534575	-0,776237	6,181816016	6,958052771	0,107381	-0,369542146	0,89361448	-0,021016051	KIAA0922	KIAA0922	ein-coding	th protein	G00000121210
134701	0,005565536	0,02272366	0,182525485	0,6245704	5,129999257	4,505428891	0,043099	0,846214345	0,610641595	-0,039887017	RIPPLY2	scriptional	ein-coding	th protein	G00000203877
284071	0,01751242	0,02272475	0,182415947	0,8439296	4,785914372	3,941984745	0,360235	0,587148464	0,904320926	-0,058968354	MEIOC	ic with coil	ein-coding	th protein	G00000180336

140690	0,015840643	0,02273176	0,182236004	0,5732587	5,674945013	5,101686331	0,280073	0,399549606	0,886683228	-0,044647153	CTCFL	binding fac	ein-coding	th protein	G00000124092
7334	0,012114905	0,02273353	0,182132364	0,5708628	9,014224697	8,443361923	0,279093	0,123068244	0,048541643	0,399949174	UBE2N	njugating e	ein-coding	th protein	G00000177889
146513	0,004902552	0,02277854	0,182022026	-3,3262	0	3,326200381	0,1841	-2,369701646	0,518066048	-0,956498735					
10384	0,024997369	0,02277982	0,181914887	-0,662367	7,080064475	7,742431357	0,530409	-0,197639864	0,092243939	-0,393464629	BTN3A3	subfamily 3	ein-coding	th protein	G00000111801
644852	0,016810293	0,0228222	0,182018594	0,8948424	2,757471684	1,862629329	0,261403	0,793061257	0,068355465	0,306897963					
22977	0,014413516	0,02285199	0,182138934	-0,831633	7,5675388	8,399172197	0,078285	-0,595608982	0,64620338	-0,055114665	AKR7A3	ctase famil	ein-coding	th protein	G00000162482
84288	0,023057087	0,0229282	0,182394207	0,546188	6,220371319	5,674183296	0,457677	-0,29651413	0,037478884	0,4759878	EFCAB2	cium bindi	ein-coding	th protein	G00000203666
1152	0,023098735	0,02294821	0,182319227	0,710959	8,757063367	8,046104365	0,401567	0,251930464	0,105688686	0,398808882	CKB	atine kinas	ein-coding	th protein	G00000166165
401250	0,023120316	0,02298871	0,18252388	0,5197349	6,694665537	6,17493064	0,068303	0,639364686	0,233230011	-0,185083991	MCCD1	ial coiled-c	ein-coding	th protein	G00000204511
389860	0,010857747	0,02307109	0,182826395	-1,859938	3,722787898	5,582725989	0,649278	-0,139120411	0,186794431	-0,427717902	PAGE2B	amily mem	ein-coding	th protein	G00000238269
129607	0,019386187	0,02310516	0,182862393	0,747104	4,752734861	4,005630837	0,464246	0,427664319	0,631068352	0,231201305	CMPK2	e monopho	ein-coding	th protein	G00000134326
387712	0,013513514	0,0231086	0,182772822	-0,77277	4,103476767	4,87624695	0,084735	-0,387891944	0,019046352	-0,441925463	ENO4	e family me	ein-coding	th protein	G00000188316
79981	0,025515764	0,02317704	0,182730647	0,7286974	5,197258759	4,468561355	0,092673	0,404704718	0,676834117	0,072302101	FRMD1	omain cont	ein-coding	th protein	G00000153303
388963	0,01455161	0,02322252	0,182740287	0,5459407	7,531526092	6,985585359	0,305174	0,261716555	0,10690382	-0,301409832	C2orf81	2 open rea	ein-coding	th protein	G00000159239
283897	0,023094661	0,02324026	0,18264778	0,6043851	4,968702182	4,364317065	0,45274	0,339768119	0,669709025	-0,181086935	C16orf54	16 open rea	ein-coding	th protein	G00000185905
54534	0,00513493	0,02326134	0,182697567	0,6289556	11,06336316	10,43440755	0,022633	0,414208145	0,193560732	0,167812885	MRPL50	al ribosoma	ein-coding	th protein	G00000136897
51109	0,0179677	0,02332515	0,1829667	1,1301469	10,02259276	8,8924459	0,002093	0,808578948	0,14001032	0,545376718	RDH11	nase 11 (all	ein-coding	th protein	G00000072042
913	0,015639562	0,02335926	0,183002428	0,5035383	5,437656094	4,934117771	0,261112	0,300245964	0,348959869	-0,105170759	CD1E	D1e molecu	ein-coding	th protein	G00000158488
79084	0,020364803	0,02336117	0,182901702	0,5439764	9,520924227	8,976947825	0,138026	0,135495746	0,110735736	0,293392204	WDR77	epeat doma	ein-coding	th protein	G00000116455
339291	0,026426559	0,02336467	0,182813582	0,6708472	6,966416589	6,295569406	0,024574	0,849612976	0,691922115	0,076931748	LRRC30	n repeat co	ein-coding	th protein	G00000206422
6777	0,018451039	0,02338784	0,182879329	0,6149757	8,235910846	7,620935177	0,512385	0,126899814	0,146430268	0,283492213	STAT5B	nd activato	ein-coding	th protein	G00000173757
148738	0,008086509	0,02343711	0,183033488	1,0364335	4,961823688	3,925390175	0,232587	0,518708485	0,478287527	0,269281432	HFE2	natosis type	ein-coding	th protein	G00000168509
333926	0,015866793	0,02344677	0,182993568	-0,557146	6,091368682	6,648514437	0,124896	-0,257022352	0,104168085	-0,310518725	PPM1J	se, Mg2+/N	ein-coding	th protein	G00000155367
3620	0,016314825	0,02354963	0,183680564	-1,544109	2,792270938	4,336379817	0,196661	-1,982988121	0,084288359	-0,484383021	IDO1	ne 2,3-diox	ein-coding	th protein	G00000131203
63901	0,022115906	0,02384894	0,184851087	-0,746773	5,897238704	6,644011345	0,02339	-0,591598985	0,348134583	0,199864408	FAM111A	nce similar	ein-coding	th protein	G00000166801
125476	0,017185326	0,0238829	0,184882897	0,687737	10,28603366	9,598296698	0,082213	0,415781576	0,417612626	0,178886785	INO80C	complex su	ein-coding	th protein	G00000153391
90167	0,028113029	0,02390336	0,184925721	1,788185	2,93466031	1,146475359	0,115738	1,578396639	0,893330256	0,105532475	FRMD7	omain cont	ein-coding	th protein	G00000165694
11221	0,018931207	0,02394301	0,185116829	-0,582	8,720487508	9,302487548	0,264671	0,156309597	0,019407802	-0,611143523	DUSP10	ficity phos	ein-coding	th protein	G00000143507
28978	0,015745055	0,02394842	0,184927775	-0,73324	9,85462924	10,58786954	0,000133	-0,433881984	0,220807354	-0,125692686	TMEM14A	mbrane pr	ein-coding	th protein	G00000096092
100131454	0,026688638	0,02395233	0,184842758	-1,621627	3,668435577	5,290062774	0,287036	-1,920295894	0,967948314	0,015929648	DBIL5P	inhibitor-li	pseudogene	pseudogene	G00000231784
1040	0,021480132	0,02405487	0,185518482	-0,894251	5,585732153	6,479982825	0,515197	-0,346607294	0,055076351	-0,709282682	CDS1	ylglycerol s	ein-coding	th protein	G00000163624
9496	0,021959604	0,02405897	0,1854346	0,5366319	5,268270725	4,731638824	0,295493	0,206782856	0,654857889	0,063646098	TBX4	T-box 4	ein-coding	th protein	G00000121075
25849	0,013305911	0,02410556	0,185678155	0,8456472	5,929459099	5,083811902	0,502554	0,125172791	0,029737947	0,301422931	PARM1	regulated r	ein-coding	th protein	G00000169116
81553	0,020470416	0,02416173	0,185879648	-0,581133	4,556702563	5,137835587	0,782168	-0,054036865	0,597070554	-0,089622891	FAM49A	ence simila	ein-coding	th protein	G00000197872
23285	0,028160582	0,02416428	0,185783887	-0,880547	5,463136779	6,343684004	0,471433	-0,188571967	0,266796453	-0,328001936	KIAA1107	KIAA1107	ein-coding	th protein	G00000069712
4103	0,026916829	0,02417379	0,185741728	1,948711	3,558741569	1,610030543	0,388185	1,090541727	0,92841888	-0,057895971	MAGEA4	family men	ein-coding	th protein	G00000147381
441733	0,00599062	0,02418142	0,185570072	-1,224862	6,359644069	7,584505701	0,004316	-1,072312185	0,090164999	0,318102342	PRKXP1	e, X-linked,	pseudogene	pseudogene	G00000259205
9455	0,027500942	0,02421671	0,185725838	-0,551988	5,584443173	6,136431388	0,051697	-0,481670346	0,990036137	0,002284803	HOMER2	caffolding p	ein-coding	th protein	G00000103942
221424	0,028435078	0,024244	0,185820045	0,6929374	6,782094649	6,089157292	0,130249	0,361574877	0,237677843	0,26467949	LRRC73	n repeat co	ein-coding	th protein	G00000204052
55742	0,011676774	0,0242459	0,185719715	-0,6216	8,774024833	9,395624977	0,381767	-0,205872743	0,276288884	0,138089543	PARVA	parvin alph	ein-coding	th protein	G00000197702
29947	0,028465176	0,02426002	0,185598328	-0,691659	5,030480931	5,722139837	0,898325	0,049184175	0,225629874	-0,259794951	DNMT3L	hyltransfer	ein-coding	th protein	G00000142182
90673	0,028470368	0,02438827	0,186119662	-0,884846	6,18513139	7,069976928	0,13824	-0,744851457	0,573622923	-0,125886585	PPP1R3E	tase 1 regu	ein-coding	th protein	G00000235194
23400	0,005967316	0,02441459	0,186205753	-0,71352	7,472039171	8,185559384	0,534901	-0,120068602	0,949444443	0,009620794	ATP13A2	ATPase 13A	ein-coding	th protein	G00000159363
171220	0,021542572	0,02442378	0,186161252	0,6598425	13,76713662	13,10729415	0,116006	0,489595472	0,492521999	-0,107792524	DSTNP2	ymerizing f	pseudogene	pseudogene	G00000248593
4207	0,011780306	0,02448234	0,186263676	0,7075427	8,739596231	8,032053554	0,045951	0,334706842	0,252046396	0,223199832	ORCS8-MEF	MEF2B rea	other	eadthroug	G00000064489
219972	0,006529432	0,02450183	0,186297567	1,5682898	4,100913712	2,5326239	0,733141	0,194620674	0,28731361	0,673579627	MPEG1	phage expr	ein-coding	th protein	G00000197629
10611	0,005638533	0,02450269	0,186189812	0,7553379	10,05678404	9,301446118	0,018467	0,376905464	0,194105511	-0,231167922	PDLIM5	nd LIM dor	ein-coding	th protein	G00000163110
5999	0,010037331	0,02453601	0,186328698	1,028811	7,849905039	6,821093994	0,303363	0,200967981	0,262247118	-0,127948541	RGS4	f G-protein	ein-coding	th protein	G00000117152
158401	0,018703647	0,02454506	0,186283181	-1,49487	2,91824865	4,413118915	0,565215	-0,415275614	0,039690773	-1,452164203	C9orf84	9 open rea	ein-coding	th protein	G00000165181
22864	0,02054738	0,02455371	0,186234693	-0,509681	7,376088353	7,885769387	0,02598	-0,275315047	0,198724577	-0,136504637	R3HDM2	main conta	ein-coding	th protein	G00000179912
5226	0,015151866	0,02460511	0,186396258	-0,651111	9,781812429	10,43292341	0,019692	-0,406916797	0,88254163	0,015106464	PGD	conate deh	ein-coding	th protein	G00000142657
55830	0,020045955	0,02468015	0,186736359	-0,5481	7,913536843	8,461636741	0,05821	-0,336483436	0,223337298	0,219152958	GLT8D1	rase 8 dom	ein-coding	th protein	G00000016864
84918	0,025949775	0,02468599	0,186666492	-0,602703	6,741855846	7,344558929	0,758259	-0,046752136	0,527240957	-0,085787311	LRP11	tor related	ein-coding	th protein	G00000120256
133746	0,024909977	0,02472267	0,186602097	0,6323144	7,945418796	7,313104428	0,389401	0,302046951	0,694202178	0,07989073	JMY	d regulator	ein-coding	th protein	G00000152409
9928	0,027591412	0,02474093	0,186626188	0,5575449	8,239936284	7,682391351	0,722407	0,062786965	0,021200762	0,47793985	KIF14	family mer	ein-coding	th protein	G00000118193
55207	0,028773542	0,02475356	0,186607779	0,6385366	10,40858803	9,770051417	0,066135	0,23656334	0,037034017	0,508750091	ARL8B	ion factor l	ein-coding	th protein	G00000134108
6383	0,014019063	0,02482896	0,186835047	1,0666238	7,775712492	6,709088669	0,098087	0,687715226	0,653157056	0,136451466	SDC2	syndecan 2	ein-coding	th protein	G00000169439
7278	0,029071701	0,02490035	0,18691805	-0,697272	4,001696244	4,698968048	0,985961	0,010311332	0,703944024	-0,200749334	TUBA3C	bulin alpha	ein-coding	th protein	G00000198033
7053	0,028709033	0,0249307	0,187032542	0,6915302	5,625286525	4,933756296	0,677068	0,113231486	0,273945572	0,253705456	TGM3	sglutamina	ein-coding	th protein	G00000125780

399904	0,026794425	0,02493467	0,186949087	0,7647663	4,352712511	3,587946197	0,268886	-0,763547665	0,167480996	0,63857335					
3108	0,009119679	0,02493752	0,186857345	-0,636201	10,7491108	11,38531172	0,055505	-0,241233707	0,021183535	-0,563405911	HLA-DMA	ility compl	ein-coding	th protein	G00000204257
149351	0,020484855	0,02497448	0,187021152	-0,82832	6,06588247	6,894202373	0,109392	-0,846967743	0,068494796	0,714754559					
4214	0,007909013	0,02500119	0,186882183	-0,990891	6,614676053	7,605566865	0,004056	-0,571708641	0,107041115	-0,464755085	MAP3K1	protein kin	ein-coding	th protein	G00000095015
54477	0,010258214	0,02505001	0,18702135	-0,555217	7,703975102	8,259191856	0,172614	-0,471919938	0,218564465	-0,182631368	PLEKHA5	logy doma	ein-coding	th protein	G00000052126
3077	0,008650388	0,02519915	0,187795235	-0,844918	6,756874368	7,601792617	0,064933	-0,285899167	0,042455845	-0,575718899	HFE	nochromat	ein-coding	th protein	G00000010704
7905	0,009886942	0,02521887	0,187829159	-0,514191	11,88591323	12,40010382	0,429089	-0,113288688	0,190182099	-0,233831257	REEP5	accessory	ein-coding	th protein	G00000129625
56344	0,021335639	0,02529255	0,188151702	2,8045231	3,167740605	0,363217459	0,921729	0,12106773	0,264073238	1,203852459	CABP5	binding p	ein-coding	th protein	G00000105507
54765	0,009121853	0,02529348	0,188045643	-0,584049	9,798036318	10,38208524	0,026185	-0,375005635	0,096837901	0,165712893	TRIM44	motif cont	ein-coding	th protein	G00000166326
729879	0,026467248	0,02533288	0,188112771	0,8529898	5,127805521	4,274815693	0,034517	0,751107023	0,334528471	0,251263528					
727738	0,028500496	0,02554305	0,188880718	0,6005997	10,71350684	10,11290711	0,108723	0,798101255	0,401371128	-0,268418758					
54474	0,016683793	0,02558778	0,188985842	0,5229892	4,033764088	3,510774843	0,005748	1,196800909	0,315652	-0,21271188	KRT20	keratin 20	ein-coding	th protein	G00000171431
26503	0,025836737	0,02559612	0,188934765	-0,731082	9,108769652	9,839851574	0,02192	-0,199722246	0,15506799	-0,352669302	SLC17A5	er family 1	ein-coding	th protein	G00000119899
653857	0,012090324	0,02561362	0,188838888	-0,637997	8,527146405	9,16514373	0,039087	-0,388523309	0,444833581	-0,128862806	ACTR3C	ated protei	ein-coding	th protein	G00000106526
55814	0,010061642	0,02562129	0,188783016	-0,612992	7,692207745	8,305200027	0,141152	-0,447618257	0,29326326	-0,164832437	BDP1	olymerase	ein-coding	th protein	G00000145734
257194	0,021578497	0,02568733	0,188932428	0,5962505	6,792826191	6,1965757	0,36465	0,270471592	0,71515718	-0,059425779	NEGR1	growth re	ein-coding	th protein	G00000172260
4175	0,01813791	0,02579233	0,1892552	-0,70696	8,571069287	9,278029162	0,038803	-0,360040018	0,70417479	-0,048897157	MCM6	intenance	ein-coding	th protein	G00000076003
7879	0,010380752	0,02580244	0,189217268	-0,74372	10,48379377	11,22751402	0,049991	-0,374111488	0,545562644	0,105992992	RAB7A	ber RAS on	ein-coding	th protein	G00000075785
54097	0,025067869	0,02587152	0,189499451	0,5703797	5,431397048	4,86101735	0,159658	0,599572579	0,88430794	-0,049553247	FAM3B	ence simila	ein-coding	th protein	G00000183844
652	0,017029641	0,02589182	0,189536075	-0,715318	10,27224784	10,98756579	0,243994	-0,10838004	0,00214391	-0,686401344	BMP4	phogenetic	ein-coding	th protein	G00000125378
3460	0,027243663	0,0259275	0,189573193	-0,783133	8,274105185	9,057238059	0,169961	-0,182741506	0,111711937	-0,43773375	IFNGR2	or 2 (interfe	ein-coding	th protein	G00000159128
171568	0,015087606	0,02596319	0,189722159	0,6797085	9,539674009	8,859965484	0,886007	0,017358379	0,802205647	-0,042057919	POLR3H	merase III	ein-coding	th protein	G00000100413
84645	0,018573444	0,02597572	0,189701786	-0,663441	4,642589079	5,306030056	0,062779	-1,346839825	0,911060265	0,02354662	C22orf23	22 open re	ein-coding	th protein	G00000128346
339263	0,00847374	0,02609673	0,190025338	0,5847325	9,567018419	8,982285931	0,164925	0,219997823	0,027998264	0,50637927	C17orf51	17 open re	ein-coding	th protein	G00000212719
730098	0,02612691	0,0261398	0,190115382	0,8026531	8,240500171	7,43784703	0,182929	0,239926966	0,909043599	0,026528533					
8516	0,014206103	0,02617748	0,190166121	1,6478344	1,984481482	0,336647077	0,002362	1,961148969	0,771817861	-0,124963436	ITGA8	n subunit a	ein-coding	th protein	G00000077943
705	0,012149108	0,02626803	0,190488778	0,7547729	10,51952914	9,764756228	0,091423	0,232126962	0,233644541	0,248629739	BYSL	bystin like	ein-coding	th protein	G00000112578
8334	0,027958472	0,0264158	0,191112745	-0,776013	8,040517247	8,816530137	0,536803	-0,272683025	0,125683807	-0,448866417	HIST1H2AC	r 1 H2A far	ein-coding	th protein	G00000180573
100128252	0,026742351	0,02644225	0,191080924	1,531298	4,776841725	3,245543767	0,036776	1,009636739	0,087831024	0,986302095	ZNF667-AS1	nse RNA 1	n-coding R	long non-c	G00000166770
11034	0,020150658	0,02645974	0,191095786	0,680191	13,11855417	12,43836313	0,346877	0,308253386	0,645593112	-0,099623615	DSTN	n depolyme	ein-coding	th protein	G00000125868
254187	0,027292497	0,02651322	0,191258972	0,6968728	6,05677786	5,359905055	0,260395	0,387395557	0,829862144	0,04054978	TSGA10IP	c 10 intera	ein-coding	th protein	G00000175513
5865	0,026669081	0,0265165	0,191171335	0,6095316	5,896681671	5,287150028	0,263631	0,328060706	0,37665233	0,113318541	RAB3B	ber RAS on	ein-coding	th protein	G00000169213
100131897	0,01072024	0,02651938	0,191080876	0,965038	5,197604702	4,232566663	0,248497	0,501456591	0,55679273	-0,213096406	FAM196B	nce similar	ein-coding	th protein	G00000204767
9630	0,014629757	0,02652618	0,191018747	1,1710504	4,285374508	3,114324123	0,025622	0,637718604	0,876428595	0,045053372	GNA14	in subunit a	ein-coding	th protein	G00000156049
56849	0,011687108	0,02652849	0,190924384	0,5173661	6,184596468	5,66723038	0,481943	0,2566827	0,748512413	0,110337954	TCEAL7	elongation	ein-coding	th protein	G00000182916
51293	0,014125402	0,02655105	0,190975747	0,5357306	11,46177627	10,92604568	0,231798	-0,288200045	0,02997722	0,598693893	CD320	320 molec	ein-coding	th protein	G00000167775
4100	0,030194085	0,02661262	0,191307506	0,8641941	5,068048629	4,203854538	0,106436	0,957784102	0,985943816	-0,007820105	MAGEA1	family men	ein-coding	th protein	G00000198681
10553	0,028539172	0,02662087	0,191255842	-0,660297	8,700144915	9,360441941	0,399715	-0,215112621	0,798716121	-0,054672992	HTATIP2	interactive	ein-coding	th protein	G00000109854
10215	0,029457407	0,02666959	0,191383852	2,3048334	2,727551561	0,42271813	0,004717	3,118075375	0,422649731	-0,42271813	OLIG2	ineage tran	ein-coding	th protein	G00000205927
112479	0,025770663	0,02667472	0,191309806	-0,806321	4,863166815	5,66948804	0,054994	-1,21816232	0,327802125	0,235361893	ERI2	uclease far	ein-coding	th protein	G00000196678
57590	0,02920778	0,02672509	0,191338689	-0,616272	7,774287799	8,390559846	0,040452	-0,326851153	0,086522231	-0,433907763	WDFY1	FYVE doma	ein-coding	th protein	G00000085449
729658	0,018298232	0,02675209	0,191421357	1,3876004	4,594658263	3,207057878	0,006353	1,207137878	0,973090337	-0,012493504	PACRG-AS3	antisense	n-coding R	long non-c	G00000225683
5189	0,02082392	0,02678898	0,191574636	-0,617636	6,570401838	7,188038117	0,311266	-0,362869541	0,734324601	0,032288968	PEX1	al biogene	ein-coding	th protein	G00000127980
88455	0,008903026	0,02681921	0,191680118	0,6086465	8,204747805	7,596101274	0,13521	0,306898158	0,28153531	-0,218033575	ANKRD13A	repeat don	ein-coding	th protein	G00000076513
6536	0,009866594	0,0268281	0,191633126	0,5722393	7,717657956	7,145418631	0,038045	0,463033645	0,521705269	0,093629008	SLC6A9	ier family 6	ein-coding	th protein	G00000196517
9641	0,008793886	0,02690155	0,191825855	-0,617244	7,548048638	8,165292594	0,112603	-0,212421744	0,128066129	-0,273837066	IKBKE	ide gene e	ein-coding	th protein	G00000263528
148213	0,013828123	0,02692717	0,191677541	-3,047114	2,954358502	6,001472964	0,110491	-1,797203395	0,837237534	0,061443979	ZNF681	nger prote	ein-coding	th protein	G00000196172
84176	0,011623378	0,02696135	0,191810586	-0,734474	5,203838136	5,938311869	0,284373	-0,200919929	0,076468251	-0,442667421	MYH16	y chain 16	seudogen	seudogen	G00000002079
90634	0,030996193	0,02697388	0,191789565	-0,788127	4,278071943	5,066199014	0,758839	-0,138432453	0,859024408	0,045857409	N4BP2L1	nding prote	ein-coding	th protein	G00000139597
29950	0,007295753	0,027003	0,19166653	0,6525649	8,494714341	7,842149423	0,002609	0,263386164	0,165127198	0,237934963	SERTAD1	omain con	ein-coding	th protein	G00000197019
7056	0,013067416	0,02702559	0,191716984	0,5371329	14,22567255	13,68853967	0,128227	0,302625559	0,001311283	-1,321008966	THBD	ombomod	ein-coding	th protein	G00000178726
81537	0,021047535	0,02711579	0,192136788	0,7167817	9,478139355	8,761357616	0,203076	0,232004737	0,162266306	0,334681728	SGPP1	phosphate	ein-coding	th protein	G00000126821
3119	0,030831838	0,02715165	0,192280858	-0,671854	5,406413102	6,078267513	0,320012	0,143858381	0,024852411	-0,607327974	HLA-DQB1	ility compl	ein-coding	th protein	G00000179344
343637	0,029739952	0,02716313	0,192252239	0,598426	6,923849407	6,325423439	0,634	0,141567405	0,203532265	0,243057767	RSPO4	R-spondin 4	ein-coding	th protein	G00000101282
2671	0,019212727	0,02728872	0,192700716	0,5028886	7,873123628	7,370235045	0,089153	0,30506816	0,22258446	0,223369306	GFER	gmenter of	ein-coding	th protein	G00000127554
26191	0,030752349	0,02735751	0,192746878	-1,280102	3,924579937	5,204682279	0,462998	0,098316349	0,129100839	-0,577489061	PTPN22	sphatase, r	ein-coding	th protein	G00000134242
3434	0,030949532	0,02736124	0,192663583	0,6601055	9,26142487	8,601319394	0,03363	0,639525424	0,387263068	0,200929564	IFIT1	ein with tet	ein-coding	th protein	G00000185745
83394	0,017829563	0,02737683	0,192554425	0,5467151	7,281164602	6,73444955	0,170064	0,237303711	0,440237001	0,133605059	PITPNM3	1 family me	ein-coding	th protein	G00000091622
140680	0,011384781	0,02740162	0,192619435	-0,588186	6,186498964	6,774685146	0,565764	-0,134792256	0,336565316	-0,191700018	C20orf96	20 open re	ein-coding	th protein	G00000196476

80757	0,007706035	0,02743803	0,192656651	0,8490121	7,525850495	6,676838436	0,231764	0,435896073	0,030223496	0,527800792	TMEM121	nbrane pr	ein-coding	th protein	G00000184986
55194	0,029441184	0,02746122	0,192492131	0,7617579	5,460462251	4,698704313	0,474244	0,343113399	0,220473802	0,388877212	EVA1B	ı-1 homolo	ein-coding	th protein	G00000142694
14	0,011045269	0,02753612	0,192798908	0,7301346	9,341192466	8,611057865	0,258381	0,162250891	0,182489647	0,274242264	AAMP	ed migrat	ein-coding	th protein	G00000127837
11027	0,009408732	0,02764014	0,193090614	0,6881926	5,956934248	5,268741662	0,01989	0,791531736	0,744808248	-0,062610264	LILRA2	noglobulin	ein-coding	th protein	G00000239998
59269	0,014785566	0,02764019	0,19298214	0,690456	6,809156818	6,118700812	0,842813	0,050983988	0,085356088	0,42381044	HIVEP3	virus type I	ein-coding	th protein	G00000127124
1290	0,02803983	0,02765314	0,192746582	0,5093827	4,67403588	4,164653181	0,084271	0,468100443	0,803204454	-0,050106921	COL5A2	type V alph	ein-coding	th protein	G00000204262
91452	0,00952228	0,02765618	0,192659397	-1,734473	8,172937656	9,907411098	0,239082	-0,5847528	0,230982121	-0,133975012	ACBD5	ling domai	ein-coding	th protein	G00000107897
51451	0,014240998	0,02768284	0,19262843	-0,653626	8,91092134	9,564546891	0,244746	-0,153660889	0,098095914	-0,38145544	LCMT1	oxyl methyl	ein-coding	th protein	G00000205629
4800	0,028125355	0,02769537	0,192607377	0,5329108	8,18244871	7,649537863	0,05317	0,346910014	0,261804396	0,191173797	NFYA	otion facto	ein-coding	th protein	G00000001167
6301	0,030313714	0,02772628	0,192714181	0,5391875	11,40561547	10,86642795	0,10735	0,306605961	0,053367239	0,362860246	SARS	tRNA synth	ein-coding	th protein	G00000031698
28985	0,008345728	0,0277346	0,192663855	0,5256166	12,17111347	11,64549692	0,10344	0,260174913	0,31933532	0,156498549	MCTS1	iation and	ein-coding	th protein	G00000232119
100289410	0,026487025	0,02778108	0,192878559	-0,827182	5,919195497	6,746377361	0,537923	-0,270605492	0,912992405	-0,021416493	MCF2L-AS1	antisense	n-coding R	long non-c	G00000235280
84858	0,025800388	0,02779011	0,192833163	0,5199566	6,138315385	5,61835875	0,118822	0,307036654	0,660069405	-0,062071661	ZNF503	nger prote	ein-coding	th protein	G00000165655
3315	0,017836039	0,02784756	0,192907603	0,5211003	11,26810829	10,74700794	0,401298	0,155004325	0,601527067	0,081725352	HSPB1	n family B	ein-coding	th protein	G00000106211
10990	0,008955062	0,02788422	0,192945734	0,5336788	5,474030085	4,940351288	0,012313	0,561183174	0,593955585	0,072102831	LILRB5	noglobulin	ein-coding	th protein	G00000105609
55231	0,031875649	0,0279153	0,193052905	0,6158936	7,013909019	6,39801541	0,32007	0,391329725	0,517122066	0,113670459	CCDC87	domain co	ein-coding	th protein	G00000182791
22925	0,02853058	0,02796187	0,1931593	-0,67178	5,67325049	6,345030731	0,036581	-0,433227615	0,146270247	-0,338747201	PLA2R1	lipase A2 r	ein-coding	th protein	G00000153246
4082	0,019306378	0,0279803	0,19317885	-0,744312	10,92483759	11,66914962	0,281153	-0,26543984	0,074144733	-0,552574592	MARCKS	e rich prote	ein-coding	th protein	G00000277443
7593	0,01165415	0,02798344	0,193092901	0,5720889	6,511491489	5,939402637	0,50508	0,143586337	0,924585947	0,013875182	MZF1	oid zinc fin	ein-coding	th protein	G00000099326
81610	0,025381546	0,02809927	0,193461084	0,5080923	9,217278225	8,709185926	0,002741	0,200186643	0,019693115	0,481272656	FAM83D	ence simila	ein-coding	th protein	G00000101447
85352	0,031620612	0,02811251	0,193444687	-0,707785	4,810814835	5,518600247	0,180647	-0,680244486	0,763435964	0,071679721	KIAA1644	KIAA1644	ein-coding	th protein	G00000138944
1051	0,014329957	0,02811906	0,193382346	0,9772992	13,7949248	12,81762561	0,052088	0,411112982	0,119077711	0,49528084	CEBPB	ncer bindin	ein-coding	th protein	G00000172216
728228	0,029164678	0,02814227	0,193434561	0,646051	5,541305017	4,895253995	0,893422	0,088555464	0,237050663	0,335122196	LINC01433	on-protein	n-coding R	long non-c	G00000230176
84726	0,028611012	0,0282425	0,193800805	0,5593101	8,622686775	8,063376637	0,268629	0,165168283	0,230828009	0,214545165	PRRC2B	rich coiled	ein-coding	th protein	G00000130723
79608	0,006830678	0,02827099	0,193781614	2,235102	2,235102031	0	0,858405	-0,218950668	0,062482288	2,166957524	RIC3	oline recep	ein-coding	th protein	G00000166405
286161	0,027044689	0,02831682	0,193881146	-0,706793	6,801222167	7,508015093	0,059299	-0,314796725	0,072734803	-0,352899901					
375056	0,031754083	0,02842801	0,193999034	0,5992001	8,82482564	8,22562555	0,141024	0,322977925	0,253360214	0,183419938	MIA3	ember 3, E	ein-coding	th protein	G00000154305
643454	0,021557779	0,02842933	0,193901209	-0,724867	8,894074672	9,618941382	0,055638	-0,235721254	0,662340113	-0,084953185					
134147	0,015697951	0,02844038	0,193869805	-0,879952	7,541970058	8,421922455	0,060784	-0,346316813	0,016856927	-0,502800649	CMBL	enebutenol	ein-coding	th protein	G00000164237
5671	0,032253515	0,02850502	0,194096768	-1,187215	6,96837607	8,155590921	0,672756	-0,136233332	0,331701279	-0,386543913	PSG3	cific beta-1	ein-coding	th protein	G00000221826
9181	0,032614954	0,02850683	0,194002481	0,5119549	7,511850083	6,999895168	0,996501	-0,001067074	0,139217089	0,244767873	ARHGEF2	nucleotide	ein-coding	th protein	G00000116584
51284	0,016328342	0,02856497	0,193971793	-1,48369	2,467537763	3,951227982	0,334955	-0,443010598	0,501792814	0,264660147	TLR7	like recept	ein-coding	th protein	G00000196664
7697	0,016144872	0,02856615	0,193873519	-0,727	7,569513108	8,296513246	0,209905	-0,669743294	0,937770909	-0,018204299	ZNF138	nger prote	ein-coding	th protein	G00000197008
80161	0,018507096	0,02869817	0,194449865	0,6922733	7,567678325	6,875405012	0,913125	-0,01448559	0,176656854	0,299724969	ASMTL-AS1	antisense	n-coding R	long non-c	G00000236017
400550	0,032808091	0,02871025	0,194319156	0,5471962	6,612786006	6,065589768	0,018415	0,949017654	0,167249565	-0,360861148	FENDRR	ling develo	n-coding R	long non-c	G00000268388
29078	0,025226807	0,02873476	0,194378829	0,5225324	9,7310478	9,208515404	0,63172	0,066631911	0,120709795	0,223875593	NDUFAF4	eductase c	ein-coding	th protein	G00000123545
10381	0,032241509	0,02881187	0,194581635	0,9181374	8,257195204	7,339057816	0,027714	0,481875361	0,901227183	0,027146106	TUBB3	in beta 3 cl	ein-coding	th protein	G00000258947
3475	0,029191156	0,02887699	0,194702896	0,5764219	9,519885774	8,94346389	0,136459	0,203013484	0,229862029	0,226705631	IFRD1	d developm	ein-coding	th protein	G00000006652
6285	0,022148648	0,02887871	0,194608564	1,4114343	4,492106647	3,080672357	0,807146	0,141216196	0,31127037	0,701556863	S100B	um binding	ein-coding	th protein	G00000160307
5826	0,013667943	0,02892476	0,19460126	-0,549643	7,196718549	7,746361382	0,253549	-0,156659583	0,321427956	-0,051671722	ABCD4	ette subfa	ein-coding	th protein	G00000119688
202	0,029612108	0,02894953	0,194662194	-0,918291	5,592375715	6,510666909	0,232982	-0,480475605	0,851593217	0,033604882	AIM1	it in melan	ein-coding	th protein	G00000112297
3029	0,024478598	0,02896371	0,194651857	0,5010343	10,35188079	9,850846514	0,199917	0,224674864	0,081867522	0,347040072	HAGH	lglutathion	ein-coding	th protein	G00000063854
6279	0,012635491	0,02901426	0,194569304	0,7023802	5,114460956	4,412080732	0,779275	0,18932899	0,444804997	-0,216289347	S100A8	um binding	ein-coding	th protein	G00000143546
11231	0,010552754	0,02902775	0,19444923	0,5913829	9,671688655	9,080305725	0,300846	0,197474361	0,004181987	0,318223807	SEC63	rotein tran	ein-coding	th protein	G00000025796
3065	0,029215646	0,02906747	0,194610032	0,5297036	9,810866188	9,281162582	0,255247	0,190544086	0,717178647	0,063490081	HDAC1	ne deacety	ein-coding	th protein	G00000116478
92346	0,018642404	0,02906897	0,19451493	-1,194898	3,568789343	4,763687441	0,504612	-0,539743797	0,154398234	-0,561242635	C1orf105	l open rea	ein-coding	th protein	G00000180999
131375	0,02820307	0,02908209	0,194497648	-1,016449	3,694165368	4,710614808	0,317479	-0,337280013	0,006066169	-1,156482413	LYZL4	sozyme like	ein-coding	th protein	G00000157093
4807	0,033010662	0,02915072	0,194536456	1,2314303	4,603509446	3,372079117	0,112041	0,625322596	0,360565881	0,339894681	NHLH1	t helix-loop	ein-coding	th protein	G00000171786
5587	0,020693425	0,02921431	0,19475098	0,6162964	3,995679332	3,379382906	0,094087	1,214756717	0,312714013	-0,577507785	PRKD1	tein kinase	ein-coding	th protein	G00000184304
731223	0,032198515	0,0293029	0,194608334	1,3186056	3,933444959	2,614839408	0,128307	2,488363989	0,241209282	-1,618584219	LINC01220	on-protein	n-coding R	long non-c	G00000259687
91526	0,009472774	0,02932533	0,19465297	1,0398576	5,682437208	4,642579558	0,079318	0,240395755	0,077546453	0,623751705	ANKRD44	repeat do	ein-coding	th protein	G00000065413
100526664	0,026791131	0,02937798	0,194897976	-1,045491	4,56946946	5,614960808	0,081626	-0,449589876	0,892214771	-0,037757831	.Y75-CD30	D302 readt	other	eadthroug	G00000248672
161725	0,032120502	0,02945041	0,195169401	0,6169772	5,597725384	4,980748177	0,057605	0,607431151	0,5027127	-0,160480334	OTUD7A	eubiquitin	ein-coding	th protein	G00000169918
8970	0,012071503	0,02950156	0,195195068	-0,711268	6,610981237	7,322249295	0,415808	-0,200625961	0,654679239	0,051583158	HIST1H2BJ	r 1 H2B fa	ein-coding	th protein	G00000124635
65981	0,030268095	0,02950557	0,195117373	0,5692468	8,771237314	8,201990512	0,196821	0,275945737	0,104716282	0,279758415	CAPRIN2	family me	ein-coding	th protein	G00000110888
5097	0,03216781	0,02952207	0,195122278	0,8427869	7,372538016	6,529751166	0,04346	0,816038831	0,614543115	-0,097417692	PCDH1	otocadheri	ein-coding	th protein	G00000156453
9791	0,010502951	0,0296304	0,195317167	0,5233761	9,81346563	9,290089572	0,046933	0,178035207	0,090998705	0,280456643	PTDSS1	idylserine s	ein-coding	th protein	G00000156471
168667	0,015062064	0,02968215	0,195242635	0,5449994	5,797302733	5,25230332	0,19716	0,133015141	0,250333858	0,183709551	BMPER	g endothel	ein-coding	th protein	G00000164619
114786	0,032153757	0,02972385	0,195309517	-2,331269	1,070106598	3,401375227	0,683558	0,560656345	0,259985776	-0,979740255	XKR4	K related	ein-coding	th protein	G00000206579

84984	0,013076427	0,02978972	0,195327832	-0,620626	6,420887197	7,041513591	0,333636	-0,552255502	0,034431902	0,20485496	CEP19	somal prot	ein-coding	th protein	G00000174007
387890	0,01910265	0,0298007	0,195193184	-1,004046	4,589855546	5,593901259	0,556871	-0,436842607	0,16247829	-0,354058282	TMEM233	mbrane pr	ein-coding	th protein	G00000224982
79937	0,024727681	0,02990242	0,195342962	-0,590032	5,594526954	6,184558465	0,050919	-0,786976804	0,159722263	-0,299158583	CNTNAP3	sociated p	ein-coding	th protein	G00000106714
9260	0,021251961	0,0299332	0,195337942	0,8363891	10,06329473	9,22690565	0,564167	0,13711636	0,147493247	0,414083827	PDLIM7	nd LIM dor	ein-coding	th protein	G00000196923
399473	0,027691787	0,02993532	0,195146174	0,5858759	6,578948227	5,993072298	0,115814	0,578731309	0,99206583	0,001861917	SPRED3	EVH1 dom	ein-coding	th protein	G00000188766
253650	0,033226063	0,02996286	0,195120328	-0,922539	3,68708636	4,609625251	0,039936	-0,823739027	0,431200021	-0,251405928	ANKRD18A	repeat don	ein-coding	th protein	G00000180071
3006	0,014567576	0,03000784	0,19531051	-0,857087	11,59612123	12,45320793	0,81258	-0,130213665	0,312204242	-0,238789421	HIST1H1C	er 1 H1 fam	ein-coding	th protein	G00000187837
1550	0,0209657	0,03007147	0,195621843	1,2984338	4,6595715	3,361137748	0,58268	0,285686881	0,691322523	0,189314427	CYP2A7P1	subfamily	pseudogene	pseudogene	G00000213908
144402	0,033882268	0,03008697	0,195619973	-0,583573	7,072966232	7,65653968	0,253922	-0,228593037	0,770838263	-0,043464323	CPNE8	copine 8	ein-coding	th protein	G00000139117
23436	0,020960151	0,03010394	0,195525011	1,3530817	4,815324401	3,462242681	0,249625	-0,757702704	0,055947197	1,225767148	CELA3B	e elastase f	ein-coding	th protein	G00000219073
10826	0,03376271	0,03018783	0,195761933	-0,554008	6,548697404	7,102704941	0,473301	0,130513065	0,014919824	-0,697187312	FAXDC2	xylase dom	ein-coding	th protein	G00000170271
729987	0,03022379	0,03019022	0,195674996	-1,661119	4,141470316	5,802589448	0,20955	-0,991425375	0,221370381	-0,961642348					
55165	0,020236649	0,03023175	0,195739322	0,6447546	10,0850818	9,440327235	0,98559	0,004593284	0,004470554	0,545627122	CEP55	somal prot	ein-coding	th protein	G00000138180
10078	0,02276441	0,03027393	0,195807708	0,5630467	9,338678369	8,775631714	0,065886	0,320623804	0,572180305	0,089688085	TSSC4	g subtransf	ein-coding	th protein	G00000184281
84287	0,032809156	0,03028166	0,195755426	-0,538944	8,999292645	9,53823657	0,880545	-0,026630764	0,663439957	-0,066339158	ZDHHC16	HHC-type c	ein-coding	th protein	G00000171307
768	0,016707268	0,03035412	0,195917101	-0,731471	6,668583242	7,400054075	0,681986	-0,096355943	0,051610682	-0,617890033	CA9	onic anhydr	ein-coding	th protein	G00000107159
340090	0,007993089	0,03041262	0,19609035	-2,098337	2,753688488	4,852024999	0,127417	-1,385008039	0,182120422	-1,102496782					
11119	0,014239791	0,03043835	0,196052124	-0,525987	7,554909121	8,080896552	0,777753	0,050642035	0,049320799	-0,51581668	BTN3A1	subfamily	ein-coding	th protein	G00000026950
375567	0,031306588	0,03053687	0,196482322	1,4258609	4,357910812	2,93204993	0,333854	0,791975417	0,536135986	0,301557937	VWC2	actor C dor	ein-coding	th protein	G00000188730
9770	0,033847605	0,03059355	0,196337035	0,5929948	5,561676353	4,968681576	0,303309	0,214612531	0,179171781	0,324731867	RASSF2	domain fa	ein-coding	th protein	G00000101265
8794	0,01520137	0,03064551	0,196568628	-0,645978	6,222896565	6,868874791	0,610561	0,069549861	0,021266855	-0,239833299	TNFRSF10C	superfamil	ein-coding	th protein	G00000173535
4666	0,015631107	0,03076443	0,197127304	0,7658844	9,510285292	8,744400872	0,005822	0,209224958	0,166429172	0,346130301	NACA	ssociated	ein-coding	th protein	G00000196531
115196	0,017882364	0,03080091	0,197258975	0,5336533	7,468010257	6,934356964	0,006659	0,339922258	0,576881394	0,079424013	ZNF554	nger prote	ein-coding	th protein	G00000172006
403296	0,026637666	0,03080271	0,197168579	-0,562976	7,2690477	7,832023354	0,010763	-0,495518756	0,319017406	-0,128371688	OR7E154P	subfamily	pseudogene	pseudogene	G00000254715
7531	0,013240405	0,03084283	0,197323373	-0,76061	10,60322325	11,36383301	0,109749	-0,390206659	0,118993436	0,403390949	YWHAE	an 5-mon	ein-coding	th protein	G00000108953
27030	0,031190867	0,0308745	0,197322163	-0,587493	6,19108086	6,778574166	0,202501	-0,691990963	0,853841222	-0,029192522	MLH3	utL homolo	ein-coding	th protein	G00000119684
90806	0,02815173	0,03092179	0,197217343	-0,63843	7,335214444	7,973643995	0,137426	-0,424673271	0,369716959	-0,173903608	ANGEL2	gel homolo	ein-coding	th protein	G00000174606
9915	0,011969209	0,03096277	0,197275543	-0,681459	6,93608107	7,617540445	0,246658	-0,191675118	0,686339942	-0,049875191	ARNT2	eceptor nu	ein-coding	th protein	G00000172379
9982	0,026052352	0,03097589	0,197257661	-0,791205	4,359056537	5,150261916	0,865928	0,042400513	0,148579184	-0,433601675	FGFBP1	th factor bi	ein-coding	th protein	G00000137440
6882	0,011432843	0,0310161	0,197412213	1,0796547	10,42197896	9,342324231	0,021222	0,776476483	0,261693819	0,213485269	TAF11	protein as	ein-coding	th protein	G00000064995
79912	0,019039378	0,031058	0,197475966	0,5968921	7,849236529	7,252344446	0,06988	0,264269678	0,64761909	0,050899685	PYROXD1	ulphide ox	ein-coding	th protein	G00000121350
3354	0,020090228	0,03107282	0,197367545	1,1018429	5,192117882	4,090274994	0,165453	0,669359303	0,181356038	0,408791027	HTR1E	ryptamine	ein-coding	th protein	G00000168830
54584	0,011362552	0,03110929	0,197396764	0,513838	6,902357598	6,388519625	0,407451	-0,073630105	0,075874678	0,201530507	GNB1L	n subunit b	ein-coding	th protein	G00000185838
6717	0,013959102	0,03121325	0,197954965	0,6894751	11,62652475	10,93704967	0,039363	0,288835743	0,120791949	0,356710631	SRI	sorcin	ein-coding	th protein	G00000075142
203260	0,032221141	0,03123994	0,197820361	0,6795031	10,71227781	10,03277475	0,133576	0,289497761	0,181327197	0,317170124	CCDC107	domain cor	ein-coding	th protein	G00000159884
84557	0,034904764	0,0312434	0,197741194	0,5223574	7,74634558	7,223988284	0,191457	0,322645534	0,433578412	0,146791431	MAP1LC3A	ed protein	ein-coding	th protein	G00000101460
340526	0,020167784	0,03125591	0,197719335	1,2022967	10,89059576	9,688299094	0,079736	0,990404654	0,608295661	-0,227100901	RGAG4	n gag dom	ein-coding	th protein	G00000242732
122773	0,011183282	0,0312629	0,1976626	-0,79695	5,740337098	6,537287514	0,167903	-0,283963833	0,176062995	-0,33653562	KLHDC1	omain cont	ein-coding	th protein	G00000197776
149992	0,035105616	0,03127298	0,197625485	1,4798247	3,740031759	2,260207046	0,246592	0,76914903	0,245426572	0,801388822	NKRD30BP	domain 30	pseudogene	pseudogene	G00000224309
64840	0,034917271	0,03132337	0,197540729	-0,556065	9,419876665	9,975941857	0,333443	-0,240842167	0,158468296	-0,274808846	PORCN	homolog (I	ein-coding	th protein	G00000102312
23190	0,02420585	0,03133035	0,197484189	-0,774504	9,633488158	10,40799182	0,103495	-0,302445135	0,843480078	-0,044136195	UBXN4	domain pro	ein-coding	th protein	G00000144224
2934	0,00915378	0,03136977	0,197531624	-0,575659	8,657210074	9,232869358	0,454077	-0,12862484	0,012853461	-0,620027419	GSN	gelsolin	ein-coding	th protein	G00000148180
84671	0,03453471	0,03149065	0,198091408	-0,920461	6,734633947	7,655094455	0,634401	-0,145267755	0,352311463	-0,280672171	ZNF347	nger prote	ein-coding	th protein	G00000197937
3269	0,034682069	0,03149275	0,198004018	0,5061533	6,467651258	5,961497933	0,565334	0,302724335	0,949329225	-0,032375209	HRH1	nine recep	ein-coding	th protein	G00000196639
401480	0,023864269	0,03166581	0,198688687	0,738845	7,453746016	6,714901017	0,320761	0,389947608	0,517865557	0,226299888					
23002	0,034117704	0,03168025	0,198678617	-0,566369	7,701092641	8,267461533	0,127554	-0,104925688	0,121335517	-0,325730042	DAAM1	ed activato	ein-coding	th protein	G00000100592
5105	0,029298932	0,03170354	0,198623551	0,5622527	4,520936679	3,958683984	0,165112	0,70502046	0,254161339	-0,165477491	PCK1	pyruvate ca	ein-coding	th protein	G00000124253
80320	0,028360354	0,03170801	0,198551139	-0,591838	7,357600896	7,949439114	0,313334	-0,197311673	0,30834541	-0,213042071	SP6	anscription	ein-coding	th protein	G00000189120
9935	0,03349778	0,03171032	0,198465297	0,6533283	6,648523912	5,995195653	0,708673	-0,286796946	0,189635802	0,301657645	MAFB	transcripti	ein-coding	th protein	G00000204103
79080	0,014097933	0,03179207	0,198675729	0,8912752	9,546661118	8,655385906	0,851987	0,039580438	0,303417947	0,286757209	CCDC86	domain co	ein-coding	th protein	G00000110104
54566	0,027125546	0,03186261	0,198415813	0,5071278	7,175468908	6,668341111	0,647437	0,149215113	0,958531172	0,013845924	EPB41L4B	brane prote	ein-coding	th protein	G00000095203
5669	0,008299575	0,0318728	0,19837951	-2,992057	3,300811372	6,292868146	0,335205	-0,644875579	0,176552677	-1,162364302	PSG1	cific beta-1	ein-coding	th protein	G00000231924
26502	0,016460197	0,03188431	0,198351494	0,6526836	9,463389061	8,810705444	0,000673	0,408922059	0,257466545	0,220263653	NARF	min A reco	ein-coding	th protein	G00000141562
342918	0,023717864	0,03191685	0,198354685	0,5164909	6,461025537	5,944534651	0,071469	0,416221291	0,382935234	-0,151057203	C19orf81	19 open re	ein-coding	th protein	G00000235034
4619	0,023447143	0,03202189	0,198708379	0,5591907	3,808136889	3,248946226	0,209488	0,82457491	0,496508396	-0,358060031	MYH1	in heavy ch	ein-coding	th protein	G00000109061
3753	0,0113713	0,03204796	0,198671063	-0,525878	5,958156983	6,484034731	0,180106	-0,377029467	0,539036301	-0,076990395	KCNE1	annel subf	ein-coding	th protein	G00000180509
374378	0,03469225	0,03208248	0,198785587	0,5274124	7,780115814	7,252703413	0,754395	0,083551906	0,339065895	0,260334929	GALNT18	ylgalactosa	ein-coding	th protein	G00000110328
29948	0,029406082	0,03218581	0,199226481	-0,550271	6,701773442	7,252044547	0,088446	-0,488210213	0,197548094	-0,159367892	OSGIN1	induced gr	ein-coding	th protein	G00000140961
2707	0,035990895	0,03218846	0,199143367	-0,850717	5,812765999	6,663482824	0,516861	-0,151650746	0,726509825	-0,077423219	GJB3	tion protei	ein-coding	th protein	G00000188910

79165	0,029986842	0,03223319	0,199320514	0,6660193	8,286722624	7,620703315	0,376223	0,12786067	0,381418921	0,181471992	LENG1	eptor clust	ein-coding	th protein	G00000105617
5167	0,032926888	0,03226908	0,199343395	0,5453853	8,862763132	8,317377844	0,061596	0,370867105	0,28178544	0,205674485	ENPP1	osphatase	ein-coding	th protein	G00000197594
93624	0,031005804	0,03240432	0,199680885	0,8183691	7,668426061	6,850056985	0,097305	0,273914654	0,133415265	0,456725156	TADA2B	ptional ada	ein-coding	th protein	G00000173011
5997	0,027850936	0,03248142	0,199857696	0,9158678	7,181558625	6,265690821	0,191898	0,348065025	0,321697193	-0,256792176	RGS2	f G-protein	ein-coding	th protein	G00000116741
51655	0,033869336	0,03250999	0,199934187	0,8472695	9,293423462	8,446153983	0,066142	0,673062203	0,129252816	-0,478570838	RASD1	examethas	ein-coding	th protein	G00000108551
5740	0,023958742	0,03252618	0,199835331	0,6463059	5,018395784	4,372089911	0,021636	0,962909625	0,527768603	-0,132421431	PTGIS	I2 (prostac	ein-coding	th protein	G00000124212
112714	0,036392683	0,03254695	0,199764717	-0,780779	5,15982472	5,94060345	0,468172	-0,420575041	0,482299324	-0,193184251	TUBA3E	ulin alpha	ein-coding	th protein	G00000152086
26022	0,031902754	0,03275439	0,200145301	0,8379618	6,164726821	5,32676499	0,036257	0,683093934	0,927998817	0,02181066	TMEM98	mbrane pr	ein-coding	th protein	G00000006042
285636	0,035856072	0,0327626	0,200096786	0,8815307	8,412486059	7,53095533	0,276364	0,343507474	0,047437526	0,650813185	C5orf51	5 open rea	ein-coding	th protein	G00000205765
9924	0,014348296	0,03277558	0,200077394	-0,551934	5,897685893	6,449619402	0,017376	-0,352950632	0,433315735	-0,114509826	PAN2	ecific ribor	ein-coding	th protein	G00000135473
54930	0,013351415	0,03292027	0,200073171	-0,645426	8,774854504	9,420280914	0,066102	-0,252661419	0,013480831	-0,293580615	HAUS4	n like comp	ein-coding	th protein	G00000092036
441024	0,013814009	0,03294881	0,200148427	0,6000138	6,977698919	6,377685108	0,784992	-0,076457307	0,217534668	0,220927071	MTHFD2L	ehydrogen	ein-coding	th protein	G00000163738
120776	0,034707225	0,03312812	0,20064725	0,6951353	4,825551762	4,130416512	0,625042	0,049490883	0,083325944	0,382029062	OR2D2	amily 2 sub	ein-coding	th protein	G00000166368
23650	0,035502378	0,03313894	0,200614685	-0,513309	7,78761249	8,300921086	0,204369	0,186018454	0,004806491	-0,916757361	TRIM29	motif cont	ein-coding	th protein	G00000137699
9906	0,021130077	0,03317951	0,200664105	-0,515112	10,39877918	10,91389083	0,173837	-0,263084422	0,78191655	-0,034528918	SLC35E2	r family 35	ein-coding	th protein	G00000215790
763	0,034974093	0,0332221	0,200823617	0,6820452	5,185751842	4,503706678	0,029191	0,740941879	0,595039859	-0,124067443	CA5A	nic anhydr	ein-coding	th protein	G00000174990
1891	0,029682658	0,03324454	0,200861226	-0,574417	10,09646599	10,67088255	0,623197	-0,061078721	0,2043978	-0,264966936	ECH1	-CoA hydra	ein-coding	th protein	G00000104823
3934	0,023399865	0,03325276	0,200812983	-1,630563	10,33870647	11,96926907	0,145871	-0,67031323	0,371022225	-0,48782071	LCN2	lipocalin 2	ein-coding	th protein	G00000148346
4357	0,033160659	0,03328182	0,200890502	-0,562978	7,429579639	7,992557719	0,34582	0,415759893	0,248462933	-0,491892697	MPST	uvate sulf	ein-coding	th protein	G00000128309
4793	0,025751972	0,03331294	0,200784774	0,8203246	8,025552435	7,205227826	0,09627	0,403094336	0,123589537	0,482382258	NFKBIB	B inhibitor	ein-coding	th protein	G00000104825
2509	0,024599297	0,03336435	0,200801545	-0,623259	15,82746973	16,4507289	0,106275	-0,264345914	0,639227439	-0,064718993	FTH1P5	y chain 1 p	seudogen	oseudogen	G00000230204
91807	0,010566968	0,03339691	0,200899887	0,7293948	5,173217357	4,443822582	0,08826	0,464910456	0,230673753	0,272734551	MYLK3	light chain	ein-coding	th protein	G00000140795
63910	0,024359884	0,03340262	0,200836695	0,6262929	8,440782827	7,814489971	0,085053	0,336017948	0,394873797	0,175700298	SLC17A9	er family 1	ein-coding	th protein	G00000101194
168620	0,033012982	0,03345448	0,200953363	0,6054481	7,520529366	6,915081252	0,194528	0,653597003	0,896097137	-0,024214838	BHLHA15	-helix fami	ein-coding	th protein	G00000180535
339400	0,024308219	0,03348094	0,200917467	-0,673911	5,303371311	5,977281812	0,018065	-0,780661218	0,081125384	0,49491542	FLG-AS1	ntisense R	n-coding R	long non-c	G00000237975
5366	0,033052469	0,03349572	0,200811556	-1,014007	9,915372012	10,92937892	0,088408	-0,581370161	0,439115535	-0,23875189	PMAIP1	e-13-aceta	ein-coding	th protein	G00000141682
57186	0,035120757	0,03356153	0,200720308	-0,887029	5,537867163	6,424896531	0,129142	-0,648772111	0,289086334	0,303330416	RALGAPA2	protein ca	ein-coding	th protein	G00000188559
83901	0,009682822	0,0335707	0,200581455	-2,140791	3,260395326	5,40118602	0,532746	-0,25996013	0,598592545	0,267491099	KRTAP9-8	sociated p	ein-coding	th protein	G00000187272
266722	0,01373342	0,0335724	0,20049488	2,8583977	3,565389993	0,70699226	0,245845	1,213791979	0,581007627	0,572877836	HS6ST3	te 6-O-sulf	ein-coding	th protein	G00000185352
718	0,023002111	0,0335745	0,200410828	-1,404285	7,898368592	9,302653489	0,068508	-0,633276748	0,129207185	-0,779251435	C3	mplement	ein-coding	th protein	G00000125730
79158	0,009154617	0,03381556	0,2010745	-0,695856	7,199936421	7,895792592	0,060296	-0,473054806	0,670118046	-0,068694911	GNPTAB	hate transf	ein-coding	th protein	G00000111670
5203	0,037217222	0,03384372	0,201048912	0,5157431	8,326432682	7,810689586	0,576168	-0,54233122	0,933273285	-0,025358907	PFDN4	oldin subu	ein-coding	th protein	G00000101132
677777	0,009293443	0,0338526	0,20100525	-0,643218	9,195995446	9,839213656	0,151797	-0,889214976	0,17051238	-0,246745876	SCARNA12	body-spec	n-coding R	small nucl	G00000238795
100131938	0,015586878	0,03386205	0,200965024	0,7360453	6,788903262	6,052857967	0,008773	0,916328693	0,209077616	-0,288757695					
4982	0,037255168	0,03389616	0,200974885	-1,463435	3,855831135	5,319265984	0,185674	-0,653329758	0,549347606	-0,300365825	TNFRSF11B	superfamil	ein-coding	th protein	G00000164761
3433	0,009704813	0,03392364	0,200849363	1,0471057	8,766941234	7,719835488	0,067427	1,115643967	0,581448222	-0,22298175	IFIT2	ein with tet	ein-coding	th protein	G00000119922
10627	0,030283123	0,03397533	0,200963268	-0,58294	13,37159529	13,95453575	0,999457	0,000126041	0,330146071	-0,197438543	MYL12A	n light cha	ein-coding	th protein	G00000101608
23552	0,031753913	0,03402528	0,200970833	0,6467528	7,109603258	6,462850451	0,04326	0,323090261	0,894716837	-0,025765456	CDK20	ependent k	ein-coding	th protein	G00000156345
2730	0,009558322	0,03402585	0,200878377	-0,779537	8,827568071	9,607104716	0,006246	-0,72262785	0,336626029	0,194110932	GCLM	ine ligase r	ein-coding	th protein	G00000023909
114926	0,026535917	0,03406368	0,200814582	-0,824175	6,782510334	7,606685717	0,105969	-0,503008998	0,791547925	0,060614791	SMIM19	il membra	ein-coding	th protein	G00000176209
285628	0,028725856	0,0340894	0,200775074	-1,496861	6,538873603	8,035734414	0,059287	-0,807930096	0,693363969	-0,120044399					
84301	0,009643722	0,0341183	0,200849742	-0,562527	6,860133118	7,422659941	0,204253	-0,670679379	0,583168199	0,079426007	DDI2	e inducible	ein-coding	th protein	G00000197312
201229	0,019840855	0,03415631	0,200978008	-0,97777	6,501001277	7,478771086	0,401094	-0,569825873	0,010620239	-0,716761338	LYRM9	notif conta	ein-coding	th protein	G00000232859
9699	0,036086619	0,03426303	0,20122357	0,537203	4,33015631	3,792953283	0,071124	0,775462412	0,436920871	-0,247267627	RIMS2	tic membr	ein-coding	th protein	G00000176406
79735	0,035218757	0,03427919	0,201127768	0,7353436	8,967213646	8,231870021	0,008488	0,656411389	0,389857953	0,200851435	TBC1D17	ain family r	ein-coding	th protein	G00000104946
23313	0,028171018	0,0343219	0,201092577	-0,67071	6,355881651	7,026592078	0,009996	-0,550312509	0,916890344	-0,01238291	KIAA0930	KIAA0930	ein-coding	th protein	G00000100364
131096	0,021832086	0,03438163	0,201157073	1,532141	2,284306909	0,752165942	0,720339	-0,423163274	0,090426089	1,368499991	KCNH8	ed channel	ein-coding	th protein	G00000183960
280	0,023688911	0,03440153	0,20117847	-0,755938	5,294709469	6,050647109	0,010881	-0,428073598	0,04782968	-0,323513757	AMY2B	alpha 2B (p	ein-coding	th protein	G00000240038
84993	0,025791714	0,0344636	0,201446335	0,6306298	9,389611565	8,758981777	0,141442	0,333922575	0,502311086	0,13162434	UBL7	biquitin like	ein-coding	th protein	G00000138629
8313	0,009492692	0,03450324	0,201487892	0,5535154	5,576123782	5,022608378	0,707832	0,080791124	0,101489875	0,414547579	AXIN2	axin 2	ein-coding	th protein	G00000168646
643224	0,037950006	0,03452194	0,201502056	0,5855453	10,60639862	10,02085337	0,187198	0,3003441	0,348445297	0,197873715	TUBBP5	beta pseud	seudogen	oseudogen	G00000159247
195827	0,012437427	0,03454087	0,201422703	-0,674608	8,651361861	9,325969871	0,131197	-0,233718152	0,052827937	-0,206119912	AAED1	nt enzyme	ein-coding	th protein	G00000158122
100128590	0,009351939	0,0346787	0,201656813	2,6723261	2,672326088	0	0,411968	1,404805931	0,422649731	1,342133905	SLC8A1-AS1	antisense	n-coding R	long non-c	G00000227028
79695	0,01037199	0,0347762	0,20175016	-0,606658	5,759777271	6,36643479	0,113999	-0,486432583	0,933577817	0,012974556	GALNT12	ylgalactosa	ein-coding	th protein	G00000119514
6335	0,038474264	0,03484997	0,201800089	-1,171504	3,32880927	4,500313616	0,84528	0,07335259	0,125428837	-0,74935124	SCN9A	ated chann	ein-coding	th protein	G00000169432
143888	0,022085888	0,0349161	0,202088525	-0,659961	7,952748765	8,612710076	0,086959	-0,312033268	0,17936426	0,305448788	KDELC2	notif conta	ein-coding	th protein	G00000178202
138428	0,037975597	0,03496921	0,20192411	0,5803563	10,0458598	9,465503541	0,02296	0,401010553	0,040591886	0,466015423	PTRH1	A hydrolas	ein-coding	th protein	G00000187024
131149	0,038742367	0,03503526	0,201928964	0,5984146	2,028498213	1,430083601	0,661233	0,312022251	0,321886842	-0,626321252	OTOL1	otolin 1	ein-coding	th protein	G00000182447
7169	0,018528603	0,03503541	0,201835885	1,1714894	9,64997333	8,478483946	0,852369	0,066321815	0,030666759	0,461012008	TPM2	myosin 2 (	ein-coding	th protein	G00000198467

91442	0,014983763	0,03504138	0,201682672	0,594624	6,859070442	6,264446475	0,24361	-0,328646132	0,469462525	0,116445664	FAAP24	complex a	ein-coding	th protein	G00000131944
79174	0,034161463	0,03508829	0,201765143	0,6703314	8,775835015	8,105503613	0,212972	0,365918547	0,10264987	0,437065539	CRELD2	with EGF li	ein-coding	th protein	G00000184164
124149	0,015570494	0,03518695	0,202144786	0,8690143	3,542531214	2,673516899	0,099207	0,886013721	0,251525214	0,519600323	ANKRD26P	domain 26	seudogeno	seudogen	G00000261239
253725	0,033028955	0,03520498	0,202060925	-0,572896	8,8179022	9,390798215	0,074757	-0,306790908	0,217150483	-0,250662804	FAM21C	ence simila	ein-coding	th protein	G00000172661
3267	0,012775313	0,03527335	0,202078764	-0,602792	8,590273805	9,193065388	0,007922	-0,372606732	0,72108703	0,021455428	AGFG1	with FG re	ein-coding	th protein	G00000173744
54504	0,021005913	0,0352974	0,202029655	-0,809151	5,678638951	6,487790358	0,276507	-0,277760387	0,303449299	-0,185110501	CPVL	tidase, vite	ein-coding	th protein	G00000106066
255119	0,016731473	0,03532467	0,202092339	2,4039574	3,414379589	1,010422221	0,979507	0,024162875	0,046855448	1,953927462	C4orf22	4 open rea	ein-coding	th protein	G00000197826
57659	0,012246734	0,03532675	0,202010954	0,6416653	9,794199356	9,152534049	0,345889	0,232782089	0,764190867	0,036944221	ZBTB4	BTB doma	ein-coding	th protein	G00000174282
1462	0,035994526	0,0353613	0,202115195	0,6222223	4,564092529	3,941870263	0,046077	0,603135643	0,902426466	-0,026747239	VCAN	versican	ein-coding	th protein	G00000038427
157310	0,038406739	0,03541098	0,202212559	0,5370138	4,954030364	4,417016528	0,249879	0,457435467	0,98407481	-0,006137047	PEBP4	anolamine	ein-coding	th protein	G00000134020
6759	0,034930455	0,03544621	0,20222727	1,0921338	2,715288232	1,623154386	0,211342	0,403341666	0,347723636	0,239537499	SSX4	amily mem	ein-coding	th protein	G00000268009
79693	0,026890085	0,03549393	0,202406283	0,5762707	10,38229537	9,806024693	0,106797	0,225982723	0,252983306	0,228941851	YRDC	noyltransfe	ein-coding	th protein	G00000196449
113763	0,014476534	0,03554917	0,20225568	-0,836076	9,210027913	10,04610371	0,867671	0,043096044	0,040811186	-0,5789105	ZBED6CL	6 C-termin	ein-coding	th protein	G00000188707
23529	0,027677277	0,03557927	0,202334008	0,5561959	9,471369343	8,915173424	0,571602	0,112211869	0,253942498	0,272453641	CLCF1	n-like cyto	ein-coding	th protein	G00000175505
817	0,019630731	0,03557965	0,202243275	-0,663192	7,869029343	8,532221656	0,063137	-0,223260373	0,216569725	-0,265337756	CAMK2D	ependent	ein-coding	th protein	G00000145349
5051	0,019825698	0,0356164	0,20235932	-0,785855	5,499807657	6,285662542	0,002404	-0,362812041	0,221340213	-0,120910403	PAFAH2	ng factor a	ein-coding	th protein	G00000158006
57232	0,013032877	0,03563931	0,202303904	-0,663289	5,008229461	5,671518228	0,561469	-0,183833238	0,561454963	0,173243981	ZNF630	nger prote	ein-coding	th protein	G00000221994
149830	0,028120712	0,03569687	0,202259827	0,7524847	3,804596988	3,052112299	0,100849	1,103052006	0,86436841	-0,082281562	PRNT	otein (testis	other	unknown	G00000180259
100128554	0,015893918	0,03573243	0,202368753	2,6800507	3,267477609	0,587426924	0,125846	1,86528023	0,395863711	0,9296326					
26184	0,02634631	0,03574599	0,202168228	-1,177887	4,119012142	5,296898801	0,589377	0,378366047	0,553822987	-0,368426044	OR1F2P	1 subfamily	seudogeno	seudogen	G00000203581
5705	0,028628499	0,03582461	0,202151351	0,5164597	11,56849781	11,05203815	0,183514	0,176724296	0,28694124	0,195866616	PSMC5	26S subur	ein-coding	th protein	G00000087191
169611	0,020106836	0,03609835	0,203325501	-0,877045	6,767993914	7,645038585	0,45544	-0,193430794	0,186845695	-0,399500469	OLFML2A	tomedin lik	ein-coding	th protein	G00000185585
9709	0,013312627	0,03610366	0,203262935	0,8748842	10,91754742	10,04266321	0,026878	0,723527531	0,065415522	0,332803923	HERPUD1	protein w	ein-coding	th protein	G00000051108
285033	0,027749483	0,03613444	0,203343834	0,5451592	6,562395479	6,017236266	0,346437	-0,238430488	0,33852194	0,170409568	STARD7-AS	7 antisense	n-coding R	long non-c	G00000204685
54882	0,021761833	0,03624	0,203291331	0,5472142	9,133944851	8,586730636	0,422613	0,227124277	0,433871271	0,195560244	ANKHD1	nd KH dom	ein-coding	th protein	G00000131503
152065	0,013256774	0,03628405	0,20344626	0,7724217	5,881905917	5,109484244	0,22905	0,14508718	0,241582531	0,266196735	C3orf22	3 open rea	ein-coding	th protein	G00000180697
84641	0,011456546	0,03629353	0,203407362	-0,600483	8,563734801	9,164217523	0,017104	-0,3788451	0,654131812	-0,066439609	MFSD14B	erfamily dc	ein-coding	th protein	G00000148110
50484	0,036756135	0,03629815	0,20334124	-0,857472	6,457099725	7,314571741	0,246073	-0,512657915	0,501655784	0,173939074	RRM2B	egulatory T	ein-coding	th protein	G00000048392
254065	0,012027243	0,03634389	0,203321594	-0,633751	6,469939391	7,103689959	0,186449	-0,530545031	0,340570326	0,181168178	BRWD3	VD repeat	ein-coding	th protein	G00000165288
91351	0,031849465	0,03634551	0,203147131	-0,8378	6,492439561	7,330239924	0,369149	-0,206630384	0,863424223	-0,042679867	DDX60L	ox helicase	ein-coding	th protein	G00000181381
5547	0,019609922	0,03636822	0,203182407	-0,637793	8,607033889	9,244826994	0,519134	-0,027042924	0,07330972	-0,165297315	PRCP	carboxypep	ein-coding	th protein	G00000137509
6713	0,02903699	0,03644198	0,203319332	0,5019087	8,861465384	8,359556667	0,252791	0,24409564	0,77952194	-0,044786785	SQLE	lene epoxi	ein-coding	th protein	G00000104549
399706	0,011764757	0,03647202	0,20339534	0,6302075	5,831161884	5,200954375	0,159426	0,509496379	0,498340908	-0,190825477	LINC00200	on-protein	n-coding R	long non-c	G00000229205
55714	0,04018615	0,03655469	0,203489857	-0,739879	4,586100127	5,325979385	0,026721	-0,827725962	0,84121488	0,03968555	TENM3	nsmembra	ein-coding	th protein	G00000218336
55861	0,030735968	0,03662612	0,203704405	0,5497051	5,573481638	5,023776557	0,748134	-0,146590272	0,179808473	0,778913175	DBNDD2	domain co	ein-coding	th protein	G00000244274
5458	0,014287717	0,03668747	0,203862501	-1,884071	2,998515215	4,882585746	0,600866	-0,349761656	0,720862402	-0,101152634	POU4F2	ass 4 home	ein-coding	th protein	G00000151615
27121	0,03707984	0,03669344	0,20380425	0,6028367	5,515387408	4,912550663	0,61372	0,073175481	0,032469885	0,534870643	DKK4	gnaling pat	ein-coding	th protein	G00000104371
10146	0,012008115	0,03670977	0,203803566	-0,970947	9,747935406	10,71888223	0,001015	-0,559147886	0,477235858	-0,173662266	G3BP1	ranule asse	ein-coding	th protein	G00000145907
2110	0,010315261	0,03673976	0,203878656	-0,942197	5,560007435	6,502204269	0,188269	-0,641347201	0,314419143	0,247712997	ETFDH	flavoprote	ein-coding	th protein	G00000171503
10558	0,027851123	0,03674406	0,203811237	-0,552984	8,453433475	9,006417559	0,658397	-0,084907418	0,258787836	-0,21183455	SPTLC1	ferase long	ein-coding	th protein	G00000090054
94134	0,028982949	0,03680625	0,203882288	-0,902545	8,580084176	9,482629257	0,20523	-0,453599356	0,823992065	-0,059015175	ARHGAP12	activating	ein-coding	th protein	G00000165322
142	0,013612521	0,03685263	0,203956762	-0,641828	8,528933371	9,17076187	0,013638	-0,349905406	0,913844634	-0,00824709	PARP1	ribose) pol	ein-coding	th protein	G00000143799
54841	0,018256286	0,03697207	0,204252707	-0,651858	6,066664153	6,7185223	0,01613	-0,496828401	0,619680929	-0,051046852	BIVM	in-like vari	ein-coding	th protein	G00000134897
11278	0,019016755	0,03697854	0,204197381	-0,695301	5,677243935	6,372545164	0,048823	-0,336041627	0,778639439	-0,03277752	KLF12	pel like fact	ein-coding	th protein	G00000118922
3933	0,012448058	0,03714692	0,204579956	0,6579337	5,732184029	5,074250323	0,097999	0,433389833	0,202169018	0,164888306	LCN1	lipocalin 1	ein-coding	th protein	G00000160349
599	0,011592315	0,03715798	0,204549891	0,5499899	11,53787278	10,98788291	0,800877	0,030845621	0,512173737	-0,089666004	BCL2L2	BCL2 like 2	ein-coding	th protein	G00000129473
50839	0,034295665	0,03721782	0,204697362	-0,581084	5,409707626	5,990791656	0,06938	-0,565123396	0,966450834	-0,007521308	TAS2R10	eceptor me	ein-coding	th protein	G00000121318
338653	0,040697494	0,03722725	0,20465834	-0,635323	6,154574405	6,789897647	0,437063	0,261856726	0,24668984	-0,425035569	KCNQ1-AS1	1 antisense	n-coding R	long non-c	G00000229414
100131289	0,021856575	0,03725781	0,204735481	-1,251869	4,039344204	5,29121277	0,423252	-1,638893423	0,205687139	-0,544778922					
7162	0,024920017	0,03733896	0,205090409	0,7034655	8,372598263	7,66913275	0,359289	0,242602918	0,158765675	0,196641207	TPBG	blast glyco	ein-coding	th protein	G00000146242
154091	0,037770366	0,03736165	0,205124088	-0,934801	5,550218164	6,485018784	0,078915	-0,622192471	0,24163341	-0,375318783	SLC2A12	er family 2	ein-coding	th protein	G00000146411
3049	0,02632914	0,0374264	0,205115911	0,5342517	5,521335673	4,987084014	0,657381	0,192437502	0,316655485	0,230165409	HBQ1	obin subuni	ein-coding	th protein	G00000086506
79180	0,037958672	0,03743645	0,205080225	0,5868276	11,88441446	11,29758688	0,159305	0,219577277	0,203978669	0,214861435	EFHD2	nain family	ein-coding	th protein	G00000142634
26683	0,017942557	0,03744364	0,204938309	0,9240722	3,65947571	2,735403557	0,035909	-1,716474221	0,041582979	1,233709122	OR4F3	amily 4 sul	ein-coding	th protein	G00000230178
83879	0,020864426	0,03746838	0,204983169	-0,983985	5,684351093	6,668335929	0,018999	-0,918952287	0,489801194	0,219625318	CDCA7	on cycle ass	ein-coding	th protein	G00000144354
578	0,018971878	0,03751374	0,205140691	0,5880194	8,753841825	8,16582239	0,170839	0,30150158	0,929076612	0,014298149	BAK1	ntagonist/	ein-coding	th protein	G00000030110
84298	0,013162442	0,03765047	0,205616061	0,6158997	9,97959137	9,3636917	0,660827	-0,141982235	0,000966326	0,315412106	LLPH	g-term syn	ein-coding	th protein	G00000139233
10485	0,013929743	0,03765409	0,205545222	-0,503399	4,679484213	5,182883462	0,932384	-0,02736637	0,121509392	-0,302917074	C1orf61	1 open rea	ein-coding	th protein	G00000125462
55370	0,036562449	0,03766974	0,205540034	-0,502798	6,288387208	6,791185504	0,275686	-0,226482042	0,400377373	-0,144943799	PPP4R1L	gulatory su	seudogeno	seudogen	G00000124224

6996	0,040849076	0,03766974	0,205449562	0,589289	8,466456976	7,877167939	0,331869	0,147747923	0,268794878	-0,213735778	TDG	e DNA glyc	ein-coding	th protein	G00000139372
403313	0,03355669	0,03771561	0,205428362	-0,958204	5,593247461	6,551451015	0,111845	-0,953432177	0,229134866	0,273627614	PLPP6	lipid phosp	ein-coding	th protein	G00000205808
57111	0,011811572	0,03787052	0,205909929	-0,82296	5,019580869	5,842541138	0,061775	-0,18784537	0,008203103	-0,305551114	RAB25	ber RAS on	ein-coding	th protein	G00000132698
11104	0,03901269	0,03793594	0,206175107	0,5944576	7,874327975	7,279870356	0,030376	0,353727897	0,348379628	0,149578036	KATNA1	catalytic su	ein-coding	th protein	G00000186625
100132169	0,01217876	0,0379706	0,206182549	0,6924599	6,330080463	5,637620559	0,126902	0,431065885	0,597123027	0,111532727	WASIR2	IL9R antise	n-coding R	long non-c	G00000231439
9060	0,032036211	0,03802622	0,206394071	-0,730328	8,381133339	9,111461372	0,325053	-0,166714677	0,80458278	0,043834175	PAPSS2	e 5'-phosp	ein-coding	th protein	G00000198682
2255	0,030535961	0,0380749	0,206386943	1,4062497	4,243630334	2,837380601	0,194115	0,861884752	0,195297645	0,773160562	FGF10	st growth f	ein-coding	th protein	G00000070193
50836	0,041058161	0,03813961	0,206376473	-1,827258	0,446595769	2,273854069	0,813108	0,410692796	0,874966134	-0,193181964	TAS2R8	receptor m	ein-coding	th protein	G00000121314
1576	0,022697372	0,03817643	0,206485486	0,7093641	4,154599015	3,445234886	0,211499	0,985118951	0,862297829	-0,109814157	CYP3A4	amily 3 sub	ein-coding	th protein	G00000160868
282566	0,011220339	0,03819726	0,206507977	-3,605107	2,300399422	5,905506197	0,001752	-5,255205986	0,092619712	-0,650300211	LINC00515	on-protein	n-coding R	long non-coding	
57165	0,023502339	0,03824672	0,206594999	0,8313215	7,782507045	6,951185568	0,968555	-0,00557424	0,453211199	-0,195958462	GJC2	on protein	ein-coding	th protein	G00000198835
29893	0,040490389	0,03830509	0,206639949	0,5426159	8,46920606	7,926590136	0,003547	0,291596309	0,09546847	0,315961845	PSMC3IP	interacting	ein-coding	th protein	G00000131470
90134	0,041806468	0,03831437	0,206600014	-0,589811	3,322236814	3,912047874	0,394241	-0,461863173	0,464551196	-0,340014508	KCNH7	ed channel	ein-coding	th protein	G00000184611
3028	0,040560262	0,03852877	0,207124912	0,5971786	11,24025247	10,64307391	0,081461	0,227302251	0,160846095	0,311782875	HSD17B10	17-beta de	ein-coding	th protein	G00000072506
388610	0,02923842	0,03863351	0,207417903	1,329772	6,165655912	4,835883888	0,145452	0,33762276	0,030698782	0,889731778	TRNP1	ated nucle	ein-coding	th protein	G00000253368
9001	0,042199817	0,03863619	0,207342416	0,6534057	3,868971751	3,215566046	0,063061	0,503763334	0,254272661	0,288373015	HAP1	associatec	ein-coding	th protein	G00000173805
3128	0,016256517	0,03868382	0,207418297	-1,133311	11,42937888	12,56268958	0,039118	-0,479954984	0,148459355	-0,57268525	HLA-DRB6	mplex, clas	seudogen	seudogen	G00000229391
3371	0,024394394	0,03869691	0,207398684	0,8244194	7,75328934	6,928869924	0,223033	0,07647347	0,055704054	0,327071677	TNC	tenascin C	ein-coding	th protein	G00000041982
5427	0,015728635	0,03875897	0,207462018	-0,896282	8,14173737	9,038018939	0,070596	-0,32809354	0,665236354	-0,033684433	POLE2	epsilon 2,	ein-coding	th protein	G00000100479
6059	0,014738148	0,03886692	0,207680801	-0,756971	9,335779293	10,09274998	0,016093	-0,533755564	0,693287706	0,078379289	ABCE1	sette subfa	ein-coding	th protein	G00000164163
126204	0,027553566	0,03897996	0,208194997	1,0534829	4,046465451	2,992982594	0,293737	0,963158489	0,817726629	-0,187318435	NLRP13	rin domain	ein-coding	th protein	G00000173572
6920	0,041064856	0,03898876	0,208152277	-0,556111	8,058068532	8,614180001	0,675449	-0,098100023	0,309575867	-0,188347821	TCEA3	n elongatio	ein-coding	th protein	G00000204219
100131060	0,042573268	0,03899914	0,208117968	0,5591716	6,386310992	5,827139409	0,009063	0,744111386	0,470201541	-0,123355285	LINC01135	on-protein	n-coding R	long non-c	G00000234807
8455	0,016840302	0,03900823	0,208076824	-0,509414	7,522453615	8,031868077	0,240098	-0,233811216	0,887948013	0,018757176	ATRN	attractin	ein-coding	th protein	G00000088812
152137	0,042608355	0,03901867	0,208042906	0,6820922	10,8160722	10,13398002	0,128043	0,403759103	0,349843475	-0,211947712	CCDC50	domain co	ein-coding	th protein	G00000152492
117156	0,026996215	0,03904347	0,207996085	-0,894543	4,12941493	5,023957956	0,439823	-1,545426567	0,199655757	-0,410951673	SCGB3A2	in family 3	ein-coding	th protein	G00000164265
400644	0,042066661	0,03906657	0,207940271	-0,620632	5,724472674	6,345104221	0,761368	0,074827079	0,390688391	-0,196709952	LINC01443	on-protein	n-coding R	long non-c	G00000266554
126070	0,016388973	0,0390666	0,207851124	-0,609916	8,502084896	9,112001189	0,419632	-0,187420159	0,376593363	-0,190484831	ZNF440	nger prote	ein-coding	th protein	G00000171295
11163	0,012117772	0,0390898	0,207885216	-0,600414	8,113864421	8,71427873	0,124639	-0,494390583	0,19244722	-0,279847339	NUDT4	lix hydrola	ein-coding	th protein	G00000173598
3930	0,041330258	0,03917408	0,208244017	-0,552849	10,05167259	10,60452151	0,059441	-0,279713746	0,68380641	0,071603576	LBR	nin B recep	ein-coding	th protein	G00000143815
8073	0,021093303	0,03920989	0,208077186	0,5429554	12,95786859	12,41491318	0,920555	0,009111602	0,026969319	0,610762922	PTP4A2	osphatase	ein-coding	th protein	G00000184007
4644	0,032451133	0,03922249	0,207965856	-0,5488	6,846120668	7,3949206	0,380992	-0,230783723	0,303586526	-0,193367344	MYO5A	myosin VA	ein-coding	th protein	G00000197535
51734	0,039237164	0,03924294	0,207896294	0,5023726	8,439181445	7,936808879	0,095741	0,254994916	0,350648591	0,155142931	MSRB1	sulfoxide r	ein-coding	th protein	G00000198736
285382	0,012732708	0,03926191	0,207818979	-1,529083	2,859515596	4,388598137	0,170003	-1,734748633	0,099209622	0,650236822	C3orf70	3 open rea	ein-coding	th protein	G00000187068
322	0,03415625	0,03927081	0,207777309	0,5180537	8,169635028	7,651581296	0,095239	0,416202657	0,809653901	-0,037043786	APBB1	protein bin	ein-coding	th protein	G00000166313
64748	0,034364726	0,0393434	0,207806302	0,5523451	8,034525201	7,482180083	0,127948	0,439423437	0,972172521	-0,00573416	PLPPR2	l phosphat	ein-coding	th protein	G00000105520
57493	0,040090754	0,03939054	0,207789475	-0,527081	8,42721286	8,95429373	0,876842	-0,030018359	0,126959122	-0,318642414	HEG1	protein wit	ein-coding	th protein	G00000173706
93349	0,028548694	0,03944767	0,207825287	-1,091732	6,049019108	7,14075153	0,21292	-0,544449212	0,110195665	-0,375371446	SP140L	lear body p	ein-coding	th protein	G00000185404
439943	0,021624225	0,03950261	0,207761248	3,0311136	3,756511952	0,725398365	0,199892	1,728116451	0,399237838	1,119417339					
7124	0,020961167	0,03957289	0,207689932	0,577776	6,557719972	5,979943974	0,001377	0,712741297	0,021308297	0,313999839	TNF	r necrosis f	ein-coding	th protein	G00000232810
11227	0,013861243	0,03967392	0,207867839	-0,572834	7,601769554	8,174603321	0,014348	-0,486529015	0,174615686	-0,178590647	GALNT5	ylgalactos	ein-coding	th protein	G00000136542
253512	0,027136813	0,03972769	0,207885738	-0,909823	5,710068496	6,619891294	0,043507	-1,128275678	0,66982402	-0,102197755	SLC25A30	r family 25	ein-coding	th protein	G00000174032
10856	0,028161962	0,03975147	0,207834588	0,5743918	11,26086061	10,68646881	0,164196	0,217351331	0,219499593	0,258398551	RUVBL2	ike AAA AT	ein-coding	th protein	G00000183207
91948	0,043214981	0,03976145	0,207799046	2,5230621	3,101900064	0,578837987	0,815476	-0,325482386	0,364225205	1,265790749	LINC00923	on-protein	n-coding R	long non-c	G00000251209
56603	0,025665616	0,03976329	0,207721002	0,5365775	6,021378484	5,484800986	0,008287	0,430101899	0,680041237	-0,067394514	CYP26B1	mily 26 sub	ein-coding	th protein	G00000003137
54967	0,039799252	0,03978	0,207720721	1,7209521	4,268186194	2,547234066	0,681381	0,455507339	0,924219089	-0,047685917	CT55	/testis anti	ein-coding	th protein	G00000169551
11141	0,030232376	0,03981465	0,207726494	-1,239228	4,93470936	6,173937732	0,531759	-0,337836852	0,874646436	0,073069173	IL1RAPL1	ptor acces	ein-coding	th protein	G00000169306
23743	0,032456605	0,0399827	0,208252386	0,960472	4,176386249	3,215914239	0,714562	0,237442649	0,272710729	0,825094769	BHMT2	steine S-m	ein-coding	th protein	G00000132840
2060	0,026645995	0,04011875	0,208697698	-0,535491	8,71940997	9,254901235	0,118819	-0,288068623	0,964147864	-0,006963466	EPS15	or recepto	ein-coding	th protein	G00000085832
157848	0,042704386	0,04013584	0,208698977	0,9995076	5,231955491	4,232447929	0,872919	0,114396065	0,929509486	0,02282899	NKX6-3	6 homeob	ein-coding	th protein	G00000165066
23556	0,014382531	0,04021567	0,209026307	-0,821252	5,841068453	6,662320108	0,134746	-0,64053707	0,235041509	0,296842762	PIGN	lycan anch	ein-coding	th protein	G00000197563
152687	0,025486343	0,04022501	0,208987141	-0,750258	6,894346504	7,644604688	0,083043	-0,458072435	0,950411079	0,013168816	ZNF595	nger prote	ein-coding	th protein	G00000272602
146909	0,037319906	0,04024204	0,208987974	0,5899881	7,813013021	7,223024955	0,106359	0,560491068	0,751825393	0,06389085	KIF18B	amily mem	ein-coding	th protein	G00000186185
374946	0,040793527	0,04027867	0,209002966	0,9144248	5,810738968	4,896314159	0,504393	0,349998251	0,188245571	0,535895847	DRAXIN	ry axon gu	ein-coding	th protein	G00000162490
10633	0,031252217	0,04029089	0,208891318	0,7765803	7,380431864	6,603851519	0,05702	0,404325537	0,779341114	0,068643359	RASL10A	amily 10 m	ein-coding	th protein	G00000100276
401321	0,015036044	0,04035237	0,208947696	-1,190644	4,55739683	5,748040553	0,022953	-1,717315819	0,148853186	0,145751396	LINC00997	on-protein	n-coding R	long non-c	G00000281332
78990	0,018418654	0,0404313	0,208745501	-0,901527	7,9961022	8,897628843	0,05038	-0,558688467	0,810157136	-0,059802086	OTUB2	e, ubiquitin	ein-coding	th protein	G00000089723
83890	0,0285531	0,04043217	0,208663011	-1,735316	2,71525729	4,450573229	0,163853	-0,999421635	0,25041444	-0,480071854	SPATA9	genesis ass	ein-coding	th protein	G00000145757
11054	0,013752159	0,04047085	0,208688717	0,5481296	9,100471628	8,552342002	0,01508	0,304875178	0,324429509	0,152983823	OGFR	owth facto	ein-coding	th protein	G00000060491

3882	0,039209043	0,04050895	0,208711444	0,594044	4,76283129	4,168787268	0,764258	-0,091458945	0,169305356	0,387059569	KRT32	keratin 32	ein-coding	th protein	G00000108759
64288	0,026396201	0,04057318	0,208868596	-0,775111	5,581161186	6,356272123	0,705272	-0,057376341	0,014449081	-0,476761981	ZSCAN31	CAN doma	ein-coding	th protein	G00000235109
29887	0,022555306	0,04066108	0,209234118	-2,041346	4,325395887	6,366741901	0,122001	-1,861557342	0,115618763	-0,520153422	SNX10	rting nexin	ein-coding	th protein	G00000086300
6844	0,016869932	0,04081347	0,209669879	0,5194802	9,747991451	9,228511262	0,763392	-0,092153404	0,844746591	0,026469696	VAMP2	ited memb	ein-coding	th protein	G00000220205
79003	0,042137411	0,04089349	0,209646154	0,5255204	8,967678555	8,442158117	0,111948	0,342790511	0,263781671	0,165706581	MIS12	hore comp	ein-coding	th protein	G00000167842
201161	0,021405609	0,04095027	0,209676891	1,1367184	10,62387427	9,487155825	0,124964	0,186599177	0,207862589	0,493477555	CENPV	omere prot	ein-coding	th protein	G00000166582
112399	0,043612123	0,04095889	0,209634358	-0,643878	6,962345864	7,606224122	0,974059	-0,003801607	0,064036315	-0,455827656	EGLN3	ypoxia indu	ein-coding	th protein	G00000129521
445372	0,044279317	0,04096399	0,2095739	-0,7308	5,485080124	6,215879672	0,159499	-0,655778654	0,952657339	0,01206703	RIM6-TRIM	RIM34 rea	other	eadthroug	G00000258588
100128563	0,03906708	0,0410071	0,209534797	0,5493009	7,665261141	7,115960231	0,746732	-0,086973234	0,016860857	0,524835802					
10421	0,035470575	0,04100757	0,209450803	0,5222879	7,925629932	7,403342068	0,180069	0,21474005	0,976445716	0,004326158	CD2BP2	nic tail bin	ein-coding	th protein	G00000169217
55333	0,012434052	0,04105253	0,20959401	0,5302148	7,819981409	7,289766594	0,633737	0,074608426	0,844237679	0,031985833	SYNJ2BP	nin 2 bindi	ein-coding	th protein	G00000213463
284023	0,022681135	0,04111907	0,20967442	3,4264665	4,261470451	0,835003946	0,586785	0,897022695	0,311991534	1,924604406					
27345	0,021681141	0,04115972	0,209795346	-1,37158	4,815290377	6,186870371	0,054141	-0,775159507	0,944635594	0,009297152	KCNMB4	nnel subfa	ein-coding	th protein	G00000135643
22852	0,030227767	0,04118396	0,209832529	-0,678507	7,375951085	8,054457723	0,235919	-0,498502933	0,827961646	-0,056058406	ANKRD26	repeat do	ein-coding	th protein	G00000107890
7766	0,043404917	0,04127749	0,209877394	-0,706812	5,682454815	6,389266682	0,218324	-0,304495613	0,85116985	0,039505709	ZNF223	nger prote	ein-coding	th protein	G00000178386
440519	0,014816813	0,04128346	0,209821604	-1,004792	7,413670607	8,418462623	0,007208	-0,934016496	0,436478869	0,227410021	ZNF724	nger prote	other	unknown	G00000196081
375189	0,029345121	0,04140587	0,210185039	0,5262305	5,825595094	5,299364642	0,415311	0,103030384	0,018441921	0,50756463	PFN4	family me	ein-coding	th protein	G00000176732
285596	0,043862464	0,04148567	0,210245434	0,8211836	5,436882026	4,615698423	0,136626	0,552872801	0,662062546	-0,147272495	FAM153A	nce similar	ein-coding	th protein	G00000170074
54361	0,032845564	0,04152807	0,21037427	0,979992	4,816512632	3,836520615	0,23717	-0,256986191	0,183989281	0,49611902	WNT4	amily mem	ein-coding	th protein	G00000162552
645010	0,044907298	0,04154725	0,210299404	0,6794179	4,629692755	3,950274806	0,290571	0,535535364	0,296595271	0,245246044					
220074	0,015521301	0,04156133	0,210284719	-0,708319	5,33115391	6,03947313	0,298087	-0,368649454	0,114676418	-0,329748322	LRTOMT	d O-methy	ein-coding	th protein	G00000184154
23683	0,022527767	0,04156647	0,210139048	-0,694234	7,238268986	7,9325032	0,038118	-0,708701871	0,298004116	0,234823129	PRKD3	tein kinase	ein-coding	th protein	G00000115825
4440	0,015404666	0,04167872	0,210277383	0,6932738	6,450914774	5,757640979	0,176986	0,436870902	0,751036614	-0,070798578	MSI1	NA binding	ein-coding	th protein	G00000135097
2038	0,042655497	0,04171497	0,210117904	0,7067579	4,770890382	4,064132501	0,455709	0,052852522	0,024963435	0,804775258	EPB42	embrane p	ein-coding	th protein	G00000166947
51651	0,030421547	0,04175732	0,210245732	0,6827259	14,71302626	14,03030037	0,075029	0,44308225	0,087248426	0,411611971	PTRH2	l-tRNA hyd	ein-coding	th protein	G00000141378
7098	0,02913687	0,0418099	0,210339492	-1,208574	4,932714253	6,14128864	0,441386	-0,273207825	0,082998485	-0,933440091	TLR3	like recept	ein-coding	th protein	G00000164342
392490	0,013742725	0,04181499	0,210279649	1,4865498	9,542050741	8,055500982	0,26169	0,733571555	0,495191226	-0,035879982					
134510	0,014170392	0,04184113	0,210325724	0,6607901	7,829383236	7,168593157	0,012371	0,275356513	0,233112656	0,239676408	UBLCP1	containin	ein-coding	th protein	G00000164332
3897	0,045180612	0,04186374	0,210354007	-0,698468	7,852807676	8,551275962	0,070131	-0,498890105	0,193568549	-0,311574094	L1CAM	adhesion m	ein-coding	th protein	G00000198910
84707	0,041652303	0,04189946	0,210448102	0,9412666	10,92022776	9,978961158	0,135964	0,52357831	0,053394727	0,640907664	BEX2	pressed X-	ein-coding	th protein	G00000133134
5648	0,016081508	0,0419269	0,210330073	0,5932133	4,594561588	4,001348296	0,028239	1,026689203	0,142559821	-0,523769299	MASP1	g lectin ser	ein-coding	th protein	G00000127241
114796	0,02891383	0,0419297	0,210173862	-0,639001	6,792333251	7,431333956	0,159518	-0,317708304	0,635926577	-0,050616406	PSMG3-AS1	nse RNA 1	n-coding R	long non-c	G00000230487
100131129	0,014529483	0,04214734	0,210753063	3,5084233	4,278512481	0,770089134	0,131185	1,356795562	0,141421914	1,721360095					
284185	0,045465571	0,04220805	0,210886359	0,6954414	7,641048641	6,945607206	0,091142	0,476958201	0,149240805	0,355444592	LINC00482	on-protein	n-coding R	long non-c	G00000185168
157378	0,024792118	0,04227303	0,211040729	-0,880485	7,8184494	8,69893417	0,004824	-0,363086361	0,81960285	-0,024784151	TMEM65	mbrane pr	ein-coding	th protein	G00000164983
449520	0,043181857	0,04244213	0,211714255	0,7254118	5,373110128	4,647698333	0,151037	0,512119611	0,89235866	0,027046381	GGNBP1	nding prote	pseudogen	pseudogen	G00000204188
440518	0,013376221	0,04246223	0,211644054	0,5877494	5,667651375	5,079902009	0,254864	0,396254581	0,75950053	0,080401259	GOLGA2P9	A2 pseudo	pseudogen	pseudogen	G00000269332
138151	0,030006704	0,0425486	0,211904038	0,698068	8,455104495	7,757036486	0,011875	0,347489444	0,147913598	0,400718188	NACC2	family mer	ein-coding	th protein	G00000148411
8089	0,026623508	0,04257907	0,211885376	-0,692849	9,015632901	9,708482184	0,25056	-0,334193016	0,576244189	0,077465195	YEATS4	omain cont	ein-coding	th protein	G00000127337
6288	0,026080441	0,04258136	0,211811654	-0,591554	8,559639641	9,151193595	0,95599	0,007107504	0,019799964	0,434647359	SAA1	um amyloi	ein-coding	th protein	G00000173432
55669	0,026949787	0,0426571	0,212018098	-0,675763	7,657751259	8,33351409	0,287379	-0,384297164	0,837721886	-0,040431841	MFN1	mitofusin 1	ein-coding	th protein	G00000171109
27033	0,01522801	0,04270707	0,212181339	0,6117343	5,997078986	5,38534464	0,324169	0,329328839	0,916899174	0,025052603	ZBTB32	BTB domai	ein-coding	th protein	G00000011590
100130934	0,031567085	0,04289229	0,2127602	0,7272089	4,433515431	3,706306543	0,201645	0,634873461	0,842272708	0,04869706	ZNF663P	rotein 663,	pseudogen	pseudogen	G00000215452
643529	0,025031212	0,04290973	0,212761509	0,5193642	5,188859367	4,669495186	0,168174	0,896488234	0,701020037	-0,198416706	LINC00865	on-protein	n-coding R	long non-c	G00000232229
148741	0,017186432	0,04298088	0,213029026	1,2952736	4,108786428	2,813512871	0,563796	0,2323044	0,190788312	0,640779556	ANKRD35	repeat do	ein-coding	th protein	G00000198483
25924	0,038492629	0,04327291	0,214048148	0,6171593	4,678056775	4,06089748	0,859515	0,055325938	0,575513236	0,189256612	MYRIP	d Rab inte	ein-coding	th protein	G00000170011
387266	0,016489262	0,04329696	0,213996164	-2,022926	4,815393542	6,838319288	0,134688	-1,247195672	0,164395613	-0,40287253	KRTAP5-3	sociated p	ein-coding	th protein	G00000196224
6525	0,027392013	0,0433009	0,213930282	0,7799774	8,102156923	7,322179506	0,120691	0,364582454	0,130665353	0,469277561	SMTN	smootheli	ein-coding	th protein	G00000183963
420	0,045562666	0,04340041	0,214165666	-0,956837	2,957648378	3,914485747	0,43413	-0,232984191	0,591049375	-0,185227728	ART4	ase 4 (Do	ein-coding	th protein	G00000111339
246122	0,018487922	0,04356699	0,2146456	-2,883699	1,333023314	4,216722631	0,413575	-0,404351164	0,190922176	-0,712381534	TTTY7	t, Y-linked	n-coding R	long non-c	G00000147753
4111	0,026769135	0,04357861	0,214617453	1,970791	4,954860132	2,984069131	0,340567	1,743840299	0,972405372	0,057011117	MAGEA12	amily mem	ein-coding	th protein	G00000213401
55435	0,046571956	0,04361816	0,214471144	0,5253121	7,904304441	7,378992319	0,713312	-0,113521294	0,118661047	0,43078977	AP1AR	mplex 1 as	ein-coding	th protein	G00000138660
3429	0,037331124	0,0436289	0,214438813	-0,743497	12,4255062	13,16900332	0,768669	-0,146250642	0,574144863	-0,236029298	IFI27	pha induci	ein-coding	th protein	G00000165949
3736	0,025873545	0,04365308	0,214387507	1,1844454	4,215819308	3,031373915	0,084284	1,094289817	0,821243724	-0,115336924	KCNA1	ed channel	ein-coding	th protein	G00000111262
462	0,023317071	0,0437417	0,214737614	1,0450567	4,935700249	3,890643593	0,150853	0,505692013	0,063790365	0,524901957	SERPINC1	amily C me	ein-coding	th protein	G00000117601
25911	0,017673491	0,04374996	0,21469306	0,5498715	9,249583274	8,699711801	0,707283	0,056611374	0,659466696	0,048743471	DPCD	ary dyskin	ein-coding	th protein	G00000166171
164633	0,026336117	0,04378028	0,214756776	-0,518268	4,746201126	5,264469512	0,377874	0,243621118	0,071395993	-0,567101273	CABP7	binding p	ein-coding	th protein	G00000100314
84101	0,024555154	0,04380139	0,214775244	0,8102748	4,236243298	3,425968524	0,595816	0,400008819	0,480677514	0,5272154	USP44	specific pe	ein-coding	th protein	G00000136014
8574	0,046920218	0,04381163	0,214740428	-0,511876	10,18362628	10,69550274	0,356929	-0,155967296	0,586164242	-0,080840126	AKR7A2	ctase famil	ein-coding	th protein	G00000053371

6737	0,039219221	0,04384238	0,214721244	-0,508232	5,955718325	6,463950589	0,022602	-0,359635617	0,18361376	-0,171532398	TRIM21	motif cont	ein-coding	th protein	G00000132109
653712	0,047137314	0,04385648	0,214620553	1,3504149	4,585854564	3,235439646	0,333514	0,611655542	0,189300589	0,878594554					
10589	0,014064919	0,04402273	0,214924628	0,7131698	12,89857828	12,18540849	0,46396	0,099126985	0,085607069	0,490016126	DRAP1	sociated pr	ein-coding	th protein	G00000175550
100169750	0,045649641	0,04407053	0,21498851	-0,772232	5,686968815	6,459201254	0,063102	-0,564907966	0,157071998	0,427729554	PRINS	protein cod	n-coding R	long non-coding	
5713	0,035359581	0,04417026	0,215220722	0,6395772	11,41558114	10,77600395	0,302721	0,195825312	0,198533765	0,345797447	PSMD7	6S subunit,	ein-coding	th protein	G00000103035
388436	0,047010033	0,0442529	0,215284647	0,6049236	6,110860915	5,505937306	0,040965	0,691038554	0,46624204	-0,167417193					
8630	0,045488085	0,04427029	0,215284678	0,9736148	4,871696884	3,898082052	0,257591	0,411066592	0,050639624	0,962886778	HSD17B6	17-beta de	ein-coding	th protein	G00000025423
266747	0,01977499	0,04434469	0,215139651	0,5016223	4,829536997	4,327914702	0,290831	0,353198917	0,89543474	0,039486272	RGL4	de dissocia	ein-coding	th protein	G00000159496
64167	0,033178959	0,04435766	0,215118318	-0,552644	5,861870276	6,414514491	0,171071	-0,10052154	0,071459839	-0,253689952	ERAP2	ticulum am	ein-coding	th protein	G00000164308
342	0,03850124	0,04436791	0,215083806	-0,588223	9,208897411	9,797120815	0,017699	-0,466487266	0,626024357	0,094609768	APOC1P1	tein C1 pse	seudogen	seudogen	G00000214855
55679	0,018575814	0,04443172	0,215224695	0,6127285	7,496678809	6,883950309	0,029645	0,281958544	0,383283381	0,161755154	LIMS2	er domain	ein-coding	th protein	G00000072163
196403	0,047658835	0,04448961	0,215168512	0,5203886	6,193041977	5,672653372	0,035829	0,210003843	0,478772085	0,110430729	DTX3	3 ubiquitin	ein-coding	th protein	G00000178498
1992	0,033676998	0,04459104	0,215406709	-0,629368	10,86950771	11,49887572	0,389584	-0,122000867	0,11777358	-0,41222232	SERPINB1	amily B me	ein-coding	th protein	G00000021355
100131096	0,035915222	0,0446354	0,215452952	-1,074583	6,372641488	7,44722437	0,357725	-0,252920898	0,107361239	-0,44816983	NRC6C-AS	C antisense	n-coding R	long non-c	G00000204282
5978	0,037777874	0,04464403	0,215410671	-0,99476	7,554648101	8,549408434	0,169479	-0,514375543	0,330578035	-0,25174366	REST	ng transcrip	ein-coding	th protein	G00000084093
7791	0,027871425	0,04467025	0,215453266	0,6153841	10,05124295	9,435858892	0,522174	0,121403368	0,307151461	0,215535217	ZYX	zyxin	ein-coding	th protein	G00000159840
83853	0,047920424	0,04484054	0,215854341	-0,701317	3,882053672	4,583370274	0,424558	-0,337318327	0,183435591	0,485632663	ROPN1L	ociated tail	ein-coding	th protein	G00000145491
81696	0,045729821	0,04490021	0,216057602	-0,666354	3,614804569	4,281159035	0,84047	0,142856572	0,220968471	-0,653087626	OR5V1	amily 5 sub	ein-coding	th protein	G00000243729
4360	0,047870623	0,04490123	0,215978582	1,543384	2,266395013	0,723010982	0,125559	1,35260438	0,608481759	0,329704735	MRC1	e receptor,	ein-coding	th protein	G00000260314
2554	0,019641785	0,04493107	0,215954408	0,6431721	3,953932106	3,310760049	0,039743	1,027788379	0,728258702	-0,115118774	GABRA1	cid type A	ein-coding	th protein	G00000022355
9046	0,046620714	0,04493504	0,215889713	0,5663834	5,236863977	4,670480607	0,384354	0,159949417	0,399467992	0,178039106	DOK2	king prote	ein-coding	th protein	G00000147443
10389	0,033110755	0,04497362	0,215740315	0,6884146	6,008171526	5,31975694	0,786677	0,077572387	0,663601548	0,092701715	SCML2	nidleg-like	ein-coding	th protein	G00000102098
4858	0,034114237	0,04502488	0,215735533	0,5059852	6,825355954	6,319370798	0,085436	0,585957754	0,679319405	-0,063546834	NOVA2	ative splici	ein-coding	th protein	G00000104967
80864	0,019410213	0,04503664	0,215708431	0,5315531	10,3721438	9,840590737	0,001916	0,521421202	0,245912357	0,20252478	EGFL8	domain m	ein-coding	th protein	G00000241404
54512	0,015662238	0,0450889	0,215791864	0,5226523	10,77217946	10,24952719	0,201734	0,153760755	0,361971011	0,1392975	EXOSC4	me compo	ein-coding	th protein	G00000178896
6990	0,037145988	0,04509074	0,21571732	0,5687515	9,014091927	8,445340453	0,053913	0,465230782	0,734612269	0,060220492	DYNLT3	ht chain Tc	ein-coding	th protein	G00000165169
22920	0,037650755	0,04518665	0,215842687	-0,635971	6,57869921	7,214670427	0,07056	-0,49414814	0,46817945	-0,178044542	KIFAP3	ssociated p	ein-coding	th protein	G00000075945
1595	0,023741958	0,04531823	0,216054586	0,6501265	11,52131405	10,87118753	0,454792	0,271120543	0,457814035	-0,167397468	CYP51A1	mily 51 sub	ein-coding	th protein	G00000001630
374897	0,014266579	0,04532026	0,21598112	0,5030456	6,111138671	5,608093114	0,062416	0,452252564	0,726827272	-0,066166584	SBSN	suprabasin	ein-coding	th protein	G00000189001
100128885	0,042225027	0,04539667	0,216178912	-2,510864	0,68328285	3,194146988	0,213119	-1,384149161	0,128970162	-1,809997827					
29926	0,044469488	0,0456003	0,216731991	0,5956955	9,46351223	8,867816698	0,054527	0,454549782	0,289395472	0,232327971	GMPPA	se pyropho	ein-coding	th protein	G00000144591
8507	0,022879065	0,0456722	0,216824114	-0,613221	9,712452044	10,3256732	0,135836	-0,210540898	0,126002344	-0,36325961	ENC1	mal-neural	ein-coding	th protein	G00000171617
285943	0,029574497	0,04571825	0,216959564	1,0789033	4,627881745	3,548978461	0,774175	0,16436279	0,032759911	0,625993149	HOXA-AS2	ster antise	n-coding R	long non-c	G00000253552
56938	0,046382383	0,04577082	0,217042732	-0,598526	7,480901907	8,079428288	0,07083	-0,576327581	0,02855789	0,554225013	ARNTL2	eptor nucle	ein-coding	th protein	G00000029153
80221	0,015558565	0,04585005	0,217168991	-0,522473	6,83166783	7,354140622	0,115467	-0,294305187	0,295454916	-0,164869865	ACSF2	hetase fam	ein-coding	th protein	G00000167107
84944	0,046116678	0,04590174	0,217247672	-0,68141	5,387034458	6,068444376	0,147936	0,202368426	0,173517501	-0,289641287	MAEL	atogenic tr	ein-coding	th protein	G00000143194
55359	0,015356761	0,0459269	0,217283725	-0,67407	4,842935074	5,517005241	0,373555	-0,26612385	0,560253665	-0,109904289	STYK1	onine/tyros	ein-coding	th protein	G00000060140
5300	0,038222638	0,04594911	0,21730579	0,522893	10,35516796	9,832274971	0,117171	0,219987727	0,528988647	0,116363121	PIN1	s isomeras	ein-coding	th protein	G00000127445
7075	0,048573121	0,0460596	0,217662098	0,553735	4,879888217	4,326153245	0,114006	0,558269177	0,633936209	0,102479106	TIE1	oglobulin	ein-coding	th protein	G00000066056
9562	0,018124256	0,04611399	0,217752998	-0,557733	7,381313636	7,939046953	0,046483	-0,301673211	0,725524438	0,05809496	MINPP1	olyphosph	ein-coding	th protein	G00000107789
257019	0,042617653	0,04613922	0,217623222	-0,514542	5,059059911	5,573601953	0,786184	0,073834975	0,223405189	-0,403890515	FRMD3	omain cont	ein-coding	th protein	G00000172159
391	0,038694313	0,04617426	0,217622733	0,5252157	10,68939692	10,16418119	0,527161	0,149093395	0,452460676	0,147020043	RHOG	log family r	ein-coding	th protein	G00000177105
574036	0,04731006	0,0461778	0,217556644	0,5933968	8,505342801	7,911945976	0,100611	0,53855021	0,511539794	0,183985486	ERTAD4-AS	4 antisens	n-coding R	long non-c	G00000203706
162073	0,024800939	0,04622397	0,217691366	-1,078724	9,603177285	10,68190152	0,300117	-0,439950614	0,10280402	-0,257126582	ITPRIPL2	te recepto	ein-coding	th protein	G00000205730
23508	0,028797138	0,04623697	0,217669828	0,5195568	5,150553116	4,630996309	0,380093	-0,20200305	0,065961201	0,43613568	TTC9	ptide repe	ein-coding	th protein	G00000133985
1830	0,033142816	0,0462483	0,217640413	0,6304495	4,619446934	3,988997435	0,191292	0,80749239	0,899020491	0,063030112	DSG3	esmoglein	ein-coding	th protein	G00000134757
9282	0,015736306	0,04625114	0,217571129	-0,731276	8,535643972	9,26692005	0,066544	-0,284537541	0,316060108	-0,225248386	MED14	complex s	ein-coding	th protein	G00000180182
641928	0,016987413	0,04626454	0,217551558	-1,52056	4,115848199	5,636408649	0,022	-0,909805715	0,64310928	-0,203601201					
145497	0,03961465	0,04629619	0,217535176	0,6674518	4,400850483	3,73339864	0,469789	0,210393995	0,062040498	0,364110006	LRRC74A	repeat cor	ein-coding	th protein	G00000100565
400682	0,047959728	0,04629711	0,217457015	-0,627778	4,190200778	4,817979025	0,640312	0,366869559	0,473212094	-0,581487876					
64838	0,046573502	0,04630512	0,21741221	-0,749285	7,301764183	8,051048752	0,173591	-0,405010719	0,648945038	0,115722121	FNDC4	e III doma	ein-coding	th protein	G00000115226
79094	0,022445774	0,0463242	0,217419347	0,726088	7,184842997	6,458755005	0,120818	0,393689577	0,132135124	0,443211803	CHAC1	gamma-gl	ein-coding	th protein	G00000128965
115708	0,042237335	0,04644582	0,217660268	0,5353029	8,272391338	7,737088417	0,293132	0,200321743	0,704460846	0,079033289	TRMT61A	thyltransfe	ein-coding	th protein	G00000166166
150221	0,049184959	0,04649098	0,21778952	0,9883955	5,704570964	4,71617549	0,905156	0,06244074	0,290717999	0,483815644	RIMBP3C	inding pro	ein-coding	th protein	G00000183246
56654	0,025260743	0,04663578	0,218055452	0,8705082	9,724830683	8,854322527	0,375301	0,201362108	0,138116702	0,515389609	NPDC1	, different	ein-coding	th protein	G00000107281
22870	0,017323558	0,0467005	0,218111047	0,8257243	9,128409977	8,302685679	0,00655	0,235451218	0,166668161	0,403461025	PPP6R1	tase 6 reg	ein-coding	th protein	G00000105063
389643	0,024709225	0,0467026	0,218038651	0,6250451	5,060741103	4,435696021	0,175054	0,302332994	0,392226536	0,171759213	NUGGC	germinal c	ein-coding	th protein	G00000189233
90141	0,028633759	0,04675787	0,218132274	0,5241398	9,226487723	8,702347963	0,137692	0,182602852	0,939852221	-0,012377024	EFCAB11	ium bindin	ein-coding	th protein	G00000140025
7542	0,020323279	0,04689323	0,218352512	0,7642149	9,634040748	8,869825865	0,047957	0,413151657	0,500561379	0,164143729	ZFPL1	ger protei	ein-coding	th protein	G00000162300

4072	0,044903103	0,04692836	0,218188003	-0,51195	7,699084545	8,211034901	0,200002	-0,1322461	0,125856255	-0,341657198	EPCAM	ell adhesio	ein-coding	th protein G00000119888
4620	0,048136898	0,04696813	0,218290957	0,6691592	4,117902311	3,448743101	0,104368	0,504618547	0,280515157	0,215611858	MYH2	in heavy c	ein-coding	th protein G00000125414
9865	0,050147446	0,04715722	0,218759359	1,8450237	4,548565444	2,703541695	0,271399	1,81899232	0,852216747	-0,268522693	TRIL	r with leuci	ein-coding	th protein G00000255690
388650	0,031436089	0,04724793	0,21893415	0,5298873	7,841308675	7,311421419	0,318351	0,130662467	0,27591649	0,21370404	FAM69A	ence simila	ein-coding	th protein G00000154511
6947	0,049411334	0,04725377	0,218797497	-0,887046	4,173287366	5,060333791	0,407122	-0,218560121	0,473037177	-0,24826717	TCN1	nscobalam	ein-coding	th protein G00000134827
92552	0,019149544	0,04725878	0,218738951	-1,864805	2,575324892	4,44012996	0,086977	-2,178866859	0,918997485	-0,097564138	ATXN3L	ataxin 3 like	ein-coding	th protein G00000123594
5163	0,040838245	0,04737745	0,219124454	-0,634693	9,07329685	9,707989739	0,109089	-0,28802909	0,191366597	-0,338630693	PDK1	ehydrogena	ein-coding	th protein G00000152256
57823	0,037375905	0,04741334	0,21904502	-0,52081	5,773009196	6,293818908	0,993717	0,001788643	0,137149231	-0,343964866	SLAMF7	family mer	ein-coding	th protein G00000026751
100129373	0,045621107	0,04748912	0,218986717	-0,973822	3,882696133	4,856518262	0,086838	-1,570393734	0,599353038	-0,29009435				
55509	0,030591467	0,04760976	0,2193797	0,5107392	8,619059544	8,108320305	0,088686	0,163767825	0,05803715	0,499716684	BATF3	ATF-like tr	ein-coding	th protein G00000123685
91752	0,036826208	0,04763941	0,219353114	0,5243737	6,305427067	5,781053381	0,067523	0,390738517	0,625850531	-0,090478771	ZNF804A	nger protei	ein-coding	th protein G00000170396
6581	0,02963896	0,04768293	0,219390373	0,5643002	6,632147562	6,067847349	0,138951	0,293848827	0,017324863	0,453428149	SLC22A3	er family 2	ein-coding	th protein G00000146477
341152	0,040904614	0,04769676	0,219291071	0,6959732	6,097419666	5,401446423	0,033852	0,76693437	0,531208158	-0,160869017	OR2AT4	amily 2 sub	ein-coding	th protein G00000171561
374355	0,043725305	0,04771754	0,219142597	1,5637587	1,997634923	0,433876205	0,195546	1,333021022	0,422649731	-0,433876205	CCDC172	domain cor	ein-coding	th protein G00000182645
645104	0,039350554	0,04785458	0,219121975	0,6869825	5,059523953	4,372541448	0,618767	0,300910938	0,662782688	0,202789656	CLRN2	clarin 2	ein-coding	th protein G00000249581
157777	0,01603878	0,04786264	0,219077912	-2,141292	0,97979087	3,121082947	0,921624	-0,06103126	0,388859749	-0,58700926	MCMDC2	aintenance	ein-coding	th protein G00000178460
182	0,040919122	0,04795368	0,219170628	0,5668733	7,316647203	6,749773929	0,333181	0,160477647	0,694704255	0,04992774	JAG1	jagged 1	ein-coding	th protein G00000101384
54551	0,02556131	0,0479955	0,219280865	-3,236316	0,285562321	3,521878231	0,247398	-2,37432805	0,679463235	-0,772473669	MAGEL2	family men	ein-coding	th protein G00000254585
79635	0,02723269	0,04803483	0,219217961	-1,131822	4,291858121	5,423679741	0,189706	-0,53373798	0,608713377	-0,113624116	CCDC121	domain cor	ein-coding	th protein G00000176714
29992	0,036989616	0,04815773	0,219536153	0,7611659	5,341219729	4,580053852	0,846829	0,068230002	0,010590469	0,723008291	PILRA	bin like typ	ein-coding	th protein G00000085514
6886	0,049145516	0,04821695	0,21964445	0,7141897	6,330026966	5,615837254	0,012476	0,692097089	0,79073697	-0,060629822	TAL1	ctor 1, eryt	ein-coding	th protein G00000162367
54464	0,029829656	0,04821932	0,219574501	-0,638972	7,150444274	7,789415789	0,089979	-0,257327635	0,405243737	-0,18531273	XRN1	xoribonucl	ein-coding	th protein G00000114127
2535	0,020898463	0,04822841	0,219535155	0,5421161	11,3903159	10,8481998	0,392743	0,171831139	0,088574723	0,44231525	FZD2	d class rece	ein-coding	th protein G00000180340
100128893	0,036124945	0,04836118	0,21973574	-1,118312	5,08859256	6,206904933	0,301643	-0,40790474	0,08414956	-0,46551054	GATA6-AS1	nse RNA 1 (	n-coding R	long non-c G00000266010
1747	0,041668764	0,04836904	0,219529856	0,5851606	5,585520073	5,000359481	0,519145	0,326417024	0,541907047	-0,120732219	DLX3	less home	ein-coding	th protein G00000064195
151827	0,048016808	0,04841811	0,219591634	-0,952814	4,644377153	5,597190934	0,186488	-0,671511367	0,332608434	0,25488968	LRRC34	n repeat co	ein-coding	th protein G00000171757
6557	0,038370001	0,04843797	0,21952097	1,1312799	3,499309775	2,368029834	0,149308	0,736035525	0,549980817	0,297974753	SLC12A1	er family 1	ein-coding	th protein G00000074803
11047	0,024491624	0,0484584	0,219533205	0,518056	10,91911606	10,40106009	0,935883	-0,012466035	0,284014337	0,190394222	ADRM1	regulating r	ein-coding	th protein G00000130706
51016	0,028193659	0,0484911	0,21960106	0,5021297	10,38803894	9,885909209	0,145097	0,24637628	0,119173938	0,34090401	EMC9	protein cor	ein-coding	th protein G00000100908
100381270	0,043660486	0,04853602	0,219643845	0,5193732	10,72941955	10,2100463	0,240882	0,550068713	0,403214778	-0,19089265	ZBED6	BED-type c	ein-coding	th protein G00000257315
400451	0,022904631	0,04854344	0,219597194	-0,725619	6,423444198	7,149062721	0,092194	-0,744001329	0,76097941	-0,03804346	FAM174B	nce similar	ein-coding	th protein G00000185442
119694	0,015861749	0,04857567	0,219582583	-3,079195	0	3,079195315	0,620428	-0,86965301	0,77477251	-0,464785784	OR51F2	amily 51 su	ein-coding	th protein G00000176925
89927	0,051414366	0,048627	0,219654292	0,6604136	7,582375094	6,92196154	0,065427	0,520003128	0,221341165	-0,332783421	C16orf45	16 open re	ein-coding	th protein G00000166780
100170841	0,034003602	0,04863023	0,219588831	0,6806541	11,8281197	11,14746563	0,059812	0,351436585	0,133541838	0,428119844	C17orf96	17 open re	ein-coding	th protein G00000273604
10780	0,048635569	0,04863066	0,219510732	-0,543511	6,265605573	6,809116906	0,092184	-0,233611167	0,524752874	-0,098709593	ZNF234	nger prote	ein-coding	th protein G00000263002
85289	0,015939783	0,04868899	0,219534011	2,9051945	2,905194464	0	0,260249	1,496455098	0,184122311	1,938735073	KRTAP4-5	sociated p	ein-coding	th protein G00000198271
389073	0,051511633	0,04870106	0,219428651	-1,189635	2,377511084	3,567145651	0,538673	-0,88926455	0,524363842	-0,279007182	C2orf80	2 open rea	ein-coding	th protein G00000188674
441268	0,01788091	0,04871333	0,219324374	0,9158629	6,865909359	5,950046468	0,033447	0,31179495	0,207701011	0,386688274				
78986	0,039846168	0,04875295	0,219423	0,6277434	6,277595776	5,649852341	0,084233	0,614232741	0,988628597	0,002853245	DUSP26	phosphata	ein-coding	th protein G00000133878
3087	0,023544876	0,04883858	0,219489371	-0,90943	5,951201322	6,860631118	0,051703	-0,723876857	0,12577014	0,268181921	HHEX	ally expres	ein-coding	th protein G00000152804
147906	0,022251878	0,0488617	0,219434036	0,6650094	6,300579086	5,635569651	0,147579	0,345309338	0,55379506	0,112873815	DACT3	g antagonis	ein-coding	th protein G00000197380
27071	0,030885128	0,04888121	0,219442087	1,3867238	3,641737887	2,255014102	0,589888	0,347469005	0,319467204	0,561978505	DAPP1	tyrosine ar	ein-coding	th protein G00000070190
51642	0,044809265	0,04888674	0,219387393	-0,502077	8,111235919	8,613312704	0,216164	-0,218436587	0,230730643	-0,183717474	MRPL48	al ribosom	ein-coding	th protein G00000175581
55763	0,030698113	0,04901581	0,219807337	-0,505352	7,767102783	8,272454971	0,117502	-0,130210157	0,598887775	-0,086353255	EXOC1	omplex con	ein-coding	th protein G00000090989
400954	0,039574941	0,04904216	0,219766379	0,5436846	5,502117073	4,958432515	0,644177	0,112659268	0,013536934	0,861050417	EML6	ubule asso	ein-coding	th protein G00000214595
55848	0,0382002	0,04906659	0,219637474	-0,580251	7,600748625	8,180999605	0,143366	-0,208941142	0,625000471	0,055452744	PLGRKT	ptor with a	ein-coding	th protein G00000107020
8638	0,017449646	0,04908621	0,219645923	0,7205771	6,623921986	5,903344841	0,002069	0,989888916	0,262884532	-0,261795622	OASL	enylate syn	ein-coding	th protein G00000135114
124222	0,050048615	0,0491577	0,21964837	-0,572172	7,142064459	7,71423688	0,173626	-0,407742829	0,495536899	-0,168941951	PAQR4	oQ recept	ein-coding	th protein G00000162073
10321	0,039103965	0,0491748	0,219645575	-0,800715	3,53746973	4,338184501	0,366904	-0,341691359	0,633752427	-0,13459161	CRISP3	ch secretor	ein-coding	th protein G00000096006
4026	0,017100806	0,04919901	0,219516189	0,5987227	9,787181002	9,188458297	0,206973	0,163672781	0,888979599	0,022317448	LPP	ferred tran	ein-coding	th protein G00000145012
4628	0,037409207	0,04922205	0,219460886	-0,60729	7,631169707	8,238459929	0,006587	-0,252070663	0,817363394	0,0445131	MYH10	n heavy ch	ein-coding	th protein G00000133026
284900	0,030043214	0,04924777	0,21949656	0,5906348	6,323139029	5,73250422	0,164927	0,426494883	0,890310946	0,033273804	TTC28-AS1	antisense	n-coding R	long non-c G00000235954
79825	0,024548048	0,04925198	0,219436367	0,5478516	5,489266381	4,941414792	0,248608	0,504005575	0,575170002	-0,13479507	EFCC1	ed-coil don	ein-coding	th protein G00000114654
10447	0,030245845	0,04940175	0,2197874	-0,914103	10,07712443	10,99122746	0,059044	-0,361461407	0,926667849	0,026869562	FAM3C	ence simila	ein-coding	th protein G00000196937
28951	0,021387714	0,04943089	0,219759164	0,5177136	9,244481609	8,726768051	0,001762	0,343518011	0,126840276	-0,314380114	TRIB2	s pseudoki	ein-coding	th protein G00000071575
84727	0,039640661	0,04946386	0,219669225	0,5689722	6,344289844	5,775317604	0,181098	0,166275517	0,227037082	0,175085209	SPSB2	domain ar	ein-coding	th protein G00000111671
50613	0,052181602	0,04954256	0,21978229	0,638909	5,823397818	5,184488796	0,367487	0,285490452	0,309259063	0,239415757	UBQLN3	ubiquilin 3	ein-coding	th protein G00000175520
160418	0,042623794	0,04954949	0,219734338	0,5011674	8,653922331	8,152754971	0,155312	0,247330773	0,461613127	-0,128253233	TMTC3	ratricopep	ein-coding	th protein G00000139324
147804	0,037322592	0,0495821	0,219800274	-0,985242	10,21474224	11,19998429	0,030316	-0,947449122	0,978488213	0,010114209	TPM3P9	osin 3 pseu	seudogen	oseudogen G00000241015

953	0,047261675	0,04959009	0,219757013	1,4277676	1,934924973	0,507157386	0,371485	0,545978737	0,184451334	0,877059052	ENTPD1	iosphate di	ein-coding	th protein	G00000138185
54981	0,043531113	0,04968995	0,219884866	-0,57103	6,662713493	7,233743798	0,847854	-0,04521228	0,618317729	0,103212261	NMRK1	iide ribosid	ein-coding	th protein	G00000106733
145483	0,050757279	0,04980881	0,219861007	0,5406356	6,906374676	6,365739052	0,780505	0,084610335	0,699172249	0,068600733	FAM161B	nce similar	ein-coding	th protein	G00000156050
112939	0,050758802	0,04991345	0,220087583	0,5150444	9,627876254	9,112831896	0,074449	0,270899582	0,61850784	0,088904743	NACC1	cumbens a	ein-coding	th protein	G00000160877
255403	0,016973984	0,04996658	0,220165103	-0,64128	6,931430117	7,572710195	0,069911	-0,369197884	0,701206555	-0,077176558	ZNF718	nger prote	ein-coding	th protein	G00000250312
