# Supplementary material for: Argonaute 2 drives miR-145-5p-dependent gene expression program in breast cancer cells
Source: Cell Death Dis. 2019 Jan 8;10(1):17. doi: 10.1038/s41419-018-1267-5 (PMC6325137; doi:10.1038/s41419-018-1267-5)
Supplement: Supplementary file 8 — Supplementary File 2 [file 41419_2018_1267_MOESM8_ESM.pdf]

entrezID	permutation p	ttest p	FDR	log2fold	mean mimic145	mean cntrl	pval(4Vs3)	log2fold(4Vs3)	pval(3Vs1)	log2fold(3Vs1)	gene
58489	0,00204856	0,00353719	0,13078049	-1,91520241	7,492661812	9,407864223	0,01782642	-1,521502	0,85726417	-0,0679232	ABHD17C
120	0,00092521	0,0005261	0,09725733	-1,26619313	6,974664128	8,240857257	0,04525323	-0,4666652	0,04881644	-0,2853258	ADD3
164	0,00525576	0,00318379	0,12803349	-0,6299136	7,514048435	8,143962036	0,07168218	-0,3947662	0,20425781	0,13065005	AP1G1
51107	0,00040038	0,0003319	0,08221823	-1,22364305	8,402638814	9,626281864	0,02067643	-0,6566915	0,41410726	-0,0889987	APH1A
160140	0,00561259	0,02256201	0,1827679	-0,96731588	3,91424612	4,881562002	0,06585915	-0,3356433	0,56839379	0,04950839	C11orf65
865	0,0036634	0,01157054	0,15748605	-0,88589664	7,919643202	8,805539844	0,06774889	-0,4419551	0,0972547	0,3289785	CBFB
55246	0,00157314	0,00763674	0,14895805	-1,12521756	8,713620163	9,838837727	0,03819783	-0,4956534	0,24777017	-0,2228947	CCDC25
124808	0,00172169	0,00143052	0,11285608	-1,05331448	8,201356479	9,254670955	0,0677357	-0,9165815	0,12596336	0,2438426	CCDC43
1021	0,00954816	0,00813081	0,15098639	-0,57825054	8,93227264	9,510523176	0,03392133	-0,3859984	0,26312626	0,13905146	CDK6
84984	0,01307643	0,02978972	0,19532783	-0,62062639	6,420887197	7,041513591	0,3336359	-0,5522555	0,0344319	0,20485496	CEP19
92675	0,00071432	0,00068837	0,10397774	-1,83830854	8,731795311	10,57010385	0,00248661	-1,4429456	0,8539363	-0,0173802	DTD1
50848	0,00079317	0,00469824	0,13822364	-1,24870979	6,793348514	8,042058305	0,01588564	-0,5067611	0,01078713	-0,772504	F11R
10447	0,03024585	0,04940175	0,2197874	-0,91410303	10,07712443	10,99122746	0,05904377	-0,3614614	0,92666785	0,02686956	FAM3C
404636	0,00062318	0,00476941	0,1386706	-1,66049638	10,14428931	11,80478569	0,013813	-1,9096595	0,15420164	-0,1912143	FAM45A
6624	0,00634266	0,01262033	0,15600287	-1,60528472	5,528738673	7,134023389	0,0335024	-0,9929808	0,16699113	-0,2776574	FSCN1
2764	0,00164469	0,00279321	0,122683	-1,83816434	7,971589552	9,809753887	0,00092754	-1,1471969	0,60572492	-0,1166299	GMFB
51280	0,00114277	0,005039	0,13993914	-1,39474848	8,660078281	10,05482676	0,0109621	-0,5498325	0,55052643	-0,0985288	GOLM1
4232	0,00259329	0,01157624	0,15739057	-0,86926533	6,039913331	6,909178664	0,00250305	-0,7956611	0,07206346	0,36187417	MEST
64419	0,00347022	0,00232997	0,12125584	-0,54516701	7,503141929	8,048308934	0,0368784	-0,1956816	0,85816973	-0,0128762	MTMR14
4644	0,03245113	0,03922249	0,20796586	-0,54879993	6,846120668	7,3949206	0,38099202	-0,2307837	0,30358653	-0,1933673	MYO5A
57447	0,0134944	0,01952358	0,17599584	-0,64559824	5,722167844	6,367766086	0,1852214	-0,2204273	0,18293568	-0,1640955	NDRG2
4697	0,00032009	9,6507E-05	0,06291199	-1,23763454	12,6425898	13,88022434	0,00303337	-0,8652383	0,05477939	-0,2215211	NDUFA4
8508	0,00643671	0,00444599	0,13870985	-0,68463138	6,380529134	7,065160513	0,19912533	-0,1455082	0,14396522	-0,195199	NIPSNAP1
4893	0,00362739	0,00999522	0,15284001	-0,56767533	9,442371994	10,01004732	0,00087658	-0,6008029	0,15431195	0,16278345	NRAS
4521	0,00423608	0,00249	0,12190132	-0,90834419	9,502556606	10,41090079	0,02123045	-0,557177	0,47132392	0,08942301	NUDT1
5281	0,00045266	0,00160301	0,11543535	-1,29005588	6,897523924	8,187579806	0,00479165	-1,0718324	0,04599524	-0,4183814	PIGF
5420	0,01795956	0,01783664	0,17020331	-0,61801369	8,994443733	9,612457425	0,00642346	-0,5040696	0,57297433	0,06715902	PODXL
5530	0,00054371	0,00277519	0,1254502	-1,3553487	8,657236134	10,01258484	0,00091747	-0,8071689	0,0110861	-0,3838394	PPP3CA
6772	0,00603262	0,02205816	0,18117469	-0,61202708	8,146640044	8,758667122	0,44386177	-0,0998132	0,0287661	-0,4909622	STAT1
23075	0,00075019	0,00568551	0,14255151	-1,41502923	7,507609912	8,922639145	0,00137865	-1,2558911	0,86694295	-0,0173781	SWAP70
56674	0,00080744	0,00410902	0,13608074	-1,89691805	7,068176974	8,965095023	0,02551351	-1,1407028	0,40471453	-0,0480991	TMEM9B
29766	0,00700919	0,01515331	0,16221981	-1,40270445	9,046821422	10,44952587	0,00089112	-1,1807319	0,69124923	-0,1076817	TMOD3
7105	0,00183341	0,00124427	0,11249254	-0,82357108	8,974282055	9,79785313	0,00795775	-0,3092479	0,04809092	-0,2144014	TSPAN6
64854	0,01525974	0,01391742	0,15946398	-1,06436658	6,577127649	7,641494228	0,06756378	-0,8423726	0,18849532	0,2760044	USP46
7525	0,01799364	0,01516219	0,1620348	-0,84244324	7,992935304	8,835378542	0,03589345	-0,6690982	0,21550917	-0,231723	YES1
51441	0,00219258	0,00749216	0,14823899	-1,28326495	8,94205328	10,22531823	0,00456816	-0,7308608	0,0481604	-0,5238912	YTHDF2
