# Supplementary material for: Argonaute 2 drives miR-145-5p-dependent gene expression program in breast cancer cells
Source: Cell Death Dis. 2019 Jan 8;10(1):17. doi: 10.1038/s41419-018-1267-5 (PMC6325137; doi:10.1038/s41419-018-1267-5)
Supplement: Supplementary file 9 — Supplementary File 3 [file 41419_2018_1267_MOESM9_ESM.pdf]

Pathways enrichment and gene ontology analyses of miR-145-5p-dependent transcripts
Genes modulated with P<0.01 were considered for this analysis

Category	Term	Lost after Ago2 depletion	Count	PValue	Genes
BIOCARTA	Sprouty regulation of tyrosine kinase signals	NO	4	1,29E-02	MAPK1, CBL, SHC1, SPRY4
KEGG_PATHWAY	Mineral absorption	YES	7	1,95E-03	MT1M, HMOX1, MT2A, MT1E, MT1B, MT1X, MT1F
KEGG_PATHWAY	Antigen processing and presentation	YES	7	2,23E-02	HLA-DRB4, HLA-DRB5, IFI30, HLA-DPA1, HLA-DPB1, CD74, HLA-DRA
KEGG_PATHWAY	Chemokine signaling pathway	NO	11	4,31E-02	MAPK1, NRAS, CCL20, CXCR6, RAP1B, SHC1, CXCL6, GRK5, GNG4, CCL28, STAT3
KEGG_PATHWAY	Cell adhesion molecules (CAMs)	YES	9	5,33E-02	F11R, ITGA6, HLA-DRB4, HLA-DRB5, CD22, HLA-DPA1, HLA-DPB1, CDH4, HLA-DRA
KEGG_PATHWAY	Fc gamma R-mediated phagocytosis	NO	6	9,56E-02	MAPK1, WASF3, CFL2, ASAP2, ARPC4, PLPP1
GOTERM_BP_DIRECT	Negative regulation of growth	YES	8	2,87E-07	ING4, MT1L, MT1M, MT2A, MT1E, MT1B, MT1X, MT1F
GOTERM_BP_DIRECT	Cellular response to zinc ion	YES	7	5,95E-06	MT1L, MT1M, MT2A, MT1E, MT1B, MT1X, MT1F
GOTERM_BP_DIRECT	Interferon-gamma-mediated signaling pathway	YES	8	2,38E-03	SP100, MT2A, HLA-DRB4, HLA-DRB5, IFI30, HLA-DPA1, HLA-DPB1, HLA-DRA
GOTERM_BP_DIRECT	IRE1-mediated unfolded protein response	NO	7	4,13E-03	TSPYL2, SYVN1, DNAJB11, TPP1, FKBP14, SHC1, WIPI1
GOTERM_BP_DIRECT	Intrinsic apoptotic signaling pathway in response to ER stress	NO	5	1,00E-02	CASP4, TRIB3, APAF1, SELENOK, ITPR1
GOTERM_BP_DIRECT	Dolichyl diphosphate biosynthetic process	YES	3	1,29E-02	DHDDS, SRD5A3, DOLK
GOTERM_BP_DIRECT	Peptidyl-tyrosine dephosphorylation	NO	8	1,44E-02	MTMR2, PTPRK, MTMR14, MTMR1, PTPRE, UBASH3B, PTPN14, DUSP22
GOTERM_BP_DIRECT	T cell receptor signaling pathway	NO	10	1,50E-02	MAPK1, PSMB6, TMEM189-UBE2V1, PSMC4, HLA-DRB4, HLA-DRB5, HLA-DPA1, HLA-DPB1, RFTN1, HLA-DRA
GOTERM_BP_DIRECT	Negative regulation of cell proliferation	NO	19	1,53E-02	PTPRK, ING4, ENPP7, KAT2B, CGRRF1, BECN1, PTPN14, CDK6, NR2E3, PLPP1, SRF, STAT3, DDR1, EIF2AK1, ADM, NUPR2, BTG3, RAPGEF2, SMARCA2
GOTERM_BP_DIRECT	Positive regulation of apoptotic process	NO	15	2,48E-02	ING4, APH1A, ZAK, ZMAT3, ARHGEF9, STK17A, ITSN1, SLIT2, NCSTN, ITGA6, ADM, HMOX1, IGF2R, DHODH, APAF1
GOTERM_BP_DIRECT	Response to drug	YES	15	2,73E-02	BECN1, LGALS1, GNA12, AK4, DPYSL2, ABCA1, AQP1, SRP19, PNP, STAT3, APOD, DHODH, TGFA, PMS2, PTCH1
GOTERM_BP_DIRECT	Intracellular protein transport	YES	12	4,41E-02	TBC1D2B, AP2B1, SEC24A, STX3, NAPG, AP1G1, TOM1L2, IPO4, CTSA, EVI5L, CD74, ADPRH
GOTERM_BP_DIRECT	Macroautophagy	NO	6	4,72E-02	ATG10, MTMR14, BECN1, TOMM20, WIPI1, VDAC1
GOTERM_BP_DIRECT	Bicellular tight junction assembly	NO	4	4,92E-02	F11R, MPP5, PATJ, SRF
GOTERM_BP_DIRECT	Small GTPase mediated signal transduction	NO	12	5,57E-02	NRAS, ARHGAP32, RABL3, RAB19, HMOX1, IFT22, RAB14, DOCK9, RAP1B, RAPGEF2, ITSN1, RHOF
GOTERM_BP_DIRECT	Signal transduction	YES	40	5,84E-02	GNA13, FGF18, SPOCK2, NMB, ARHGAP17, NR2E3, SP110, CXCL6, CD74, RGMB, TMEM9B, MYD88, CCL20, HLA-DRB4, SHC1, MR1, ZPR1, ANKDD1A, NRG2, PTPRK, GOLT1B, LGALS1, NDFIP2, CLIC2, DPYSL2, ARHGAP24, ANXA4, ITPR1, STAT3, GMFB, MAPK1, ARHGAP32, GAPVD1, ADM, TOM1L2, GRN, IGF2R, KLRC4-KLRK1, ASB2, IGFBP1

Pathways enrichment and gene ontology analyses of transcripts modulated by miR-145-5p only in the absence of Ago2
Genes modulated with P<0.01 were considered for this analysis

Category	Term	Count	%	PValue	Genes
KEGG_PATHWAY	Sphingolipid signaling pathway	4	2,7027	0,02584726	GNA13, NRAS, TNF, S1PR5
KEGG_PATHWAY	Ubiquitin mediated proteolysis	4	2,7027	0,03627511	UBE2E3, SYVN1, MAP3K1, CUL1
KEGG_PATHWAY	Protein processing in endoplasmic reticulum	4	2,7027	0,06091528	UBE2E3, EIF2AK1, SYVN1, CUL1
KEGG_PATHWAY	Proteoglycans in cancer	4	2,7027	0,09057808	NRAS, TNF, RDX, STAT3
GOTERM_BP_DIRECT	Positive regulation of NFAT protein import into nucleus	3	2,02703	0,00178325	TNF, LACRT, PPP3CA
GOTERM_BP_DIRECT	Establishment of protein localization to plasma membrane	3	2,02703	0,02985827	TNF, MPP5, RDX
GOTERM_BP_DIRECT	Cell adhesion	7	4,72973	0,07574882	CDH8, EGFL6, NUAK1, PODXL, ITGA8, PRKX, CDH11
