# Supplementary material for: Argonaute 2 drives miR-145-5p-dependent gene expression program in breast cancer cells
Source: Cell Death Dis. 2019 Jan 8;10(1):17. doi: 10.1038/s41419-018-1267-5 (PMC6325137; doi:10.1038/s41419-018-1267-5)
Supplement: Supplementary file 10 — Supplementary Table 1 [file 41419_2018_1267_MOESM10_ESM.pdf]

Supplementary Table 1: Primers used for RT-qPCR

Gene	Sequence
GOLM1 fw	CAGCGTGAAAAGCGGAATC
GOLM1 rev	TCGGCCCTGTTGTGAAATA
H3 fw	GTGAAGAAACCTCATCGTTACAGGCCTGGT
H3 rev	CTGCAAAGCACCAATAGCTGCACTCTGGAA
JAM-A fw	TCACCGCCTATCATCTGCAT
JAM-A rev	CAGCAGTGGTAGGAAAGGGA
AGO2 fw	AGGCTGCTCTAACCCTCTTG
AGO2 rev	GCTGTGCCTTGTAAAACGCT
AGO1 fw	CCGGCATCTCAAGAACACCT
AGO1 rev	CGTTCTTCACCTGCACACAC
DECR1 fw	CGGCCAGGGTTTTCTTTACT
DECR1 rev	AGCATCGCTTTTTGAAGAGG
GFM2 fw	TGACCAACTTGAGGATATTTGC
GFM2 rev	ATGGGAGGATTGATGATGGA
DOCK9 fw	GCTGCTCTTCCCTTACGATG
DOCK9 rev	GACCCTGTCGTCTCAGGATG
VDAC3 fw	GGCGTTGGTTTGAAGACCT
VDAC3 rev	GACCATGCCAAAGCCATATC
ETFB fw	GCAGAGAAGGAGAAGGTGGA
ETFB rev	CCTGTGGCCAGTCAAGAAAT
