# Supplementary material for: Argonaute 2 drives miR-145-5p-dependent gene expression program in breast cancer cells
Source: Cell Death Dis. 2019 Jan 8;10(1):17. doi: 10.1038/s41419-018-1267-5 (PMC6325137; doi:10.1038/s41419-018-1267-5)
Supplement: Supplementary file 11 — Supplementary Table 2 [file 41419_2018_1267_MOESM11_ESM.pdf]

Supplementary Table 2: Clinical characteristics of Breast Cancer patients

Gender/ Age	Tumor size (cm)	Histotype	Tumor grade	pT	pN	M	Stage	Ki67 (%)
F/76	1,6	Ductal	G3	T1	0	x	IA	80
F/68	3	Ductal	G3	T2	x	x	nd	80
F/74	4,5	Ductal	G3	T2	0	x	IIA	70
F/52	1,5	Ductal	G3	T1	0	x	IA	5
F/46	2,5	Ductal	G3	T2	0	x	IIA	90
F/66	2,8	Ductal	G3	T2	3a	x	IIIC	50
F/68	1,5	Ductal	G3	T1	0	x	IA	70
F/72	2,6	Ductal	G3	T2	1a	x	IIB	70
F/50	6	Ductal	G3	T3	1a	x	IIIA	70
F/64	3,9	Ductal	G3	T4	2a	x	III3	80
F/63	2,5	Ductal	G3	T2	3a	x	IIIC	50
